# Probabilistic Phylogenetic Inference with Insertions and Deletions

**DOI:** 10.1371/journal.pcbi.1000172

**Published:** 2008-09-19

**Authors:** Elena Rivas, Sean R. Eddy

**Affiliations:** Janelia Farm Research Campus, Howard Hughes Medical Institute, Ashburn, Virginia, United States of America; University of California Santa Cruz, United States of America

## Abstract

A fundamental task in sequence analysis is to calculate the probability of a multiple alignment given a phylogenetic tree relating the sequences and an evolutionary model describing how sequences change over time. However, the most widely used phylogenetic models only account for residue substitution events. We describe a probabilistic model of a multiple sequence alignment that accounts for insertion and deletion events in addition to substitutions, given a phylogenetic tree, using a rate matrix augmented by the gap character. Starting from a continuous Markov process, we construct a non-reversible generative (birth–death) evolutionary model for insertions and deletions. The model assumes that insertion and deletion events occur one residue at a time. We apply this model to phylogenetic tree inference by extending the program dnaml in phylip. Using standard benchmarking methods on simulated data and a new “concordance test” benchmark on real ribosomal RNA alignments, we show that the extended program dnaml *ε* improves accuracy relative to the usual approach of ignoring gaps, while retaining the computational efficiency of the Felsenstein peeling algorithm.

## Author Summary

We describe a computationally efficient method to use insertion and deletion events, in addition to substitutions, in phylogenetic inference. To date, many evolutionary models in probabilistic phylogenetic inference methods have only accounted for substitution events, not for insertions and deletions. As a result, not only do tree inference methods use less sequence information than they could, but also it has remained difficult to integrate phylogenetic modeling into sequence alignment methods (such as profiles and profile-hidden Markov models) that inherently require a model of insertion and deletion events. Therefore an important goal in the field has been to develop tractable evolutionary models of insertion/deletion events over time of sufficient accuracy to increase the resolution of phylogenetic inference methods and to increase the power of profile-based sequence homology searches. Our model offers a partial answer to this problem. We show that our model generally improves inference power in both simulated and real data and that it is easily implemented in the framework of standard inference packages with little effect on computational efficiency (we extended dnaml, in Felsenstein's popular phylip package).

## Introduction

A fundamental task in sequence analysis is calculating the probability of a multiple alignment given a phylogenetic tree relating the sequences and an evolutionary model describing how sequences change over time. This quantity is already at the heart of phylogenetic tree inference by either maximum likelihood [1] or Bayesian approaches [2]–[5]. It is also desirable to integrate evolutionary modeling into the probabilistic models widely used for other sequence analysis problems such as HMMs and SCFGs for homology search and genefinding [6]. However, whereas HMMs, SCFGs, and other sequence analysis models account for insertion and deletion events in their probabilistic framework, many widely used phylogenetic models only account for residue substitution events.

The general approach of modeling residue substitution as a continuous-time Markov process was introduced by the Jukes-Cantor model of nucleotide substitution in DNA [7] and the Dayhoff pam model of amino acid substitution in proteins [8]. It has since been extensively developed both for nucleotides [9]–[14] and amino acids [15]–[18], and extended to models of more than a single residue, such as codon to codon substitutions [19],[20] and RNA basepair to basepair substitutions [21]–[23].

Given a substitution model and a tree, one can efficiently calculate the probability of an *ungapped* multiple alignment using Felsenstein's peeling algorithm [1]. The Felsenstein algorithm scales linearly with the length of the alignment and the number of sequences. Because of its economy, it is the basis of maximum likelihood methods in many practical phylogenetic inference tools, including phylip [24], paup* [25], and others [26]–[33]. The Felsenstein algorithm is readily integrated with other probabilistic models for ungapped alignment analysis, including HMMs [14], [34]–[38] and SCFGs [21]. However, when this approach is applied to gapped multiple sequence alignments, gap characters are typically treated as missing data (an unknown residue), effectively equivalent to ignoring them.

A variety of more formal approaches for treating insertions and deletions exist [39]–[49]. The canonical model in this active area of research is the Thorne-Kishino-Felsenstein model (tkf91) [50]. tkf91 treats insertion and deletion events as a continuous-time process governed by explicit insertion and deletion rate parameters, allowing multiple insertions and deletions to accumulate at the same “site” over long times. tkf91 improved, for example, upon methods that parsimoniously assume no more than one change per site per branch, including the pioneering Bishop and Thompson pairwise alignment likelihood model that preceded tkf91 [51]. Many extensions of tkf91 have appeared 52–56, including practical applications for pairwise alignment [57],[58] and multiple alignment [59]–[62]. Several approaches based on tkf91 or related models have addressed the problem of simultaneously aligning and inferring the phylogeny of a group of related sequences [63]–[68]. In general all algorithms in this class have difficult time complexities, worst-case exponential in the number of sequences, and at least in the case of parsimony models akin to tkf91 , the problem of inferring the optimal insertion and deletion history given a tree has been formally shown to be NP-complete [47]. This complexity is inherent to the problem. Any implementation of evolutionary models that allow insertions and deletions, tkf91-based or otherwise, seeks to make approximations that make calculations tractable.

Here we are specifically concerned with the problem of calculating the probability of a *given* multiple alignment and phylogeny. A tkf91-based approach for this problem [69] used a clever approach of using ungapped columns in the alignment to constrain and subdivide the solution space (a so-called “homology structure”), but (because tkf91 is not invariant under column rearrangement) a sum over all subalignments compatible with a given homology structure is still required. As a result, though time complexity exponential in the number of sequences can be avoided in the average case where not many gaps occur, the approach remains expensive in absolute terms; a Bayesian Markov chain Monte Carlo phylogenetic inference for an alignment of 10 globins was reported to require 3 CPU hours on a 1.25 GHz G4 Apple Macintosh [69]. Although tkf91 has many desirable and realistic properties as a model of evolution, it would be advantageous to have even more computationally efficient approaches, particularly for problems where a gain in efficiency might outweigh sacrificing some of the realism of the model.

An alternative is to use a continuous-time Markov process for insertions and deletions that remains fully compatible with the Felsenstein algorithm [41],[46]. This requires an unrealistic assumption of column independence, but nonetheless, it may be better than ignoring gaps altogether. Here we further explore such models. We propose a non-reversible generative model based on a Markov process that we readily incorporated into an existing phylogenetic inference application, resulting in a gain in its accuracy.

## Results

### Premises of the Model

The originating idea is to use an extended (*K*+1)×(*K*+1) rate matrix for *K* residues (4 nucleotides or 20 amino acids) plus the gap character to describe the rate of change of a residue to a residue (substitution), a residue to a gap (deletion), and a gap to a residue (insertion) [6],[41],[46]. From this Markov process, we construct a generative model of sequence evolution that includes insertions and deletions. Several consequences flow from this, which here we discuss informally by way of introduction to the rest of the paper.

This model describes the evolution of single residues in one column of a multiple alignment, given a phylogenetic tree. The total probability of the alignment is then assumed to be an independent product of each column probability. For each column, a variant of the Felsenstein peeling algorithm recursively infers the probability of ancestral characters at each tree node, where an ancestral character is either a residue or a gap. Assuming that insertion and deletion events happen one residue at a time necessarily implies a linear “gap cost”. This is a much less satisfactory model of insertion and deletion processes than models that can assume an affine or arbitrary gap cost.

It has generally been thought that models based on a gap-extended rate matrix must be conceptually flawed, because it appears necessary to assume that all ancestral and descendant sequences fit in a fixed number of columns. This fundamentally conflicts with allowing any number of insertions and deletions to occur, and it produces a so-called “memory effect” artifact [70] in which descendants “remember” how many gap characters were present in ancestral sequences, allowing insertions up to that length and precluding longer ones. A related conceptual flaw would be treating gaps like residues, assigning a probability to a gap/gap alignment (as one would do for any residue/residue alignment), rather than recognizing that a gap/gap alignment may represent no evolutionary event at all from the standpoint of just the descendant and ancestral sequence; rather, gap/gap alignments are imposed by events that occurred in *other* sequences. For a model to be at all satisfactory, one must be able to describe a generative evolutionary model *unconditional* on any fixed sequence length, in terms of substitution, insertion and deletion events that evolve one (unaligned) sequence to another, and show how that generative process relates uniquely to the column-by-column inference algorithm that one will apply to a given multiple alignment.

Here we will develop such a generative model, by borrowing terms from a Markov process for a rate matrix extended for the gap character. The rate matrix includes the gap character, but the subsequent generative evolutionary model does not treat gaps as an extra residue. Rather, it describes evolutionary insertion and deletion (birth-death) events [71], where the evolved sequences form alignments of arbitrary length. The existence of a generative evolutionary model for unaligned sequences, and the mapping of its events to the column-by-column inference procedure, is the crucial point of differentiation between our work and previous work on efficient column-based phylogenetic inference with gap-extended rate matrices [6],[41].

Similarly, one must have a consistent way of dealing with columns that are unobserved in the alignment of extant sequences – that is, places where ancestral residues have been inserted and deleted, where alignment columns would exist if all the ancestral sequences were known in addition to just the extant sequences. Therefore the likelihood we calculate for an extant multiple alignment will be its marginal likelihood, marginalized over all possible ancestral sequences including unobserved alignment columns that left no trace in the observed alignment.

Another conceptual problem of column-based models arises if one adopts the usual practice of making the substitution process reversible (in the sense that the probability of an ancestor/descendant sequence alignment is independent of the direction of time along the branch that connects them; this is mathematically convenient for applying the “pulley principle” and using the Felsenstein peeling algorithm on unrooted trees). If one assumes reversibility for a substitution process that includes gaps, one necessarily imposes a frequency of gap characters that is constant with respect to divergence time [41]; but obviously in the limit of zero divergence time, there are no gap characters in an alignment of homologous sequences. Moreover, reversibility clearly cannot hold if insertion and deletion rates are free parameters. For example, for a nonzero insertion rate and zero deletion rate, the shorter of two homologous sequences automatically must be the ancestor. A reversible insertion/deletion model would also imply that all homologous sequences have the same expected length at all divergence times. We overcome these problems by adopting a non-reversible model that states an explicit prior length distribution for unaligned ancestral sequences (as opposed to assuming that all *K*+1 characters including gaps occur in the ancestor at the stationary frequencies of the Markov process). Because our model is non-reversible, we must always work with rooted phylogenies.

Because we map generative evolutionary model events onto an alignment column, the column is assumed to be correctly aligned phylogenetically – all aligned residues are assumed to be homologous and related only by substitution events. This means that no more than one insertion event may occur in any given column. Enforcing this assumption requires modification of the usual Felsenstein peeling algorithm to include some extra bookkeeping.

Given alignments are unlikely to be phylogenetically correct, because humans and alignment programs tend to produce aesthetically pleasing alignments that compress columns containing few residues. Importantly, for any arbitrary tree topology and any arrangement of observed residues and gap characters in an alignment column, there exists at least one possible assignment of characters to ancestral nodes that makes all extant aligned residues homologous. Therefore the problem with using phylogenetically incorrect alignments is not that the algorithm will fail altogether (as would happen if some combinations of alignments and trees were impossible), but rather that we can expect its inference ability to be degraded by forced inference of incorrect histories in phylogenetically incorrectly aligned columns. How much the overall inference is degraded by this and by the other assumptions described above is a matter for empirical testing, which we describe in the second half of the paper, after we describe the model itself.

### Solving the Markov Process for a Gap-Extended Rate Matrix Model

First, we start by solving the Markov process associated with a rate matrix extended to include a gap character. For an alphabet of *K* residues, probabilistic substitution models are defined by a *K*×*K* rate matrix *R* such that the matrix of conditional probabilities *Q_t_*(*i*,*j*) ≡ *P*(*j*|*i*,*t*) is given by
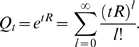
(1)


We extend this to include the gap character by augmenting the rate matrix to a (*K*+1)×(*K*+1) matrix *R^ε^* that depends on arbitrary rates of deletions *μ*≥0 and insertions *λ*≥0:
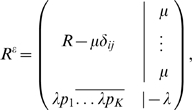
(2)where *p* = (*p*_1_,…,*p_K_*) is the distribution of inserted residues 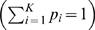, and *δ_ij_* stands for the Kronecker delta in the *K*×*K* subspace (valued one if *i* = *j* and zero otherwise). Since the extended rate matrix *R^ε^* has the property that each row adds up to zero, we can construct a model of evolution for the extended rate matrix defined as
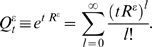
(3)At zero divergence, the probabilities of any insertion or deletion of a residue or any substitution of a residue to a different residue are all zero.

Generally, the extended conditionals 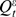 can be cast into the form,
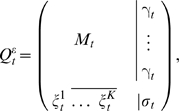
(4)with the conditions 
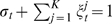
 and 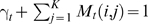, for each row *i*, and where *M_t_* is the *K*×*K* conditional substitution matrix (to be defined later).

In particular, for a reversible *K*×*K* rate matrix *R*, if we assume that the distribution of inserted residues in equation (2) is the stationary distribution associated to the reversible rate *R* (*π_i_R*(*i*,*j*) = *π_j_R*(*j*,*i*)) then one can derive the analytic expression for 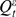 in terms of the solution for the *K*×*K* conditional matrix *Q_t_* = *e^tR^* and the rates of insertion and deletion. This particular solution for the extended conditional probabilities is given by
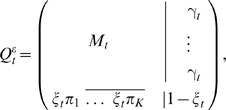
(5)where the gap-specific functions *γ_t_* and *ξ_t_* are given by
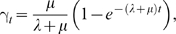
(6)

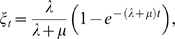
(7)when at least one of the two rates is positive. For the particular case of no insertions and deletions (*λ* = *μ* = 0) both functions are defined as identically zero.

Finally, we have to describe how to obtain the *K*×*K* conditional substitution matrix subspace *M_t_* in 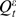 from a rate matrix *R* and the insertion and deletion rates. Generally, any biologically relevant *K*×*K* substitution rate matrix *R* must have zero as a non-degenerate eigenvalue, and at least one other negative eigenvalue. To express *M_t_* in general form, let (0,−*e*_1_,…,−*e_A_*) represent the eigenvalues of *R*, for 1≤*A*≤*K*−1, with *e_a_*>0 for 1≤*a*≤*A*. Then the standard conditional substitution probabilities could be expressed as:
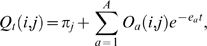
(8)where *O_a_* is a *K*×*K* matrix of real numbers specific for each unique nonzero eigenvalue. Text S1 shows how to derive the *O_a_* matrices from the similarity transformation that relates R with its diagonal form.

Now the extended conditional matrix in the substitution-only subspace *M_t_* is expressed as:
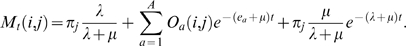
(9)If *λ* = *μ* = 0, *M_t_*(*i*,*j*) is defined as the standard substitution conditional matrix *Q_t_*(*i*,*j*) in Equation 8.

For example, the F84 model [14] is defined by the substitution rate *R*(*i*≠*j*) = *βπ_j_*+*α*Δ*_ij_*, where 
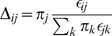
, and where the function *ε_ij_* is 1 if *i*,*j* are both either purines or pyrimidines, and 0 otherwise. The F84 rate depends on two non-negative parameters *α* and *β*, and on a stationary residue distribution *π*. The F84 rate matrix has non-zero eigenvalues *e*_1_ = *β*, and *e*_2_ = *α*+*β*. The matrix *O*_1_ is given by *O*_1_(*i*,*j*) = Δ*_ij_*−*π_j_*, and the matrix *O*_2_ is given by *O*_2_(*i*,*j*) = *δ_ij_*−Δ*_ij_*. For the gap-extended F84 model, we obtain
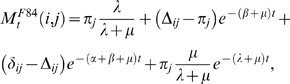
(10)which for the particular case *λ* = *μ* = 0 is defined as the original F84 model for substitutions,
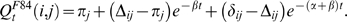
(11)


The conditional model of McGuire *et al.* [41] describes a particular solution of the extended F84 model presented here, in which the rates of insertions and deletions are constrained to satisfy the conditions *λ* = *β* and *μ* ∝ *β*.

A more detailed description of the characteristic differential equations for an arbitrary gap-augmented rate matrix and the particular solution described above is provided in Text S1. Under different assumptions, such as a reversible substitution rate matrix but using a distribution of inserted residues other than the stationary distribution for *R*, or a non-reversible substitution rate matrix, 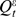 could still be obtained numerically [72].

A note about reversibility. For a reversible substitution rate matrix, the particular Markov process including gaps solved here is also reversible, as can be seen by using the marginal frequencies *π_i_λ*/(*λ*+*μ*) for a residue, and *μ*/(*λ*+*μ*) for the gap character. However, one has to distinguish between the reversibility of a Markov process and the reversibility of an evolutionary process constructed using that Markov chain. A Markov chain is said to be reversible if and only if there exists a marginal distribution that satisfies the reversibility condition [73]. However, regardless of whether the Markov process is reversible in the strict sense, when constructing an evolutionary process from it one may specify an ancestral marginal distribution other than what reversibility requires. In evolutionary models, “reversibility” is generally taken to mean that the joint probability of an ancestral and a descendant sequence is invariant regardless of which sequence is used as the ancestor and which is used as the descendant. In probabilistic inference, this latter definition of reversibility is the most relevant (for instance to invoke the pulley principle [1]). From here on in this paper we use the term reversibility in this latter (broader) sense. Thus, the evolutionary model that we construct in the next section is not reversible in this (usual) sense though the Markov chain that it is based on is reversible in the strict sense.

Up to this point, this is essentially McGuire's model [41] (with a minor generalization). That model has the conceptual problems we described in the preamble, thought to be inherent to approaches based on gap-extended rate matrices. In the next section, we show how these problems may be circumvented.

### The Generative Model

We construct the generative model as an independent product of single-event (substitution, deletion or insertion) contributions. What we will do to construct the model is to borrow terms from the conditional Markov process introduced in the previous section. We describe the probability of an insertion as proportional to *ξ_t_π_i_* in Equation 7, the probability of a deletion as proportional to *γ_t_* in Equation 6, and that of a substitution as proportional to *M_t_*(*i*,*j*) in Equation 9, but we ignore the gap to gap transition of the Markov process. This allows us to derive a generative model that describes insertions and deletions not as mere “gap character” replacements on a fixed length alignment but as true evolutionary events (births and deaths of residues).

A given sequence **x** = {*x*_1_…*x_l_*} that evolves to another sequence **y** = {*y*_1_…*y_l_*_′_} corresponds unambiguously to a pairwise alignment in which a substitution is represented by a conserved or mismatched column, a deletion by an ancestral residue aligned to a gap in the descendant sequence, and an insertion is represented by an ancestral gap aligned to a residue. Let **x**ˆ = *xˆ*_1_‥*xˆ*_L_ and **y**ˆ = *yˆ*_1_‥*yˆ*_L_ mean the aligned sequences **x** and **y**. Specifically, the probability that **y** was generated from **x** after time *t* with pairwise alignment **x**ˆ**y**ˆ that includes *s*≤*l* substitutions, (*l*−*s*) deletions, and (*l*′−*s*) insertions {I_1_…I*_l_*_′−*s*_} (where the subset of residues 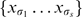 from **x** are substituted by 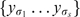 in sequence **y**) is constructed as
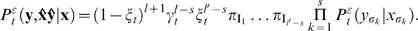
(12)The functions *γ_t_*, *ξ_t_* and 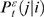 are given by the Markov model solutions (Equations 6, 7, an 9). The residue distribution *π*, is set to the stationary distribution of the substitutions rate matrix. In that way, for a reversible substitution rate matrix, this generative model is quasi-reversible (*i.e.* reversible in the substitutions subspace).

In equation (12), everything but the term (1−*ξ_t_*)*^l^* ^+1^ is borrowed from the Markov process. This extra term is responsible for having a normalized distribution, such that the sum of the contributions of all sequences **y** of all possible lengths and all possible alignments is one. The extra term accounts for the fact given a sequence **x** of length *l*, insertions can occur at (*l*+1) places. That is, at each of the (*l*+1) places that an insertion in **x** could occur, an insertion of length *z* occurs with probability 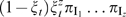 (a normalized geometric distribution).

Using the generative probability distribution (Equation 12), one can calculate the expected length (in residues without gaps) of descendant sequences originated from an ancestral sequence of length *l* after summing to all possible patterns of substitutions, insertions and deletions, that is given by (full derivation in Text S2):
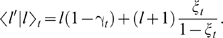
(13)


Crucially, this model does not assume that all observed sequences are generated from a preset number of aligned columns. Thus there is no “memory effect” [70]. Our model is a generative probabilistic model with a close correspondence to the birth-death description of the tkf91 model [50], (see Text S2, for more detail).

We have implemented this generative model in a computer program for evolving sequences named *ε* rate.

### The Joint Probability of a Pairwise Ancestor/Descendant Alignment

Given the *conditional* probabilities of the generative model, one wants to calculate the *joint* probability of a multiple alignment of sequences generated with the model, given a phylogenetic tree. Before doing that, let us consider the joint probability of a pairwise ancestor/descendant alignment, as a building block for a full-fledged algorithm for phylogenetic inference on multiple alignments.

Using the same notation as in the previous section, we can calculate the joint probability of the pairwise ancestor/descendant alignment (**x**ˆ,**y**ˆ) after divergence time *t* using the expression
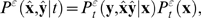
(14)where *P^ε^*(**x**) is the probability of the ancestral sequence. This distribution is a prior, not determined by the generative model. In order to have this prior factorize effectively into columns, we will assume the length of ancestral sequences follows a geometric distribution (1−*p*)*p^l^* for a sequence of length *l* with arbitrary Bernoulli frequency parameter 0<*p*<1.

The joint probability of a pairwise ancestor/descendant alignment can then be factorized as a product of terms over alignment columns as:
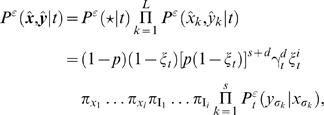
(15)where the length of the alignment l = *s*+*d*+*i*. Normalization of this joint probability requires including the extra term *P^ε^*(★|*t*) = (1−*p*)(1−*ξ_t_*) that cannot be associated to any observed column. Intuitively this term can be viewed as an extra (terminating) column (★). An analogous “extra column” term will appear in the multiple alignment case with the Felsenstein pruning algorithm.

In Text S3, we use the joint probabilities in Equation 15 to calculate other related length distributions of the model such as the length distribution for descendant sequences and the length distribution of alignment length, by summing over the other variables (marginalization). Because the model is non-reversible, the distribution of descendant sequences is different from that of ancestral sequences, and depends on the divergence time (non-stationary).

From the joint alignment probability distribution, we can also calculate the expected frequencies of insertions 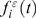 and deletions 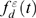 in pairwise ancestor/descendant alignments (see Text S3 for derivation). These quantities illustrate some important properties of the model. They will also be useful in making comparisons to the properties of other reversible models (see Discussion). They are,
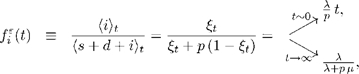
(16)

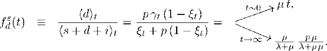
(17)


Because the model is non-reversible, 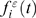 and 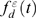 are different (note that we avoid the traditional term “indel” in this paper, because we are not treating them reversibly). When the insertion rate is zero (and the deletion rate is positive), the expected frequency of insertions is zero for all divergence times but the expected frequency of deletions is not, as one would expect. Similarly, when the deletion rate is zero (and the insertion rate is positive), the expected frequency of deletions is zero for all divergence times while the expected frequency of insertions is not. For more detail, see Text S3.

### Felsenstein's Peeling Algorithm Extended to Gaps

Felsenstein's peeling algorithm is an efficient algorithm for calculating the probability of a multiple alignment given a tree and a Markov substitution model. Extending the Felsenstein algorithm to include insertion and deletion events with the model we propose here requires four modifications. Those are: extra bookkeeping in the Felsenstein recursions to enforce that no more than one insertion occurs per column, so that all aligned residues are homologous; including a term from the prior ancestral sequence length distribution in the calculation of each individual column likelihood; including in the overall alignment likelihood the extra normalization terms collected in the “extra column” (★); and finally, marginalizing the contributions of possible ancestral residues that have left no trace in extant sequences. Otherwise, the substitution model assumed by the Felsenstein peeling algorithm is simply replaced by the generative model described in the previous section by Equation 12 which includes substitutions, insertions and deletions.

Using the notation of [6], for a given position *u* in the alignment, let *P_u_*(*L_k_*,*i*) be the probability up to node *k* given that the character (residue or gap) at node *k* is *i*. For a residue *i*, *P_u_*(*L_k_*,*i*) cannot contain any insertion only substitutions and deletions. For a gap, *P_u_*(*L_k_*,–) has to include one and only one insertion. Thus, *P_u_*(*L_k_*,–) is defined to be zero if all the nodes under *k* are gaps for that position. These probabilities are calculated recursively starting from the leaves of the binary tree as,

If node *k* is a leaf, for a residue *i*,
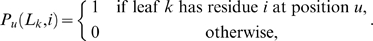
(18)for a gap,
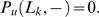
(19)


**Figure 1:**
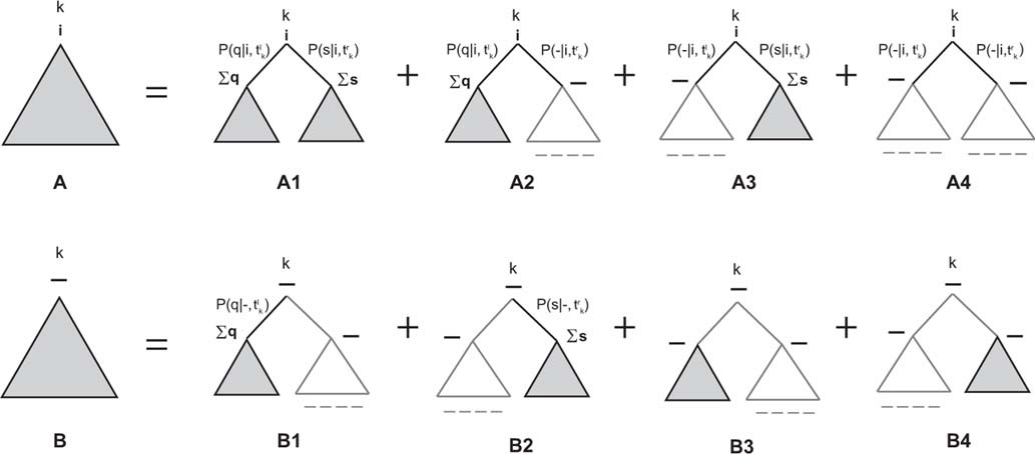
Felsenstein's peeling algorithm extended to gaps. Graphical description of the extended Felsenstein algorithm to calculate the probability of an alignment given a phylogenetic tree using the generative model for gaps presented in this work, given by Equations 20 and 21. For a given column in a multiple alignment, recursion A corresponds to the probability of a tree up to node *k* with a residue at the node (Equation 20). Recursion B corresponds to the probability of a tree up to node *k* with a gap at the node (Equation. 21). The algorithm needs to consider only evolutionarily correct events, thus recursion A includes substitutions and deletions but no insertions, and recursion B has to include one and only one insertion. In the case of having a residue *i* at node *k* (recursion A), the A1 term corresponds to the original substitution-only Felsenstein algorithm, where *q* and *s* are residues, and ∑*q* and ∑*s* stand for the sum to all possible residue substitutions. The terms A2 and A3 represent a deletion occurring for the right and left child respectively (and a substitution for the other child node). Because no insertion can occur in this recursion, there are no evolutionary events happening for descendants for the child that suffers the deletion. Thus, for a A2 or A3 term to have a contribution all nodes down to the leaves for the child that suffers the deletion have to be gaps. (We represent with gray the situation of no evolutionary event happening, and with an empty gray triangle with gaps at the bottom the situation of gaps at all nodes including the leaves for a given subtree.) The A4 term corresponds to a deletion for both children nodes, and no evolutionary event from there on. A A4 term contributes only if with all the leaves under node *k* are gaps. In the case of having a gap at node *k* (recursion B), the insertion might occur for the left or right child, which is represented by graphs B1 and B2 respectively. The insertion might instead be delayed to a node under the left or right child, which is represented by graphs B3 and B4 respectively. In all four cases, the child node for which no insertion occurs does not have any evolutionary events, and has to include all gaps at the internal nodes and leaves. The substitution, deletion and insertion probabilities are given by the generative model as per Equations 22–24, respectively.

If node *k* is not a leaf, for a residue *i*,
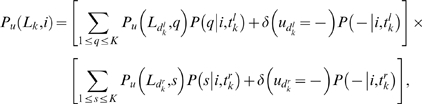
(20)for a gap,
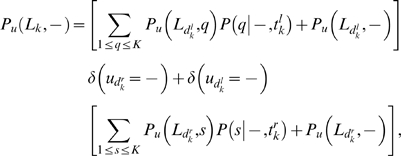
(21)where 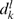 and 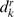 are the two daughters of node *k*, 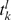, and 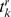 are the distances from node *k* to its left and right child respectively, and where the probabilities for the daughter nodes have already been calculated by the recursion. *u_k_* stands for the subset of leaves under node *k* for column *u*, and *u_k_* = – indicates that all leaves under node *k* are gaps for column *u*. The single-event conditional probabilities are dictated by the generative model in Equation 12 as,
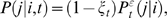
(22)

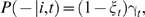
(23)

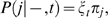
(24)for 1≤*i*,*j*≤*K*, where the functions *γ_t_*, *ξ_t_* and 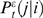 are given by the Markov model solutions (Equations 6, 7, and 9). Figure 1 shows a graphical interpretation of the Felsenstein recursions described in Equations 20 and 21.

The second modification is due to the existence of a length distribution for ancestral sequences for this model. In order to calculate the total probability of a given aligned column *P*(*u*|*T*,*R^ε^*,*p*), we need to use the probability of the sequence at the root. The total probability of site *u* is given by
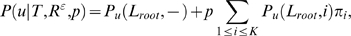
(25)where *p* is the parameter of the geometric distribution of ancestral sequences, and *π* are the residue frequencies at the root.

Third, the factorization in columns of the unconditional in length alignment distribution leaves some normalization terms that we gather together into what we think of as an “extra column” (★) contribution. Thus, when calculating the total probability of a multiple alignment as the product of *l* individual columns, there is an additional term in the equation:
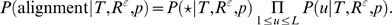
(26)The term *P*(★|*T*,*R^ε^*,*p*) = (1−*p*)*P*_★_(*L_root_*) is calculated by a similar peeling algorithm,
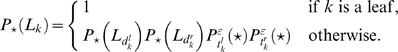
(27)where 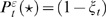.

Lastly, one could delete an ancestral residue leaving no trace in extant sequences—a column could exist but not be present in the observed alignment—therefore we are interested in calculating *P*(*L*_0_) the probability of an alignment with *L*_0_ observed columns after marginalizing the unobserved columns. The probability of an alignment with *L*_0_ observed columns and *k* unobserved columns is given by
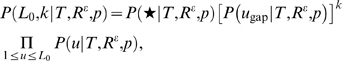
(28)where the probability of an all-gaps column *u*_gap_ is a particular case of Equation. 25. Then, for a given alignment of *L*_0_ observed columns, we marginalize to all possible unobserved columns as,
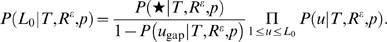
(29)This is the final expression for the probability of a given alignment of *L*_0_ columns, after marginalizing all possible unobserved ancestral residues.

This algorithm will now reproduce the results of the original Felsenstein algorithm for ungapped alignments when the parameters *λ* = *μ* = 0 (except for a geometric term which is the same for all trees, thus will not affect the maximum likelihood estimation of a tree). In addition, *λ* = *μ* = 0 are the optimal parameter choices for any ungapped multiple alignment for any given tree. Notice that for the previous statement to be true, it is crucial to have the extra term (1−*ξ_t_*) in the generative process of a substitution. Once we set *μ* = 0, the residue-residue substitution process in the presence of gaps (described by Equation 9) reaches the same asymptotic value (described by Equation 8) for any value of *λ*, including both the limit *λ* = 0 and the limit *λ* = ∞. It is the extra term that renders the total probability of any finite-length alignment to zero in the case *λ* = ∞, and makes *λ* = 0 optimal on ungapped alignments.

The extended peeling algorithm has worse-case time complexity 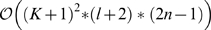 for an alphabet of size *K* and a multiple alignment of l columns and *n* sequences.

### An Implementation in phylip: dnaml*ε*

To test an application of our model, we modified the program dnaml (from the phylip package version 3.66, 4 August 2006 [24]) to use our generative model. Given a nucleotide multiple alignment, the dnaml program infers a phylogenetic tree for the sequences by maximum likelihood under an F84 rate matrix model [14]. Our modified program dnaml *ε* uses the extended F84 rate matrix given by Equations 5, 6, 7, and 10, and implements the extended Felsenstein algorithm described in the previous section.

dnaml *ε* uses the same core algorithms for maximum likelihood tree inference as the original dnaml. For every dnaml function we implemented a dnaml *ε* counterpart. In addition, dnaml *ε* optimizes the gap parameters (*λ*, *μ*). After each new branch is added to the tree using dnaml's Newton-Raphson method, dnaml *ε* midpoint roots the tree, and then jointly optimizes both branch lengths and gap parameters using conjugate gradient descent. Midpoint rooting is an easy but simplistic rooting method; we made no attempt in dnaml *ε* to optimize the placement of the root, though this would be possible without a significant efficiency cost, using methods described in [74], for example.

For simplicity, in this paper's results, we approximate the average length of ancestral sequences (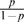) by the average length of sequences in the given alignment. This is in line with common practice in maximum likelihood methods for phylogenetic inference for substitutions, which approximate the prior residue distribution at the root by the observed residue frequencies in the data [24]. In Bayesian methods, the root residue distribution and other parameters of the rate matrix are determined as part of the inference process usually in combination with Markov-chain Monte Carlo (MCMC) methods as for instance in [5]; parameter *p* could be estimated using similar techniques.

Given an ungapped alignment, dnaml *ε* and dnaml are expected to produce identical results (same unrooted tree with same likelihood, same branch lengths, and confidence limits). Given a gapped alignment, comparison of the two implementations allows us to ask how much performance improves when gaps are treated by our extended model as opposed to treating gaps as missing data.

### Benchmarking

We compared dnaml *ε* and dnaml in three types of benchmarking experiments. First, using simulated ungapped alignment data, we confirmed that the two programs give essentially identical results when no insertions and deletions are present. Second, we assessed the ability of the two programs to accurately infer phylogenetic tree topologies for simulated alignment data with insertions and deletions from arbitrarily generated trees. Third, we assessed the programs' ability to correctly infer phylogenetic trees on real ribosomal RNA data.

The problem with benchmarking phylogenetic inference methods is knowing the ground truth. Evolutionary trees are only experimentally known in a few unusual cases for short time scales and rapidly evolving organisms; all other trees have been inferred. The strength of simulated data experiments is that the tree is known, but their weakness is that the simulation must assume an evolutionary model that may be biologically unrealistic and/or too similar to the evolutionary model assumed by the inference method to be tested. On the other hand, the strength of testing on real biological alignments is that the data are realistic, and the weakness is that the true tree is unknown. To evaluate inferences on real data, we have developed a “concordance test” that does not rely on the true tree being known. Testing on both simulated and real data should help compensate for weaknesses of either approach.

#### Tests on simulated ungapped alignments

We first made sure that dnaml *ε* and dnaml produce essentially identical inferences when no insertions or deletions are present. This is a control experiment, making sure that dnaml *ε* correctly infers that optimal deletion and insertion rates are 0 on ungapped alignments, and that the likelihood calculation correctly reduces to the original ungapped version.

We generated simulated 8-taxon rooted trees using the algorithm of Kuhn and Felsenstein [75], which samples a variety of branch lengths and topologies. Each tree was rescaled to a chosen average branch length.

We used the program rose to generate simulated alignments from these sampled phylogenetic trees [76]. We use rose because it allows us to implement reasonably realistic models of insertion and deletion, which we will describe and use in the next section; in these initial ungapped control experiments, we turn off the insertion and deletion parameters. We modified rose to use the F84 rate matrix model for residue substitution, the same model used in dnaml and dnaml *ε*. We used a uniform stationary distribution of 25% for each residue, and a transition/transversion ratio of 2.0 (the dnaml default).

We sampled trees for 9 different average branch lengths, ranging from 0.005 to 2.0 substitutions/site and branch in roughly 2–3× multiplicative steps (corresponding to average pairwise identities ranging from 98% down to a fully saturated 25% in the 8 sequences). (The units of evolutionary time are in principle arbitrary. Substitution rate matrices are traditionally normalized to units of substitutions/site. dnaml *ε* reports time in units of *changes* per site, where changes include substitutions, insertions, and deletions.) For each choice of average branch length, we sample 100 different trees. For each tree, we generate different alignment lengths ranging from 50 to 1000 in steps of 5. A total of 900 trees and 171900 alignments were generated (100 samples each for 9 choices of branch length and 191 choices of alignment length). For each alignment, we infer a maximum likelihood tree topology using dnaml *ε* and dnaml.

To evaluate the correctness of inferred tree topologies, we computed three standard measures: the fraction of correct topologies (true positives, TP), the symmetric difference distance (SDD) [77], and the branch score distance (BSD) [75]. TP simply counts an inferred topology as right or wrong (high TP is better). SDD counts the number of non-identical internal branches in the two (unrooted) trees, where “identical” means splitting the two trees into the same disjoint sets of taxa; for a comparison of trees of 8 taxa, SDD ranges from 0 for identical trees to 10 for maximally dissimilar topologies. BSD evaluates not just the topology but also the correctness of inferred branch lengths, by summing the cost assigned to each internal branch of the square of the difference to the identical branch in the other tree (if there is no identical branch in the other tree, the cost is the whole branch square). Because BSD depends on total tree branch length, in order to compare results across different branch lengths, we calculate a normalized BSD (nBSD) on trees rescaled to an average branch length of one. Both SDD and BSD were calculated using the phylip program treedist. Better inferences are indicated by larger TP and by smaller SDD and nBSD.

**Figure 2:**
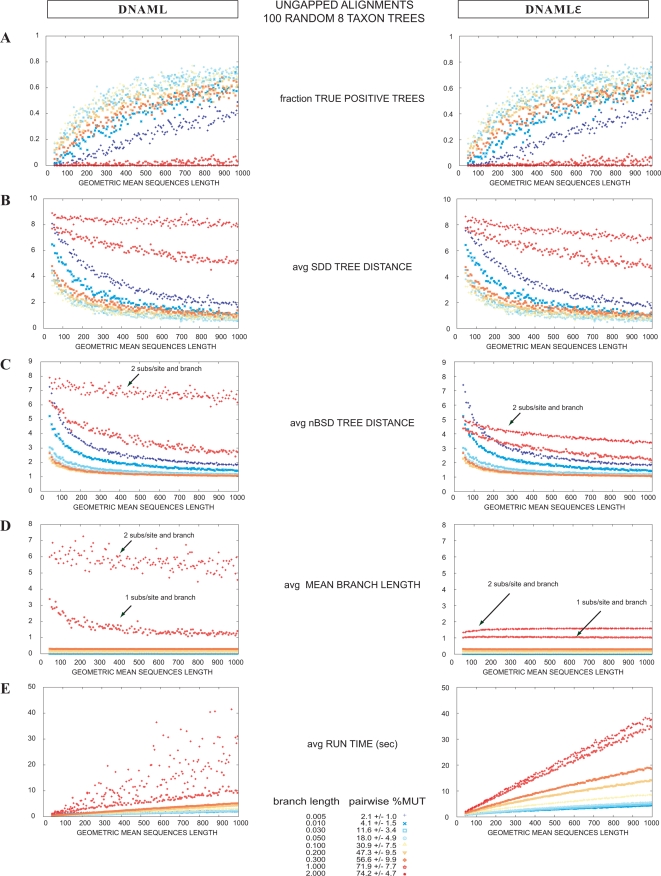
Comparison of dnaml versus dnaml *ε* for ungapped alignments. Tree reconstruction for ungapped alignments generated according to a F84 substitution model (2.0 transition to transversion ratio and equiprobable residues), for nine different time divergences, ranging from 0.005 to 2.0 substitutions per site and branch. For a given divergence value, 100 random trees with eight taxa were used. For each tree, single alignments were generated with lengths ranging from 50 to 1000 residues in 5 residue increments. For each alignment, a tree was inferred using the programs dnaml and dnaml *ε*. Results are displayed as a function of the length of the alignments. (A) Fraction of trees which topology was correctly inferred as a function of the alignment length. The best performance occurs for alignments that contain about 18% pairwise substitutions on average (0.05 substitutions per site and branch). In this case, detectability seems to asymptote to approximately 78% for alignments of at least 800 residues. (B,C) Corresponding results when using the SDD and nBSD measures respectively. (D) Average mean branch length for each length bin. Overall, the two methods show similar performance for ungapped alignment. We mark with an arrow some extreme cases in which the two methods perform differently when inferring the tree branch lengths. (E) Comparison of computational time performance.

**Figure 3:**
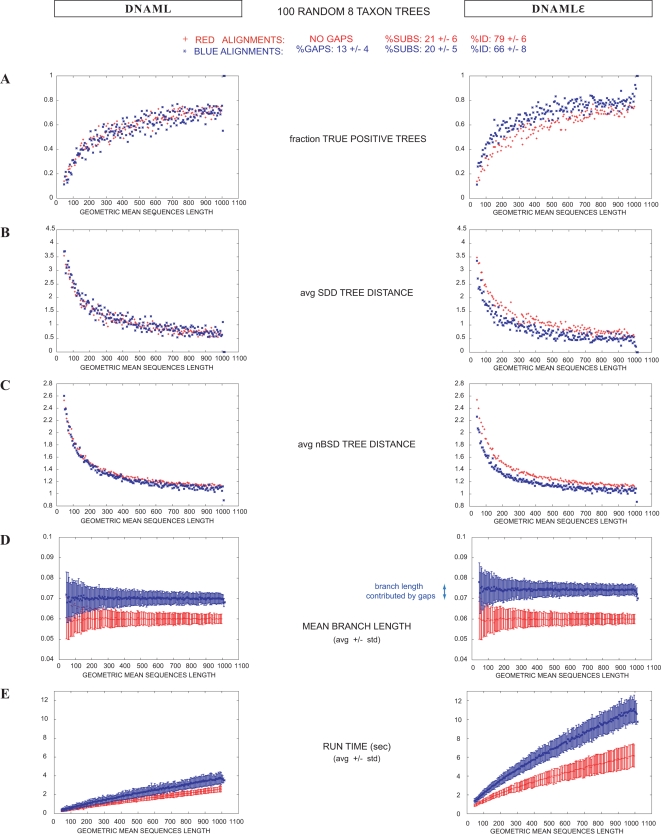
Comparison of dnaml versus dnaml *ε* for synthetic alignments with gaps. Tree reconstruction test for simulated alignments with gaps generated with the program rose [76] according to 100 random eight-taxon trees, using the F84 model for substitutions and a Poisson gap length distribution (
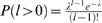
, for *λ* = 0.5). Alignments lengths range from 50 to 1,000 residues. Here we compare the performance of dnaml (left) versus dnaml *ε* (right) for two different evolutionary situations: in red alignments with no gaps and 0.06 substitutions per site and branch, which produce alignments with 21%±6% pairwise average substitutions; in blue alignments with gaps (*p_ins_* = *p_del_* = 0.001 and 0.07 substitutions per site and branch) with a similar percentage of pairwise substitutions (20%±5%) as the ungapped alignments. The alignments with gaps have a percentage of pairwise gaps of 13%±4%. Results are presented as a function of the geometric mean of sequences lengths. The tree reconstruction test assesses the similarity between the inferred tree and the original tree. Three measures of tree similarity are displayed in (A), (B), and (C), respectively: a binary count of whether the trees are topologically identical or not (TP), the Symmetric Difference Distance (SDD) and the normalized Branch Scoring Distance (nBSD). (D) Mean branch length of the inferred trees, and (E) the running time required for the different inferences.

The results are shown in Figure 2. As expected, the accuracy of tree topologies inferred by the two methods is essentially identical by the TP and SDD measures. As in any phylogenetic inference method, accuracy improves with alignment length (more data is better), and shows an optimum average branch length of about 0.05, corresponding to average pairwise sequence identities of about 82% (more similar sequences have fewer substitutions and less signal, and less similar sequences are more saturated). In the best cases (0.05 branch length, alignments longer than 800 nt) both methods infer the correct tree about 78% of the time (Figure 3A). nBSD values are also essentially identical for most choices of average branch length. We do see significant differences in branch length estimation (either by the nBSD test, or by plotting average inferred branch length) at large, saturating choices of average branch lengths of 1.0 or 2.0, corresponding to almost uncorrelated random sequences. In this extreme (and not biologically relevant) regime, branch lengths make little difference in likelihood so long as they are large (and accordingly, likelihoods assigned by the two methods are not significantly different, despite the different inferred branch lengths), and the inferred branch lengths become sensitive to details of the optimization method. Thus these extreme cases appear to be identifying a biologically irrelevant weakness in dnaml's optimizer, which infers overly large branch lengths (5.7±0.5 instead of 2.0 for the largest alignments), whereas dnaml *ε* results, produced by a different implementation of optimizer, are closer to the correct value (1.55±0.05). Overall, with the exception of this minor difference in numerical optimization, these results indicate that dnaml *ε* and dnaml do produce essentially identical results on ungapped alignments, as expected.

#### Tests on simulated alignments with insertions and deletions

Using the same protocol as above, we then asked whether dnaml *ε* would produce better inferences on gapped alignments than dnaml. rose simulates insertions and deletions by inserting and deleting a block of residues at a random position according to a chosen length distribution, and controlled by insertion/deletion probability parameters *p_ins_* and *p_del_* per unit branch length. Note that because rose generates multi-residue insertions and deletions, it simulates a different and more biologically realistic process than the column-independent insertion/deletion process our model assumes. We therefore expect our model to suffer in the simulation from its assumption that gaps are uncorrelated, much as it would in real data; the question is whether this is outweighed by having at least some model of insertion and deletion, compared to dnaml's treatment of gaps as missing data.

#### Tests using a Poisson insertion and deletion length distribution

We had rose sample insertion and deletion lengths *l* from a Poisson distribution 
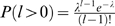
 with *λ* = 0.5. This particular distribution allows with probability 0.3935 insertions or deletions of more than one nucleotide at the time. This is still a weak violation of the column independence assumptions, and in the next section we will test the limits of our model regarding this issue. We used a range of 14 different gap probability parameters *p_ins_* = *p_del_*, ranging from *p_ins_* = *p_del_* = 0 (no gaps) to *p_ins_* = *p_del_* = 0.1.

Results of an illustrative comparison of two choices of gap probability and average branch length are shown in Figure 3. These two datasets have roughly equal frequencies of observed substitution and therefore show roughly equal performance with dnaml (left side of Figure 3): in the first (red points) *p_ins_* = *p_del_* = 0 and average substitution branch length of 0.06 produced ungapped alignment datasets in which all changes were substitutions (pairwise comparisons averaged 21% substitutions and 79% overall identity), and in the second (blue points), setting *p_ins_* = *p_del_* = 0.001 and an average substitution branch length of 0.07 produced alignment datasets with substitution events that averaged 20% in pairwise comparisons, and insertion/deletion events that averaged 13% in pairwise comparisons. These average percentages of substitutions and gaps in the blue dataset are reasonably realistic, comparable to those observed in our real rRNA alignments; see Table 4. The right side of Figure 3 then shows the effect of dnaml *ε* being able to use the information in gaps in addition to substitutions: for dnaml *ε* inferences, the blue dataset (the one with gaps) is more informative, and accuracy increases.

Table 1 shows the relative performance of dnaml *ε* versus dnaml for a large range of alignments with gaps. In order to summarize with one number curves similar to those presented in Figure 3, we calculate the area under the curve (auc) in analogy to ROC curves. In particular, we introduce the relative auc difference, 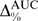. For a given measure *f*, the quantity 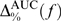 describes (as a percentage) the difference between the auc for dnaml *ε* versus that of dnaml relative to the larger of the two auc's. dnaml *ε* shows consistently better performance than dnaml, and the improvement increases with the percentage of gaps in the alignment.

**Table 1:** Poisson *λ* = 0.5 gap distribution for 8-taxon alignments.

Ave Subs per Site and Branch	rose Gap Parameter	Pairwise % ID	Pairwise % SUBS	Pairwise % GAPS	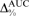 (TP)	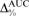 (SDD)	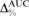 (nBSD)	Mean MBL		Time (L = 1000) (s)	
								dnaml	dnaml *ε*	dnaml	dnaml *ε*
0.005	0.0020	96±2	2±1	2±1	**23.4**	−**18.5**	−**17.6**	0.005	0.007	2.9±1.7	4.7±0.8
		96±2	2±1	2±1	**3.8**	−**5.0**	−**16.3**	0.006	0.008	2.5±1.1	4.6±0.6
0.010	0.0010	94±2	4±1	2±1	**14.8**	−**16.1**	−**7.9**	0.010	0.012	2.4±0.8	5.3±0.7
		94±2	4±2	2±1	−4.8	2.3	−**6.7**	0.010	0.013	2.4±0.9	5.2±0.8
0.010	0.0020	92±3	4±1	4±2	**22.7**	−**26.1**	−**15.7**	0.010	0.014	2.4±0.6	5.9±0.8
		91±3	5±2	4±2	−4.9	4.3	−**14.2**	0.012	0.018	2.4±0.4	5.8±0.8
0.020	0.0010	88±4	8±2	4±2	**12.9**	−**19.1**	−**6.7**	0.020	0.024	2.4±0.2	6.0±0.7
		87±4	9±3	4±2	−8.7	12.3	−**5.3**	0.023	0.027	2.4±0.2	5.9±0.8
0.020	0.0020	85±5	7±2	8±3	**20.5**	−**32.9**	−**12.3**	0.020	0.028	2.8±0.6	7.0±1.0
		83±6	10±4	7±2	−5.2	7.1	−**11.3**	0.030	0.039	2.9±0.6	7.1±0.8
0.030	0.0005	86±4	11±3	3±1	**7.4**	−**14.7**	−**2.7**	0.030	0.032	2.5±0.7	6.0±0.7
		85±4	12±3	3±1	−12.8	15.9	−**1.4**	0.032	0.035	2.7±0.6	6.1±0.7
0.030	0.0010	83±5	11±3	6±2	**14.3**	−**22.4**	−**6.4**	0.030	0.035	2.7±0.5	7.0±1.0
		81±5	13±4	5±2	−10.5	13.3	−**4.8**	0.036	0.042	2.8±0.5	6.9±0.9
0.030	0.0020	78±6	10±3	11±4	**18.4**	−**35.6**	−**12.1**	0.030	0.042	2.9±0.6	8.2±1.2
		75±8	16±5	9±3	−5.5	5.8	−**9.2**	0.050	0.062	3.1±0.4	8.6±1.0
0.040	0.0005	82±5	14±4	4±2	**9.6**	−**17.2**	−**2.7**	0.040	0.042	2.7±0.4	6.5±0.7
		81±5	15±4	4±2	−16.4	19.3	−**0.7**	0.043	0.047	2.7±0.3	6.7±0.6
0.040	0.0010	78±6	14±4	8±3	**15.3**	−**26.1**	−**6.2**	0.040	0.045	3.0±0.5	8.0±0.9
		76±7	17±5	7±2	−10.1	12.3	−**3.7**	0.051	0.058	3.2±0.8	7.9±0.7
0.040	0.0015	75±7	13±4	12±4	**16.9**	−**33.1**	−**8.4**	0.040	0.048	3.1±0.4	8.9±1.2
		72±8	19±6	9±3	−7.1	7.8	−**6.2**	0.061	0.071	3.4±0.6	9.1±1.2
0.040	0.0020	73±7	12±3	15±4	**20.7**	−**37.2**	−**10.6**	0.040	0.052	3.4±0.5	9.4±1.3
		68±9	21±7	11±3	−4.7	4.6	−**8.4**	0.072	0.085	3.7±0.4	10.8±1.3
0.070	0.0005	71±7	22±5	7±3	**12.6**	−**21.3**	−**3.0**	0.070	0.071	3.3±0.4	9.6±1.0
		70±8	24±6	6±2	−11.6	15.0	−**0.3**	0.080	0.085	3.4±0.5	9.3±0.9
0.070	0.0010	66±8	20±5	13±4	**16.0**	−**31.2**	−**6.3**	0.070	0.074	3.7±0.4	10.8±1.1
		62±10	28±8	10±3	−9.2	9.3	−**3.2**	0.101	0.109	3.9±0.3	11.6±1.2
0.070	0.0015	62±9	19±5	19±5	**20.2**	−**39.6**	−**8.2**	0.070	0.078	4.0±0.6	12.5±1.7
		57±11	31±8	12±3	−7.1	5.0	−**9.1**	0.126	0.135	4.7±0.6	14.6±1.8
0.070	0.0020	58±10	18±4	24±7	**23.7**	−**41.5**	−**10.8**	0.070	0.083	4.3±0.7	13.5±1.7
		52±12	35±9	13±4	−4.4	3.1	−**11.9**	0.154	0.162	5.1±0.7	16.8±2.4
0.100	0.0005	63±9	28±6	10±3	**17.1**	−**29.7**	−**3.6**	0.100	0.100	4.0±0.7	12.1±1.3
		60±10	32±8	8±3	−11.7	12.7	−**0.4**	0.121	0.125	4.1±0.5	12.0±1.5
0.100	0.0010	57±9	25±6	18±6	**20.6**	−**40.3**	−**6.3**	0.100	0.100	4.5±0.7	14.5±1.3
		52±12	36±9	11±3	−7.9	6.6	−**6.3**	0.160	0.165	4.7±0.5	15.4±2.3
0.100	0.0015	52±10	23±5	25±6	**25.3**	−**44.1**	−**8.8**	0.100	0.105	4.9±0.6	16.6±2.2
		47±12	40±10	13±4	−4.0	2.0	−**12.2**	0.201	0.202	5.7±1.0	19.0±3.3
0.100	0.0020	48±11	21±4	31±7	**27.2**	−**50.7**	−**11.4**	0.100	0.110	5.4±0.7	18.3±2.2
		43±12	43±10	14±4	**0.3**	−**0.5**	−**16.9**	0.242	0.234	6.0±1.0	20.7±3.1
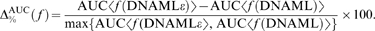											
For a given method M, the area under the curve (auc): 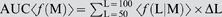 ,											
where:											
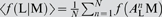 , for alignments 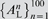 with geometric mean of sequence length l, and Δl = 5 nts, for these experiments.											
**Nomenclature:**											
TP = fraction of true positive trees.											
SDD = Symmetric Difference Distance.											
NBSD = normalized Branch Score Distance.											
MBL = mean branch length.											

For a large variety of synthetic alignments of 8 taxa with gaps generated using a Poisson (*λ* = 0.5) length distribution. We compare the performance of dnaml *ε* respect to dnaml using three different measures. For each measure, we report the relative area under the curve (auc) difference 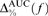, which for a given measure *f* describes (as a percentage) the difference between the auc for dnaml *ε* versus that of dnaml relative to the larger of the two auc's. In white, we show the results obtained with the phylogenetically correct alignments, in gray after realigning with clustalw 1.83. In bold when the result is favorable to dnaml *ε*.

Figure 3 and Table 1 also show the mean branch lengths inferred by the two methods. For the gapped alignments (blue), dnaml *ε* infers longer branch lengths than dnaml (in units of changes/site) because it counts insertions and deletions as changes, in addition to substitutions.

Table 1 also shows results (in gray) when instead of using the phylogenetically correct alignments, we allow the sequences to be realigned using a standard (and not phylogenetically aware) alignment algorithm. In particular for the experiments in this paper, we used clustalw version 1.83. The more noticeable effect of realigning is that the columns of the alignment get compressed. The number of substitutions increases and the number of gaps decreases respect to the phylogenetically correct alignments. As expected, performance of dnaml *ε* declines in terms of predicting the phylogenetic topology correctly, (although the two methods become almost equivalent in terms of topological measures as the number of gaps in the realignments increases). However, as seen in Table 1, dnaml *ε* still shows improvement in all cases in terms of the nBSD measure which takes into account the combined effect of both the accuracy of the topology and the accuracy of the branch lengths. These results show that the loss due to the lack of a phylogenetically correct alignment seems to be compensated by the contribution to branch lengths introduced by treating insertions and deletions as real evolutionary events.

The time performance of dnaml *ε* is similar to that of dnaml. Both scale linearly with alignment length (and with number of taxa, though this is not shown by these experiments, which use a fixed number of 8 taxa). For both methods, the scaling factor increases as the number of substitutions and the number of gaps increases (see Table 1). dnaml *ε* shows (for trees of eight taxa and alignments of 1,000 residues) about a three-fold increase in average run time compared to dnaml, whether the alignment has gaps or not.

#### Tests sampling from the generative model

As a matter of completeness, we have also sampled from the generative model described in this work using the program *ε* rate. The model inserts residues following a geometric distribution controlled by the time-dependent parameter *ξ_t_* in Equation 7. We applied the protocol described in the previous section. Results for gapped alignments with a large range of degrees divergence (comparable to those presented in the previous section) are given in Table 2. As expected, this is a relatively easier test than that posed by alignments with Poisson distributed gaps, but in general, similar trends are observed in the results (compare Table 1 with Table 2, see also Table 3).

**Table 2:** Geometric gap distribution for 8-taxon alignments.

Ave Subs per Site and Branch	*λ* = *μ* Parameter	Pairwise % ID	Pairwise % SUBS	Pairwise % GAPS	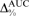 (TP)	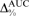 (SDD)	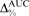 (nBSD)	Mean MBL		Time (L = 1000) (s)	
								dnaml	dnaml *ε*	dnaml	dnaml *ε*
0.005	0.30	96±2	2±1	2±1	**28.9**	−**27.5**	−**24.9**	0.005	0.007	2.8±1.5	4.4±0.6
		95±2	3±1	2±1	**7.6**	−**7.5**	−**22.3**	0.006	0.009	2.2±0.5	4.5±0.6
0.010	0.15	94±2	4±2	2±1	**19.8**	−**21.4**	−**13.1**	0.010	0.012	2.2±0.7	5.0±0.8
		93±2	5±2	2±1	−3.2	2.9	−**11.7**	0.012	0.014	2.2±0.2	5.0±0.7
0.010	0.30	92±3	4±1	4±2	**28.7**	−**36.1**	−**20.9**	0.010	0.014	2.8±1.3	5.7±0.8
		91±3	6±2	3±1	**0.3**	0.2	−**17.1**	0.015	0.021	2.4±0.3	6.0±0.9
0.020	0.15	88±4	8±3	4±2	**16.0**	−**27.8**	−**11.7**	0.020	0.024	2.5±0.4	5.9±0.9
		87±4	9±3	4±1	−7.0	7.6	−**8.2**	0.025	0.030	2.5±0.3	6.1±0.9
0.020	0.30	85±4	7±2	8±3	22.3	−40.6	−18.0	0.020	0.028	2.7±0.4	6.7±0.9
		86±4	12±4	2±1	0.0	0.0	−**11.6**	0.036	0.045	2.4±0.3	5.7±0.7
0.030	0.06	86±4	11±3	2±1	**12.0**	−**18.9**	−**5.4**	0.030	0.032	2.5±0.4	5.9±0.7
		86±4	12±4	2±1	−9.3	11.3	−**3.5**	0.032	0.053	2.4±0.3	5.7±0.7
0.030	0.15	83±5	11±3	6±2	**17.7**	−**33.5**	−**10.4**	0.030	0.035	2.6±0.5	6.7±0.9
		81±6	14±5	5±2	−5.0	6.7	−**6.7**	0.041	0.048	2.9±0.4	6.9±0.8
0.030	0.30	78±6	10±3	12±3	**22.1**	−**45.9**	−**16.4**	0.030	0.040	3.0±0.4	8.2±0.9
		73±8	19±6	8±2	**1.9**	−**1.6**	−**9.5**	0.060	0.071	3.3±0.3	9.0±1.1
0.040	0.06	82±5	14±4	3±1	**11.0**	−**22.6**	−**5.0**	0.040	0.042	2.7±0.3	6.5±0.6
		81±5	16±5	3±1	−10.4	14.6	−**2.6**	0.044	0.047	2.7±0.4	6.5±0.8
0.040	0.15	78±6	14±4	8±3	**18.3**	−**35.7**	−**9.4**	0.040	0.045	2.9±0.4	7.7±0.8
		75±7	19±6	6±2	−4.1	4.4	−**4.8**	0.058	0.066	3.1±0.4	8.2±1.0
0.040	0.24	75±7	13±4	12±4	**20.9**	−**42.3**	−**13.1**	0.040	0.049	3.2±0.5	8.8±1.1
		70±8	22±7	8±2	−0.6	0.6	−**7.4**	0.074	0.085	3.6±0.4	9.6±1.2
0.040	0.30	73±7	12±3	15±4	**23.4**	−**46.5**	−**13.9**	0.040	0.052	3.3±0.5	9.3±1.6
		66±9	25±7	9±3	**2.5**	−**1.4**	−**8.4**	0.085	0.097	4.0±0.5	11.0±1.6
0.070	0.06	72±7	22±5	7±3	**14.3**	−**30.2**	−**4.6**	0.070	0.071	3.3±0.6	8.9±0.9
		70±8	25±7	5±2	−7.5	10.4	−**1.4**	0.082	0.086	3.4±0.4	9.1±1.1
0.070	0.15	67±7	20±5	13±4	**23.5**	−**42.8**	−**9.1**	0.070	0.074	3.8±0.7	11.3±1.3
		61±10	31±8	8±3	−3.2	2.9	−**5.4**	0.113	0.121	4.1±0.6	12.2±2.0
0.070	0.24	61±9	19±4	20±5	**25.3**	−**47.5**	−**11.5**	0.070	0.079	4.2±0.7	12.8±1.6
		55±11	35±9	10±3	−4.4	1.8	−**7.7**	0.147	0.154	4.8±0.8	15.0±2.7
0.070	0.30	58±9	18±4	24±6	**24.1**	−**47.3**	−**13.2**	0.070	0.083	4.4±0.5	13.9±1.6
		51±11	38±9	11±3	−3.0	1.8	−**10.1**	0.170	0.176	5.0±0.6	16.5±2.8
0.100	0.06	64±8	28±6	8±2	**16.8**	−**34.6**	−**5.5**	0.100	0.097	3.8±0.6	11.2±1.5
		61±10	33±8	6±2	−11.6	10.1	−**0.7**	0.123	0.127	3.9±0.4	11.7±1.7
0.100	0.15	57±10	25±6	18±5	**22.4**	−**44.2**	−**9.8**	0.100	0.100	4.5±0.6	14.8±4.7
		51±11	39±9	10±3	−6.1	3.4	−**7.3**	0.175	0.179	5.0±0.5	16.1±3.2
0.100	0.24	51±10	22±5	26±6	**29.6**	−**50.8**	−**11.5**	0.101	0.105	5.3±0.8	17.0±2.1
		46±12	43±10	11±3	**4.7**	−**2.9**	−**12.4**	0.223	0.220	5.6±0.7	19.1±4.1
0.100	0.30	47±11	21±4	32±7	**29.1**	−**53.5**	−**13.7**	0.102	0.110	5.4±0.8	18.2±2.4
		43±12	46±10	12±3	**4.1**	−**3.8**	−**15.3**	0.254	0.243	5.8±0.8	21.0±3.8
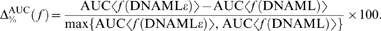											
For a given method M, the area under the curve (auc): 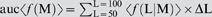											
where:											
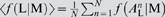 , for alignments 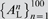 with geometric mean of sequence length l, and Δl = 5 nts, for these experiments.											
**Nomenclature:**											
TP = fraction of true positive trees.											
SDD = Symmetric Difference Distance.											
NBSD = normalized Branch Score Distance.											
MBL = mean branch length.											

Similarly to Table 1, we show results for a large variety of synthetic alignments of 8 taxa with gaps generated using the generative model described in this paper. We compare the performance of dnaml *ε* respect to dnaml using three different measures. As in Table 1 and for each measure, we report the relative area under the curve (auc) difference 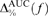 defined above. White rows indicate results for the phylogenetically correct alignments. Gray rows indicate results after realigning the evolved sequences with clustalw 1.83. In bold when the result is favorable to dnaml *ε*.

**Table 3:** 8-Taxon alignments.

Gap Length Distribution	Ave Subs per Site and Branch	Gap Parameter	Pairwise % id	Pairwise % subs	Pairwise % gaps	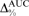 (TP)	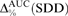	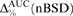
*ε* rate	0.005	0.30	96±2	2±1	2±1	**28.9**	−**27.5**	−**24.9**
poisson *λ* = 0.5	0.005	0.0020	96±2	2±1	2±1	**24.6**	−**19.8**	−**16.8**
coding *c*/*t* = 100	0.005	0.0020	96±2	2±1	2±2	**4.8**	−**2.5**	11.8
coding *c*/*t* = 6	0.005	0.0010	96±4	2±1	2±4	−4.5	5.0	36.8
coding *c*/*t* = 1	0.005	0.0005	96±4	2±1	2±4	−8.1	7.0	41.3
*ε* rate	0.020	0.30	85±4	7±2	8±3	**22.3**	−**40.6**	−**18.0**
poisson *λ* = 0.5	0.020	0.0020	85±5	7±2	8±3	**20.5**	−**32.9**	−**12.3**
coding *c*/*t* = 100	0.020	0.0020	85±5	7±2	7±4	**2.9**	−**0.5**	11.3
coding *c*/*t* = 6	0.020	0.0010	84±8	7±2	9±7	−11.6	19.9	43.3
coding *c*/*t* = 1	0.020	0.0005	85±8	7±2	7±8	−15.1	20.9	51.6
*ε* rate	0.040	0.30	73±7	12±3	15±4	**23.4**	−**46.5**	−**13.9**
poisson *λ* = 0.5	0.040	0.0020	73±7	12±3	15±4	**20.7**	−**37.2**	−**10.6**
coding *c*/*t* = 100	0.040	0.0020	73±8	13±4	14±5	**0.7**	0.2	8.3
coding *c*/*t* = 6	0.040	0.0010	72±10	12±4	16±10	−21.9	31.3	40.4
coding *c*/*t* = 1	0.040	0.0005	73±11	13±4	14±11	−26.9	36.4	50.1
*ε* rate	0.070	0.30	58±9	18±4	24±6	**24.1**	−**47.3**	−**13.2**
poisson *λ* = 0.5	0.070	0.0020	58±10	18±4	24±7	**23.7**	−**41.4**	−**10.8**
coding *c*/*t* = 100	0.070	0.0020	60±10	18±4	22±7	**2.4**	−**2.4**	5.5
coding *c*/*t* = 6	0.070	0.0010	57±12	17±5	26±12	−28.6	34.5	38.0
coding *c*/*t* = 1	0.070	0.0005	59±13	18±5	23±13	−39.4	50.1	48.9
**Nomenclature:**								
auc = area under the curve								
TP = fraction of true positive trees.								
SDD = Symmetric Difference Distance.								
NBSD = normalized Branch Score Distance.								

We assess the performance of dnaml *ε* for five different gap length distributions using alignments of 8 taxa with similar average pairwise substitutions and gaps. The gap distributions used to create the synthetic alignments are ordered by increasing degree of violation of the column independence assumption. For a given measure, we report the relative auc difference 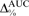 introduced in Table 1. In bold when the result is favorable to dnaml *ε*.

#### Test of the column independence assumption

In order to test the limitations of considering individual gaps as independent events, we also had rose sample insertions and deletions from a distribution that simulates a DNA alignment of a protein-coding region. To that purpose, we used a nucleotide version of the empirically-derived coding gap distribution introduced in simprot [78] that allows gaps to occur only as multiple of three. We also added an option in rose in order to allow insertions and deletions to occur randomly but only at multiple of three positions with respect to the start of the alignment.

The simprot gap length distribution depends on one parameter *c*/*t*. We selected three different values, in increasing order of divergence: *c*/*t* = 100 for which *p*(3 nts) = 0.7238 and *p*(24 nts) = 0.0001; a second distribution *c*/*t* = 6 which is a good approximation to a empirically-determined distribution trained on protein sequences with less than 100 PAM sequence divergence [79]; and finally *c*/*t* = 1 which corresponds exactly to a distribution empirically determined from proteins sharing no more than 25% sequence identity [80]. We limited the maximum number of inserted or deleted nucleotides to 100. For the *c*/*t* = 6 distribution, one has *p*(3 nts) = 0.4510 and *p*(99 nts) = 0.0009. For the *c*/*t* = 1 distribution, one has *p*(3 nts) = 0.2938 and *p*(99 nts) = 0.0034. For each of the three protein-coding gap distributions, we applied the same protocol as for the Poisson distribution used previously.

As we observe in Table 3, the *c*/*t* = 100 coding gap length distribution dnaml *ε* still shows an improvement respect to dnaml at least for the two topological measures, this is despite the fact that this distribution breaks the column independence assumption more strongly than the Poisson distribution used in the previous section (*p*(*l*≥6 nts) is 0.2762 for this distribution, compared to 0.0002 for the Poisson distribution). However, for the two more divergent coding gap length distributions, the size of the insertion/deletion blocks is so large that by taking gaps into account, we cannot even reproduce the topology of the original substitutions-only tree.

#### Test for long-branch attraction

To make sure that dnaml *ε* does not suffer for some unexpected reason from significantly more systematic long-branch attraction than dnaml itself, we used 4-taxon trees depending on two parameters [81]. One parameter is the branch length of the internal branch and two opposite leaves (the three-branch parameter, *t*_3_), the other parameter is the branch length of the other two opposite leaves (the two-branch parameter, *t*_2_). Using the same protocol as before, we assessed the programs performance on alignments generated according to 4-taxon trees in which the two-branch parameter is 10 times longer than the three-branch parameter, for all gap length distributions used above.

Results are presented in Table 4. For the phylogenetically correct alignments, we do not observe any long branch attraction effect. In fact, in most cases (except for the two most extreme coding distributions and the most divergent cases) dnaml *ε* tends to perform better than dnaml, and the improvement is larger for the case prone to long branch attraction (*t*_3_ = 10*t*_2_ or *r* = 10) than for the balanced case in which all five branches are equal (*t*_3_ = *t*_2_ or *r* = 1). For the realigned tests, both dnaml and dnaml *ε* show a certain long-branch attraction tendency when the amount of substitutions nearly triples that of gaps. For the more extreme coding distribution, the effect seems to be more severe for dnaml *ε*. This effect can be attributed to the column independence assumption. These results show that while there is some systematic long-branch attraction for dnaml *ε* and dnaml that effect occurs in some extreme situations which we do not see reproduced in real alignment such as those of rRNA.

**Table 4:** 4-Taxon alignments.

Gap Length Distribution	Ave Subs per Site and Branch	Gap Parameter	Pairwise % id	Pairwise % subs	Pairwise % gaps	〈TP〉_%_ *r* = 1	〈TP〉_%_ *r* = 1	〈TP〉_%_ *r* = 10	〈TP〉_%_ *r* = 10
						dnaml	dnaml *ε*	dnaml	dnaml *ε*
*ε* rate	0.005	0.30	97±2	1±1	2±1	94.3	96.2	67.1	80.6
			97±2	2±1	1±1	93.6	95.6	64.0	69.4
poisson *λ* = 0.5	0.005	0.0020	97±2	2±1	2±1	93.5	95.1	65.2	76.0
			97±2	2±1	2±1	93.5	94.5	63.8	69.7
coding *c*/*t* = 100	0.005	0.0020	97±2	1±1	1±1	91.9	92.6	63.3	72.1
			97±2	2±1	1±1	92.2	92.4	63.1	69.8
coding *c*/*t* = 6	0.005	0.0010	97±2	1±1	2±3	91.4	91.6	62.9	96.6
			97±3	2±1	2±3	91.1	91.0	61.5	68.5
coding *c*/*t* = 1	0.005	0.0005	97±4	1±1	1±3	90.7	91.4	62.7	69.0
			97±4	2±1	1±4	91.3	90.4	62.3	69.0
*ε* rate	0.010	0.30	94±3	3±1	3±2	97.4	98.3	78.2	89.6
			94±3	4±2	2±1	97.1	97.1	71.3	70.0
poisson *λ* = 0.5	0.010	0.0020	94±3	3±1	3±1	97.6	98.6	77.4	86.4
			94±3	3±1	3±1	97.2	98.1	74.0	72.5
coding *c*/*t* = 100	0.010	0.0020	94±3	3±1	3±2	96.9	97.3	76.0	81.1
			94±3	3±1	3±2	96.8	96.8	75.4	74.4
coding *c*/*t* = 6	0.010	0.0010	94±5	3±1	3±4	96.5	97.0	75.6	78.4
			94±5	3±2	3±4	96.6	96.2	75.3	75.8
coding *c*/*t* = 1	0.010	0.0005	94±5	3±1	3±5	96.8	96.8	74.5	77.6
			94±6	3±2	3±5	96.4	96.4	75.1	77.3
*ε* rate	0.030	0.30	83±7	8±3	9±4	99.5	99.9	89.9	96.7
			80±7	14±6	6±2	99.3	99.4	65.8	**43.6**
poisson *λ* = 0.5	0.030	0.0020	84±7	8±3	8±4	99.4	99.8	89.8	95.4
			83±7	11±5	7±3	99.2	99.4	76.3	55.5
coding *c*/*t* = 100	0.030	0.0020	84±7	8±3	8±4	99.5	99.5	90.0	90.4
			83±7	9±4	7±4	99.3	98.9	85.1	66.3
coding *c*/*t* = 6	0.030	0.0010	83±9	8±3	10±8	99.2	98.6	88.4	85.8
			83±9	9±4	9±7	99.0	96.8	85.4	67.9
coding *c*/*t* = 1	0.030	0.0005	83±10	8±3	8±9	98.2	97.2	89.2	85.3
			83±10	9±4	8±8	98.0	95.4	86.2	74.5
*ε* rate	0.050	0.30	75±10	12±4	14±6	99.6	100.0	92.86	98.0
			72±10	23±8	8±2	99.3	99.5	**46.8**	**25.7**
poisson *λ* = 0.5	0.050	0.0020	75±10	11±4	13±6	99.8	99.9	92.5	97.1
			73±10	19±5	10±2	99.2	99.4	62.6	**36.1**
coding *c*/*t* = 100	0.050	0.0020	76±10	11±4	12±6	99.5	99.6	92.2	92.0
			74±9	15±6	9±4	99.4	98.8	82.7	52.3
coding *c*/*t* = 6	0.050	0.0010	74±12	12±4	15±10	99.3	98.7	90.8	84.7
			72±12	14±6	13±8	98.4	95.6	82.9	55.8
coding *c*/*t* = 1	0.050	0.0005	74±12	12±4	13±11	99.0	97.6	91.4	83.6
			74±13	14±6	12±10	98.2	94.7	86.0	63.6
*ε* rate	0.100	0.30	58±14	19±5	24±9	99.8	100.0	92.9	98.8
			53±11	38±9	10±3	98.1	98.3	**34.9**	**32.4**
poisson *λ* = 0.5	0.100	0.0020	55±14	19±5	24±9	99.6	100.0	93.7	98.0
			54±11	35±10	11±4	98.3	98.5	**35.1**	**33.8**
coding *c*/*t* = 100	0.100	0.0020	59±14	17±5	22±9	99.8	99.7	94.0	91.5
			53±11	29±9	13±5	99.0	98.0	51.4	**43.8**
coding *c*/*t* = 6	0.100	0.0010	54±16	18±6	28±14	98.4	98.4	92.1	78.6
			54±15	26±9	20±10	97.5	92.4	51.3	**38.9**
coding *c*/*t* = 1	0.100	0.0005	56±16	19±6	24±15	99.0	97.4	92.5	77.0
			56±15	25±9	19±11	97.9	91.4	63.8	**44.1**
**nomenclature:**									
〈TP〉_%_ = mean percentage of true positive trees.									

We test the possibility of spurious long branch attraction associated to dnaml *ε* using 4-taxon trees depending on two parameters: *t*_3_ the length of the internal branch and two opposite leaves, and *t*_2_, the length of the other two opposite leaves [81]. We compare the performance of dnaml *ε* respect to that of dnaml using alignments generated by trees with similar average substitutions branch length (abl) but such that in one case the four leaves are identical (*t*_3_ = *t*_2_ or *r* = 1) with another extreme case prone to long branch attraction in which two opposite leaves are 10 times longer than the other two leaves and the internal branch (*t*_2_ = 10-*t*_3_ or *r* = 10). For a given “abl” and a given parameter *r*, one has 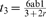 and *t*_2_ = *rt*_3_. For each method (dnaml and dnaml *ε*) and tree configuration (*r* = 1 and *r* = 10), we report the mean percentage of true positives (TP). No long branch attraction is observed for the phylogenetically correct alignment (in white). For the clustalw 1.83 alignments (in gray), both methods have a tendency to infer the wrong tree as the divergence increases. In bold when more than 50% of the trees are incorrectly predicted.

#### Concordance test on ribosomal RNA alignments

We developed a test to evaluate a phylogenetic inference method on real data, called the “concordance test”. We split an alignment randomly into two disjoint sets of columns, infer a tree on each, and ask if the two trees are identical. The frequency that the same tree is obtained for different subsets of columns from the same alignment should be correlated with phylogenetic inference accuracy, because in general, we only expect to obtain the same tree if it is the correct tree. The concordance test should be well suited for evaluating whether a modified method extracts more information from a given alignment, which is the question at hand here. The test may be less suited for evaluating the absolute accuracy of an entirely new inference program, because some systematic errors, such as long branch attraction, could result in agreement on the wrong tree; simulations could be used to detect such systematic problems, though.

We found it is important to choose columns randomly, rather than simply splitting an alignment in half, because real alignments often contain preferentially 5′- or 3′-truncated sequence fragments.

In order to evaluate the performance of dnaml *ε* and dnaml on real alignments, we applied the concordance test to a large number of ribosomal RNA alignments. We obtained curated rRNA alignments from the Comparative RNA Web Site (CRW; http://www.rna.ccbb.utexas.edu) [82] for different domains of life – five small subunit (SSU) alignments and four large subunit (LSU) alignments – and filtered out sequences more than 95% identical to another. These datasets are summarized in Table 5. We randomly sampled a large number of eight-taxon subalignments from these datasets and applied the concordance test with the dnaml *ε* and dnaml methods.

**Table 5:** rRNA alignment statistics.

SSU	Archaea	Chloroplasts	Bacteria	Eukarya	Mitochondria
No. seqs	74	75	1601	900	617
Alignment length	1756	2456	3094	7160	4716
Geometric mean seqs	1446	1446	1497	1788	1011
Pairwise % ID	71±7	74±11	68±6	56±14	49±20
Pairwise % SUBS	23±5	19±7	22±4	20±5	25±6
Pairwise % GAP	6±4	7±5	11±5	24±12	26±20
Total % gaps	17.6	39.0	53.2	74.8	78.0

LSU	Archaea	Chloroplasts	Bacteria	Eukarya
No. seqs	26	32	120	89
Alignment length	3346	4311	4555	9055
Geometric mean seqs	2994	2936	2918	3623
Pairwise % ID	65±9	70±10	67±6	50±12
Pairwise % SUBS	29±7	20±5	25±4	22±5
Pairwise % GAP	6±2	11±5	9±4	28±11
Total % gaps	10.5	31.8	35.9	59.6

Statistics of the rRNA alignments obtained from the Comparative RNA Web Site [82] after sequences with more than 95% identity to each other have been removed from the alignments.

**Figure 4:**
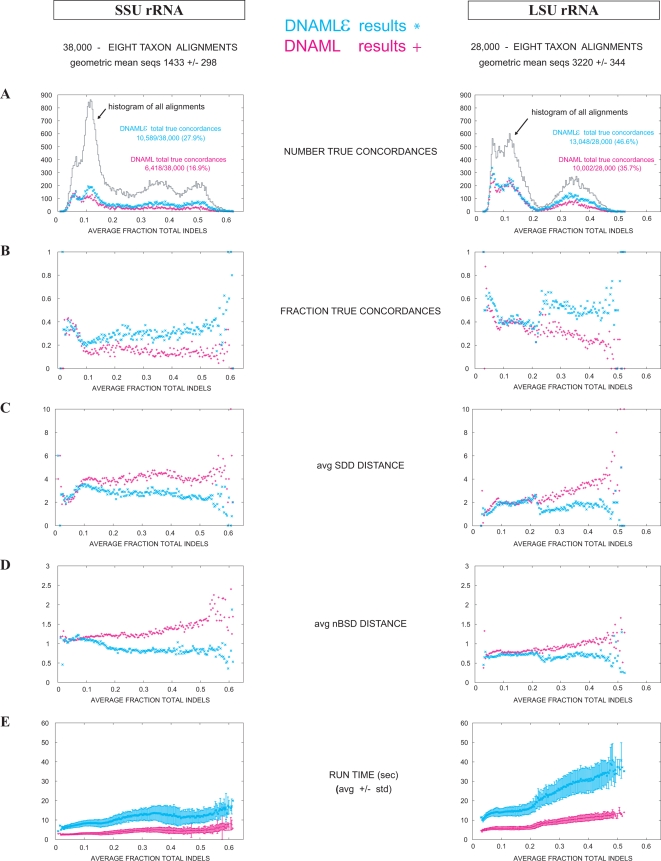
Comparison of dnaml versus dnaml *ε* using the “tree concordance test” on ribosomal RNA alignments. Tree concordance test for SSU (left) and LSU (right) rRNA alignments displayed as a function of the total fraction of gaps present in the alignment. We used five SSU and four LSU alignments described in Table 5. For each alignment, we randomly selected a large number of eight taxa alignments (4,000 for the Archaea and Chloroplasts alignments, and 10,000 for the Eukarya, Bacteria and Mitochondria alignments). Each eight taxa alignment was first shuffled and then split in two halves. The tree concordance test assesses the similarity between the two trees inferred for the two sections of the alignment. Three measures of tree similarity are displayed: a binary count of whether the trees are topologically identical or not (TP), the Symmetric Difference Distance (SDD) and the normalized Branch Scoring Distance (nBSD). Results for all SSU (LSU) tests have been summarized together. (A) A histogram of total alignments, as well as the number of TPs for dnaml (magenta) and dnaml *ε* (cyan) as a function of the total fraction of gaps in the alignment. (B,C,D) Results for the fraction of TPs, the SDD, and the nBSD respectively. Overall tree concordance for SSU rRNA is 27.9% (10,589/38,000) for dnaml *ε*, versus for 16.9% (6,418/38,000) dnaml. Overall tree concordance for LSU rRNA is 46.6% (13,048/28,000) for dnaml *ε* , versus 35.7% (10,002/28,000) for dnaml. (E) shows a comparison of time performance. dnaml *ε* shows on average a two to three fold time increase respect to dnaml for eight taxa alignments.

Results are summarized in Figure 4. Overall, dnaml *ε* shows tree concordance of 27.9% for SSU and 46.6% for LSU, while dnaml shows tree concordance in 16.9% for SSU and 35.7% for LSU. The error estimate for all these results is about 0.5–0.6%, which indicates that the improvement obtained by dnaml *ε* is significant. LSU alignments are longer than SSU (4205±1179 versus 1959±579), probably explaining the better performance. For alignments with few gaps, the two methods produce similar results. The improvement of dnaml *ε* over dnaml increases with the frequency of gaps in the alignments.

With respect to computational time, Figure 4D shows that both methods scale similarly, with dnaml *ε* taking about two- to three-fold longer.

## Discussion

We have presented a non-reversible probabilistic model of sequence evolution accounting for substitutions, insertions, and deletions that is based on a continuous-time Markov process. This model does not assume a pre-stated number of columns. Rather, it describes a generative evolutionary model of substitution, insertion, and deletion events, starting from an explicit prior distribution over ancestral sequences of any length. This avoids the conceptual flaws that easily arise in column-independent models based on a Markov process that includes the gap character. The model remains compatible with efficient post-order transversal algorithms to calculate the likelihood of a phylogenetic tree. The model can also be used to calculate the probability of ancestral sequences with an arbitrary number of residues that leave no trace in the observed alignment.

To do this, the model assumes column independence, an assumption that is problematic because insertions and deletions typically involve multiple residues at the same time. Although we can indeed produce synthetic examples for which our model breaks down due to the column independence assumption, our results for real rRNA alignments show that the gap-extended model is able to produce better trees than the standard model, indicating that the cost of the assumption is outweighed by the gain in modeling gaps instead of ignoring them as missing data. McGuire *et al.* have already made the same observation [41]. There are ways in which we might relax the column independence assumption; for example, one could extend the ideas of context-dependent substitution rate matrices and context-dependent residue distributions [83].

The model introduces as free parameters the rates of insertions and deletions, and the geometric probability parameter for the distribution of ancestral sequences. In the results presented here, for simplicity, we have used the F84 substitution rate matrix, identical deletion rates for all residues, and assumed that inserted residues have the same probability distribution as the stationary distribution of the substitution process. Our model does not require these simplifications. Our results may be generalized to arbitrary substitution rate matrices, deletion rates, and inserted residue probability distributions.

It would be straightforward to integrate this method into any probabilistic model of sequence alignment, including phylo-HMMs [38],[84] and phylo-SCFGs [21], in order to account for insertion and deletion events as well as substitution events, without changing the algorithmic complexity of the existing algorithms, and where the insertion and deletion parameters can be inferred from the data [46].

### Comparison to Reversible Models

The tkf91 model and McGuire's model are designed to be reversible. Our model is non-reversible with respect to insertions and deletions. Reversibility is a mathematically convenient assumption in phylogenetic inference (allowing inference on unrooted trees, for example), and for residue substitution events, usually seem reasonable. However, in the case of insertion/deletion processes, reversibility seem less easy to justify, if one expects the insertion rate *λ* and the deletion rate *μ* to behave as independent parameters.

For a model to be reversible with respect to insertions/deletions, obviously constraints must be imposed on the insertion rate *λ* and deletion rate *μ*; moreover, the constraints imposed by reversibility can be counterintuitive. Consider what happens in a case of zero deletion rate and a positive insertion rate. For McGuire's model, reversibility requires that the frequency of gaps is a constant (*π*_–_), and that it is related to the rates of insertions and deletions by the condition *π*_–_ = *μ*/(*λ*+*μ*). For the tkf91 model, reversibility requires a length distribution for evolved sequences identical to that of ancestral sequences which is geometric with parameter *λ*/*μ* [85]. So, in McGuire's model, imposing *μ* = 0 automatically implies that the gap frequency is zero, effectively converting the model into a substitution-only model, regardless of the insertion rate. In tkf91, the length distribution breaks down entirely in the *μ*<*λ* regime, and gives arbitrarily large joint “probabilities”. Our model remains valid for any arbitrary (positive or zero) values for insertion and deletion rate.

Another consequence of imposing reversibility is that the expected frequencies of insertions and deletions in a pairwise alignment must be identical. For McGuire's model, the expected frequencies of insertions and deletions in a pairwise alignment are given by,
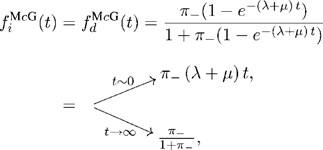
(30)


where *π*_–_ is the constant frequency of a gap, and *λ*+*μ* = *β*(1−*π*_–_), where *β* is one of the two parameters of the F84 substitution model [14]. For the tkf91 model, the expected frequencies of insertions and deletions in a pairwise alignment are given by (see Text S4):
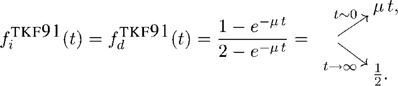
(31)


Therefore in tkf91 for small divergence times, the expected frequency of *insertions* is solely dependent on the *deletion* rate *μ*; indeed, Equation 31 does not depend on the insertion rate at all. If one desires insertion and deletion rates to be independent parameters, it is more logical to expect that the observed frequencies of deletions and insertions should be in general different, and should depend on both deletion and insertion rates. This is the case in our model, shown in Equations 16 and 17. At one extreme, for *λ* = 0 (zero insertion rate), 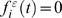 and 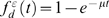. At another extreme, for *λ* = ∞, 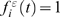, 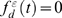 for any positive time *t*.

However, this is not to say that our model is free of its own problems. Most significantly, our model's assumption of column independence is problematic. Rather, in return for this simplification, our model's advantage is that it allows computationally efficient likelihood inference while using a birth-death generative model allowing arbitrary rates of insertion and deletion.

## Materials and Methods

The C source code for the modified phylip 3.66 package [14] that contains the program dnaml *ε* , the C source code for evolving sequences with the generative model (*ε* rate ), the modified rose package (version 1.3) [76], as well as all the Perl scripts and datasets used to generate the results presented in this paper are provided as a tarball in Dataset S1. The program dnaml *ε* uses the easel sequence analysis library (SRE, unpublished) which is also provided.

## Supporting Information

Dataset S1 Supplemental Material
(24.89 MB GZ)

Text S1 Appendix 1
(0.16 MB PDF)

Text S2 Appendix 2
(0.11 MB PDF)

Text S3 Appendix 3
(0.14 MB PDF)

Text S4 Appendix 4
(0.13 MB PDF)
