# Supplementary material for: Probabilistic Phylogenetic Inference with Insertions and Deletions
Source: PLoS Comput Biol. 2008 Sep 19;4(9):e1000172. doi: 10.1371/journal.pcbi.1000172 (PMC2527138; doi:10.1371/journal.pcbi.1000172)
Supplement: Dataset S1 — Supplemental Material (24.89 MB GZ) [file pcbi.1000172.s001.gz › erate-supplement-R2/src/phylip3.66-erate/doc/dnapenny.html]

dnapenny


version 3.66

# Dnapenny - Branch and bound to find all most parsimonious trees for nucleic acid sequence parsimony criteria

© Copyright 1986-2006 by The University of
Washington. Written by Joseph Felsenstein. Permission is granted to copy
this document provided that no fee is charged for it and that this copyright
notice is not removed.

Dnapenny is a program that will find all of the most parsimonious trees
implied by your data when the nucleic acid sequence parsimony criterion is
employed. It does so not by examining all possible trees,
but by using the more sophisticated "branch and bound" algorithm, a
standard computer science search strategy first applied to
phylogenetic inference by Hendy and Penny (1982). (J. S. Farris
[personal communication, 1975] had also suggested that this strategy,
which is well-known in computer science, might
be applied to phylogenies, but he did not publish this suggestion).

There is, however, a price to be paid for the certainty that one has
found all members of the set of most parsimonious trees. The problem of
finding these has been shown (Graham and Foulds, 1982; Day, 1983) to be
NP-complete, which is equivalent to saying that there is no
fast algorithm that is guaranteed to solve the problem in all cases
(for a discussion of NP-completeness, see the Scientific American
article by Lewis and Papadimitriou, 1978). The result is that this program,
despite its algorithmic sophistication, is VERY SLOW.

The program should be slower than the other tree-building programs
in the package, but useable up to about ten species. Above this it will
bog down rapidly, but exactly when depends on the data and on how much
computer time you have (it may be more effective in the hands of someone
who can let a microcomputer grind all night than for someone who
has the "benefit" of paying for time on the campus mainframe computer). IT
IS VERY IMPORTANT FOR YOU TO GET A FEEL FOR HOW LONG THE PROGRAM
WILL TAKE ON YOUR DATA. This can be done by running it on subsets
of the species, increasing the number of species in the run until you
either are able to treat the full data set or know that the program
will take unacceptably long on it. (Making a plot of the logarithm of
run time against species number may help to project run times).

## The Algorithm

The search strategy used by Dnapenny starts by making a tree consisting of the
first two species (the first three if the tree is to be unrooted). Then
it tries to add the next species in all possible places (there are three
of these). For each of the resulting trees it evaluates the number of
base substitutions. It adds the next species to each of these, again in all
possible spaces. If this process would continue it would simply
generate all possible trees, of which there are a very large number even
when the number of species is moderate (34,459,425 with 10 species). Actually
it does not do this, because the trees are generated in a
particular order and some of them are never generated.

This is because the order in which trees are generated is not quite as implied
above, but is a "depth-first search". This means that first one adds the third
species in the first possible place, then the fourth species in its first
possible place, then the fifth and so on until the first possible tree has
been produced. For each tree the number of steps is evaluated. Then one
"backtracks" by trying the alternative placements of the last species. When
these are exhausted one tries the next placement of the next-to-last
species. The order of placement in a depth-first search is like this for a
four-species case (parentheses enclose monophyletic groups):

     Make tree of first two species:     (A,B)  
          Add C in first place:     ((A,B),C)  
               Add D in first place:     (((A,D),B),C)  
               Add D in second place:     ((A,(B,D)),C)  
               Add D in third place:     (((A,B),D),C)  
               Add D in fourth place:     ((A,B),(C,D))  
               Add D in fifth place:     (((A,B),C),D)  
          Add C in second place:     ((A,C),B)  
               Add D in first place:     (((A,D),C),B)  
               Add D in second place:     ((A,(C,D)),B)  
               Add D in third place:     (((A,C),D),B)  
               Add D in fourth place:     ((A,C),(B,D))  
               Add D in fifth place:     (((A,C),B),D)  
          Add C in third place:     (A,(B,C))  
               Add D in first place:     ((A,D),(B,C))  
               Add D in second place:     (A,((B,D),C))  
               Add D in third place:     (A,(B,(C,D)))  
               Add D in fourth place:     (A,((B,C),D))  
               Add D in fifth place:     ((A,(B,C)),D)  

Among these fifteen trees you will find all of the four-species
rooted trees, each exactly once (the parentheses each enclose
a monophyletic group). As displayed above, the backtracking
depth-first search algorithm is just another way of producing all
possible trees one at a time. The branch and bound algorithm
consists of this with one change. As each tree is constructed,
including the partial trees such as (A,(B,C)), its number of steps
is evaluated. In addition a prediction is made as to how many
steps will be added, at a minimum, as further species are added.

This is done by counting how many sites which are invariant in the data up the
most recent species added will ultimately show variation when further species
are added. Thus if 20 sites vary among species A, B, and C and their root,
and if tree ((A,C),B) requires 24 steps, then if there are 8 more sites which
will be seen to vary when species D is added, we can immediately say that no
matter how we add D, the resulting tree can have no less than 24 + 8 = 32
steps. The point of all this is that if a previously-found tree such as
((A,B),(C,D)) required only 30 steps, then we know that there is no point in
even trying to add D to ((A,C),B). We have computed the bound that enables us
to cut off a whole line of inquiry (in this case five trees) and avoid going
down that particular branch any farther.

The branch-and-bound algorithm thus allows us to find all most parsimonious
trees without generating all possible trees. How much of a saving this
is depends strongly on the data. For very clean (nearly "Hennigian")
data, it saves much time, but on very messy data it will still take
a very long time.

The algorithm in the program differs from the one outlined here
in some essential details: it investigates possibilities in the
order of their apparent promise. This applies to the order of addition
of species, and to the places where they are added to the tree. After
the first two-species tree is constructed, the program tries adding
each of the remaining species in turn, each in the best possible place it
can find. Whichever of those species adds (at a minimum) the most
additional steps is taken to be the one to be added next to the tree. When
it is added, it is added in turn to places which cause the fewest
additional steps to be added. This sounds a bit complex, but it is done
with the intention of eliminating regions of the search of all possible
trees as soon as possible, and lowering the bound on tree length as quickly
as possible. This process of evaluating which species to add in which
order goes on the first time the search makes a tree; thereafter it uses that
order.

The program keeps a list of all the most parsimonious
trees found so far. Whenever it finds one that has fewer losses than
these, it clears out the list and
restarts it with that tree. In the process the bound tightens and
fewer possibilities need be investigated. At the end the list contains
all the shortest trees. These are then printed out. It should be
mentioned that the program Clique for finding all largest cliques
also works by branch-and-bound. Both problems are NP-complete but for
some reason Clique runs far faster. Although their worst-case behavior
is bad for both programs, those worst cases occur far more frequently
in parsimony problems than in compatibility problems.

## Controlling Run Times

Among the quantities available to be set from the menu of
Dnapenny, two (howoften and howmany) are of particular
importance. As Dnapenny goes along it will keep count of how many
trees it has examined. Suppose that howoften is 100 and howmany is 1000,
the default settings. Every time 100 trees have been examined, Dnapenny
will print out a line saying how many multiples of 100 trees have now been
examined, how many steps the most parsimonious tree found so far has,
how many trees of with that number of steps have been found, and a very
rough estimate of what fraction of all trees have been looked at so far.

When the number of these multiples printed out reaches the number howmany
(say 1000), the whole algorithm aborts and prints out that it has not
found all most parsimonious trees, but prints out what is has got so far
anyway. These trees need not be any of the most parsimonious trees: they are
simply the most parsimonious ones found so far. By setting the product
(howoften times howmany) large you can make
the algorithm less likely to abort, but then you risk getting bogged
down in a gigantic computation. You should adjust these constants so that
the program cannot go beyond examining the number of trees you are reasonably
willing to pay for (or wait for). In their initial setting the program will
abort after looking at 100,000 trees. Obviously you may want to adjust
howoften in order to get more or fewer lines of intermediate notice of how
many trees have been looked at so far. Of course, in small cases you may
never even reach the first multiple of howoften, and nothing will be printed
out except some headings and then the final trees.

The indication of the approximate percentage of trees searched so far will
be helpful in judging how much farther you would have to go to get the full
search. Actually, since that fraction is the fraction of the set of all
possible trees searched or ruled out so far, and since the search becomes
progressively more efficient, the approximate fraction printed out will
usually be an underestimate of how far along the program is, sometimes a
serious underestimate.

A constant
at the beginning of the program that affects the result is
"maxtrees",
which controls the
maximum number of trees that can be stored. Thus if maxtrees is 25,
and 32 most parsimonious trees are found, only the first 25 of these are
stored and printed out. If maxtrees is increased, the program does not
run any slower but requires a little
more intermediate storage space. I recommend
that maxtrees be kept as large as you can, provided you are willing to
look at an output with that many trees on it! Initially, maxtrees is set
to 100 in the distribution copy.

## Method and Options

The counting of the length of trees is done by an algorithm nearly
identical to the corresponding algorithms in Dnapars, and thus the remainder
of this document will be nearly identical to the Dnapars document.

This program carries out unrooted parsimony (analogous to Wagner
trees) (Eck and Dayhoff, 1966; Kluge and Farris, 1969) on DNA
sequences. The method of Fitch (1971) is used to count the number of
changes of base needed on a given tree. The assumptions of this
method are exactly analogous to those of Dnapars:

1. Each site evolves independently.- Different lineages evolve independently.- The probability of a base substitution at a given site is
       small over the lengths of time involved in
       a branch of the phylogeny.- The expected amounts of change in different branches of the phylogeny
         do not vary by so much that two changes in a high-rate branch
         are more probable than one change in a low-rate branch.- The expected amounts of change do not vary enough among sites that two
           changes in one site are more probable than one change in another.

Change from an occupied site to a deletion is counted as one
change. Reversion from a deletion to an occupied site is allowed and is also
counted as one change.

That these are the assumptions of parsimony methods has been documented
in a series of papers of mine: (1973a, 1978b, 1979, 1981b,
1983b, 1988b). For an
opposing view arguing that the parsimony methods make no substantive
assumptions such as these, see the papers by Farris (1983) and Sober (1983a,
1983b), but also read the exchange between Felsenstein and Sober (1986).

Change from an occupied site to a deletion is counted as one
change. Reversion from a deletion to an occupied site is allowed and is also
counted as one change. Note that this in effect assumes that a deletion
N bases long is N separate events.

The input data is standard. The first line of the input file contains the
number of species and the number of sites. If the Weights option is being
used, there must also be a W in this first line to signal its presence.
There are only two options requiring information to be present in the input
file, W (Weights) and U (User tree). All options other than W (including U) are
invoked using the menu.

Next come the species data. Each
sequence starts on a new line, has a ten-character species name
that must be blank-filled to be of that length, followed immediately
by the species data in the one-letter code. The sequences must either
be in the "interleaved" or "sequential" formats
described in the Molecular Sequence Programs document. The I option
selects between them. The sequences can have internal
blanks in the sequence but there must be no extra blanks at the end of the
terminated line. Note that a blank is not a valid symbol for a deletion.

The options are selected using an interactive menu. The menu looks like this:

|  |
| --- |
| ``` Penny algorithm for DNA, version 3.6  branch-and-bound to find all most parsimonious trees  Settings for this run:   H        How many groups of  100 trees:  1000   F        How often to report, in trees:   100   S           Branch and bound is simple?  Yes   O                        Outgroup root?  No, use as outgroup species  1   T              Use Threshold parsimony?  No, use ordinary parsimony   W                       Sites weighted?  No   M           Analyze multiple data sets?  No   I          Input sequences interleaved?  Yes   0   Terminal type (IBM PC, ANSI, none)?  ANSI   1    Print out the data at start of run  No   2  Print indications of progress of run  Yes   3                        Print out tree  Yes   4          Print out steps in each site  No   5  Print sequences at all nodes of tree  No   6       Write out trees onto tree file?  Yes  Are these settings correct? (type Y or the letter for one to change) ``` |

The user either types "Y" (followed, of course, by a carriage-return)
if the settings shown are to be accepted, or the letter or digit corresponding
to an option that is to be changed.

The options O, T, W, M, and 0 are the usual ones. They are described in the
main documentation file of this package. Option I is the same as in
other molecular sequence programs and is described in the documentation file
for the sequence programs.

The T (threshold) option allows a continuum of methods
between parsimony and compatibility. Thresholds less than or equal to 1.0 do
not have any meaning and should
not be used: they will result in a tree dependent only on the input
order of species and not at all on the data!

The W (Weights) option allows only weights of 0 or 1.

The M (Multiple data sets) option for this program does not allow multiple
sets of weights. We hope to change this soon.

The options H, F, and S are not found in the other molecular sequence programs.
H (How many) allows the user to set the quantity howmany, which we have
already seen controls number of times that the program
will report on its progress. F allows the user to set the quantity howoften,
which sets how often it will report -- after scanning how many trees.

The S (Simple) option alters a step in Dnapenny which reconsiders the
order in which species are added to the tree. Normally the decision as to
what species to add to the tree next is made as the first tree is being
constructed; that ordering of species is not altered subsequently. The S
option causes it to be continually reconsidered. This will probably
result in a substantial increase in run time, but on some data sets of
intermediate messiness it may help. It is included in case it might prove
of use on some data sets. The Simple option, in which the ordering is
kept the same after being established by trying alternatives during
the construction of the first tree, is the default. Continual reconsideration
can be selected as an alternative.

Output is standard: if option 1 is toggled on, the data is printed out,
with the convention that "." means "the same as in the first species".
Then comes a list of equally parsimonious trees, and (if option 2 is
toggled on) a table of the
number of changes of state required in each character. If option 5 is toggled
on, a table is printed
out after each tree, showing for each branch whether there are known to be
changes in the branch, and what the states are inferred to have been at the
top end of the branch. If the inferred state is a "?" or one of the IUB
ambiguity symbols, there will be multiple
equally-parsimonious assignments of states; the user must work these out for
themselves by hand. A "?" in the reconstructed states means that in
addition to one or more bases, a deletion may or may not be present. If
option 6 is left in its default state the trees
found will be written to a tree file, so that they are available to be used
in other programs. If the program finds multiple
trees tied for best, all of these are written out onto the output tree
file. Each is followed by a numerical weight in square brackets (such as
[0.25000]). This is needed when we use the trees to make a consensus
tree of the results of bootstrapping or jackknifing, to avoid overrepresenting
replicates that find many tied trees.

---

### TEST DATA SET

|  |
| --- |
| ```     8    6 Alpha1    AAGAAG Alpha2    AAGAAG Beta1     AAGGGG Beta2     AAGGGG Gamma1    AGGAAG Gamma2    AGGAAG Delta     GGAGGA Epsilon   GGAAAG ``` |

---

### CONTENTS OF OUTPUT FILE (if all numerical options are on)

|  |
| --- |
| ``` Penny algorithm for DNA, version 3.66  branch-and-bound to find all most parsimonious trees   8 species,   6  sites  Name         Sequences ----         ---------  Alpha1       AAGAAG Alpha2       ...... Beta1        ...GG. Beta2        ...GG. Gamma1       .G.... Gamma2       .G.... Delta        GGAGGA Epsilon      GGA...   requires a total of              8.000       9 trees in all found     +--------------------Alpha1       !     !                 +--Delta        !              +--3     !           +--7  +--Epsilon      1           !  !     !     +-----6  +-----Gamma2       !     !     !     !  +--4     +--------Gamma1       !  !  !     !  !  !           +--Beta2        +--2  +-----------5        !              +--Beta1           !        +-----------------Alpha2        remember: this is an unrooted tree!   steps in each site:          0   1   2   3   4   5   6   7   8   9      *-----------------------------------------     0|       1   1   1   2   2   1              From    To     Any Steps?    State at upper node                              ( . means same as in the node below it on tree)            1                AAGAAG    1   Alpha1       no     ......    1      2         no     ......    2      4         no     ......    4      6         yes    .G....    6      7         no     ......    7      3         yes    G.A...    3   Delta        yes    ...GGA    3   Epsilon      no     ......    7   Gamma2       no     ......    6   Gamma1       no     ......    4      5         yes    ...GG.    5   Beta2        no     ......    5   Beta1        no     ......    2   Alpha2       no     ......     +--------------------Alpha1       !     !                 +--Delta        !           +-----3     !           !     +--Epsilon      1     +-----6     !     !     !     +--Gamma2       !     !     +-----7     !  +--4           +--Gamma1       !  !  !     !  !  !           +--Beta2        +--2  +-----------5        !              +--Beta1           !        +-----------------Alpha2        remember: this is an unrooted tree!   steps in each site:          0   1   2   3   4   5   6   7   8   9      *-----------------------------------------     0|       1   1   1   2   2   1              From    To     Any Steps?    State at upper node                              ( . means same as in the node below it on tree)            1                AAGAAG    1   Alpha1       no     ......    1      2         no     ......    2      4         no     ......    4      6         yes    .G....    6      3         yes    G.A...    3   Delta        yes    ...GGA    3   Epsilon      no     ......    6      7         no     ......    7   Gamma2       no     ......    7   Gamma1       no     ......    4      5         yes    ...GG.    5   Beta2        no     ......    5   Beta1        no     ......    2   Alpha2       no     ......     +--------------------Alpha1       !     !                 +--Delta        !              +--3     !           +--6  +--Epsilon      1           !  !     !     +-----7  +-----Gamma1       !     !     !     !  +--4     +--------Gamma2       !  !  !     !  !  !           +--Beta2        +--2  +-----------5        !              +--Beta1           !        +-----------------Alpha2        remember: this is an unrooted tree!   steps in each site:          0   1   2   3   4   5   6   7   8   9      *-----------------------------------------     0|       1   1   1   2   2   1              From    To     Any Steps?    State at upper node                              ( . means same as in the node below it on tree)            1                AAGAAG    1   Alpha1       no     ......    1      2         no     ......    2      4         no     ......    4      7         yes    .G....    7      6         no     ......    6      3         yes    G.A...    3   Delta        yes    ...GGA    3   Epsilon      no     ......    6   Gamma1       no     ......    7   Gamma2       no     ......    4      5         yes    ...GG.    5   Beta2        no     ......    5   Beta1        no     ......    2   Alpha2       no     ......     +--------------------Alpha1       !     !                 +--Delta        !              +--3     1           +--7  +--Epsilon      !           !  !     !  +--------6  +-----Gamma2       !  !        !     !  !        +--------Gamma1       +--2        !              +--Beta2           !           +--5        +-----------4  +--Beta1                       !                    +-----Alpha2        remember: this is an unrooted tree!   steps in each site:          0   1   2   3   4   5   6   7   8   9      *-----------------------------------------     0|       1   1   1   2   2   1              From    To     Any Steps?    State at upper node                              ( . means same as in the node below it on tree)            1                AAGAAG    1   Alpha1       no     ......    1      2         no     ......    2      6         yes    .G....    6      7         no     ......    7      3         yes    G.A...    3   Delta        yes    ...GGA    3   Epsilon      no     ......    7   Gamma2       no     ......    6   Gamma1       no     ......    2      4         no     ......    4      5         yes    ...GG.    5   Beta2        no     ......    5   Beta1        no     ......    4   Alpha2       no     ......     +--------------------Alpha1       !     !                 +--Delta        !           +-----3     1           !     +--Epsilon      !  +--------6     !  !        !     +--Gamma2       !  !        +-----7     +--2              +--Gamma1          !        !              +--Beta2           !           +--5        +-----------4  +--Beta1                       !                    +-----Alpha2        remember: this is an unrooted tree!   steps in each site:          0   1   2   3   4   5   6   7   8   9      *-----------------------------------------     0|       1   1   1   2   2   1              From    To     Any Steps?    State at upper node                              ( . means same as in the node below it on tree)            1                AAGAAG    1   Alpha1       no     ......    1      2         no     ......    2      6         yes    .G....    6      3         yes    G.A...    3   Delta        yes    ...GGA    3   Epsilon      no     ......    6      7         no     ......    7   Gamma2       no     ......    7   Gamma1       no     ......    2      4         no     ......    4      5         yes    ...GG.    5   Beta2        no     ......    5   Beta1        no     ......    4   Alpha2       no     ......     +--------------------Alpha1       !     !                 +--Delta        !              +--3     1           +--6  +--Epsilon      !           !  !     !  +--------7  +-----Gamma1       !  !        !     !  !        +--------Gamma2       +--2        !              +--Beta2           !           +--5        +-----------4  +--Beta1                       !                    +-----Alpha2        remember: this is an unrooted tree!   steps in each site:          0   1   2   3   4   5   6   7   8   9      *-----------------------------------------     0|       1   1   1   2   2   1              From    To     Any Steps?    State at upper node                              ( . means same as in the node below it on tree)            1                AAGAAG    1   Alpha1       no     ......    1      2         no     ......    2      7         yes    .G....    7      6         no     ......    6      3         yes    G.A...    3   Delta        yes    ...GGA    3   Epsilon      no     ......    6   Gamma1       no     ......    7   Gamma2       no     ......    2      4         no     ......    4      5         yes    ...GG.    5   Beta2        no     ......    5   Beta1        no     ......    4   Alpha2       no     ......     +--------------------Alpha1       !     !                 +--Delta        !              +--3     !           +--7  +--Epsilon      1           !  !     !        +--6  +-----Gamma2       !        !  !     !  +-----2  +--------Gamma1       !  !     !     +--4     +-----------Alpha2          !        !              +--Beta2           +--------------5                       +--Beta1         remember: this is an unrooted tree!   steps in each site:          0   1   2   3   4   5   6   7   8   9      *-----------------------------------------     0|       1   1   1   2   2   1              From    To     Any Steps?    State at upper node                              ( . means same as in the node below it on tree)            1                AAGAAG    1   Alpha1       no     ......    1      4         no     ......    4      2         no     ......    2      6         yes    .G....    6      7         no     ......    7      3         yes    G.A...    3   Delta        yes    ...GGA    3   Epsilon      no     ......    7   Gamma2       no     ......    6   Gamma1       no     ......    2   Alpha2       no     ......    4      5         yes    ...GG.    5   Beta2        no     ......    5   Beta1        no     ......     +--------------------Alpha1       !     !                 +--Delta        !           +-----3     !           !     +--Epsilon      1        +--6     !        !  !     +--Gamma2       !  +-----2  +-----7     !  !     !        +--Gamma1       !  !     !     +--4     +-----------Alpha2          !        !              +--Beta2           +--------------5                       +--Beta1         remember: this is an unrooted tree!   steps in each site:          0   1   2   3   4   5   6   7   8   9      *-----------------------------------------     0|       1   1   1   2   2   1              From    To     Any Steps?    State at upper node                              ( . means same as in the node below it on tree)            1                AAGAAG    1   Alpha1       no     ......    1      4         no     ......    4      2         no     ......    2      6         yes    .G....    6      3         yes    G.A...    3   Delta        yes    ...GGA    3   Epsilon      no     ......    6      7         no     ......    7   Gamma2       no     ......    7   Gamma1       no     ......    2   Alpha2       no     ......    4      5         yes    ...GG.    5   Beta2        no     ......    5   Beta1        no     ......     +--------------------Alpha1       !     !                 +--Delta        !              +--3     !           +--6  +--Epsilon      1           !  !     !        +--7  +-----Gamma1       !        !  !     !  +-----2  +--------Gamma2       !  !     !     +--4     +-----------Alpha2          !        !              +--Beta2           +--------------5                       +--Beta1         remember: this is an unrooted tree!   steps in each site:          0   1   2   3   4   5   6   7   8   9      *-----------------------------------------     0|       1   1   1   2   2   1              From    To     Any Steps?    State at upper node                              ( . means same as in the node below it on tree)            1                AAGAAG    1   Alpha1       no     ......    1      4         no     ......    4      2         no     ......    2      7         yes    .G....    7      6         no     ......    6      3         yes    G.A...    3   Delta        yes    ...GGA    3   Epsilon      no     ......    6   Gamma1       no     ......    7   Gamma2       no     ......    2   Alpha2       no     ......    4      5         yes    ...GG.    5   Beta2        no     ......    5   Beta1        no     ...... ``` |
