# Supplementary material for: Probabilistic Phylogenetic Inference with Insertions and Deletions
Source: PLoS Comput Biol. 2008 Sep 19;4(9):e1000172. doi: 10.1371/journal.pcbi.1000172 (PMC2527138; doi:10.1371/journal.pcbi.1000172)
Supplement: Dataset S1 — Supplemental Material (24.89 MB GZ) [file pcbi.1000172.s001.gz › erate-supplement-R2/src/phylip3.66-erate/doc/consense.html]

consense


version 3.66

# Consense -- Consensus tree program

© Copyright 1986-2006 by The University of
Washington. Written by Joseph Felsenstein. Permission is granted to copy
this document provided that no fee is charged for it and that this copyright
notice is not removed.

Consense reads a file of computer-readable trees and prints
out (and may also write out onto a file) a consensus tree. At the moment
it carries out a family of consensus tree methods called the
*Ml*
methods (Margush and McMorris, 1981). These include strict consensus and
majority rule consensus. Basically the
consensus tree consists of monophyletic groups
that occur as often as possible in the data. If a group occurs in more than
a fraction *l* of all the input trees it will definitely
appear in the consensus tree.

The tree printed out has at each fork a number indicating how many times the
group which consists of the species to the right of (descended from) the fork
occurred. Thus if we read in 15 trees and find that a fork has the number
15, that group occurred in all of the trees. The strict consensus tree
consists of all groups that occurred 100% of the time, the rest of the
resolution being ignored. The tree printed out here includes groups down
to 50%, and below it until the tree is fully resolved.

The majority rule consensus tree consists of all groups that occur more than
50% of the time. Any other percentage level between 50% and 100% can also
be used, and that is why the program in effect
carries out a family of methods. You
have to decide on the percentage level, figure out for yourself what number
of occurrences that would be (e.g. 15 in the above case for 100%), and
resolutely ignore any group below that number. Do not use numbers at or below
50%, because some groups occurring (say) 35% of the time will not be shown
on the tree. The collection of all groups that occur 35% or more of the
time may include two groups that are mutually self contradictory and cannot
appear in the same tree. In this program, as the default method I have
included groups that occur
less than 50% of the time, working downwards in their frequency of occurrence,
as long as they continue to resolve the tree and do not contradict more
frequent groups. In this respect the method is similar to the Nelson consensus
method (Nelson, 1979) as explicated by Page (1989) although it is not identical
to it.

The program can also carry out Strict consensus, Majority Rule consensus
without the extension which adds groups until the tree is fully
resolved, and other members of the Ml family, where the
user supplied the fraction of times the group must appear in the input
trees to be included in the consensus tree.
For the moment the program cannot carry out any other
consensus tree method, such as Adams consensus (Adams, 1972, 1986) or methods
based on
quadruples of species (Estabrook, McMorris, and Meacham, 1985).

## INPUT, OUTPUT, AND OPTIONS

Input is a tree file (called intree)
which contains a series of trees in the Newick
standard form -- the form used when many of the programs in this package
write out tree files. Each tree starts on a new line. Each tree can have
a weight, which is a real number and is located in comment brackets "["
and "]" just before the final ";" which
ends the description of the tree. When the input trees have weights
(like [0.01000]) then the total number of trees will be the total of those
weights, which is often a number like 1.00. When the a tree doesn't have
a weight it will each be assigned a weight of 1. This means that when we have
tied trees (as from a parsimony program) three alternative tied trees will
be counted as if each was 1/3 of a tree.

Note that this program can correctly
read trees whether or not they are bifurcating: in fact they can be
multifurcating at any level in the tree.

The options are selected from a menu, which looks like this:

|  |
| --- |
| ``` Consensus tree program, version 3.6  Settings for this run:  C         Consensus type (MRe, strict, MR, Ml):  Majority rule (extended)  O                                Outgroup root:  No, use as outgroup species  1  R                Trees to be treated as Rooted:  No  T           Terminal type (IBM PC, ANSI, none):  ANSI  1                Print out the sets of species:  Yes  2         Print indications of progress of run:  Yes  3                               Print out tree:  Yes  4               Write out trees onto tree file:  Yes  Are these settings correct? (type Y or the letter for one to change) ``` |

Option C (Consensus method) selects which of four methods the
program uses. The program defaults to using the extended Majority
Rule method. Each time the C option is chosen the program moves on
to another method, the others being in order Strict, Majority Rule,
and Ml. Here are descriptions of the methods. In each
case the fraction of times a set appears among the input trees
is counted by weighting by the weights of the trees (the numbers
like [0.6000] that appear at the ends of trees in some
cases).

Strict: A set of species must appear in all input trees to be included in the strict consensus tree. Majority Rule (extended): Any set of species that appears in more than 50% of the trees is included. The program then considers the other sets of species in order of the frequency with which they have appeared, adding to the consensus tree any which are compatible with it until the tree is fully resolved. This is the default setting. Ml: The user is asked for a fraction between 0.5 and 1, and the program then includes in the consensus tree any set of species that occurs among the input trees more than that fraction of then time. The Strict consensus and the Majority Rule consensus are extreme cases of the Ml consensus, being for fractions of 1 and 0.5 respectively. Majority Rule: A set of species is included in the consensus tree if it is present in more than half of the input trees.

Option R (Rooted) toggles between the default assumption that the input trees
are unrooted trees and the selection that
specifies that the tree is to be treated as a rooted tree and not
re-rooted. Otherwise the tree will be treated as outgroup-rooted and will
be re-rooted automatically at the first species encountered on the first
tree (or at a species designated by the Outgroup option).

Option O is the usual Outgroup rooting option. It is in effect only if
the Rooted option selection is not in effect. The trees will be re-rooted
with a species of your choosing. You will be asked for the number of the
species that is to be the outgroup. If we want to outgroup-root the tree on
the line leading to a
species which appears as the third species (counting left-to-right) in the
first computer-readable tree in the input file, we would invoke select
menu option O and specify species 3.

Output is a list of the species (in the order in which they appear in the
first tree, which is the numerical order used in the program), a list
of the subsets that appear in the consensus tree, a list of those that
appeared in one or another of the individual
trees but did not occur frequently enough to get into the consensus tree,
followed by a diagram showing the consensus tree. The lists of subsets
consists of a row of symbols, each either "." or "\*". The species
that are in the set are marked by "\*". Every ten species there is
a blank, to help you keep track of the alignment of columns. The
order of symbols corresponds to the order of species in the species
list. Thus a set that consisted of the second, seventh, and eighth out
of 13 species would be represented by:

```
          .*....**.. ...
```

Note that if the trees are unrooted the final tree will have one group,
consisting of every species except the Outgroup (which by default is the
first species encountered on the first tree), which always appears. It
will not be listed in either of the lists of sets, but it will be shown in
the final tree as occurring all of the time. This is hardly surprising:
in telling the program that this species is the outgroup we have specified
that the set consisting of all of the others is always a monophyletic set. So
this is not to be taken as interesting information, despite its dramatic
appearance.

Option 2 in the menu gives you the option of turning off the writing of
these sets into the output file. This may be useful if you are primarily
interested in getting the tree file.

Option 3 is the usual tree file option. If this is on (it is by default)
then the final tree will be written onto an output tree file (whose default
name is "outtree").

## Branch Lengths on the Consensus Tree?

Note that the lengths on the tree on the output tree file
are not branch lengths but the number of times that
each group appeared in the input trees. This
number is the sum of the weights of the trees in which it appeared, so that
if there are 11 trees, ten of them having weight 0.1 and one weight 1.0,
a group that appeared in the last tree and in 6 others would be shown as
appearing 1.6 times and its branch length will be 1.6.
This means that if you take the consensus tree from the output tree file
and try to draw it, the branch lengths will be strange. I am often asked
how to put the correct branch lengths on these (this is one of our
Frequently Asked Questions).

There is no simple answer to this. It depends on what "correct" means.
For example, if you have a group of species that shows up in 80% of the trees,
and the branch leading to that group has average length 0.1 among that
80%, is the "correct" length 0.1? Or is it (0.80 x 0.1)? There is no
simple answer.

However, if you want to take the consensus tree as an estimate of the
true tree (rather than as an indicator of the conflicts among trees)
you may be able to use the User Tree (option U) mode of the phylogeny
program that you used, and use it to put branch lengths on that tree.
Thus, if you used Dnaml, you can take the consensus tree, make sure it
is an unrooted tree, and feed that to Dnaml using the original data set
(before bootstrapping) and Dnaml's option U. As
Dnaml wants an unrooted tree, you may have to use Retree to make the tree
unrooted (using the W option of Retree and choosing the unrooted option
within it). Of course you will also want to change the tree file name
from "outree" to "intree".

If you used a phylogeny program that does not infer branch lengths, you
might want to use a different one (such as Fitch or Dnaml) to infer the
branch lengths, again making sure the tree is unrooted, if the program
needs that.

## CONSTANTS

The program uses the consensus tree algorithm originally designed for
the bootstrap programs. It is quite fast, and execution time is unlikely
to be limiting for you (assembling the input file will be much more of a
limiting step). In the future, if possible, more consensus tree methods
will be incorporated (although the current methods are the ones needed
for the component analysis of bootstrap estimates of phylogenies, and in
other respects I also think that the
present ones are among the best).

```
---


### TEST SET OF INPUT TREES


|  |
| --- |
| ``` (A,(B,(H,(D,(J,(((G,E),(F,I)),C)))))); (A,(B,(D,((J,H),(((G,E),(F,I)),C))))); (A,(B,(D,(H,(J,(((G,E),(F,I)),C)))))); (A,(B,(E,(G,((F,I),((J,(H,D)),C)))))); (A,(B,(E,(G,((F,I),(((J,H),D),C)))))); (A,(B,(E,((F,I),(G,((J,(H,D)),C)))))); (A,(B,(E,((F,I),(G,(((J,H),D),C)))))); (A,(B,(E,((G,(F,I)),((J,(H,D)),C))))); (A,(B,(E,((G,(F,I)),(((J,H),D),C))))); ``` |


---


### TEST SET OUTPUT


|  |
| --- |
| ``` Consensus tree program, version 3.66  Species in order:     1. A   2. B   3. H   4. D   5. J   6. G   7. E   8. F   9. I   10. C   Sets included in the consensus tree  Set (species in order)     How many times out of    9.00  .......**.                   9.00 ..********                   9.00 ..****.***                   6.00 ..***.....                   6.00 ..***....*                   6.00 ..*.*.....                   4.00 ..***..***                   2.00   Sets NOT included in consensus tree:  Set (species in order)     How many times out of    9.00  .....**...                   3.00 .....*****                   3.00 ..**......                   3.00 .....****.                   3.00 ..****...*                   2.00 .....*.**.                   2.00 ..*.******                   2.00 ....******                   2.00 ...*******                   1.00   Extended majority rule consensus tree  CONSENSUS TREE: the numbers on the branches indicate the number of times the partition of the species into the two sets which are separated by that branch occurred among the trees, out of   9.00 trees                                       +--------------------C                                      |                               +--6.0-|             +------H                               |      |      +--4.0-|                               |      +--6.0-|      +------J                        +--2.0-|             |                        |      |             +-------------D                        |      |                 +--6.0-|      |                    +------F                 |      |      +----------------9.0-|                 |      |                           +------I          +--9.0-|      |          |      |      +----------------------------------G   +------|      |   |      |      +-----------------------------------------E   |      |   |      +------------------------------------------------B   |   +-------------------------------------------------------A     remember: this is an unrooted tree! ``` |
```
