# Supplementary material for: Probabilistic Phylogenetic Inference with Insertions and Deletions
Source: PLoS Comput Biol. 2008 Sep 19;4(9):e1000172. doi: 10.1371/journal.pcbi.1000172 (PMC2527138; doi:10.1371/journal.pcbi.1000172)
Supplement: Dataset S1 — Supplemental Material (24.89 MB GZ) [file pcbi.1000172.s001.gz › erate-supplement-R2/src/phylip3.66-erate/doc/clique.html]

clique


version 3.66

# Clique -- Compatibility Program

© Copyright 1986-2006 by the University of
Washington. Written by Joseph Felsenstein. Permission is granted to copy
this document provided that no fee is charged for it and that this copyright
notice is not removed.

This program uses the compatibility method for unrooted two-state
characters to obtain the largest cliques of characters and the trees
which they suggest. This approach originated in the work of Le Quesne
(1969), though the algorithms were not precisely specified until the
later work of Estabrook, Johnson, and McMorris (1976a, 1976b). These
authors proved the theorem that a group of two-state characters which
were pairwise compatible would be jointly compatible. This program uses
an algorithm inspired by the Kent Fiala - George Estabrook program
CLINCH, though closer in detail to the algorithm of Bron and Kerbosch
(1973). I am indebted to Kent Fiala for pointing out that paper to me,
and to David Penny for decribing to me his branch-and-bound approach to
finding largest cliques, from which I have also borrowed. I am
particularly grateful to Kent Fiala for catching a bug in versions 2.0
and 2.1 which resulted in those versions failing to find all of the
cliques which they should. The program computes a compatibility matrix
for the characters, then uses a recursive procedure to examine all
possible cliques of characters.

After one pass through all possible cliques, the program knows the
size of the largest clique, and during a second pass it prints out the
cliques of the right size. It also, along with each clique, prints out a
the tree suggested by that clique.

## INPUT, OUTPUT, AND OPTIONS

Input to the algorithm is
standard, but the "?", "P", and "B" states are not allowed. This is a
serious limitation of this program. If you want to find large cliques in
data that has "?" states, I recommend that you use MIX instead with the T
(Threshold) option and the value of the threshold set to 2.0. The theory
underlying this is given in my paper on character weighting (Felsenstein,
1981b).

The options are chosen from a menu, which looks like this:

|  |
| --- |
| ``` Largest clique program, version 3.6  Settings for this run:   A   Use ancestral states in input file?  No   F              Use factors information?  No   W                       Sites weighted?  No   C          Specify minimum clique size?  No   O                        Outgroup root?  No, use as outgroup species  1   M           Analyze multiple data sets?  No   0   Terminal type (IBM PC, ANSI, none)?  ANSI   1    Print out the data at start of run  No   2  Print indications of progress of run  Yes   3        Print out compatibility matrix  No   4                        Print out tree  Yes   5       Write out trees onto tree file?  Yes    Y to accept these or type the letter for one to change ``` |

The A (Ancestors), F (Factors), O (Outgroup) ,M (Multiple Data Sets),
and W (Weights)
options are the usual ones, described in the main
documentation file.

If you use option A (Ancestors) you should also choose it in the menu.
The compatibility matrix calculation in effect
assumes if the Ancestors option is invoked that there is in the data
another species that has all the ancestral states. This changes the
compatibility patterns in the proper way. The Ancestors option also
requires information on the ancestral states of each character to be in
the input file.

The O (Outgroup) option will take effect only if the tree is not rooted by the
Ancestral States option.

The C (Clique Size) option indicates that
you wish to specify a minimum clique size and print out all cliques (and their
associated trees) greater than or equal to than that size. The program
prompts you for the minimum clique size.

Note that this allows you to list all cliques (each with its tree) by
simply setting the minimum clique size to 1. If you do one run and find
that the largest clique has 23 characters, you can do another run with
the minimum clique size set at 18, thus listing all cliques within 5
characters of the largest one.

Output involves a compatibility matrix (using the symbols "." and "1")
and the cliques and trees.

If you have used the F option there will be two lists of characters
for each clique, one the original multistate characters and the other the
binary characters. It is the latter that are shown on the tree. When the
F option is not used the output and the cliques reflect only the binary
characters.

The trees produced have it
indicated on each branch the points at which derived character states
arise in the characters that define the clique. There is a legend
above the tree showing which binary character is involved. Of course
if the tree is unrooted you can read the changes as going in either
direction.

The program runs very quickly but if the
maximum number of characters is large it will need a good deal of
storage, since the compatibility matrix requires ActualChars x ActualChars
boolean variables, where ActualChars is the number of characters (in the
case of the factors option the total number of true multistate characters.

## ASSUMPTIONS

Basically the following assumptions are made:

1. Each character evolves independently.- Different lineages evolve independently.- The ancestral state is not known.- Each character has a small chance of being one which evolves
         so rapidly, or is so thoroughly misinterpreted, that it
         provides no information on the tree.- The probability of a single change in a character (other than
           in the high rate characters) is low but not as low as the
           probability of being one of these "bad" characters.- The probability of two changes in a low-rate character is much
             less than the probability that it is a high-rate character.- The true tree has segments which are not so unequal in length
               that two changes in a long are as easy to envisage as one
               change in a short segment.

The assumptions of compatibility methods have been treated in
several of my papers (1978b, 1979, 1981b, 1988b), especially the 1981
paper. For an opposing view arguing that the parsimony methods
make no substantive
assumptions such as these, see the papers by Farris (1983) and Sober (1983a,
1983b), but also read the exchange between Felsenstein and Sober (1986).

A constant available for alteration at the beginning of the
program is the form width, "FormWide",
which you may want to change to make it as large as possible
consistent with the page width available on your output device,
so as to avoid the output of cliques and of trees getting wrapped
around unnecessarily.

---

### TEST DATA SET

|  |
| --- |
| ```      5    6 Alpha     110110 Beta      110000 Gamma     100110 Delta     001001 Epsilon   001110 ``` |

---

### TEST SET OUTPUT (with all numerical options on)

|  |
| --- |
| ``` Largest clique program, version 3.66   5 species,   6  characters Species  Character states -------  --------- ------  Alpha       11011 0 Beta        11000 0 Gamma       10011 0 Delta       00100 1 Epsilon     00111 0  Character Compatibility Matrix (1 if compatible) --------- ------------- ------ -- -- -----------                       111..1                      111..1                      111..1                      ...111                      ...111                      111111   Largest Cliques ------- -------   Characters: (  1  2  3  6)     Tree and characters:       2  1  3  6      0  0  1  1               +1-Delta             +0--1-+   +--0-+     +--Epsilon      !    !   !    +--------Gamma        !   +-------------Alpha        !   +-------------Beta        remember: this is an unrooted tree! ``` |
