# Supplementary material for: Probabilistic Phylogenetic Inference with Insertions and Deletions
Source: PLoS Comput Biol. 2008 Sep 19;4(9):e1000172. doi: 10.1371/journal.pcbi.1000172 (PMC2527138; doi:10.1371/journal.pcbi.1000172)
Supplement: Dataset S1 — Supplemental Material (24.89 MB GZ) [file pcbi.1000172.s001.gz › erate-supplement-R2/src/phylip3.66-erate/doc/main.html]

 
 
 
 main 
 
 
 
 
 
 
 
 
 
 PHYLIP 
 Phylogeny Inference Package 
 
 
 
 Version 3.66 
 
 July, 2006 
 
 by Joseph Felsenstein 
 
 
 
  
 
Department of Genome Sciences and Department of Biology 
University of Washington 
Box 357730 
Seattle, WA   98195-7730 
USA
 
  
 
 E-mail address:     joe  (at)  gs.washington.edu  
 
 
 
    
 Contents of This Document  
 
 
 Contents of This Document 
 
 A Brief Description of the Programs 
 
 Copyright Notice for PHYLIP 
 
 The Documentation Files and How to Read Them 
 
 What The Programs Do 
 
 Running the Programs 
 
      A word about input files
 
      Running the programs on a Windows machine
 
      Running the programs on a Macintosh with Mac OS X
 
      Running the programs on a Macintosh with Mac OS 8 or 9
 
      Running the programs on a Unix or Linux system
 
      Running the programs in MSDOS
 
      Running the programs in background or under control of a command file
 
 Preparing Input Files 
 
      Input and output files
 
      Data file format
 
 The Menu 
 
 The Output File 
 
 The Tree File 
 
 The Options and How To Invoke Them 
 
      Common options in the menu
 
        The  U  (User tree) option
 
        The  G  (Global) option
 
        The  J  (Jumble) option
 
        The  O  (Outgroup) option
 
        The  T  (Threshold) option
 
        The  M  (Multiple data sets) option
 
        The  W  (Weights) option
 
        The option to write out the trees into a tree file
 
        The ( 0 ) terminal type option
 
 The Algorithm for Constructing Trees 
 
      Local rearrangements
 
      Global rearrangements
 
      Multiple jumbles
 
      Saving multiple tied trees
 
      Strategy for finding the best tree
 
 A Warning on Interpreting Results 
 
 Relative Speed of Different Programs and Machines 
 
      Relative speed of the different programs
 
      Speed with different numbers of species
 
      Relative speed of different machines
 
 General Comments on Adapting the Package to Different Computer Systems 
 
 Compiling the programs 
 
      Unix and Linux
 
      On Windows systems
 
           Compiling with Cygnus Gnu C++
 
           Compiling with Microsoft Visual C++
 
           Compiling with Borland C++
 
           Compiling with Metrowerks Codewarrior for Windows 
 
      Macintosh
 
           Compiling with GCC on Mac OS X with our Makefile
 
           Compiling with GCC on Mac OS X with X Windows
 
           Compiling with Metrowerks Codewarrior
 
           Creating the Metrowerks project file.
 
      VMS VAX systems
 
      Parallel computers
 
      Other computer systems
 
 Frequently Asked Questions 
 
      How to make it do various things
 
      Background information needed:
 
      Questions about distribution and citation:
 
      Questions about documentation
 
      Additional Frequently Asked Questions, or: "Why didn't it occur to you to ...
 
      (Fortunately) obsolete questions
 
 New Features in This Version 
 
 Coming Attractions, Future Plans 
 
 Endorsements 
 
      From the pages of  Cladistics 
 
      ... in the pages of other journals:
 
      ... and in the comments made by users when they register:
 
 References for the Documentation Files 
 
 Credits 
 
 Other Phylogeny Programs Available Elsewhere 
 
      PAUP*
 
      MacClade
 
      MrBayes
 
      MEGA
 
      PAML
 
      TREE-PUZZLE
 
      DAMBE
 
      Hennig86
 
      RnA
 
      NONA
 
      TNT
 
 How You Can Help Me 
 
 In Case of Trouble 
 
    
 
 A Brief Description of the Programs  
 
 PHYLIP , the Phylogeny Inference Package, is a package of programs for
inferring phylogenies (evolutionary trees).  It has been distributed since
1980, and has over 20,000 registered users, making it the most widely
distributed package of phylogeny programs.  It is available free, from
its web site:
 
 
  
 http://evolution.gs.washington.edu/phylip.html   

 
 
 PHYLIP  is available as source code in C, and also as executables for
some common computer systems.  It can infer phylogenies by parsimony,
compatibility, distance matrix methods, and likelihood.  It can also
compute consensus trees, compute distances between trees, draw trees,
resample data sets by bootstrapping or jackknifing, edit trees, and
compute distance matrices.  It can handle data that are nucleotide
sequences, protein sequences, gene frequencies, restriction sites,
restriction fragments, distances, discrete characters, and continuous
characters.
 
 
    
 
   
 
 Copyright Notice for PHYLIP  
 
The following copyright notice is intended to cover all source code, all
documentation, and all executable programs of the PHYLIP package.
 
© Copyright 1980-2006.  University of Washington.  All
rights reserved.  Permission is granted to reproduce, perform, and modify
these programs and documentation files.  Permission is granted to distribute
or provide access to these
programs provided that this copyright notice is not removed, the programs are
not integrated with or called by any product or service that generates
revenue, and that your distribution of these documentation files and programs
are free.  Any modified
versions of these materials that are distributed or accessible shall indicate
that they are based on these program.  Institutions of higher education are
granted permission to distribute this material to their students and staff
for a fee to recover distribution costs.  Permission requests for any other
distribution of this program should be directed to   license  (at)  u.washington.edu  .
 
    

 
    
 
 The Documentation Files and How to Read Them  
 
 PHYLIP  comes with an extensive set of documentation files.  These
include the main documentation file (this one), which you should read
fairly completely.  In addition there are files for groups of programs,
including ones for the  molecular sequence 
programs, the  distance matrix 
programs, the
 gene frequency and continuous characters 
programs, the  discrete characters  programs,
and the  tree drawing  programs.  Finally,
each program has its own documentation file.  References for the
documentation files are all gathered together in this main documentation
file.  A good strategy is to:
 
 Read this main documentation file.
 Tentatively decide which programs are of interest to you.
 Read the documentation files for the groups of programs that
contain those.
 Read the documentation files for those individual programs.
 
 
There is an excellent guide to using PHYLIP 3.6 also available.  It was written by
Jarno Tuimala of the Center for Scientific Computing in Espoo, Finland and is
available as a PDF  here .
 
    
 
 What The Programs Do  
 
Here is a short description of each of the programs.  For more detailed
discussion you should definitely read the documentation file for the
individual program and the documentation file for the group of programs
it is in.  In this list the name of each program is a link which will
take you to the documentation file for that program.  Note that there is no
program in the PHYLIP package called PHYLIP.
 
   Protpars  
 Estimates phylogenies from protein sequences (input using the
   standard one-letter code for amino acids) using the parsimony method, in
   a variant which counts only those nucleotide changes that change the amino
   acid, on the assumption that silent changes are more easily accomplished.
   Dnapars  
 Estimates phylogenies by the parsimony method using nucleic acid
   sequences.  Allows use the full IUB ambiguity codes, and estimates 
   ancestral nucleotide states.  Gaps treated as a fifth nucleotide state.
   It can also fo transversion parsimony.  Can cope with multifurcations,
   reconstruct ancestral states, use 0/1 character weights, and infer
   branch lengths.
   Dnamove  
 Interactive construction of phylogenies from nucleic acid
   sequences, with their evaluation by parsimony and compatibility and the
   display of reconstructed ancestral bases.  This can be used to find
   parsimony or compatibility estimates by hand.  
   Dnapenny  
 Finds all most parsimonious phylogenies for nucleic acid
   sequences by branch-and-bound search.  This may not be practical (depending
   on the data) for more than 10-11 species or so.
   Dnacomp  
 Estimates phylogenies from nucleic acid sequence data using
   the compatibility criterion, which searches for the largest number of sites 
   which could have all states (nucleotides) uniquely evolved on the same 
   tree.  Compatibility is particularly appropriate when sites vary greatly in 
   their rates of evolution, but we do not know in advance which are the less 
   reliable ones.
   Dnainvar  
 For nucleic acid sequence data on four species, computes
   Lake's and Cavender's phylogenetic invariants, which test alternative tree
   topologies.  The program also tabulates the frequencies of occurrence of the
   different nucleotide patterns.  Lake's invariants are the method which he
   calls "evolutionary parsimony".
   Dnaml  
  Estimates phylogenies from nucleotide sequences by maximum
   likelihood.  The model employed allows for unequal expected frequencies of
   the four nucleotides, for unequal rates of transitions and transversions,
   and for different (prespecified) rates of change in different categories of
   sites, and also use of a Hidden Markov model of rates, with
   the program inferring which sites have which rates.  This also
   allows gamma-distribution and gamma-plus-invariant sites distributions of
   rates across sites.
   Dnamlk  
 Same as Dnaml but assumes a molecular clock.  The use of the
   two programs together permits a likelihood ratio test of the
   molecular clock hypothesis to be made.
   Proml  
 Estimates phylogenies from protein amino acid sequences by maximum 
   likelihood.  The PAM, JTT, or PMB models can be employed, and also use
   of a Hidden Markov model of rates, with the program inferring which sites
   have which rates.  This also allows gamma-distribution and
   gamma-plus-invariant sites distributions of rates across sites.
   It also allows different rates of change at known sites.
   Promlk  
 Same as Proml but assumes a molecular clock.  The use of the
   two programs together permits a likelihood ratio test of the
   molecular clock hypothesis to be made.
   Dnadist  
  Computes four different distances between species from nucleic acid
   sequences.  The distances can then be used in the distance matrix programs.
   The distances are the Jukes-Cantor formula, one based on Kimura's 2-
   parameter method, the F84 model used in Dnaml, and the LogDet distance.
   The distances can also be corrected for gamma-distributed and
   gamma-plus-invariant-sites-distributed rates of change in different sites.
   Rates of evolution can vary among sites in a prespecified way, and also
   according to a Hidden Markov model.  The program can also make a table of
   percentage similarity among sequences.
   Protdist  
 Computes a distance measure for protein sequences, using
   maximum likelihood estimates based on the Dayhoff PAM matrix,
   the JTT matrix model, the PBM model, Kimura's 1983 approximation to these,
   or a model based on the genetic code plus a constraint on changing to a
   different category of amino acid.  The distances can also be corrected for
   gamma-distributed and gamma-plus-invariant-sites-distributed rates of change
   in different sites.  Rates of evolution can vary among sites in a
   prespecified way, and also according to a Hidden Markov model.  The program
   can also make a table of percentage similarity among sequences.  The
   distances can be used in the distance matrix programs.
   Restdist  
 Distances calculated from restriction sites data or
   restriction fragments data.  The restriction sites option is the one to
   use to also make distances for RAPDs or AFLPs.
   Restml  
 Estimation of phylogenies by maximum likelihood using
   restriction sites data (not restriction fragments but presence/absence of
   individual sites).  It employs the Jukes-Cantor symmetrical model of
   nucleotide change, which does not allow for differences of rate between
   transitions and transversions.  This program is  very  slow.
   Seqboot  
 Reads in a data set, and produces multiple data sets from
   it by bootstrap resampling.  Since most programs in the current version of
   the package allow processing of multiple data sets, this can be used
   together with the consensus tree program Consense to do bootstrap (or
   delete-half-jackknife) analyses with most of the methods in this package.
   This program also allows the Archie/Faith technique of permutation of
   species within characters.  It can also rewrite a data set to convert
   it from between the PHYLIP Interleaved and Sequential forms, and into
   a preliminary version of a new XML sequence alignment format
   which is under development and which is described in the
    Seqboot documentation web page .
   Fitch  
 Estimates phylogenies from distance matrix data under the
   "additive tree model" according to which the distances are expected to
   equal the sums of branch lengths between the species.  Uses the
   Fitch-Margoliash criterion and some related least squares criteria, or
   the Minimum Evolution distance matrix method.  Does
   not assume an evolutionary clock.  This program will be useful with
   distances computed from molecular sequences, restriction sites or fragments
   distances, with DNA hybridization measurements, and with genetic distances 
   computed from gene frequencies.
   Kitsch  
 Estimates phylogenies from distance matrix data under the 
   "ultrametric" model which is the same as the additive tree model except 
   that an evolutionary clock is assumed.  The Fitch-Margoliash criterion and 
   other least squares criteria, or the Minimum Evolution criterion are
   possible.  This program will be useful with 
   distances computed from molecular sequences, restriction sites or
   fragments distances, with distances from DNA hybridization measurements, 
   and with genetic distances computed from gene frequencies. 
   Neighbor  
 An implementation by Mary Kuhner and John Yamato of Saitou and
   Nei's "Neighbor Joining Method," and of the UPGMA (Average Linkage
   clustering) method.  Neighbor Joining is a distance matrix method producing
   an unrooted tree without the assumption of a clock.  UPGMA does assume a
   clock.  The branch lengths are not optimized by the least squares criterion
   but the methods are very fast and thus can handle much larger data sets.
   Contml  
 Estimates phylogenies from gene frequency data by maximum
   likelihood under a model in which all divergence is due to genetic drift in
   the absence of new mutations.  Does not assume a molecular clock.  An 
   alternative method of analyzing this data is to compute Nei's genetic 
   distance and use one of the distance matrix programs.
   This program can also do maximum likelihood analysis of continuous
   characters that evolve by a Brownian Motion model, but it assumes that
   the characters evolve at equal rates and in an uncorrelated fashion, so
   that it does not take into account the usual correlations of characters.
   Gendist  
 Computes one of three different genetic distance formulas
   from gene frequency data.  The formulas are Nei's genetic distance, the
   Cavalli-Sforza chord measure, and the genetic distance of Reynolds et. al.
   The former is appropriate for data in which new mutations occur in an
   infinite isoalleles neutral mutation model, the latter two for a model
   without mutation and with pure genetic drift.  The distances are written to
   a file in a format appropriate for input to the distance matrix programs.
   Contrast  
 Reads a tree from a tree file, and a data set with continuous
   characters data, and produces the independent contrasts for those
   characters, for use in any multivariate statistics package.  Will also
   produce covariances, regressions and correlations between characters for
   those contrasts.  Can also correct for within-species sampling variation
   when individual phenotypes are available within a population.
   Pars  
 Multistate discrete-characters parsimony method.   Up to 8 states
  (as well as " ? ") are allowed.  Cannot do Camin-Sokal or Dollo Parsimony.
  Can cope with multifurcations, reconstruct ancestral states, use character
  weights, and infer branch lengths.
   Mix  
 Estimates phylogenies by some parsimony methods for discrete
   character data with two states (0 and 1).  Allows use of the
   Wagner parsimony method, the Camin-Sokal parsimony method, or arbitrary
   mixtures of these.  Also reconstructs ancestral states and allows weighting
   of characters (does not infer branch lengths).
   Move  
 Interactive construction of phylogenies from discrete character
   data with two states (0 and 1).  Evaluates parsimony and compatibility
   criteria for those phylogenies and displays reconstructed states throughout
   the tree.  This can be used to find parsimony or compatibility estimates by 
   hand. 
   Penny  
 Finds all most parsimonious phylogenies for discrete-character
   data with two states, for the Wagner, Camin-Sokal, and mixed parsimony
   criteria using the branch-and-bound method of exact search.  May be
   impractical (depending on the data) for more than 10-11 species. 
   Dollop  
 Estimates phylogenies by the Dollo or polymorphism parsimony
   criteria for discrete character data with two states (0 and 1).  Also
   reconstructs ancestral states and allows weighting of characters.  Dollo
   parsimony is particularly appropriate for restriction sites data; with
   ancestor states specified as unknown it may be appropriate for restriction
   fragments data.
   Dolmove  
 Interactive construction of phylogenies from discrete
   character data with two states (0 and 1) using the Dollo or polymorphism
   parsimony criteria.  Evaluates parsimony and compatibility criteria for
   those phylogenies and displays reconstructed states throughout the tree. 
   This can be used to find parsimony or compatibility estimates by hand. 
   Dolpenny  
 Finds all most parsimonious phylogenies for
    discrete-character data with two states, for the Dollo or polymorphism
   parsimony criteria using the branch-and-bound method of exact search.  May
   be impractical (depending on the data) for more than 10-11 species. 
   Clique  
 Finds the largest clique of mutually compatible characters, and
   the phylogeny which they recommend, for discrete character data with two 
   states.  The largest clique (or all cliques within a given size range of 
   the largest one) are found by a very fast branch and bound search method.  
   The method does not allow for missing data.  For such cases the  T 
   (Threshold) option of Pars or Mix may be a useful alternative. 
   Compatibility methods are particular useful when some characters are of
   poor quality and the rest of good quality, but when it is not known in
   advance which ones are which.
   Factor  
 Takes discrete multistate data with character state trees and 
   produces the corresponding data set with two states (0 and 1).  Written by 
   Christopher Meacham.  This program was formerly used to accomodate
   multistate characters in Mix, but this is less necessary now that PARS is
   available.
   Drawgram  
 Plots rooted phylogenies, cladograms, circular trees and phenograms in a
   wide variety of user-controllable formats.  The program is interactive and
   allows previewing of the tree on PC, Macintosh, or X Windows screens, 
   or on Tektronix or Digital graphics terminals.  Final output can be 
   to a file formatted for one of the drawing programs, for a ray-tracing or
   VRML browser, or one at can be sent to a laser printer (such as Postscript
   or PCL-compatible printers), on graphics screens or terminals, on pen
   plotters or on dot matrix printers capable of graphics.
   Drawtree  
 Similar to Drawgram but plots unrooted phylogenies.
   Treedist  
  Computes the Branch Score distance between trees, which allows for
differences in tree topology and which also makes use of branch lengths.  Also
computes the Robinson-Foulds symmetric difference distance between trees, which
allows for differences in tree topology but does not use branch lengths.
   Consense  
 Computes consensus trees by the majority-rule consensus tree 
   method, which also allows one to easily find the strict consensus tree.  
   Is not able to compute the Adams consensus tree.  Trees are input in a tree
   file in standard nested-parenthesis notation, which is produced by many of
   the tree estimation programs in the package.  This program can be used as
   the final step in doing bootstrap analyses for many of the methods in the
   package.
   Retree  
 Reads in a tree (with branch lengths if necessary) and allows
   you to reroot the tree, to flip branches, to change species names and
   branch lengths, and then write the result out.  Can be used to convert
   between rooted and unrooted trees, and to write the tree into a
   preliminary version of a new XML tree file format which is under
   development and which is described in the
    Retree documentation web page .
 
 
    
 
 Running the Programs  
 
This section assumes that you have obtained PHYLIP as compiled executables
(for Windows, Mac OS X, Mac OS 9 or Linux), or have obtained the source code
and compiled it yourself (for Linux, Unix, or OpenVMS).   For machines for
which compiled executables are available, there will usually be no need for
you to have a compiler or compile the programs yourself.  This section
describes how to run the programs.  Later in this document we will
discuss how to download and install PHYLIP (in case you are
reading this without yet having done that).  Normally you will only read
your copy of this document after downloading and installing PHYLIP.
 
 A word about input files. 
 
For all of these types of machines, it is
important to have the input files for the programs (typically data files)
prepared in advance.  They can be prepared in any editor, but it is important
that they be saved in Text Only ("flat ASCII") format, not in the format that
word processors such as Microsoft Word want to write.  It is up to you to read
the PHYLIP documentation files which describe the files formats that are
needed.  There is a partial description in the next section of this document.
The input files can also be obtained by running a program that
produces output files in PHYLIP format (some of these programs do, and so do
programs by others such as sequence alignment programs such as ClustalW and
sequence format conversion programs such as Readseq).   There is  not  any
input file editor available in any program in PHYLIP (you should  not 
simply start running one of the programs and then expect to click a mouse
somewhere to start creating a data file).  
 
When they start running, the programs look first for input files with
particular names (such as  infile ,  treefile ,   intree , or  fontfile ).
Exactly which file names they look for varies a bit from program to program,
and you should read the documentation file for the particular program to
find out.  If you have files with those names the programs will use them
and not ask you for the file name.  If they do not find files of those
names, the programs will say that they cannot find a file of that name, and
ask you to type in the file name.
For example, if DnaML looks
for the file  infile  and does not find one of that name,
it prints the message:
 
   
 dnaml: can't find input file "infile" 
Please enter a new file name> 
   
  This does not mean that an error
has occurred.   All you need to do is to type in the name of the file.
 
The program looks for the input files in the same folder that the
program is in (a folder is the same thing as a "directory").  In Windows, 
Mac OS X, Linux, or Unix, if you are asked for the
file name you can type in the path to the file, as part of the name (thus,
if the file is in the folder containing the current folder, you can type in
a file name such as  ../myfile.dna ).   If you do not know what a
"folder" is, or what "above" means, then you are a member of the new
generation who just clicks the mouse and assumes that a list of file names
will magically appear.  (Typically members of this generation have no idea
where the files are on their system, and accumulate enormous amounts of
unnecessary clutter in their file systems.)  In this case you should ask
someone to explain folders to you.
 
 Running the programs on a Windows machine. 
 
Double-click on the icon for
the program.  A window should open with a menu in it.  Further dialog with the
program occurs
by typing on the keyboard in response to what you see in the window.  The
programs can be interrupted either by typing Control-C (which means to
press down on the  Ctrl  key while typing the letter  C ), or by using
the mouse to open the  File  menu in the upper-left corner of the program's
window area and then select  Quit .  Other than this, most PHYLIP programs
make no use of the mouse.  The tree-drawing programs Drawtree and Drawgram
do allow use of the mouse to select some options.
 
The programs open a window for their menus.  This window may be too small for
your tastes. They can be resized by tugging on the lower-right corner of the
window.  In addition, the font may be too small.  On most versions of Windows,
you can click on the small icon
symbol at the upper-left corner of the window, and choose the  Properties  menu choice there.  One of its options is to change the font and size of
the print.  I prefer large font sizes such as 16x12.  Some versions of Windows
wuch as Windows XP also ask you if you want to apply this choice to all
such windows, not just to the current one.
 
The programs can also be run in a Command window under Windows, in much the
same way as they were under the MSDOS operating system, which is what the
Command window emulates.  Type the name of the program
in lower-case letters (such as  dnaml ).  To interrupt the program while
it is running, type Control-C (which means to press down on the  Ctrl  key
while typing the letter  C ).  
 
 Running the programs on a Macintosh with Mac OS X 
 
We have provided a Mac OS X version of the executables, in the form of
"univeral binaries" that should run either on PowerMac or Intel iMac
systems.  The programs can be
run by clicking on their icons.  They open a Terminal window, and the
menu appears in it.  Note that after the program is finished, the Terminal
window remains open, and operations can be done in it.   You will have to
close the window yourself if you don't want it.  The programs can be
interrupted by typing control-C (press down the "control" key in the
lower-left corner of the keyboard and type "c").
 
It is also possible to run the executables from within a
Terminal window by typing the program name, but this is harder.
You do need to have links made in the  exe  folder to the
programs.  This can be done the first time you need them, by entering
the  exe  folder and opening a Terminal window, and then typing
 source linkmac .  This creates the proper links, and thereafter
you do not need to do this again.  The programs can be run by typing
their names in a Terminal window whose current working directory is
 exe   The programs work well this way,
though the programs Drawgram and Drawtree may be slow to
open and close plotting windows.
The programs can be interrupted by typing control-C or by
closing the Terminal window by using the red button in the upper-left
corner of the window.
 
On Mac OS X systems the Mac OS 8 or 9 executables can also be run
using the Classic environment
included with it which allows executables for earlier versions of
Mac OS to run.
 
If your Mac OS X system has the X windows windowing system installed,
you could also make executables that will work with that.  See below, under
"Compiling with GCC on Mac OS X with X Windows" for instructions on how to do
this.
 
One problem we have often encountered using Mac OS X is that it is possible for
data files to have the wrong kind of characters at the ends of their lines.
They may have carriage-return (ASCII/ISO 13 or control-M) characters at the
ends of their lines when they should instead have the Unix newline character
(ASCII/ISO 10 or control-J) there.  This can happen with files transferred
from other operating systems or files produced in some word processors.
It results in segmentation-fault or memory errors.  If you encounter these,
check this possibility carefully.
 
 Running the programs on a Macintosh with Mac OS 8 or 9 
 
Double-click on the icon for
the program.  A window should open.  Further dialog with the program occurs
by typing on the keyboard in response to what you see in the window.  The
programs can be interrupted by using
the mouse to open the  File  menu in the upper-left corner of the program's
window area and then select  Quit .  Alternatively, you can use the
Command-Q key combination.
 
When you use Quit, the program will ask you whether you want to save
a file whose name is the program name (often followed by  .out  -- for
example, if you are using Dnaml it will ask you if you want to save file
 Dnaml.out .  This file is simply a record of everything that
displayed on the program window, and you usually will not want to save it.
Pressing the  Enter  key or selecting the Do Not Save button with
the mouse will keep this from being saved.
 
If you encounter memory limitations on a Mac OS 8 or 9 Macintosh,
and determine that
this is not due to a problem with the format of the input file, as it
often will be, you may be able to solve it by raising the limits of the
stack and heap sizes of the program.  To do this click on the program
and then select  Get Info  from the Finder  File  menu.
This will open a window which can be made to show the memory limits
of the program.  These can be changed by selecting them and typing in
larger numbers.  This may relieve nagging memory problems.  If it does
not, consult your local documentation and suspect problems with your
input file format.
 
 Running the programs on a Unix or Linux system. 
 
Type the name of the program
in lower-case letters (such as  dnaml ).  To interrupt the program while
it is running, type Control-C (which means to press down on the  Ctrl  key
while typing the letter  C ).
 
On some systems you may need to type  ./  before the program name,
so that in the above case it would be  ./dnaml .  This is mostly
needed if the users PATH does not include their current directory, something
which is often done as a security precaution.
 
 Running the programs in background or under control of a command file 
 
In running the programs, you may sometimes want to put them in background
so you can proceed with other work.  On systems with a windowing environment
they can be put in their own window, and commands like the Unix and Linux
 nice  command used to make
them have lower priority so that they do not interfere with interactive
applications in other windows.  This part of the discussion will
assume either a Windows system or a Unix or Linux system.  I will
note when the commands work on one of these systems but not the other.
Mac OS X is actually Unix (surprise! surprise!) and you can
run PHYLIP programs in background on any Mac OS X system by simply following
the instructions for Unix, using a terminal window to do so if necessary.
Running jobs in background on Mac OS 8 or 9 systems is an arcane art into whose
mysteries I have not been initiated (or perhaps no one has been initiated).
 
If there is no windowing
environment, on a Unix or Linux system you will want to use an
ampersand ( & ) after the command file name when invoking it to put the
job in the background.  You will have to put all the responses to the
interactive menu of the program into a file and tell the background job
to take its input from that file.
 
On Windows systems there is no  &  or  nice  command
but input and output redirection and command files work fine in a Commmand
window.   A command file can either be invoked by clicking on its icon or
by typing its name from a Command window.  The a file of commands must have
a name ending in  .bat , such as  foofile.bat .  You can 
run the batch file from a Command window by typing its name (such as
 foofile ) without the  .bat .
 
For example: suppose you want to run Dnapars in a background, taking its
input data from a file called  sequences.dat , putting its interactive
output to file called  screenout , and using a file called  input  as
the place to store the interactive input.  The file  input  need only
contain two lines:
 
   
 
sequences.dat
Y
 
   
 
which is what you would have typed to run the program interactively, in
response to the program's request for an input file name if it did not
find a file named  infile , in in response the the menu.
 
To run the program in background, in Unix or Linux you would simply give the command:
 
 dnapars < input > screenout &
 
 
These run the program with input responses coming from  input  and
interactive output being put into file  screenout .  The usual output
file and tree file will also be created by this run (keep that in mind
as if you run any other PHYLIP program from the same directory while
this one is running in background you may overwrite the output file from
one program with that from the other!).
 
If you wanted to give the program lower priority, so that it would
not interfere with other work, and you have Berkeley Unix type job control
facilities in your Unix or Linux (and you usually do), you can use the
 nice  command:
 
 nice +10 dnapars < input > screenout &
 
 
which lowers the priority of the run.  To also time the run and put the
timing at the end of  screenout , you can do this:
 
 nice +10 ( time dnapars < input ) >& screenout &
 
 
which I will not attempt to explain.
 
On Unix or Linux systems
you may also want to explore putting the interactive output into the
null file  /dev/null  so as to not be bothered with it (but then you
cannot look at it to see why something went wrong).  If you have problems
with creating output files that are too large, you may want to
explore carefully the turning off of options in the programs you run.
 
If you are doing several runs in one, as for example when you do a
bootstrap analysis using Seqboot, Dnapars (say), and Consense, you
can use an editor to create a "command file" with these commands:
 
   
 
seqboot   screenout
mv outfile infile
dnapars  > screenout
mv outtree intree
consense  > screenout
 
   
 
This is the Unix or Linux version -- in the Windows version, the renaming
of files and the appending of output to the file  screenout  is
handled differently.
 
On Unix or Linux the command file might be named something like
 foofile , and on Windows systems might be named  foofile.bat .
 
On Unix or Linux the command file must be given
execute permission by using the command   chmod +x foofile  followed
by the command  rehash .  The job that  foofile  describes
can be run in background on Unix or Linux by giving the command
 
 foofile & 
 
On Windows systems it can be run by
clicking on the icon of the command file.  Its icon will have a little gear
symbol.
 
Note that you must also have the interactive input
commands for Seqboot (including the random number seed), Dnapars, and
Consense in the separate files  input1 ,  input2 , and  input3 .
Note also that when PHYLIP programs attempt to open a new output file (such as
 outfile ,  outtree , or  plotfile , if they see
a file of that name already in existence they will ask you if you want to
overwrite it, and offer alternatives including writing to another file,
appending information to that file, or quitting the program without writing to
the file.  This means that in writing batch files it is important to know
whether there will be a prompt of this sort.  You must know in advance
whether the file will exist.  You may want to put in your batch file a
command that tests for the existence of a pre-existing output file and
if so, removes it.  You might even want to put in a command that creates a
file of that name, so that you can be sure it is there!  Either way,
you will then know whether to put into your file of keyboard responses the
proper response to the inquiry about overwriting that output file.
 
    
 
 Preparing Input Files  
 
The input files for PHYLIP programs must be prepared separately - there is
no data editor within PHYLIP.  You can use a word processor (or text
editor) to prepare them yourself, or you can use a program that produces
a PHYLIP-format output.  Sequence alignment programs such as ClustalW
commonly have an option to produce PHYLIP files as output, and some
other phylogeny programs, such as MacClade and TreeView, are capable of
producing a PHYLIP-format file.
 
The format of the input files is discussed below, and you should also
read the other PHYLIP documentation relevant to the particular type of
data that you are using, and the particular programs you want to run, as
there will be more details there.
 
It is very important that the input files be in "Text Only" or "flat
ASCII" format.  This means that they contain only printable ASCII/ISO
characters, and not any unprintable characters.  Many word processors such
as Microsoft Word save their files in a format that contains unprintable
characters, unless you tell them not to.  For Microsoft Word you can
select  Save As  from its  File  menu, and choose  Text Only 
as the file format.  This can also be done in WordPad utility in Windows .
Other word processors will have equivalent
options.  Text editors such as the  vi  and  emacs  editors on
Unix and Linux, Windows Notepad, the  SimpleText  editor in Mac OS, or the  pico 
editor that comes with the  pine 
mailer program, produce their files in Text Only format and should not
cause any trouble.
 
 Input and output files 
 
For most of the PHYLIP programs, information comes from a series of
input files, and ends up in a series of output files:
 
 
 
  
 
                   -------------------
                  |                   |
infile ---------> |                   |
                  |                   |
intree ---------> |                   | -----------> outfile
                  |                   |
weights --------> |      program      | -----------> outtree
                  |                   |
categories -----> |                   | -----------> plotfile
                  |                   |
fontfile -------> |                   |
                  |                   |
                   -------------------
 
  
 
   

 
The programs interact with the user by presenting a menu.  Aside from the
user's choices from the menu, they read
all other input from files.  These files have default names.  The program
will try to find a file of that name - if it does not, it will ask the
user to supply the name of that file.
Input data such as DNA sequences
comes from a file whose default name is  infile .  If the user
supplies a tree, this is in a file whose default name is  intree .
Values of weights for the characters are in  weights , and the
tree plotting program need some digitized fonts which are supplied in
 fontfile  (all these are default names).
 
For example, if Dnaml looks
for the file  infile  and does not find one of that name,
it prints the message:
 
   
 dnaml: can't find input file "infile" 
Please enter a new file name> 
   
 
This simply means that it wants you to type in the name of the
input file.
 
 Data file format 
 
I have tried to adhere to a rather stereotyped input and output
format.  For the parsimony, compatibility and maximum likelihood programs, 
excluding the distance matrix methods, the simplest version of the input
data file looks something like this:
 
   
 
   6   13
Archaeopt CGATGCTTAC CGC
HesperorniCGTTACTCGT TGT
BaluchitheTAATGTTAAT TGT
B. virginiTAATGTTCGT TGT
BrontosaurCAAAACCCAT CAT
B.subtilisGGCAGCCAAT CAC
   
 
 
The first line of the input file contains the number of species and the
number of characters (in this case sites).  These are in free format, separated
by blanks.  The information for each species follows, starting with a
ten-character species name (which can include blanks and some punctuation
marks), and continuing with the characters for that species.  The name should
be on the same line as the first character of the data for that species.
(I will use the term "species" for the tips of the trees, recognizing
that in some cases these will actually be populations or individual gene
sequences).
 
The name should be ten characters in length, filled out to the full
ten characters by blanks if shorter.  Any printable ASCII/ISO character is
allowed in the name, except for parentheses (" ( " and " ) "), square
brackets (" [ " and " ] "), colon (" : "), semicolon (" ; ") and comma (" , ").
If you forget to extend the names to ten characters in length by blanks,
the program will get out of synchronization with the contents of the data
file, and an error message will result.
 
Note that Tab characters count as only one character in the species names.
Their inclusion can cause trouble.  The name will appear to you to be filled
out to a full ten characters, but it may be shorter than that to the program.
If you make the data file in a word processor such as Word, you would be
well-advised to make sure the names contain no Tab characters.  You can check
for their presence by advancing through the names with your cursor keys, and
looking for sudden jumps of two or more characters.  It is best to fill the
names out with blanks, not Tabs.
 
In the
discrete-character programs, DNA sequence programs and protein sequence
programs the characters are each a
single letter or digit, sometimes separated by blanks.  In
the continuous-characters programs they are real numbers with decimal points,
separated by blanks:
 
 Latimeria  2.03  3.457  100.2  0.0  -3.7 
 
The conventions about continuing the data beyond one line per species are
different between the molecular sequence programs and the others.  The 
molecular sequence programs can take the data in "aligned" or "interleaved"
format, in which we first have some lines giving the first part of each of the
sequences, then some
lines giving the next part of each, and so on.  Thus the sequences might
look like this:
 
   
 
    6   39
Archaeopt CGATGCTTAC CGCCGATGCT
HesperorniCGTTACTCGT TGTCGTTACT
BaluchitheTAATGTTAAT TGTTAATGTT
B. virginiTAATGTTCGT TGTTAATGTT
BrontosaurCAAAACCCAT CATCAAAACC
B.subtilisGGCAGCCAAT CACGGCAGCC

TACCGCCGAT GCTTACCGC
CGTTGTCGTT ACTCGTTGT
AATTGTTAAT GTTAATTGT
CGTTGTTAAT GTTCGTTGT
CATCATCAAA ACCCATCAT
AATCACGGCA GCCAATCAC
 
   
 
Note that in these sequences we have a blank every
ten sites to make them easier to read: any such blanks are allowed.  The blank
line which separates the two groups of lines (the ones
containing sites 1-20 and ones containing sites 21-39) may or may not
be present, but if it is, it should be a line of zero length and not contain
any extra blank
characters (this is because of a limitation of the current versions
of the programs).  It is important that the number of sites in each
group be the same for all species (i.e., it will not be possible to run
the programs successfully if the first species line contains 20 bases, but
the first line for the second species contains 21 bases).
 
Alternatively, an option can be selected in the menu to take the data in
"sequential" format, with all of the data for the first species,
then all of the characters for the next species, and so on.  This is also
the way that the discrete characters programs and the gene frequencies
and quantitative characters programs want to read the data.  They do not
allow the interleaved format.
 
In the sequential format, the character data can run on to a new line at any
time (except in the middle of a species name or, in the case of continuous
character and distance matrix programs where you cannot go to a new line in
the middle of a real number).  Thus it is legal to have:
 
 Archaeopt 001100
 
1101
 
 
 
or even:
 
 Archaeopt 
 
0011001101
 
 

 
though note that the  full  ten characters of the species name  must 
then be present: in the above case there must be a blank after the "t".  In all
cases it is possible to put internal blanks between any of the character
values, so that
 
 Archaeopt 0011001101 0111011100
 
 
is allowed.
 
Note that you can convert molecular sequence data between the interleaved
and the sequential data formats by using the Rewrite option of the J
menu item in Seqboot.
 
If you make an error in the format of the input file, the programs can
sometimes detect that
they have been fed an illegal character or illegal numerical value and issue
an error message such as  BAD CHARACTER STATE: , often printing out the 
bad value, and sometimes the number of the species and character in which it
occurred.  The program will then stop shortly after.  One of the things which
can lead to a bad value is the omission of something earlier in the file, or
the insertion of something superfluous, which cause the reading of the file to
get out of synchronization.  The program then starts reading things it
didn't expect, and concludes that they are in error.  So if you see this error
message, you may also want
to look for the earlier problem that may have led to the program becoming
confused about what it is reading.
 
Some options are described below, but you should also read the documentation
for the groups of the programs and for the individual programs.
 
 
    
 The Menu 
 
The menu is straightforward.  It typically looks like this (this one is for
Dnapars):
 
   
 
DNA parsimony algorithm, version 3.6

Setting for this run:
  U                 Search for best tree?  Yes
  S                        Search option?  More thorough search
  V              Number of trees to save?  10000
  J   Randomize input order of sequences?  No. Use input order
  O                        Outgroup root?  No, use as outgroup species  1
  T              Use Threshold parsimony?  No, use ordinary parsimony
  N           Use Transversion parsimony?  No, count all steps
  W                       Sites weighted?  No
  M           Analyze multiple data sets?  No
  I          Input sequences interleaved?  Yes
  0   Terminal type (IBM PC, ANSI, none)?  ANSI
  1    Print out the data at start of run  No
  2  Print indications of progress of run  Yes
  3                        Print out tree  Yes
  4          Print out steps in each site  No
  5  Print sequences at all nodes of tree  No
  6       Write out trees onto tree file?  Yes

  Y to accept these or type the letter for one to change
 
   
 
If you want to accept the default settings (they are shown in the above case)
you can simply type  Y  followed by pressing on the  Enter  key.
If you want to change any of the options, you should type the letter
shown to the left of its entry in the menu.  For example, to set a threshold
type  T .  Lower-case letters will also work.  For many of the options
the program will ask for supplementary information, such as the value of
the threshold. 
 
Note the  Terminal type  entry, which you will find on all menus.  It
allows you to specify which type of terminal your screen is.  The options
are an IBM PC screen, an ANSI standard terminal, or  none .
Choosing zero ( 0 ) toggles
among these three options in cyclical order, changing each time the  0 
option is chosen.  If one of them is right for your terminal the screen will be
cleared before the menu is displayed.  If none works, the  none  option
should probably be chosen.  The programs should start with a terminal option
appropriate for your computer, but if they do not, you can change the
terminal type manually.  This is particularly important in program Retree
where a tree is displayed on the screen - if the terminal type is set to the
wrong value, the tree can look very strange.
 
The other numbered options control which information the program will
display on your screen or on the output files.  The option to  Print
indications of progress of run  will show information such as the names of
the species as they are successively added to the tree, and the
progress of rearrangements.  You will usually want to see these as
reassurance that the program is running and to help you estimate how long
it will take.  But if you are running the program "in background" as can be
done on multitasking and multiuser systems, and do not have the
program running in its own window, you may want to turn this option off so
that it does not disturb your use of the computer while the program is
running.  Note also menu option 3, "Print out tree".  This can be useful
when you are running many data sets, and will be using the resulting trees
from the output tree file.  It may be helpful to turn off the printing out
of the trees in that case, particularly if those files would be too big.
 
    
 The Output File 
 
 
Most of the programs write their output onto a file called (usually)  outfile , and a representation of the trees found onto a file called
 outtree .
 
The exact contents of the output file vary from program to program and also
depend on which menu options you have selected.  For many programs, if you
select all possible output information, the output will consist of
(1) the name of the program and its
version number, (2) some of the input information printed out, and (3) a series of
phylogenies, some with associated information indicating how much change
there was in each character or on each part of the tree.  A typical rooted tree
looks like this:
 
   
 
                                     +-------------------Gibbon
        +----------------------------2
        !                            !      +------------------Orang
        !                            +------4
        !                                   !  +---------Gorilla
  +-----3                                   +--6
  !     !                                      !    +---------Chimp
  !     !                                      +----5
--1     !                                           +-----Human
  !     !
  !     +-----------------------------------------------Mouse
  !
  +------------------------------------------------Bovine
 
   
 
The interpretation of the tree is fairly straightforward: it "grows"
from left to right.  The numbers at the forks are arbitrary and are used (if
present) merely to identify the forks.  For many of the programs the tree 
produced is unrooted.   Rooted and unrooted trees are printed in nearly the
same form, but the unrooted ones are accompanied by the
warning message:
 
    remember: this is an unrooted tree!
 
 
to indicate that this is an unrooted tree and to warn against
taking the position of its root too seriously.  Mathematicians still call
an unrooted tree a tree, though some systematists unfortunately use the term
"network" for an unrooted tree.  This conflicts with standard mathematical
usage, which reserves the name "network" for a completely different kind of
graph).  The root of this tree could be anywhere, say on the line leading
immediately to  Mouse .  As an exercise,
see if you can tell whether the following tree is or is not a different
one from the above:
 
   
 
             +-----------------------------------------------Mouse
             !
   +---------4                                   +------------------Orang
   !         !                            +------3
   !         !                            !      !       +---------Chimp
---6         +----------------------------1      !  +----2
   !                                      !      +--5    +-----Human
   !                                      !         !
   !                                      !         +---------Gorilla
   !                                      !
   !                                      +-------------------Gibbon
   !
   +-------------------------------------------Bovine

   remember: this is an unrooted tree!
 
   
 
(it is  not  different).  It is  important  also to realize that the
lengths of the segments of the printed tree may not be significant: some
may actually represent branches of zero length, in the sense that there is no
evidence that 
those branches are nonzero in length.  Some of the diagrams of trees attempt
to print branches approximately proportional to estimated
branch lengths, while in others the lengths are purely conventional and
are presented just to make the topology visible.  You will have to look closely 
at the documentation that accompanies each program to see what it presents
and what is known about the lengths of the branches on the tree.  The above
tree attempts to represent branch lengths approximately in the diagram.  But
even in those cases, some of the smaller branches are likely to be
artificially lengthened to make the tree topology clearer.  Here is what
a tree from Dnapars looks like, when no attempt is made to make the
lengths of branches in the diagram proportional to estimated branch
lengths:
 
   
 
                 +--Human
              +--5
           +--4  +--Chimp
           !  !
        +--3  +-----Gorilla
        !  !
     +--2  +--------Orang
     !  !
  +--1  +-----------Gibbon
  !  !
--6  +--------------Mouse
  !
  +-----------------Bovine

  remember: this is an unrooted tree!
 
   
 
When a tree has branch lengths, it will be accompanied by a table showing
for each branch the numbers (or names) of the nodes at each end of the
branch, and the length of that branch.  For the first tree shown above,
the corresponding table is:
 
   
 
 Between        And            Length      Approx. Confidence Limits
 -------        ---            ------      ------- ---------- ------

    1          Bovine            0.90216     (  0.50346,     1.30086) **
    1          Mouse             0.79240     (  0.42191,     1.16297) **
    1             2              0.48553     (  0.16602,     0.80496) **
    2             3              0.12113     (     zero,     0.24676) *
    3             4              0.04895     (     zero,     0.12668)
    4             5              0.07459     (  0.00735,     0.14180) **
    5          Human             0.10563     (  0.04234,     0.16889) **
    5          Chimp             0.17158     (  0.09765,     0.24553) **
    4          Gorilla           0.15266     (  0.07468,     0.23069) **
    3          Orang             0.30368     (  0.18735,     0.41999) **
    2          Gibbon            0.33636     (  0.19264,     0.48009) **

      *  = significantly positive, P  
   
 
Ignoring the asterisks and the approximate confidence limits, which will be
described in the documentation file for Dnaml, we can see that the table
gives a more precise idea of what the lengths of all the branches are.
Similar tables exist in distance matrix and likelihood programs, as well
as in the parsimony programs Dnapars and Pars.
 
Some of the parsimony programs in the package can print out a table
of the number of steps that different characters (or sites) require on
the tree.  This table may not be obvious at first.  A typical example looks like
this:
 
   
 
 steps in each site:
         0   1   2   3   4   5   6   7   8   9
     *-----------------------------------------
    0!       2   2   2   2   1   1   2   2   1
   10!   1   2   3   1   1   1   1   1   1   2
   20!   1   2   2   1   2   2   1   1   1   2
   30!   1   2   1   1   1   2   1   3   1   1
   40!   1
 
   
 
The numbers across the top and down the side indicate which site
is being referred to.  Thus site 23 is column "3" of row "20"
and has 1 step in this case.
 
There are many other kinds of information that can appear in the
output file,  They vary from program to program, and we leave their
description to the documentation files for the specific programs.
 
    
 The Tree File 
 
In output from most programs,
a representation of the tree is also written into the tree file
 outtree .  The tree is specified by nested pairs
of parentheses, enclosing
names and separated by commas.  We will describe how this works
below.  If there are any blanks in the names,
these must be replaced by the underscore character " _ ".  Trailing blanks
in the name may be omitted.  The pattern of the parentheses indicates
the pattern of the tree by having each pair of parentheses enclose all
the members of a monophyletic group.  The tree file could look like this:
 
 ((Mouse,Bovine),(Gibbon,(Orang,(Gorilla,(Chimp,Human)))));
 
 
In this tree the first fork separates the lineage leading to
 Mouse  and  Bovine  from the lineage leading to the rest.  Within the 
latter group there is a fork separating  Gibbon  from the rest, and so on.
The entire tree is enclosed in an outermost pair of parentheses.  The tree ends
with a semicolon.  In some programs such as Dnaml, Fitch, and Contml,
the tree will be unrooted.  An unrooted tree should have its 
bottommost fork have a
three-way split, with three groups separated by two commas:
 
 (A,(B,(C,D)),(E,F));
 
 
Here the three groups at the bottom node are  A ,  (B,C,D) , and
 (E,F) .  The single three-way split corresponds to one of the interior
nodes of the unrooted tree (it can be any interior node of the tree).  The
remaining forks are encountered as you move out from that first node.
In newer programs, some are able to tolerate these other forks being
multifurcations (multi-way splits).
You should check the documentation files
for the particular programs you are using to see in which of these forms
you can expect the user tree to be in.  Note that many of the programs
that actually estimate an unrooted tree (such as Dnapars) produce trees in the
treefile in rooted form!  This is done for reasons of arbitrary internal bookkeeping.  The placement of the root is arbitrary.  We are working toward
having all programs be able to read all trees, whether rooted or unrooted,
multifurcating or bifurcating, and having them do the right thing with
them.  But this is a long-term goal and it is not yet achieved.
 
For programs that infer branch lengths, these are given in the trees in the
tree file as real numbers following a colon, and placed immediately
after the group descended from that branch.  Here is a typical tree
with branch lengths:
 
 ((cat:47.14069,(weasel:18.87953,((dog:25.46154,(raccoon:19.19959, 
bear:6.80041):0.84600):3.87382,(sea_lion:11.99700, 
seal:12.00300):7.52973):2.09461):20.59201):25.0,monkey:75.85931);
 
 
Note that the tree may continue to a new line at any time except in the
middle of a name or the middle of a branch length, although in trees
written to the tree file this will only be done after a comma.
 
These representations of trees are a subset of the standard adopted
on 24 June 1986 at the annual meetings of the Society for the Study of
Evolution by an informal committee (its final session in Newick's
lobster restaurant - hence its name, the Newick standard)
consisting of Wayne Maddison (author of MacClade), David Swofford (PAUP),
F. James Rohlf (NTSYS-PC), Chris Meacham (COMPROB and the original
PHYLIP tree drawing programs), James Archie,
William H.E. Day, and me.  This standard is a generalization of
PHYLIP's format, itself based on a well-known representation of trees in 
terms of parenthesis patterns which is due to the famous mathematician 
Arthur Cayley, and which has been around for over a century.  The
standard is now employed by most phylogeny computer programs but unfortunately
has yet to be decribed in a formal published description.  Other
descriptions by me and by Gary Olsen can be accessed using the Web at:
 
 
  
 http://evolution.gs.washington.edu/phylip/newicktree.html   
 
 
    
 The Options and How To Invoke Them 
 
Most of the programs allow various options that alter the amount of
information the program is provided or what is done with the
information.  Options are selected in the menu.
 
 Common options in the menu 
 
A number of the options from the menu, the  U  (User tree),  G  (Global),
 J  (Jumble),  O  (Outgroup),  W  (Weights),
 T  (Threshold),  M  (multiple data sets), and the tree output options, are used
so widely that it is best to discuss them in this document.
 
 The  U  (User tree) option.   This option toggles between the default
setting, which allows the program to search for the best tree, and the
User tree setting, which reads a tree or trees ("user trees") from the input
tree file and evaluates them.  The input tree file's
default name is  intree .  In many cases the programs will also
tolerate having the trees
be preceded by a line giving the number of trees:
 
   
 
((Alligator,Bear),((Cow,(Dog,Elephant)),Ferret));
((Alligator,Bear),(((Cow,Dog),Elephant),Ferret));
((Alligator,Bear),((Cow,Dog),(Elephant,Ferret)));
 
   
 
An initial line with the number of trees was formerly
required, but this now can be omitted.  Some programs require rooted
trees, some unrooted
trees, and some can handle multifurcating trees.  You should read
the documentation for the particular program to find out which it
requires.  Program Retree can be used to convert trees among
these forms (on saving a tree from Retree, you are asked whether
you want it to be rooted or unrooted).
 
In using the user tree option, check the pattern of parentheses
carefully.  The programs do not always detect
whether the tree makes sense, and if it does not there will probably be
a crash (hopefully, but not inevitably, with an error message indicating
the nature of the problem).  Trees written out by programs are
typically in the proper form.
 
 The  G  (Global) option.   In  the programs which construct trees (except for
Neighbor, the "...penny" programs and Clique, and of course 
the "...move" programs where you construct the trees yourself),
after all species have been added to the tree a rearrangements phase
ensues.  In most of these programs the rearrangements are automatically
global, which in this case means that subtrees will be removed from the tree
and put back on in all possible ways so as to have a better chance of
finding a better tree.  Since this can be time consuming (it roughly
triples the time taken for a run) it is left as an option in some of the
programs, specifically Contml, Fitch, and Dnaml.  In these programs
the G menu option toggles between the default of local rearrangement and
global rearrangement.  The rearrangements are explained more below.
 
 The  J  (Jumble) option.   In most of the tree construction programs
(except for the "...penny" programs and Clique), the exact 
details of the search of different trees depend on the order of input of
species.  In these programs  J  option enables you to tell the program to use
a random number 
generator to choose the input order of species.  This option is toggled on
and off by
selecting option  J  in the menu.  The program will then prompt you for
a "seed" for the random number generator.  The seed should be an integer
between 1 and 2 32 -3 (which is 4,294,967,293), and should be
of form 4 n +1,
which means that it must give a remainder of 1 when divided by 4.  This can be
judged by looking at the last two digits of the number (for example, in the
upper limit given above, the last two digits are 93, which is of form 4 n +1.  Each different seed 
leads to a different sequence of addition of species.  By simply changing the 
random number seed and re-running the programs one can look for other, and 
better trees.  If the seed entered is not odd, the program will not proceed,
but will prompt for another seed.
 
The Jumble option also causes the program to ask you how many times you
want to restart the process.  If you answer 10, the program will
try ten different orders of species in constructing the trees, and the
results printed out will reflect this entire search process (that is,
the best trees found among all 10 runs will be printed out, not the
best trees from each individual run).
 
Some people have asked what are good values of the random number seed.
The random number seed is used to start a process of choosing "random"
(actually pseudorandom) numbers, which behave as if they were
unpredictably randomly chosen between 0 and 2 32 -1 (which is
4,294,967,295).  You could put in the number 133 and find that the
next random number was 221,381,825.  As they are effectively
unpredictable, there is no such thing as a choice that is better than
any other, provided that the numbers are of the form 4 n +1.  However
if you re-use a random number seed, the sequence of random numbers
that result will be the same as before, resulting in exactly the same
series of choices, which may not be what you want.
 
 The  O  (Outgroup) option.   This specifies which species is to
have the root of the tree be on the line leading to it.  For example, if the
outgroup is a species "Mouse" then the root of the tree will be placed in the
middle of the branch which is connected to this species, with Mouse branching
off on one side of the root and the lineage leading to the rest of the tree
on the other.  This option is toggled on and off by choosing  O  in the 
menu (the alphabetic character  O , not the digit  0 ).  When it 
is on, the program will then prompt for the
number of the outgroup (the species being taken in the numerical order that
they occur in the input file).  Responding by typing  6  and then an
 Enter  character indicates that the sixth species in the data
(the 6th in the first set of data if there are multiple data sets)
is taken as the outgroup.  Outgroup-rooting will not be attempted if the
data have already established a root for the tree from some other 
consideration, and may not be if it is a user-defined tree,
despite your invoking the option.  Thus programs such as Dollop that
produce only rooted trees do not allow the Outgroup option.  It is also
not available in Kitsch, Dnamlk, or Clique.  When it is used, the tree as
printed out is still listed as being an
unrooted tree, though the outgroup is connected to the bottommost node
so that it is easy to visually convert the tree into rooted form.
 
 The  T  (Threshold) option.   This sets a threshold forn the
parsimony programs such that if the
number of steps counted in a character is higher than the threshold, it
will be taken to be the threshold value rather than the actual number of
steps.  The default is a threshold so high that it will never be
surpassed (in which case the steps whill simply be counted).  The  T 
menu option toggles on and off asking the user to
supply a threshold.  The use of thresholds to obtain methods intermediate
between parsimony and compatibility methods is described in my 1981b paper. 
When the T option is in force, the program
will prompt for the numerical threshold value.  This will be a positive
real number greater than 1.  In programs Mix, Move, Penny, Protpars,
Dnapars, Dnamove, and Dnapenny, do not use threshold values less
than or equal to 1.0, as they have no meaning and lead to a tree which
depends only on considerations such as the input order of species and not at 
all on the character state data!  In programs Dollop, Dolmove, and Dolpenny
the threshold should never be 0.0 or less, for the same
reason.  The  T  option is an 
important and underutilized one: it is, for example, the only way in this 
package (except for program Dnacomp) to do a compatibility analysis when there 
are missing data.   It is a method of de-weighting characters that evolve
rapidly.  I wish more people were aware of its properties.  
 
 The  M  (Multiple data sets) option.   In menu programs there is an
 M  menu
option which allows one to toggle on the multiple data sets option.  The
program will ask you how many data sets it should expect.  The data sets
have the same format as the first data set.  Here is a (very small) input file
with two five-species data sets:
 
   
 
      5    6
Alpha     CCACCA
Beta      CCAAAA
Gamma     CAACCA
Delta     AACAAC
Epsilon   AACCCA
5    6
Alpha     CACACA
Beta      CCAACC
Gamma     CAACAC
Delta     GCCTGG
Epsilon   TGCAAT
 
   
 
The main use of this option will be to allow all of the methods in these
programs to be bootstrapped.  Using the program Seqboot one can take any
DNA, protein, restriction sites, gene frequency or binary character data set and
make multiple data sets by bootstrapping.  Trees can be produced for all of
these using the  M  option.  They will be written on the tree output file if
that option is left in force.  Then the program Consense can be used with
that tree file as its input file.  The result is a majority rule consensus
tree which can be used to make confidence intervals.  The present version
of the package allows, with the use of Seqboot and Consense and the M option,
bootstrapping of many of the methods in the package.
 
Programs Dnaml, Dnapars and Pars can also take multiple weights
instead of multiple data sets.  They can then do bootstrapping by
reading in one data set, together with a file of weights that show how
the characters (or sites) are reweighted in each bootstrap sample.  Thus a
site that is omitted in a bootstrap sample has effectively been given
weight 0, while a site that has been duplicated has effectively been
given weight 2.  Seqboot has a menu selection to produce the file of
weights information automatically, instead of producing a file of
multiple data sets.  It can be renamed and used as the input weights file.
 
 The  W  (Weights) option .  This signals the program that, in
addition to the data set, you want to read in a series of weights that
tell how many times each character is to be counted.  If the weight
for a character is zero ( 0 ) then that character is in effect to
be omitted when the tree is evaluated.  If it is ( 1 ) the
character is to be counted once.  Some programs allow weights greater than
1 as well.  These have the effect that the character is counted as
if it were present that many times, so that a weight of 4 means that the
character is counted 4 times.
The values 0-9 give weights 0 through 9, and the
values A-Z give weights 10 through 35.  By use of the weights we can
give overwhelming weight to some characters, and drop others from the
analysis.  In the molecular sequence programs only two values of the
weights, 0 or 1 are allowed.
 
The weights are used to analyze subsets of the characters, and also can be
used for resampling of the data as in bootstrap and jackknife resampling.
For those programs that allow weights to be greater than 1, they can also
be used to emphasize information from some characters more strongly than
others.  Of course, you must have some rationale for doing this.
 
The weights are provided as a sequence of digits.  Thus they might be
 
 10011111100010100011110001100 
 
The weights are to be provided in an input file
whose default name is  weights .   The weights in it are
a simple string of digits.  Blanks in the weightfile are skipped over and
ignored, and the weights can continue to a new line.  In programs such as
Seqboot
that can also output a file of weights, the input weights have a default
file name of  inweights , and the output file name has a default
file name of  outweights . 
 
Weights can be used to analyze different subsets of characters (by weighting
the rest as zero).  Alternatively, in the discrete characters programs
they can be used to force a certain
group to appear on the phylogeny (in effect confining consideration to only
phylogenies containing that group).  This is done by adding an imaginary
character that has  1 's for the members of the group, and  0 's
for all the
other species.  That imaginary character is then given the highest weight
possible: the result will be that any phylogeny that does not contain that
group will be penalized by such a heavy amount that it will not (except in
the most unusual circumstances) be considered.  Of course, the new character
brings extra steps to the tree, but the number of these can be calculated
in advance and subtracted out of the total when reporting the results.  This 
use of weights is an important one, and one sadly ignored
by many users who could profit from it.  In the case of molecular sequences
we cannot use weights this way, so that to force a given group to appear we
have to add a large extra segment of sites to the molecule, with (say) A's
for that group and C's for every other species.
 
 The option to write out the trees into a tree file .  This specifies that you
want the program to write
out the tree not only on its usual output, but also onto a file in
nested-parenthesis notation (as described above).  This option is sufficiently
useful that it is turned on by default in all programs that allow it.  You
can optionally turn it off if you wish, by typing the appropriate number
from the menu (it varies from program to program).  This option is useful for
creating tree files that can be directly read into the programs, including
the consensus tree and tree distance programs, and the tree plotting programs.
 
The output tree file has a default name of  outtree .
 
 The ( 0 ) terminal type option  .  (This is the digit  0 , not
the alphabetic character  O ). The program will default to
one particular assumption about your terminal (ANSI in the case of Linux,
Unix, or Mac OS X, none in the case of Mac OS 9, and IBM PC in the case of
Windows). You can
alternatively select it to be either an IBM PC, or nothing.
This affects the ability of the programs to clear the screen when they
display their menus, and the graphics characters used to display trees
in the programs Dnamove, Move, Dolmove, and Retree.  In the case of Windows,
the screen will clear properly with either the IBM PC or the ANSI settings,
but the graphics characters needed by Move, Dnamove, Dolmove, or Retree
will display correctly only with the IBM PC setting.
 
    
 
 The Algorithm for Constructing Trees  
 
All of the programs except Factor, Dnadist, Gendist, Dnainvar, Seqboot,
Contrast, Retree, and the plotting and
consensus tree programs act to construct an estimate of a phylogeny.  Move, 
Dolmove, and Dnamove let you construct it yourself by hand.  All of 
the rest but Neighbor, the "...penny" programs and Clique make use of
a common approach involving additions and rearrangements.  They are
trying to minimize or maximize some quantity over the space of all
possible evolutionary trees.  Each program contains a part that, given
the topology of the tree, evaluates the quantity that is being minimized
or maximized.  The straightforward approach would be to evaluate all
possible tree topologies one after another and pick the one which,
according to the criterion being used, is best.  This would not be
possible for more than a small number of species, since the number of
possible tree topologies is enormous.  A review of the literature on the
counting of evolutionary trees will be found one of my papers
(Felsenstein, 1978a) and in my book (Felsenstein, 2004, chapter 3).
 
Since we cannot search all topologies, these programs are not
guaranteed to always find the best tree, although they seem to do quite
well in practice.  The strategy they employ is as follows: the species
are taken in the order in which they appear in the input file.  The
first two (in some programs the first three) are taken and a tree
constructed containing only those.  There is only one possible topology for
this tree.  Then the next species is taken, and we consider where it
might be added to the tree.  If the initial tree is (say) a rooted tree
with two species and we want the resulting three-species tree to be a
bifurcating tree, there are only three places where we could add the
third species.  Each of these is tried, and each time the resulting tree is
evaluated according to the criterion.  The best one is chosen to be the
basis for further operations.  Now we consider adding the fourth
species, again at each of the five possible places that would result in
a bifurcating tree.  Again, the best of these is accepted.  This is
usually known as the Sequential Addition strategy.
 
 Local rearrangements 
 
The process continues in this manner, with one important exception.  After 
each species is added, and before the next
is added, a number of rearrangements of the tree are tried, in an effort
to improve it.  The algorithms move through the tree, making all
possible local rearrangements of the tree.  A local rearrangement involves an
internal segment of the tree in the following manner.  Each internal
segment of the tree is of this form (where T1, T2, and T3 are subtrees
- parts of the tree that can contain further forks and tips):
 
 
            T1      T2       T3
             \      /        /
              \    /        /
               \  /        /
                \/        /
                 *       /
                  *     /
                   *   /
                    * /
                     *
                     !
                     !
 
 
the segment we are discussing being indicated by the asterisks.  A local
rearrangement consists of switching the subtrees T1 and T3 or T2 and T3,
so as to obtain one of the following:
 
 
          T3       T2      T1            T1       T3      T2
           \       /       /              \       /       /
            \     /       /                \     /       /
             \   /       /                  \   /       /
              \ /       /                    \ /       /
               \       /                      \       /
                \     /                        \     /
                 \   /                          \   /
                  \ /                            \ /
                   !                              !
                   !                              !
                   !                              !
 
 
Each time a local rearrangement is successful in finding a better tree,
the new arrangement is accepted.  The phase of local rearrangements does
not end until the program can traverse the entire tree, attempting local
rearrangements, without finding any that improve the tree.
 
This strategy of adding species and making local rearrangements will look
at about  (n-1)x(2n-3)  different topologies, though if
rearrangements are frequently successful the number may be larger.  I
have been describing the strategy when rooted trees are being
considered.  For unrooted trees there is a precisely similar strategy,
though the first tree constructed may be a three-species tree and the
rearrangements may not start until after the addition of the fifth
species.
 
These local rearrangements have come to be called Nearest Neighbor
Interchanges (NNIs) in the phylogeny literature.
 
Though we are not guaranteed to have found the best tree topology,
we are guaranteed that no nearby topology (i. e.  none accessible by a
single local rearrangement) is better.  In this sense we have reached a
local optimum of our criterion.  Note that the whole process is
dependent on the order in which the species are present in the input
file.  We can try to find a different and better solution by reordering
the species in the input file and running the program again (or, more
easily, by using the  J  option).  If none of
these attempts finds a better solution, then we have some indication
that we may have found the best topology, though we can never be certain
of this.
 
Note also that a new topology is never accepted unless it is better
than the previous one, so that the rearrangement process can never fall
into an endless loop.  This is also the way ties in our criterion are
resolved, namely by sticking with the tree found first.  However, the tree 
construction programs other than Clique, Contml, Fitch, 
and Dnaml do keep a record of all trees found that are tied with the best one 
found.  This gives you some immediate idea of which parts of the tree can be 
altered without affecting the quality of the result.
 

 Global rearrangements 
 
A feature of most of the programs, such as Protpars, Dnapars,
Dnacomp, Dnaml, Dnamlk, Restml, Kitsch, Fitch, Contml, Mix, and Dollop,
is "global" optimization of the tree.  In four of these (Contml,
Fitch, Dnaml and Dnamlk) this is an option,  G .  In the others it
automatically applies.  When 
it is present there is an additional stage to the search for the best tree.  
Each possible subtree is removed from the tree from the tree and added back in 
all possible places.  This process continues until all subtrees can be removed 
and added again without any improvement in the tree.  The purpose of this 
extra rearrangement is to make it less likely that one or more a species gets 
"stuck" in a suboptimal region of the space of all possible trees.  The use of 
global optimization results in approximately a tripling (3 x ) of the run-time, 
which is why I have left it as an option in some of the slower programs.
 
What PHYLIP calls "global" rearrangements are more properly called
SPR (subtree pruning and regrafting) by Swofford et. al. (1996) as distinct
from the NNI (nearest neighbor interchange) rearrangements that PHYLIP
also uses, and the TBR (tree bisection and reconnection) rearrangements
that it does not use.  My book (Felsenstein, 2004, chapter 4) contains
a review of work on these and other rearrangements and search methods.
 
The programs doing global optimization print out a dot " . " after each group is 
removed and re-added to the tree, to give the user some sign that the 
rearrangements are proceeding.  A new line of dots is started whenever a new 
round of global rearrangements is started following an improvement in the 
tree.  On the line before the dots are printed there is printed a bar of
the form "!---------------!" to show how many dots
to expect.  The dots will
not be printed out at a uniform rate, but the later dots, which represent
removal of larger groups from the tree and trying them consequently in fewer
places, will print out more quickly.  With some compilers each row of dots may
not be printed out until it is complete.
 
It should be noted that Penny, Dolpenny, Dnapenny and Clique use a more 
sophisticated strategy of "depth-first search" with a "branch and bound"
search method that guarantees that all
of the best trees will be found.  In the case 
of Penny, Dolpenny and Dnapenny there can be a considerable sacrifice of 
computer time if the number of species is greater than about ten: it is a 
matter for you to consider whether it is worth it for you to guarantee finding 
all the most parsimonious trees, and that depends on how much free computer 
time you have!  Clique finds all largest cliques, and does so without undue 
burning of computer time.   Although all of these problems that have been
investigated fall into the
category of "NP-hard" problems that in effect do not have a rapid solution,
the cases that cause this trouble for the largest-cliques algorithm in
Clique apparently are not biologically realistic and do not occur in actual
data.
 

 Multiple jumbles 
 
As just mentioned, for most of these programs the search depends on the order
in which the species are entered into the tree.  Using the  J  (Jumble)
option you can supply a random number seed which will allow the program to put
the species in in a random order.  Jumbling can be
done multiple times.  For example, if you tell the program to do it
10 times, it will go through the tree-building process 10 times, each with a
different random order of adding species.  It will keep a record of the trees
tied for best over the whole process.  In other words, it does not just
record the best trees from each of the 10 runs, but records the best ones
overall.  Of course this is slow, taking 10 times longer than a single run.
But it does give us a much greater chance of finding all of the most
parsimonious trees.  In the terminology of Maddison (1991) it
can find different "islands" of trees.  The present algorithms do not
guarantee us to find all trees in a given "island" from a single run, so
multiple runs also help explore those "islands" that are found.
 
 Saving multiple tied trees 
 
For the parsimony and compatibility programs, one can have a perfect tie
between two or more trees.  In these programs these trees are all
saved.  For the newer parsimony programs such as Dnapars and Pars,
global rearrangement is carried out on all of these tied trees.  This can
be turned off in the menu.
 
For trees with criteria which are real numbers, such as the distance
matrix programs Fitch and Kitsch, and the likelihood programs Dnaml,
Dnamlk, Contml, and Restml, it is difficult to get an exact tie between
trees.  Consequently these programs save only the single best tree
(even though the others may be only a tiny bit worse).
 
 Strategy for finding the best tree 
 
In practice, it is advisable to use the Jumble option to evaluate many
different orderings of the input species.   It is advisable to use the
Jumble option and specify that it be done many times (as many as
different orderings
of the input species).   (This is usually not necessary when bootstrapping,
though the programs will then default to doing it once to avoid artifacts
caused by the order in which species are added to the tree.)
 
People who want a magic "black box" program whose results they do
not have to question (or think about) often are upset that these
programs give results that are dependent on the order in which the species
are entered in the data.  To me this property is an advantage, for it
permits you to try different searches for better trees, simply by
varying the input order of species.  If you do not use the multiple Jumble
option, but do multiple individual runs instead, you
can easily decide which to pay most attention to - the one or ones that 
are best according to the criterion employed (for example, with parsimony, 
the one out of the runs that results in the tree with the fewest changes).
 
In practice, in a single run, it usually seems best to put species that are
likely to be sources of confusion in the topology last, as by the time they are
added the arrangement of the earlier species will have stabilized into a
good configuration, and then the last few species will by fitted into
that topology.  There will be less chance this way of a poor initial
topology that would affect all subsequent parts of the search.  However,
a variety of arrangements of the input order of species should be tried,
as can be done if the  J  option is used,
and no species should be kept in a fixed place in the order of input.
Note that the results of the "...penny" programs and Clique
are not sensitive to the input order of species, and Neighbor is only
slightly sensistive to it, so that multiple Jumbling is not possible
with those programs.  Note also that with global search, which
is standard in many programs and in others is an 
option, each group (including
each individual species) will be removed and re-added in all possible
positions, so that a species causing confusion will have more chance of moving
to a new location than it would without global rearrangement.
 
    
 
 A Warning on Interpreting Results  
 
Probably the most important thing to keep in mind while running any of the
parsimony or compatibility programs is not
to overinterpret the result.  Many users treat the set of most parsimonious
trees as if it were a confidence interval.  If a group appears in all of the
most parsimonious trees then they treat it as well established.  Unfortunately
 the confidence interval on phylogenies appears to be much
larger than the set of all most parsimonious trees  (Felsenstein, 1985b).
Likewise, variation of result among different methods will not be a good
indicator of the size of the confidence interval.  Consider a simple data set
in which, out of 100 binary characters, 51 recommend the unrooted tree
 ((A,B),(C,D))  and 49 the tree  ((A,D),(B,C)) .  Many different
methods will all give the same result on
such a data set: they will estimate the tree as  ((A,B),(C,D)) .
Nevertheless it is
clear that the 51:49 margin by which this tree is favored is not statistically
significantly different from 50:50.  So  consistency among different methods
is a poor guide to statistical significance .
 
    
 
 Relative Speed of Different 
Programs and Machines  
 
 Relative speed of the different programs 
 
C compilers differ in efficiency of the code they generate,
and some deal with some features of the language better than with
others.  Thus a program which is unusually fast on one computer may be
unusually slow on another.  Nevertheless, as a rough guide to relative
execution speeds, I have tested the programs on three data sets, each of
which has 10 species and 40 characters.  The first is an imaginary one
in which all characters are compatible - ("The Willi Hennig Memorial
Data Set" as J. S. Farris once called ones like it).  The second is the binary
recoded form of the fossil horses data set of Camin and Sokal (1965).
The third data set has data that is completely random: 10 species and 20
characters that have a 50% chance that each character state is  0  or
 1  (or  A  or  G ).  The data sets thus range from a completely
compatible one in which there is no homoplasy (paralellism or convergence), 
through the horses data set, which requires 29 steps where the possible 
minimum number would be 20, to the random data set, which requires 49 steps.  
We can thus see how this increasing messiness of the data affects running 
times.  The three data sets have all had 20 sites of  A 's added to the 
end of each sequence, so as to prevent likelihood or distance matrix programs 
from having infinite branch lengths (the test data sets used for timing
previous versions of PHYLIP were the same except that they lacked these
20 extra sites).
 
Here are the nucleotide sequence versions of the three data sets:
 
   
 
    10   40
A         CACACACAAAAAAAAAAACAAAAAAAAAAAAAAAAAAAAA
B         CACACAACAAAAAAAAAACAAAAAAAAAAAAAAAAAAAAA
C         CACAACAAAAAAAAAAAACAAAAAAAAAAAAAAAAAAAAA
D         CAACAAAACAAAAAAAAACAAAAAAAAAAAAAAAAAAAAA
E         CAACAAAAACAAAAAAAACAAAAAAAAAAAAAAAAAAAAA
F         ACAAAAAAAACACACAAAACAAAAAAAAAAAAAAAAAAAA
G         ACAAAAAAAACACAACAAACAAAAAAAAAAAAAAAAAAAA
H         ACAAAAAAAACAACAAAAACAAAAAAAAAAAAAAAAAAAA
I         ACAAAAAAAAACAAAACAACAAAAAAAAAAAAAAAAAAAA
J         ACAAAAAAAAACAAAAACACAAAAAAAAAAAAAAAAAAAA
 
   
 
   
 
    10   40
MesohippusAAAAAAAAAAAAAAAAAAAAAAAAAAAAAAAAAAAAAAAA
HypohippusAAACCCCCCCAAAAAAAAACAAAAAAAAAAAAAAAAAAAA
ArchaeohipCAAAAAAAAAAAAAAAACACAAAAAAAAAAAAAAAAAAAA
ParahippusCAAACAACAACAAAAAAAACAAAAAAAAAAAAAAAAAAAA
MerychippuCCAACCACCACCCCACACCCAAAAAAAAAAAAAAAAAAAA
M. secunduCCAACCACCACCCACACCCCAAAAAAAAAAAAAAAAAAAA
Nannipus  CCAACCACAACCCCACACCCAAAAAAAAAAAAAAAAAAAA
NeohippariCCAACCCCCCCCCCACACCCAAAAAAAAAAAAAAAAAAAA
Calippus  CCAACCACAACCCACACCCCAAAAAAAAAAAAAAAAAAAA
PliohippusCCCACCCCCCCCCACACCCCAAAAAAAAAAAAAAAAAAAA
 
   
 
   
 
    10   40
A         CACACAACCAAACAAACCACAAAAAAAAAAAAAAAAAAAA
B         AAACCACACACACAAACCCAAAAAAAAAAAAAAAAAAAAA
C         ACAAAACCAAACCACCCACAAAAAAAAAAAAAAAAAAAAA
D         AAAAACACAACACACCAAACAAAAAAAAAAAAAAAAAAAA
E         AAACAACCACACACAACCAAAAAAAAAAAAAAAAAAAAAA
F         CCCAAACACCCCCAAAAAACAAAAAAAAAAAAAAAAAAAA
G         ACACCCCCACACCCACCAACAAAAAAAAAAAAAAAAAAAA
H         AAAACAACAACCACCCCACCAAAAAAAAAAAAAAAAAAAA
I         ACACAACAACACAAACAACCAAAAAAAAAAAAAAAAAAAA
J         CCAAAAACACCCAACCCAACAAAAAAAAAAAAAAAAAAAA
 
   
 
Here are the timings of many of the version 3.6 programs on these three data
sets as run after being compiled by Gnu C (version 3.2) and run on an
AMD Athlon XP 2200+ computer under Linux.
 
 
 
    
 Hennigian Data 
 Horses Data 
 Random Data 
 
  Protpars 
 0.00500 
 0.00670 
 0.01289 
 
  Dnapars 
 0.01050 
 0.00940 
 0.00980 
 
  Dnapenny 
 0.01400 
 0.00860 
 1.71100 
 
  Dnacomp 
 0.00240 
 0.00250 
 0.00590 
 
  Dnaml 
 0.17749 
 0.23970 
 0.21350 
 
  Dnamlk 
 0.21740 
 0.19450 
 0.24400 
 
  Proml 
 1.3527 
 3.2085 
 2.0055 
 
  Promlk 
 3.3567 
 8.6078 
 4.4886 
 
  Dnainvar 
 0.00020 
 0.00020 
 0.00020 
 
  Dnadist 
 0.00140 
 0.00080 
 0.00150 
 
  Protdist 
 0.09220 
 0.09210 
 0.09310 
 
  Restml 
 0.14560 
 0.28810 
 0.21540 
 
  Restdist 
 0.00110 
 0.00090 
 0.00080 
 
  Fitch 
 0.00760 
 0.01280 
 0.00880 
 
  Kitsch 
 0.00180 
 0.00260 
 0.00280 
 
  Neighbor 
 0.00020 
 0.00050 
 0.00050 
 
  Contml 
 0.01310 
 0.01500 
 0.01780 
 
  Gendist 
 0.00070 
 0.00070 
 0.00070 
 
  Pars 
 0.00780 
 0.00610 
 0.02930 
 
  Mix 
 0.00360 
 0.00410 
 0.00610 
 
  Penny 
 0.00190 
 0.00470 
 0.8060 
 
  Dollop 
 0.00480 
 0.00450 
 0.00820 
 
  Dolpenny 
 0.00200 
 0.01060 
 1.1270 
 
  Clique 
 0.00100 
 0.00070 
 0.00130 
 
 
 
 
 

 
In all cases the programs were run under the default options with optimized 
compiler switches ( -03 -fomit-frame-pointer ), except as
specified here.  
The data sets used for the discrete characters programs have  0 's and  1 's
instead of  A 's and  C 's.  For Contml the  A 's and  C 's
were made into  0.0 's and  1.0 's and considered as 40 2-allele loci.
For the distance programs 10  x  10 distance matrices were
computed from the three data sets.
For the restriction sites programs  A  and  C  were changed into
 +  and  - .  It does not
make much sense to benchmark Move, Dolmove, or Dnamove, although when there
are many characters and many species the response time after each 
alteration of the tree should be proportional to the product of the number of
species and the number of characters.
For Dnaml, Dnamlk, and Dnadist the frequencies of the four bases were
set to be equal rather than determined empirically as is the default.  For
Restml the number of enzymes was set to 1.
 
In most cases, the benchmark was made more accurate by analyzing 100 data
sets using the  M  (Multiple data sets) option and dividing the resulting
time by 100.  Times were determined as user times using the Linux  time 
command.  Several patterns will be apparent from this.  The algorithms (Mix,
Dollop, Contml, Fitch, Kitsch, Protpars, Dnapars, Dnacomp, and
Dnaml, Dnamlk, Restml) that use the above-described addition strategy have
run times that do not depend strongly on the messiness of the data.  The only 
exception to this is that if a data set such as the Random data requires
extra rounds of global rearrangements it takes longer.  The
programs differ greatly in run time: the protein likelihood programs
Proml and Promlk were very slow, and the other likelihood programs
Restml, Dnaml and
Contml are slower than the rest of the programs.  The protein sequence parsimony
program, which has to do a considerable amount of bookkeeping to keep track of
which amino acids can mutate to each other, is also relatively slow.
 
Another class of algorithms includes Penny, Dolpenny, Dnapenny and Clique.
These are branch-and-bound methods: in principle they should have execution
times that rise exponentially with the number of species and/or
characters, and they might be much more sensitive to messy data.  This is
apparent with Penny, Dolpenny, and Dnapenny, which go from being reasonably
fast with clean data to very slow with messy data.  Dolpenny is particularly
slow on messy data - this is because this algorithm cannot make use of some of
the lower-bound calculations that are possible with Dnapenny and Penny.  Clique
is very fast on all
data sets.  Although in theory it should bog down if the number of cliques in
the data is very large, that does not happen with random data, which in
fact has few cliques and those small ones.  Apparently the "worst-case"
data sets that cause exponential run time are much rarer for Clique than for
the other branch-and-bound methods.
 
Neighbor is quite fast compared to Fitch and Kitsch, and should make it
possible to run much larger cases, although the results are expected to be
a bit rougher than with those programs.
 
 
 Speed with different numbers of species 
 
How will the speed depend on the number of species and the number
of characters?  For the sequential-addition algorithms, the speed should
be proportional to somewhere between the cube of the number of species and
the square of the number of species, and to the number
of characters.  Thus a case that has, instead of 10 species and 20
characters, 20 species and 50 characters would take (in the cubic case)
2  x  2  x  2  x  2.5 = 20
times as long.  This implies that cases with more than 20 species will
be slow, and cases with more than 40 species  very  slow.  This places a
premium on working on small subproblems rather than just dumping a whole
large data set into the programs.
 
An exception to these rules will be some of the DNA programs that use an
aliasing device to save execution time.  In these programs execution time
will not necessarily increase proportional to the number of sites,
as sites that show the same pattern of nucleotides will be detected
as identical and the calculations for them will be done only once, which does
not lead to more execution time.  This is particularly
likely to happen with few species and many sites, or with data sets that have
small amounts of evolutionary divergence.
 
For programs Fitch and Kitsch, the distance matrix is square, so
that when we double the number of species we also double the number of
"characters", so that running times will go up as the fourth power of
the number of species rather than the third power.  Thus a 20-species
case with Fitch is expected to run sixteen times more slowly than a 10-species
case.
 
For programs like Penny and Clique the run times will rise faster
than the cube of the number of species (in fact, they can rise faster
than any power since these algorithms are not guaranteed to work in
polynomial time).  In practice, Penny will frequently bog down above 11
species, while Clique easily deals with larger numbers.
 
For Neighbor the speed should vary only as the cube of the number of
species, so a case twice as large will take only eight times as long.  This
will make it an attractive alternative to Fitch and Kitsch for large data
sets.
 
 Suggestion:  If you are unsure of how long a program will take, try it first on
a few species, then work your way up until you get a feel for the speed
and for what size programs you can afford to run.
 
Execution time is not the most important criterion for a program,
particularly as computer time gets much cheaper than your time or a
programmer's time.  With workstations on which background jobs can be run
all night, execution speed is not overwhelmingly relevant.  Some of us have been
conditioned by an earlier era of computing to consider execution speed
paramount.  But ease of use, ease of adaptation to your computer system,
and ease of modification are much more important in practice, and in
these respects I think these programs are adequate.  Only if you are
engaged in 1960's style mainframe computing, or if you have very large
amounts of data is minimization of execution
time paramount.  If you spent six months getting your data, it may not be
overwhelmingly important whether your run takes 10 seconds or 10 hours.
 
Nevertheless it would have been nice to have made the programs
faster.  The present speeds are a compromise between speed and
effectiveness: by making them slower and trying more rearrangements in the 
trees, or by enumerating all possible trees, I could have made the programs
more likely to find the best tree.  By trying fewer rearrangements I
could have speeded them up, but at the cost of finding worse trees.  I
could also have speeded them up by writing critical sections in assembly
language, but this would have sacrificed ease of distribution to new
computer systems.  There are also some options included in these programs that
make it 
harder to adopt some of the economies of bookkeeping that make other programs 
faster.  However to some extent I have simply made the decision not to spend 
time trying to speed up program bookkeeping when there were new likelihood and 
statistical methods to be developed.
 
 
 Relative speed of different machines 
 
It is interesting to compare different machines using Dnapars as the
standard task.  One can rate a machine on the Dnapars benchmark by summing the
times for all three of the data sets.  Here are relative total timings over 
all three data sets (done with various versions of Dnapars) for some machines,
taking an AMD Athlon 1.2 GHz computer running Linux with gcc as the
standard.  Benchmarks from versions 3.4 and 3.5 of the program are
also included (respectively the Pascal and C versions whose timings are in
parentheses).  They are compared only with each other and are scaled to the
rest of the timings using the joint runs on the 386SX and the Pentium MMX 266.
This use of separate standards is necessary not
because of different languages but because different versions of the package
are being compared.  Thus, the "Time" is the ratio of the Total to that for
the Pentium, adjusted by the scalings of machines using 3.4 and 3.5 when
appropriate.  The Relative Speed is the reciprocal of the Time.
 
 
 
   Machine  
  Operating System  
  Compiler  
  Total  
  Time  
  Relative Speed  
 
  Toshiba T1100+ 
 MSDOS 
 Turbo Pascal 3.01A 
 (269) 
 10542 
 0.00009486 
 
  Apple Mac Plus 
 Mac OS 
 Lightspeed Pascal 2 
 (175.84) 
 6891 
 0.00014511 
 
  Toshiba T1100+ 
 MSDOS 
 Turbo Pascal 5.0 
 (162) 
 6349 
 0.00015750 
 
  Macintosh Classic 
 Mac OS 
 Think Pascal 3 
 (160) 
 6271 
 0.00015947 
 
  Macintosh Classic 
 Mac OS 
 Think C 
 (43.0) 
 4771 
 0.0002096 
 
  IBM PS2/60 
 MSDOS 
 Turbo Pascal 5.0 
 (58.76) 
 2303 
 0.0004343 
 
  80286 (12 Mhz) 
 MSDOS 
 Turbo Pascal 5.0 
 (47.09) 
 1845.4 
 0.0005419 
 
  Apple Mac IIcx 
 Mac OS 
 Think Pascal 3 
 (42) 
 1645.5 
 0.0006077 
 
  Apple Mac SE/30 
 Mac OS 
 Think Pascal 3 
 (42) 
 1645.6 
 0.0006077 
 
  Apple Mac IIcx 
 Mac OS 
 Lightspeed Pascal 2 
 (39.84) 
 1561.6 
 0.0006404 
 
  Apple Mac IIcx 
 Mac OS 
 Lightspeed Pascal 2# 
 (39.69) 
 1555.0 
 0.00006431 
 
  Zenith Z386 (16MHz) 
 MSDOS 
 Turbo Pascal 5.0 
 (38.27) 
 1539.0 
 0.0006498 
 
  Macintosh SE/30 
 Mac OS 
 Think C 
 (13.6) 
 1508.4 
 0.0006630 
 
  386SX (16 MHz) 
 MSDOS 
 Turbo Pascal 6.0 
 (34) 
 1333.6 
 0.0007498 
 
  386SX (16 MHz) 
 MSDOS 
 Microsoft Quick C 
 (12.01) 
 1333.6 
 0.0007499 
 
  Sequent-S81 
 DYNIX 
 Silicon Valley Pascal 
 (13.0) 
 509.0 
 0.0019646 
 
  VAX 11/785 
 Unix 
 Berkeley Pascal 
 (11.9) 
 466.3 
 0.002144 
 
  80486-33 
 MSDOS 
 Turbo Pascal 6.0 
 (11.46) 
 449.0 
 0.02227 
 
  Sun 3/60 
 SunOS 
 Sun C 
 (3.93) 
 435.7 
 0.002295 
 
  NeXT Cube (68030) 
 Mach 
 Gnu C 
 (2.608) 
 289.3 
 0.003456 
 
  Sequent S-81 
 DYNIX 
 Sequent Symmetry C 
 (2.604) 
 288.9 
 0.003461 
 
  VAXstation 3500 
 Unix 
 Berkeley Pascal 
 (7.3) 
 286.5 
 0.003491 
 
  Sequent S-81 
 DYNIX 
 Berkeley Pascal 
 (5.6) 
 219.5 
 0.004557 
 
  Unisys 7000/40 
 Unix 
 Berkeley Pascal 
 (5.24) 
 205.3 
 0.004870 
 
  VAX 8600 
 VMS 
 DEC VAX Pascal 
 (3.96) 
 155.23 
 0.006442 
 
  Sun SPARC IPX 
 SunOS 
 Gnu C version 2.1 
 (1.28) 
 142.04 
 0.007040 
 
  VAX 6000-530 
 VMS 
 DEC C 
 (0.858) 
 95.14 
 0.010511 
 
  VAXstation 4000 
 VMS 
 DEC C 
 (0.809) 
 89.81 
 0.011135 
 
  IBM RS/6000 540 
 AIX 
 XLP Pascal 
 (2.276) 
 89.14 
 0.011219 
 
  NeXTstation(040/25) 
 Mach 
 Gnu C 
 (0.75) 
 83.15 
 0.012027 
 
  Sun SPARC IPX 
 SunOS 
 Sun C 
 (0.68) 
 75.43 
 0.01326 
 
  486DX (33 MHz) 
 Linux 
 Gnu C # 
 (0.63) 
 69.95 
 0.01430 
 
  Sun SPARCstation-1 
 Unix 
 Sun Pascal 
 (1.7) 
 66.62 
 0.01501 
 
  DECstation 5000/200 
 Unix 
 DEC Ultrix C 
 (0.45) 
 49.97 
 0.02001 
 
  Sun SPARC 1+ 
 SunOS 
 Sun C 
 (0.40) 
 44.37 
 0.02254 
 
  DECstation 3100 
 Unix 
 DEC Ultrix Pascal 
 (0.77) 
 30.11 
 0.03321 
 
  IBM 3090-300E 
 AIX 
 Metaware High C 
 (0.27) 
 29.98 
 0.03336 
 
  DECstation 5000/125 
 Unix 
 DEC Ultrix C 
 (0.267) 
 29.58 
 0.03381 
 
  DECstation 5000/200 
 Unix 
 DEC Ultrix C 
 (0.256) 
 28.38 
 0.03524 
 
  Sun SPARC 4/50 
 SunOS 
 Sun C 
 (0.249) 
 27.62 
 0.03621 
 
  DEC 3000/400 AXP 
 Unix 
 DEC C 
 (0.224) 
 24.85 
 0.04024 
 
  DECstation 5000/240 
 Unix 
 DEC Ultrix C 
 (0.1889) 
 20.96 
 0.04771 
 
  SGI Iris R4000 
 Unix 
 SGI C 
 (0.184) 
 20.41 
 0.04898 
 
  IBM 3090-300E 
 VM 
 Pascal VS 
 (0.464) 
 18.12 
 0.05519 
 
  DECstation 5000/200 
 Unix 
 DEC Ultrix Pascal 
 (0.39) 
 15.188 
 0.06583 
 
  Pentium 120 
 Linux 
 Gnu C 
 1.848 
 11.953 
 0.08366 
 
  Pentium Pro 180 
 Linux 
 Gnu C 
 1.009 
 6.527 
 0.1532 
 
  Pentium 266 MMX 
 Linux 
 Gnu C (PHYLIP 3.5) 
 (0.054) 
 5.996 
 0.1668 
 
  Pentium 266 MMX 
 Linux 
 Gnu C 
 0.927 
 5.996 
 0.1668 
 
  Pentium 200 
 Linux 
 Gnu C 
 0.853 
 5.517 
 0.1812 
 
  SGI PowerChallenge 
 Irix 
 Gnu C 
 0.844 
 5.459 
 0.1832 
 
  DEC Alpha 400 4/233 
 DUNIX 
 Digital C (cc -fast) 
 0.730 
 4.722 
 0.2118 
 
  Pentium II 500 
 Linux 
 Gnu C 
 0.368 
 2.380 
 0.4201 
 
  Dual 448/633 MHz Pentiums 
 Linux 
 gcc 
 0.3069 
 1.985 
 0.5037 
 
  Sun Ultra 10 
 Solaris 8 
 gcc 
 0.25848 
 1.672 
 0.5981 
 
  Macintosh G3 300 MHz 
 Mac OS X 
 Gnu C (-O 3) 
 0.2330 
 1.5071 
 0.6635 
 
  Compaq/Digital Alpha 500au 
 DUNIX 
 Digital C (cc -fast) 
 0.167 
 1.080 
 0.9257 
 
  AMD Athlon 1.2 GHz 
 Linux 
 gcc 
 0.1546 
 1.0 
 1.0 
 
  Intel Pentium 4 2.26 GHz 
 Windows XP 
 Cygwin gcc 
 0.1078 
 0.6973 
 1.434 
 
  Pentium 4 1700 MHz 
 Linux 
 Gnu C 
 0.10730 
 0.6940 
 1.441 
 
  Macintosh G4 1.2GHz 
 Mac OS X 
 Gnu C (-O 3) 
 0.0582 
 0.3765 
 2.656 
 
 
 
 
 
This benchmark not only reflects integer performance of these machines
(as Dnapars has few floating-point operations) but also the efficiency
of the compilers.  Some of the machines (the DEC 3000/400 AXP
and the IBM RS/6000, in particular) are much faster than this benchmark
would indicate.  The numerical programs benchmark below gives them a
fairer test.  The Compaq/Digital Alpha 500au times are exaggerated because,
although their compiles are optimized for that processor, some of the Pentium
compiles are not similarly optimized.
 
Note that parallel machines like the Sequent and the SGI PowerChallenge are not
really as slow as indicated by the data here, as these runs did nothing to take
advantage of their parallelism.
 
These benchmarks have now extended over 17 years, and in the Dnapars
benchmark they extend over a range of over 28,000-fold in speed!
The experience of our laboratory, which seems typical, is that
computer power grows by a factor of about 1.85 per year.  This is
roughly consistent with these benchmarks.
 
For a picture of speeds for a more numerically intensive program,
here are benchmarks using Dnaml, with an AMD Athlon 1.2 GHz Linux system 
as the standard.  Some of the timings, the ones in parentheses, are
using PHYLIP version 3.5, and those are compared to that version run on
the Pentium 266.  Runs using the PHYLIP 3.4 Pascal version are adjusted
using the 386SX timings where both were run.  Numbers are
total run times (total user time in the case of Unix) over all three data sets.
 
 
 
   Machine  
  Operating System  
  Compiler  
  Seconds  
  Time  
  Relative Speed  
 
  386SX 16 Mhz 
 PCDOS 
 Turbo Pascal 6 
 (7826) 
 1027.55 
 0.0009732 
 
  386SX 16 Mhz 
 PCDOS 
 Quick C 
 (6549.79) 
 1027.55 
 0.0009732 
 
  Compudyne 486DX/33 
 Linux 
 Gnu C 
 (1599.9) 
 251.0 
 0.003984 
 
  SUN Sparcstation 1+ 
 SunOS 
 Sun C 
 (1402.8) 
 220.1 
 0.004543 
 
  Everex STEP 386/20 
 PCDOS 
 Turbo Pascal 5.5 
 (1440.8) 
 189.17 
 0.005286 
 
  486DX/33 
 PCDOS 
 Turbo C++ 
 (1107.2) 
 173.70 
 0.005757 
 
  Compudyne 486DX/33 
 PCDOS 
 Waterloo C/386 
 (1045.78) 
 164.07 
 0.006094 
 
  Sun SPARCstation IPX 
 SunOS 
 Gnu C 
 (960.2) 
 150.64 
 0.006638 
 
  NeXTstation(68040/25) 
 Mach 
 Gnu C 
 (916.6) 
 143.80 
 0.006954 
 
  486DX/33 
 PCDOS 
 Waterloo C/386 
 (861.0) 
 135.08 
 0.007403 
 
  Sun SPARCstation IPX 
 SunOS 
 Sun C 
 (787.7) 
 123.58 
 0.008091 
 
  486DX/33 
 PCDOS 
 Gnu C 
 (650.9) 
 102.12 
 0.009792 
 
  VAX 6000-530 
 VMS 
 DEC C 
 (637.0) 
 99.94 
 0.01001 
 
  DECstation 5000/200 
 Unix 
 DEC Ultrix RISC C 
 (423.3) 
 66.41 
 0.01506 
 
  IBM 3090-300E 
 AIX 
 Metaware High C 
 (201.8) 
 31.65 
 0.03159 
 
  Convex C240/1024 
 Unix 
 C 
 (101.6) 
 15.940 
 0.06274 
 
  DEC 3000/400 AXP 
 Unix 
 DEC C 
 (98.29) 
 15.42 
 0.06485 
 
  Pentium 120 
 Linux 
 Gnu C 
 25.26 
 19.230 
 0.05200 
 
  Pentium Pro 180 
 Linux 
 Gnu C 
 18.88 
 14.372 
 0.06957 
 
  Pentium 200 
 Linux 
 Gnu C 
 16.51 
 12.569 
 0.07956 
 
  SGI PowerChallenge 
 IRIX 
 Gnu C 
 12.446 
 9.475 
 0.10554 
 
  DEC Alpha 400 4/233 
 Linux 
 Gnu C (cc -fast) 
 8.0418 
 6.122 
 0.16335 
 
  Pentium MMX 266 
 Linux 
 Gnu C (PHYLIP 3.5) 
 (36.15) 
 5.671 
 0.17632 
 
  Pentium MMX 266 
 Linux 
 Gnu C 
 7.45 
 5.671 
 0.17632 
 
  Pentium II 500 
 Linux 
 Gnu C 
 6.02 
 4.583 
 0.2182 
 
  Dual 448/633 MHz Pentiums 
 Linux 
 Gnu C 
 3.7225 
 2.834 
 0.3529 
 
  Sun Ultra 10 
 Solaris 8 
 Gnu C 
 3.7101 
 2.824 
 0.3541 
 
  Pentium 4 1.7 GHz 
 Linux 
 Gnu C 
 2.0668 
 1.5734 
 0.6356 
 
  Macintosh G3 300 MHz 
 Mac OS X 
 Gnu C (-O 3) 
 1.805 
 1.3741 
 0.7278 
 
  Intel Pentium 4 2.26 GHz 
 Windows XP 
 Cygwin gcc 
 1.55457 
 1.1834 
 0.8450 
 
  AMD Athlon 1.2 GHz 
 Linux 
 Gnu C 
 1.3136 
 1.0 
 1.0 
 
  Compaq/Digital Alpha 500au 
 Linux 
 Gnu C (cc -fast) 
 0.9383 
 0.7143 
 1.4000 
 
  Macintosh G4 1.2 GHz 
 Mac OS X 
 Gnu C (-O 3) 
 0.7080 
 0.5390 
 1.8554 
 
 
 
 
As before, the parallel machines such as the Convex and the SGI PowerChallenge
were only run using one processor, which does not take into account the
gain that could be obtained by parallelizing the programs.  The speed of the
Compaq/Digital Alpha 500au is exaggerated because it was compiled in a way
optimized for its processor, while some of the Pentium compiles were not.
 
You are invited to send me figures for your machine for
inclusion in future tables.  Use the data sets above and compute the total
times for Dnapars and for Dnaml for the three data sets (setting the
frequencies of the four bases to 0.25 each for the Dnaml runs).  Be sure to
tell me the name and version of your compiler, and the version of PHYLIP you
tested.
If the times are too small to be measured accurately, obtain the times
for 10 or 100 data sets (the Multiple data sets option) and divide by 10 or
100.
 
    
 
 General Comments on Adapting 
the Package to Different Computer Systems  
 
In the sections following you will find instructions on how to adapt the 
programs to different computers and compilers.  The programs should compile
without alteration on most versions of C.  They use the "malloc" library
or "calloc" function to allocate memory so that the upper limits on how many
species or how many sites or characters they can run is set by the system memory
available to that memory-allocation function.
 
In the document file for each program, I have supplied a small
input example, and the output it produces, to help you check whether the
programs are running properly.
 
 
    
 Compiling the programs 
 
 
If you have not been able to get executables for PHYLIP, you should be
able to make your own.  This can be easy under Linux and Unix, but more
difficult if you have a Macintosh or a Windows system.  If you have the
latter, we strongly recommend you download and use the Macintosh and
Windows executables that we distribute.  If you do that, you will not need
to have any compiler or to do any compiling.  I get a certain number of
inquiries each year from confused users who are not sure what a compiler
is but think they need one.  After downloading the executables they
contact me and complain that they did not find a compiler included in the
package, and would I please e-mail them the compiler.  What they really
need to do is use the executables and forget about compiling them.
 
Some users may also need to compile the programs in order to modify them.
The instructions below will help with this.
 
I will discuss how to compile PHYLIP using one of a number of widely-used
compilers.  After these I will comment on compiling PHYLIP on other, less
widely-used systems.
 
 Unix and Linux 
 
In Unix and Linux (which is Unix in all important functional respects, if
not in all legal respects) it is often easy to compile PHYLIP yourself.
Unix (and Linux)
systems generally have a C compiler and have the  make  utility.  We
distribute with the PHYLIP source code a Unix-compatible  Makefile .
 
However, note that some popular Linux distributions do not include a C
compiler in their default configuration.  For example, in RedHat Linux version
8, the "Personal Workstation" installation that is the default does not
include the C compiler or the X Windows libraries needed to compile
PHYLIP.  These are available, and can be loaded from the CDROMs in the
distribution.  The following instructions assume that you have the
C compiler and X libraries.  If you cannot easily configure your system to
include them, you should look into using the RedHat RPM binary
distribution, mentioned on the PHYLIP 3.6 web page.
 
As is mentioned below (under Macintoshes) the Mac OS X operating system is
a Unix, and if the X windows windowing system is installed, these Unix
instructions will work for it.
 
After you have finished unpacking the Documentation and Source Code
archive, you will find that you have created a folder  phylip-3.6 
in which there are three
folders, called  exe ,  src , and  doc .  
There is also an HTML web page,  phylip.html .  The  exe 
folder
will be empty,  src  contains the source code files, including the
 Makefile .  Directory  doc  contains the documentation files.
 
Enter the  src  folder.  Before you compile, you will want to
look at the  Makefile  and see whether you want to alter the compilation
command.  We have the default C compiler flags set with no flags.  If you
have modified the programs, you might want to use the debugging flags
"-g".  On the other hand, if you are trying to make a fast executable using
the GCC compiler, you may want to use the one which is "An optimized one
for gcc".  In either case, remove the "#" before that CFLAGS command, and
place it before the CFLAGS command that was previously in use.
There are careful instructions on this in the Makefile.
Once you have set up the CFLAGS and DFLAGS statements to be the way you
want, to compile all the programs just type:
 
 make install 
 
You will then see the compiling commands as they happen, with
occasional warning messages.  If these are warnings, rather than errors,
they are not too serious.  A typical warning would be like this:
 
 dnaml.c:1204: warning: static declaration for re_move follows non-static 
 
After a time the compiler will finish compiling.  If you have done a
 make install  the system will then move the executables into the
 exe  folder and also save space by erasing all the relocatable
object files that were produced in the process.  You should be left with
useable executables in the  exe  folder, and the  src 
folder should be as before.   To run the executables, go into the
 exe  folder and type the program name (say  dnaml , which
you may or may not have to precede by a dot and a slash ./ ).
The names of the
executables will be the same as the names of the C programs, but without the
 .c  suffix.  Thus  dnaml.c  compiles to make an executable called  dnaml .
 
One caution is that our two tree-drawing programs, Drawgram and Drawtree,
need to have the Athena Widgets, which are provided with most X Windows
installations.  If you get error messages saying that some files with "Xaw"
in the name cannot be found, this means that either the Athena Widgets
are not installed on your system, or that the paths do not find them.
If they are there but not found during the compile, a simple change to the DLIBS
line in the Makefile may cure the problem.
 
If you see messages that the compilation could not find "Xlib.h" and other,
similar functions, this means that some parts of the X Windows development
environment is not installed on your system.  You will need to
make sure that they are installed properly.
 
Another is that the usual Linux C compiler is the Gnu GCC compiler.
In some Linux systems it is not invoked by the command  cc  but
by  gcc .  You would then need to edit the Makefile to reflect this
(see below for comments on that process).
 
A typical Unix or Linux installation would put the directory  phylip-3.6 
in  /usr/local .  The name of the executables directory  EXEDIR 
could be changed to be  /usr/local/bin , so that the  make install 
command puts the executables there.  If the users have  /usr/local/bin 
in their paths, the programs would be found when their names are typed.
The font files  font1  through  font6  could also be
placed there.  A batch script containing the lines
 
 
      ln -s /usr/local/bin/font1 font1
      ln -s /usr/local/bin/font2 font2
      ln -s /usr/local/bin/font3 font3
      ln -s /usr/local/bin/font4 font4
      ln -s /usr/local/bin/font5 font5
      ln -s /usr/local/bin/font6 font6
 
 
could be used to establish links in the user's working directory so that
Drawtree and Drawgram would find these font files when users
type a name such as  font1  when the program asks
them for a font file name.  The
documentation web pages are in subdirectory  doc  of the
main PHYLIP directory, except for one,  phylip.html  which is
in the main PHYLIP directory.  It has a table of all of the documentation
pages, including this one.  If users create a bookmark to that page
it can be used to access all of the other documentation pages.
 
To compile just one program, such as Dnaml, type:
 
 make dnaml 
 
After this compilation,  dnaml  will be in the  src 
subdirectory.  So will some relocatable object code files that
were used to create the executable.  These have names ending in
 .o  - they can safely be deleted.
 
If you have problems with the compilation command, you can edit the
 Makefile .  It has careful explanations at its front of how you
might want to do so.  For example, you might want to change the C
compiler name  cc  to the name of the Gnu C compiler,  gcc .
This can be done by removing the comment character  #  from the
front of one line, and placing it at the front of a nearby line.
How to do so should be clear from the material at the beginning of the
 Makefile .  We have included sample lines for using the  gcc 
compiler and for using the Cygwin Gnu C++ environment on Windows, as
well as the default of  cc .
 
Some older C compilers (notably the Berkeley C compiler which is
included free with some Sun systems) do not adhere to the ANSI C
standard (because they were written before it was set down).
They have trouble with the function prototypes which are in
our programs.  We have included an  #ifndef  preprocessor
command to eliminate the problem, if you use the switch  -DOLDC 
when compiling.  Thus with these compilers you need only use this in
your C flags (in the Makefile) and compilers such as Berkeley C
will cause no trouble. 
 
 Windows systems 
 
We distribute Windows executables, and most likely you can use these and
do not need to recompile them.  The following instructions will only be
necessary if you want to modify the programs and need to recompile them.
They are given for several different compilers available on Windows systems.
Another major compiler is Intel compiler -- we do not have information yet
on how to use it, but expect that PHYLIP will compile on it.
 
 Compiling with Cygnus Gnu C++ 
 
Cygnus Solutions (now a part of Red Hat, Inc.) has adapted the Gnu C compiler
to Windows systems and
provided an environment, CygWin, which mimics Unix for compiling.
Currently, this is the compiler that we use to prepare the Windows
executables.
Cygwin is available for purchase, and they also make it
available to be downloaded for free.  The download is large.  To get it, go
to  their download site  at
 http://sources.redhat.com/cygwin  and follow the
instructions there.  To download it you need to download
their  setup.exe  program and then it will download the rest
when it is run.  You will need a lot of disk space for it (about a gigabyte).
 
When installing Cygwin it is important to install gcc and make.  During
the course of the setup program Setup will ask you to select packages.
Expand the Devel Category by clicking on it.  Scroll down to gcc and
click on "skip".  "Skip" will change to the current version of gcc.
Scroll down to the make package and click on "Skip" again. These two
programs are nessessary to install phylip.
  
Once you have
installed the free CygWin environment and the associated Gnu C compiler
on your Windows system, compiling PHYLIP is closely similar to
what one does for Unix or Linux:
 
 
  Enter the Cygwin environment (which you can do using the Windows
 Start  menu and its  Programs  menu item.  There should be
a  Cygnus  menu choice within that submenu, which you can use to
start the Cygnus environment.  This puts you in an imitation of a Unix
shell.)  Alternatively you may have a CygWin icon on your desktop and you
can enter the environment by clicking on that.
 
  On entering the CygWin environment you will find yourself in one of the
folders within the CygWin folder.  Change to the folder where the
PHYLIP programs have been put.  For example, if you are user
"fred" on a Windows xp system, and have extracted our self-extracting
archive of the Sources and Documentation files onto your desktop,
you will want to change folders by typing the command
    cd C:/"Documents and Settings"/fred/phylip3.66/phylip3.6   
 
  Make sure that there is a folder there called  exe  as well as
one called  src .  The former is where the executables will be
copied if you do a full recompile of the package.  If you have our
existing executables in  exe  you might want to save them by
copying them elsewhere at this point.  If  exe  does not exist
then you should create it ( mkdir exe ), and also the folder  doc  if that does
not exist ( mkdir doc ).
Go into the folder  src  by typing the command  cd src .  There should be a folder  icons 
within this folder as well.
 
  Make sure that the file  Makefile  has been renamed as  Makefile.unix  and that a copy of  Makefile.cyg  has been made and renamed
as  Makefile .  We will have done this in the copy of the sources that
comes for the Windows platform; if you have instead obtained the sources
from those in another form of our distribution you may need to do these
renamings and copies yourself.
 
  You should then be able to compile PHYLIP
by issuing the appropriate make command, such as  make install .
If you have modified one of our source code files such as  dnaml.c ,
it would be wise to
have saved the original version of it first as, say,  dnaml.c0 .
 
  Our Makefile will automatically associate the appropriate icon from
the folder  icons  with the executables.  To associate an icon with
a program (say DnaML), we have an icon
file (say  dna.ico ) which contains the icon in standard format.
There is also a file called  dnaml.rc  which contains the single
line:
 
 dnaml ICON "dna.ico" 
 
We have provided a folder  icons  in the  src 
folder, containing a full set of icons and a full set of resource
files ( *.rc ) so you will not have to do this yourself.
 
 The executables will now be in the folder  exe .  You can run
them by clicking on their icons, or by using a Command window or a CygWin
window and typing their names ( dnaml , or  dnaml.exe , or
 ./dnaml.exe ).
 
 
 Compiling with Microsoft Visual C++  
 
With Microsoft Visual C++, you can compile using a Makefile.  We have supplied
this in the source code distrubution as  Makefile.msvc .
You will need to preserve the Unix Makefile by renaming  Makefile  to,
say,  Makefile.unix , then make a copy of  Makefile.msvc 
and call it  Makefile .   (You may have to change your windows'
settings to make the three-letter extensions visible, or you could use
the RENAME command in the Command tool).
 
 Setting the path. 
Before using  nmake  you will need to have the paths
set properly.  For this, use the Start menu to open Command or
a Dos Prompt first.  To set the path type 
 
set MSVC=Path
 
where Path is where Microsoft Visual Studio is installed 
(e.g. it might be in  c:\Microsoft Visual Studio ).
However the path you type should not have any spaces in it, so
that pathname cannot be used.
There are sometimes alternative paths that can get you to the
same place.  In my lab's Windows XP system, the path
 C:\msvsnet\Vc7  also leads to the same place, and that
pathname could be used.
You may have to work out an appropriate pathname yourself.
This means that you may have to use a folder's
DOS filename.  One way to get the DOS filename is to use the command
 DIR /X  in a Command window.
In general to get a DOS name you take the first six letters of 
the folder name and follow them by  ~1 . For example,
 Microsoft Visual Studio  will have a DOS name 
 Micros~1 ,  Program Files  will be  Progra~1 ).
Depending on what other
file are in the folder the DOS name may be the first six letters followed
by  ~2,~3,~4 , etc... (e.g.  Micros~3  or  Progra~5 ).
It may take some
experimentation to figure it out. With older Versions of Windows (pre-win2000)
it may be possible to just right click on the folder icon and select 
Properties to get the DOS name.
 
Once you have set MSVC, type
 
PATH=%PATH%;%MSVC%\VC98\bin
 
If you are using Visual C++ 6.0,
or type
 
PATH=%PATH%;%MSVC%\VC7\bin
 
if you are using Visual C++ .NET 2002 (or later).
 
Then the Makefile will need to be edited.  The line
 
MSVCPATH=c:\Micros~1\VC98
 
will need to be changed so that 
It points to wherever Microsoft Visual Studio is installed followed by
  \VC98  (for Visual Studio 6.0) or  \VC7  (for Visual
Studio C++ .NET 2002 or later).
 
 Using the Makefile . The Makefile is invoked using the
 nmake  command.  If you simply type  nmake  you
will get a list of possible  make  commands.  For example,
to compile a single program such as  Dnaml  but not
install it, type  nmake dnaml .  To compile and install all
programs type  nmake install .  We have supplied all the
support files and icons needed for the compilations.  They are
in folder  msvc  in the main source code
folder.
 
However, notice that as of latest Microsoft Visual C++ .NET 2002
some of the important compiler optimizations are available only in
the Professional edition, which is expensive.  For this reason, we have
switched to the GCC compiler for producing our distributed Windows
executables.
 
 Compiling with Borland C++  
 
Borland C++ can be downloaded for free from Inprise (Borland)
(see their site
 
http://www.borland.com/products/downloads/download_cbuilder.html .
You should download the compiler as it includes all the utilities needed 
to compile phylip.  It can compile using a Makefile. We have supplied 
this in the source code distribution as  Makefile.bcc . You will need to 
preserve the Unix Makefile by renaming it to, say,  Makefile.unix , then 
make a copy of  Makefile.bcc  and call it  Makefile . The
 Makefile  is invoked using the make command.
 
You will first need to create an  ilink32.cfg  and a  bcc32.cfg 
file and put the files into the  phylip-3.6/src  folder.  These files are text files
and their contents are described in the  readme.txt  that comes with the 
Borland tools.  If the Borland tools are in the default location the 
contents of  ilink32.cfg  would be.
 
 
-L"c:\Borland\Bcc55\lib"
 
 
and the contents of  bcc32.cfg 
 
 
-I"c:\Borland\Bcc55\include"
-L"c:\Borland\Bcc55\lib"
 
 
These files can be created in a text editor such as Notepad or Wordpad.
 
To invoke the  make  command you will first need to open a command
prompt window.  Then set the path appropriately.  To set the path, type
 
 
set BORLAND=Path
 
 
Where "Path" is where Borland is installed, such as  C:\Borland\BCC55 .
Then type
 
 
PATH=%PATH%;%BORLAND%\Bin
 
 
If you simply type  make  you will get a list of possible make commands. 
For example, to compile a single program such as Dnaml but not install 
it, type  make dnaml . To compile and install all programs type
 make install . We have supplied all the the support files and icons
needed for 
the compilations. They are in folder  bcc  of the main PHYLIP
ource code folder. 
We have had to supply a complete second set of the resource files with 
names  *.brc  because Borland resource files have a minor
incompatibility with Microsoft Visual C++ resource files.
 
 Compiling with Metrowerks Codewarrior for Windows  
 
As with Macintosh systems, Metrowerks Codewarrior requires 
you to have project files for each program you compile.
For Metrowerks Codewarrior for Windows we are not providing the projects
themselves, but we are providing
projects which have been exported as XML files. To open one of these one
cannot just click on 
File/Open but instead on the menu option File/Import Project.
Metrowerks will then ask you for the project name.
Type in the name of the program (e.g. dnaml). Once this is done Metrowerks will
act like this is a regular project file.
 
We have supplied a complete set of these XML project files in the
source code distribution.  They are in folder  metro 
of the main source code folder.  This is supplied with the
source code distribution for Windows (it is not in the source
code distributions for other platforms).
For Metrowerks Codewarrior for Windows we are not providing the projects
themselves, but we are providing
projects which have been exported as XML files. To open one of these one
cannot just click on
File/Open but instead on the menu option File/Import Project.
Metrowerks will then ask you for the project name.
Type in the name of the program (e.g. dnaml). Once this is done Metrowerks will
act like this is a regular project file.
 
To compile the program
pull down the  Project  menu and select  Make .  The
program should then compile, possibly with ignorable warning messages.
 
For the moment we are not giving here the details of
how to create these projects yourself -- you usually will not need
to, as you have the project files we have supplied.
 
 Macintosh 
 
 Compiling with GCC on Mac OS X with our Makefile 
 
The executables distributed by us for Mac OS X are currently compiled
using the GCC compiler that is distributed with Mac OS X.  You may not need
to recompile them, unless you want to make changes in the programs.
We are distributing "universal binaries" that work on both PowerMac and
Intel iMac.
You may not need to recompile unless you need to make a version of the
executables more closely adapted to your system, or unless you want to
modify the programs.
 
If you do want to recompile, conder the following:
 
  Make sure you have the GCC compiler installed.  If you are using a
Terminal window, you can check this by typing the command  gcc .
If the response is that the gcc compiler cannot find a program to compile,
then it is there.  If it is not there, you need to install it.
  Go to the Phylip  src  folder. Rename our default 
Unix  Makefile  to  Makefile.unix  (to keep it from getting
overwritten).
  Copy  Makefile.osx  and call the copy  Makefile .
This  Makefile  is needed to create clickable executables and store
them in the proper directories. In addition to
the Makefile, make sure that all the icon files
(for Dnaml, for example, the icon file is  dnaml.icns )
are present in the  mac  folder which is inside the
 src  folder, and so are  command.in , 
and  Info.plist.in .
  If you need to put the executables somewhere else other than the
 exe  folder, edit the  Makefile .
  Then type
 
 make install 
 
 You will see compiling remarks with occasional warning
messages, which should not be too serious. When done, the  make install 
command should have moved the executables to the  exe  subdirectory.
Note that this
 Makefile  also creates command line executables.
  The  Makefile  will create universal binaries that can run
on either PowerPC or on Intel iMacs.  If your compiler does not successfully
make universal binaries, you will want to edit the CC command in this
 Makefile , using the CC command following the one that makes
universal binaries.
 To run a program, open the  exe  directory (or wherever
you decided to put them) and click on 
the icon of the program. A Terminal window should appear, and you can type in
your choices from the menus that appear.   
  Alternatively, you may want to execute a program from within a Terminal
window by typing the program name.  We have provided in the  exe 
folder a file that makes links to the executables to enable this.  Go to the
executables folder and type the command  source linkmac  to create
a set of links.  These will run the program when the program name is typed
in a Terminal window which has been set so that its current
folder is the  exe  folder (but note that Drawgram and Drawtree run
this way may be slow to open and close plotting windows).
  Note that an alternative way to get command-line executables is to
compile for X11, as described in the next section.
 If you want to compile just one program, say Dnaml, and move it into
the executables directory use the command
 
 make dnaml.install 
 
 This compiles the executable  dnaml.app  and then moves
it to the  exe 
directory, and deletes the unnecessary associated files  dnaml.o 
and  dnaml  from the  src  directory.  
 
 
 Compiling with GCC on Mac OS X with X Windows 
 
On Mac OS X systems you can also use the GCC compiler and X Windows to
compile a version of the executables that runs from the command line
in native mode.
To do that, you must have the GCC compiler and the X11 windows
development kit materials installed.
X Windows is part of the default distribution of Mac OS X for version
10.3 (Panther) and later, but it needs to be installed.  For earlier versions
of Mac OS X a version of it
is available at  http://www.apple.com/macosx/x11/download/ 
and it is easy to download and install on a Mac OS X system.
 
If you have the GCC compiler and the X11 libraries installed, you can use a
Terminal window (which you
will find available in the Utilities folder in the Applications folder) and
compile PHYLIP by treating it as a Unix or Linux application and following the
instructions given above under "Unix and Linux".  Basically you just get
into the folder that contains the PHYLIP source code and type
 
make install
 
This uses the ordinary Unix/Linux Makefile, which works well for
Mac OS X with the gcc compiler.
 
 Compiling with Metrowerks Codewarrior on Macintosh 
 
The Metrowerks Codewarrior compiler was widely used on Macintosh PowerMac
systems.  More recently it is owned by Freescale, Semiconductor, and seems to
be fading from the general Macintosh compiler market.
This will assume that you have a recent version of the Metrowerks
Codewarrior  C++
compiler.  This description, and the project files that we provide,
assume Codewarrior 8.3.  We also assume some familiarity with
the use of the Codewarrior compiler and its Integrated Development
Environment (IDE).  These instructions are for native-mode compiling
under Mac OS X, and also for Mac OS 9 or for the Classic
environment under Mac OS X.
 
Start with our  src  folder (directory) that contains the C source
code files such as  dnaml.c  and also the Codewarrior resource
files such as  dnaml.rsrc , which are provided by us.
 
We have provided a full set of project files in the
self-extracting Macintosh Stuffit archive.
Usually users will have no need to compile
the programs, but occasionally they may want to change a setting or
add a feature.  In that case the Metrowerks Codewarrior compiler can be
used.  We have provided support for compiling the programs in its
most recent version, version 8.3.  The following discussion will
assume that you have obtained and installed the compiler.
 
You should find in the source code folder 
 src  a folder called  mac  which contains the
Metrowerks Codewarrior compiler "project files" (with names ending in
 .proj , as well as the resource files (which end in  .rsrc 
for each program.  You can get into this folder, activate the
Metrowerks compiler, and open the appropriate project file.  To
compile the program, simply make sure that the project file is an
active window, and type  Command-M  (which is to say, hold down
the  Command  key while typing  M ).  Alternatively,
pull down the  Project  window and select  Make .  The
program should then compile, possibly with ignorable warning messages.
 
 Creating the Metrowerks project file. 
 
As said above, for the Metrowerks compiler we have provided
you with a complete set of project files.   Generally, you will not need to
create project files.   But if you do want to create them and compile
using them yourself, we have
instructions here.  We will use Dnaml as our example:
 
 Start up the Codewarrior IDE integrated development environment.
 Create a new project file by choosing  New...  on the  File 
menu.
 Type in the project name  dnaml.proj 
 On the Project menu on the left side of the  New  window, double-click on  Mac OS C Stationery 
 In the  New project  window that opens, click on the triangle to the left of  Mac OS Classic  (if you are using Mac OS 9) or
 Mac OS X Mach-O  (if you are using Mac OS X).
 Then click on the triangle to the left of  Standard Console .
 Click on the menu item  SIOUX-WASTE C Classic  (if you are using
Mac OS 9) or  C Console SIOUX Mach-O  (if you are using Mac OS X).
 Press the  OK  button.  After a bit a window  dnaml.proj  will open.
 Click on the triangle to the left of the  Sources  menu item.  A
template item called  HelloWorld.c  will open.
 Select  HelloWorld.c .
 Open the  Edit  menu at the top of the Mac screen and select
 Clear .  A box will open asking if you want to remove  HelloWorld.c  from the project.
 Select  OK .
 If the  dnaml.c  file came from the self-extracting Macintosh
archive that we distribute, it should show a yellow-and-back-striped Metrowerks
icon (if not, as when you get it from some other form of our distribution,
you may have to pass it through a program like Microsoft Word, making
sure to save it as a Text Only file, to get
Metrowerks to be able to see it as a potential source code file).
 Drag the  dnaml.c  file onto the  Sources  item in your
 dnaml.proj  window.
 Drop it onto Sources so that it appears under the  Sources  choice.
This may take a few tries -- if it appears above  Sources  grab it
and move it again.
 Now add the other files that must be compiled with  dnaml.c .
These can be identified by looking at our  Makefile  -- for Dnaml
they are  seq.c ,  phylip.c ,  seq.h , and  phylip.h .  Each of them needs to be added to the project file in the same way that
 dnaml.c  was.
 In addition to the files indiciated in the  Makefile ,
 macface.c  and  macface.h  (or  interface.c  and 
 interface.h  if making the project file for drawtree or drawgram)
need to be added to the project in the same way as mentioned in the previous
step.
 Drag  dnaml.rsrc  (if you are using Mac OS 9) or  dna.plc 
and  dna.icns  (if you are using Mac OS X) into  Sources  in the
same way.  It doesn't matter whether these appear before or after
 dnaml.c .
 Go to the  Edit  menu and select the  PPC Std C SIOUX-WASTE Settings  item.  A window of that name will then open.
 Under the  Target  item select  PPC Target  (if you are using
Mac OS X). A window will open to the right.
 Change the name in the  File Name  box to be  dnaml  (if you
are using Mac OS 9) or  dnaml.app  (if you are using Mac OS X).
 If you are using Mac OS 9, change the  ????  in the  Creator 
box to (say)  PHYD 
 Change the  Preferred Heap Size  to  1024  (you may also
need to have it default to larger than that).
 Under  Language Settings  in the left-hand menu of the window,
select  C/C++ Language .  A window called  C/C++ Language 
will open to the immediate right.
  (If you are using Mac OS X) Change the Prefix File from 
 MSLCarbonPrefix.h  to  osx_mwcw_prefix.h  (this file should be
included with our source code files).
 Click on  Require Function Prototypes  to deselect that setting.
 Click on the  Save  button at the lower-right of the project
settings window.
 Close the  PPC Std C SIOUX-WASTE Settings  window using the usual
box in the upper-left corner.
 On your Desktop you should now find a folder  PHYLIP .
If it has a
file called  HelloWorld.c  you may want to discard that file.
 In that  PHYLIP  folder you will find a file  dnaml.proj .
 Double-click on that project file.  If the Metrowerks is not already open,
it should open now.
 If a window called  Project Messages  opens and there is a
complaint in it about access paths being wrong, you should fix these by
selecting the  Reset project entry paths  item in the  Project 
menu.
 In the  Project  menu, select  Make 
 
 
 VMS VAX systems 
 
We have not tried to compile version 3.6 on an OpenVMS system but the
following instructions should work.
On the OpenVMS operating system with DEC VAX VMS C the programs will compile
without alteration.  The commands for compiling a typical program
(Dnapars, which depends on the separately compiled files  phylip.c 
and  seq.c ) are:
 
 $ DEFINE LNK$LIBRARY SYS$LIBRARY:VAXCRTL
 
$ CC DNAPARS.C
 
$ CC PHYLIP.C
 
$ CC SEQ.C
 
$ LINK DNAPARS,PHYLIP,SEQ
 
 
 
Once you use this  $ DEFINE  statement during a given interactive session,
you need not repeat it again as the symbol  LNK$LIBRARY  is thereafter
properly defined.  The compilation process leaves a file  DNAPARS.OBJ 
in your directory: this can
be discarded.  The executable program is named  DNAPARS.EXE .  To run the program
one then uses the command:
 
 $ R DNAPARS 
 
The compiler defaults to the filenames  INFILE. ,  OUTFILE. , and
 TREEFILE. .
If the input file  INFILE.  does not exist the program will prompt you to
type in its name.  Note that some commands on VMS such as  TYPE OUTFILE 
will fail because the name of the file that it will attempt to type out will be not
 OUTFILE.  but  OUTFILE.LIS .  To get it to type the write file you
would have to instead issue the command  TYPE OUTFILE. .
 
When you are
using the interactive previewing feature of Drawgram (or Drawtree) on
a Tektronix or DEC ReGIS compatible terminal, you will want before
running the program to have issued the command:
 
 $ SET TERM/NOWRAP/ESCAPE 
 
so that you do not run into trouble from the VMS line length limit of
255 characters or the filtering of escape characters.
 
To know which files to compile together, look at the entries in the
 Makefile .
 
VMS systems are rapidly disappearing, so we will not devote much
effort to get PHYLIP working on them.
 
 Parallel computers 
 
As parallel computers become more common, the issue of how to compile
PHYLIP for them has become more pressing.  People have been compiling
PHYLIP for vector machines and parallel machines for many years.  We
have not made a version for parallel machines because there is still
no standard parallel programming environment on such machines (or rather,
there are many standards, so that one cannot find one that makes
a parallel execution version of PHYLIP widely distributable).  However
symmetric multiprocessing using the
MPI Message Passing Interface is spreading rapidly, and we will
probably support it in future versions of PHYLIP.
 
Although the underlying algorithms of most programs,
which treat sites independently, should be amenable to vector and
parallel processors,
there are details of the code which might best be changed.
In certain of the programs ( Dnaml ,  Dnamlk ,
 Proml ,  Promlk ) I have put a special
comment statement next to the loops in the program where
the program will spend most of its time, and which are the places
most likely to benefit from parallelization.  This comment statement is: 
 
           /* parallelize here */
 
In particular
within these innermost loops of the programs there are often scalar quantities
that are used for temporary bookkeeping.  These quantities, such as
 sum1, sum2, zz, z1, yy, y1, aa, bb, cc, sum,  and  denom  in procedure makenewv
of  Dnaml  and similar quantities in procedure nuview) are there to
minimize the number of array references.  For vectorizing and parallelizing
compilers it will 
be better to replace them by arrays so that processing can occur
simultaneously. 
 
If you succeed in making a parallel version of PHYLIP we would like to
know how you did it.  In particular, if you can prepare a web page which
describes how to do it for your computer system, we would like to use
material from it
in our PHYLIP web pages.  Please e-mail it to me.  We hope to
have a set of pages that give detailed instructions on how to make parallel
version of PHYLIP on various kinds of machines.  Alternatively, if we
were given your modified version of the program we might be able to
figure out how to make modifications to our source code to allow
users to compile the program in a way which makes those modifications.
 
 Other computer systems 
 
As you can see from the variety of different systems on which these
programs have been successfully run, there are no serious
incompatibility problems with most computer systems.  PHYLIP in various
past Pascal versions has also been compiled on 8080 and Z80 CP/M Systems, Apple
II systems running UCSD Pascal, a variety of minicomputer systems such as
DEC PDP-11's and HP 1000's, on 1970's era mainframes such as CDC
Cyber systems, and so on.  In a later era
it was also compiled on IBM 370 mainframes, and of course on DOS and
Windows systems and on Macintosh systems.
We have gradually
accumulated experience on a wider variety of C compilers.  If you succeed in
compiling the C version of PHYLIP on a different machine or a different
compiler, I would like to
hear the details so that I can consider including the instructions in a future version
of this manual.
 
 
    
 Frequently Asked Questions  
 
This set of Frequently Asked Questions, and their answers, is from the
PHYLIP web site.  A more up-to-date version can be found there, at:
 
 
 
 http://evolution.gs.washington.edu/phylip/faq.html   
 
 
 Problems that are encountered 
 
 
  "It doesn't work!  It doesn't work!!  It says  can't find infile. " 
 Actually, it's working just fine.  Many of the programs look for an input file called  infile ,
and if one of that name is not present in the current folder, they then ask
you to type in the name of the input file.  That's all that it's doing. This
is done so that
you can get the program to read the file without you having to type in its
name, by making a copy of your input file and calling it  infile .
If you don't do that, then the program issues this message.  It looks
alarming, but really all that it is trying to do is to get you to type in
the name of the input file.  Try giving it the name of the input file.
  "The program reads my data file and then says it has
a memory allocation error!" 
 This is what tends to happen if there is a problem with the format of the data
file, so that the programs get confused and think they need to set aside memory
for 1,000,000 species or so.  The result is a "memory allocation error" (the
error message may say that "the function asked for an inappropriate amount of memory").  Check the data file format against the documentation:
make sure that the data files have  not  been saved in the format of
your word processor (such as Microsoft Word) but in a "flat ASCII" or "text only"
mode.  Note that adding memory to your computer is  not  the
way to solve this problem -- you probably have plenty of memory
to run the program once the data file is in the correct format.
  "On our Mac OS 9 system, larger data files fail to run." 
 We have set the memory allowances on the Mac OS 9 executables
to be generous, but not too big.  You therefore may need to
increase them.  Use the  Get Info  item on the Finder  File  menu.
  "I opened the program but I don't see where to create
a data file!" 
 The programs (there are more than one) use data
files that have been created outside of the program.  They do not have any
data editor within them.  You can create a data file by using an editor,
such as Microsoft Word, EMACS, vi, SimpleText, Notepad, etc.  But be sure
 not  to save the file in Microsoft Word's own format.  It should be saved in
Text Only format.  You can use the documentation files, including the examples
at the end of those files, to figure out the format of the input file.
Documentation files such as  main.html ,  sequence.html ,
 distance.html  and many others should be consulted.  Many users
create their data files by having their alignment program (such as
ClustalW), output its alignments in PHYLIP format.  Many alignment programs
have options to do that.
  "I ran PHYLIP, and all it did was say it was extracting a bunch of files!" 
 
There is no executable program
named  PHYLIP  in the PHYLIP package!  But in some cases
(especially the Windows distribution) there is a file called
 phylip.exe .
That file is an archive of documentation and source code.  Once you have
run it and extracted the files in it, so that they are in the folder,
running it again will just do the extraction again, which is unnecessary.
Similarly for the archive files for the Windows executables, which
have names like  phylipwx.exe  and  phylipwy.exe .
They are run only once to extract their contents.
  "One program makes an output file and then the next program crashes while reading it!" 
 Did you rename the file?  If a program makes a file called  outfile , and then the
next program is told to use  outfile  as its input file, terrible things will
happen.  The second program first opens  outfile  as an output file, thus
erasing it.  When it then tries to read from this empty  outfile 
a psychological
crisis ensues.  The solution is simply to rename  outfile  before trying to
use it as an input file.
  "I make a file called infile and then the program can't find it!" 
 Let me guess.  You are using Windows, right?  You made your file in Word or
in Notepad or WordPad, right?  If you made a file in one of these editors, and
saved it, not in Word format, but in Text Only format, then you were doing the
right thing.  But when you told the operating system to save the file as
 infile , it actually didn't.  It saved it as
 infile.txt . Then just to make
life harder for you, the operating system is set up by default to not show
that three-letter extension to the file name.  Next to its icon it will show
the name  infile .  So you think, quite reasonably, that
there is a file called  infile .  But there isn't a file of that
name, so the program, quite reasonably, can't find a file called
 infile .  If you want to check what the actual file name is, use
the  Properties 
menu item of the  File  item on your folder (in Windows versions, anyway).  
You should be able to get the program to work by telling it that the file name
is  infile.txt .
  "Consense gives wierd branch lengths! How do I
get more reasonable ones?"  
 Consense gives branch lengths which are simply the numbers of replicates
that support the branch.  This is not a good reflection of how long those
branches are estimated to be.  The best way to put better branch lengths on a
consensus tree is to use it as a User Tree in a program that will estimate
branch lengths for it, such as DnaML.  You may need to convert it to being an unrooted tree,
using Retree, first.  If the original program you were using was a
program that does not estimate branch lengths, you may instead have to
use one that does.  You can use a likelihood program, or make
some distances between your species (using, for example, Dnadist) and use
Fitch to put branch lengths on the user tree.  Here is the sequence of
steps you should go through:
 
 Take the tree and use Retree to make sure it is Unrooted (just
read it into Retree and then save it, specifying Unrooted)
 Use the unrooted tree as a User Tree (option  U ) in one of
our programs (such as Fitch or Dnaml).   If you use Fitch, you also
first need to use one of the distance programs such as Dnadist to
compute a set of distances to serve as its input.
 Specify that the branch lengths
of the tree are not to be used but should be re-estimated.  This
is actually the default.
 
  "I looked at the tree printed in the output file  outfile 
and it looked wierd.  Do I always need to look at it in Drawgram?" 
 It's possible you are using the wrong font for looking at the tree in
the output file.  The tree is drawn with dashes and exclamation points.  If
a proportional font such as Times Roman or Helvetica is used, the tree lines
may not connect.  Try selecting the whole tree and setting the font to
a fixed-width one such as Courier.  You may be astounded how much clearer
the tree has become.
  "DrawTree (or DrawGram) doesn't work: it can't find the font file!" 
 Six font files, called  font1  through  font6 , are
distributed with the executables
(and with the source code too).  The program looks for a copy of one of them
called  fontfile .  If you haven't made such a copy called
 fontfile  it then asks
you for the name of the font file.  If they are in the current folder, just
type one of  font1  through  font6 .  The reason for
having the program look for  fontfile 
is so that you can copy your favorite font file, call the copy
 fontfile ,
and then it will be found automatically without you having to type the name of
the font file each time.
  "Can Drawgram draw a scale beside the tree? Print the branch lengths as numbers?" 
 It can't do either of these.  Doing so would make the program more complex, and
it is not obvious how to fit the branch length numbers into a tree that has
many very short internal branches.  If you want these scales or numbers,
choose an output plot file format (such as Postscript, PICT or PCX) that can be read by
a drawing program such as Adobe Illustrator, Freehand, Canvas, CorelDraw,
or MacDraw.
Then you can add the scales and branch length numbers yourself by hand.  Note
the menu option in Drawtree and Drawgram that specifies the tree size to be
a given number of centimeters per unit branch length.
  "How can I get Drawgram or Drawtree to print the bootstrap values
next to the branches?" 
 When you do bootstrapping and use Consense, it prints the bootstrap
values in its output file (both in a table of sets, and on the diagram
of the tree which it makes).  These are also in the output tree file of
Consense.  There they are in place of branch lengths.  So to get them to
be on the output of Drawgram or Drawtree, you must write the tree in the
format of a drawing program and use it to put the values in by hand, as
mentioned in the answer to the previous question.
  "I have an HP laser printer and can't get DrawGram to print on it" 
 Drawgram and Drawtree produce a plot file (called  plotfile ): they
do not send it to the printer.  It is up to you to get the plot file to 
the printer.  If you are running Windows this can probably be done
with the Command tool and the command  COPY/B PLOTFILE PRN: , unless
your printer is a networked printer.  The  /B 
is important.  If it is omitted the copy command will strip off the
highest bit of each byte, which can cause the printing to fail or produce
garbage.
  "Dnaml won't read the treefile that is produced by Dnapars!" 
 That's because the Dnapars tree file is a rooted tree, and Dnaml wants an
unrooted tree.  Try using Retree to change the file to be an unrooted tree
file. Our most recent versions of the programs usually automatically
convert a rooted tree into an unrooted one as needed.  But the programs
such as Dnamlk or Dollop that need a rooted tree won't be able to use an
unrooted tree.
  "What is a good value for the random number seed?" 
  The random number seed is used to start a process of choosing "random"
(actually pseudorandom) numbers, which behave as if they were
unpredictably randomly chosen between 0 and 2 32 -1 (which is
4,294,967,295).  You could put in the number 133 and find that the
next random number was 221,381,825.  As they are effectively
unpredictable, there is no such thing as a choice that is better than
any other, provided that the numbers are of the form 4 n +1.  However
if you re-use a random number seed, the sequence of random numbers
that result will be the same as before, resulting in exactly the same
series of choices, which may not be what you want.
  "In bootstrapping, Seqboot makes too large a file" 
 If there are 1000 bootstrap replicates, it will make a file
1000 times as long as your original data set.  But for many methods
there is another way that uses much less file space.  You can use
Seqboot to make a file of multiple sets of weights, and use those
together with the original data set to do bootstrapping.
  "In bootstrapping, the output file gets too big." 
  When running a program such as Neighbor or Dnapars with multiple data
sets (or multiple weights) for purposes of bootstrapping,
the output file is usually not needed, as it
is the output tree file that is used next.  You can use the menu
of the program to turn off the writing of trees into the
output file.  The trees will still be written into the output tree file.
  "Why don't your programs correctly read the sequence alignment
files produced by ClustalW?" 
 They do read them correctly if you make the right kind.  Files from
ClustalV or ClustalW whose names end in  ".aln"  are not in PHYLIP
format, but in Clustal's own format which will not work in PHYLIP.
You need to find the option to output PHYLIP format files, which ClustalW and
ClustalV usually assign the extension  .phy .
  "Why doesn't Neighbor read my DNA sequences correctly?" 
 Because it  wants
to  have as input a distance matrix, not sequences.  You have to use Dnadist to
make the distance matrix first.
 
 
 How to make it do various things 
 
 
  "How do I bootstrap?" 
 The general method of bootstrapping
involves  running  Seqboot  to make multiple bootstrapped data sets out of your
one data set, then running one of the tree-making programs  with  the  Multiple
data  sets option to analyze them all, then running Consense to make a majority
rule consensus tree from the resulting tree file.  Read  the  documentation  of
Seqboot  to  get  further information.  Before, only parsimony methods could be
bootstrapped.  With this new system almost any of the  tree-making  methods  in
the package can be bootstrapped.  It is somewhat more tedious but you will find
it much more rewarding.
  "How do I specify a multi-species outgroup
with your parsimony  programs?"  
 
 
It's  not  a  feature  but  is  not too hard to do in many of the programs.  In
parsimony programs like Mix, for which the W (Weights) and A (Ancestral states)
options are available, and weights can be larger than 1, all you need to do is:
 
   (a)      In Mix, make up an extra character with states 0 for  all  the  outgroups
and  1  for all the ingroups.  If using  Dnapars the ingroup can have (say)
 G  and the outgroup  A .  
   (b)      Assign this character an enormous weight
(such as  Z  for 35) using  the  W
option,  all other characters getting weight 1, or whatever weight they had
before.  
   (c)      If it is available, Use the A (Ancestral
states) option to designate that
for  that  new  character the state found in the  outgroup is the ancestral
state.  
   (d)   In Mix do not use the O (Outgroup) option.
  
   (e)       After the tree is found, the designated
ingroup  should  have  been  held
together  by the fake character.  The tree will be  rooted somewhere in the
outgroup (the program may or may not have a preference for  one  place  in
the  outgroup  over  another).   Make sure that you subtract from the total
number of steps on the tree all steps in the new character.  
  In programs like Dnapars, you cannot use this method as weights  of  sites
cannot  be  greater  than  1.   But you do an analogous trick, by adding a
largish number of extra sites to the data, with one nucleotide state ("A")
for the ingroup and another ("G") for the outgroup.  You will then have to
use Retree to manually reroot the tree in the desired place.  
 
 
  "How do I force certain groups to remain  monophyletic in your
parsimony programs?"  
 By  the same method as in the previous question, using multiple fake characters, any number of
groups of species can be forced to be  monophyletic.   In  Move,  Dolmove,  and
Dnamove  you  can  specify  whatever  outgroups  you want without going to this
trouble.
  "How can I reroot one of the trees written out by PHYLIP?" 
 Use the program
Retree.  But keep in mind whether the tree inferred by the original program was
already rooted, or whether you are free to reroot it without changing its
meaning.
  "What do I do  about  deletions  and  insertions  in  my  sequences?" 
 The
molecular  sequence  programs  will  accept  sequences  that have gaps (the " - "
character).  They do various things with them,  mostly  not  optimal.   Dnapars
counts  "gap"  as  if it were a fifth nucleotide state (in addition to A, C, G,
and T).  Each site counts one change when a  gap  arises  or  disappears.   The
disadvantage  of  this  treatment is that a long gap will be overweighted, with
one event per gapped site.  So a gap of 10 nucleotides will count as  being  as
much  evidence  as  10  single site nucleotide substitutions.  If there are not
overlapping gaps, one way to correct this is to recode the first  site  in  the
gap  as " - " but make all the others be " ? " so the gap only counts as one event.
Other programs such as Dnaml and Dnadist count gaps as  equivalent  to  unknown
nucleotides  (or  unknown  amino  acids) on the grounds that we don't know what
would be there if  something  were  there.   This  completely  leaves  out  the
information  from  the presence or absence of the gap itself, but does not bias
the gapped sequence to be close  to  or  far  from  other  gapped  or  ungapped
sequences.
So it is not necessary to remove gapped regions from your
sequences, unless the presence of gaps indicates that the region is
badly aligned.
  "How can I produce distances for my data set which
has 0's and 1's?"  
 You can't do it in a simple and general
way, for a straightforward reason.  Distance methods must correct the
distances for superimposed changes.  Unless we know specifically how to
do this for your particular characters, we cannot accomplish the
correction.  There are many formulas we could use, but we can't choose
among them without much more information.  There are issues of superimposed
changes, as well as heterogeneity of rates of change in different
characters.  Thus we have not provided a distance program for 0/1 data.
It is up to you to figure out what is an appropriate stochastic model
for your data and to find the right distance formulas.
  "I have RFLP fragment data: which programs should I
use?" 
 This is a more difficult question than you may imagine.
Here is quick tour of the issues:
  You can code fragments as 0 and 1 and use a parsimony program.  It is
not obvious in advance whether 0 or 1 is ancestral, though it is likely that
change in one direction is more likely than change in the other for each
fragment.  One can use either Wagner parsimony (programs  PARS ,
 Mix ,  Penny  or  Move ) or use Dollo parsimony
( Dollop, Dolpenny  or  Dolmove )
with the ancestral states all set as unknown (" ? ").
 You can use a distance matrix method using the RFLP distance of Nei and
Li (1979).  Their restriction fragment distance is available in our
program RestDist. 
 You should be very hesitant to bootstrap RFLP's.  The individual
fragments do not evolve independently: a single nucleotide substitution
can eliminate one fragment and create two (or vice versa).
 
For restriction  sites  (rather than fragments) life is a bit
easier: they evolve nearly independently so bootstrapping is possible
and  Restml  can be used, as well as restriction sites distances
computed in  Restdist .  Also directionality of change
is less ambiguous when parsimony is used.  A more complete tour of the
issues for restriction sites and restriction fragments is given in chapter
15 of my book (Felsenstein, 2004).
  "Why don't your parsimony programs  print  out  branch  lengths?" 
 Well, Dnapars and PARS can.  The others have not yet been upgraded to the
same level.  The longer answer is that it is because
there  are  problems  defining  the branch lengths.  If you look closely at the
reconstructions of the states of the hypothetical ancestral  nodes  for  almost
any  data  set  and  almost  any  parsimony method you will find some ambiguous
states on those nodes.  There is then usually an ambiguity as to  which  branch
the  change  is  actually  on.  Other parsimony programs resolve this in one or
another arbitrary fashion, sometimes with the user specifying how (for example,
methods  that push the changes up the tree as far as possible or down it as far
as possible).  Our older programs leave it to the user to do this.  In
Dnapars and PARS we use an algorithm discovered by Hochbaum and Pathria (1997)
(and independently by Wayne Maddison) to compute branch lengths that average
over all possible placements of the changes.  But these branch lengths, as
nice as they are, do not correct for mulitple superimposed changes.  Few
programs  available  from  others  currently  correct  the  branch  lengths for
multiple changes of state that may have overlain each other.  One possible  way
to  get  branch  lengths  with  nucleotide  sequence  data  is to take the tree
topology that you got, use Retree to convert  it  to  be  unrooted,  prepare  a
distance matrix from your data using Dnadist, and then use Fitch with that tree
as User Tree and see what branch lengths it estimates.
  "Why can't your programs handle unordered multistate  characters?" 
 In this 3.6 release there is a program PARS which does parsimony for
undordered multistate characters with up to 8 states, plus  ? .  The
other the discrete characters parsimony programs can only handle two states,
 0  and  1 .
This is mostly because I have not yet had time to modify them to do so  -  the
modifications would have to be extensive.  Ultimately I hope to get these done.
If you have four or fewer states and need a feature that is not in PARS,
you could  recode your states to look like nucleotides
and use the parsimony programs in the molecular sequence section of PHYLIP, or
you could use one of the excellent parsimony programs produced by others.
 
 
 Background information needed: 
 
 
  "What file format do I use for the sequences?" 
"How do I use the programs?  I can't find any documentation!" 
 These are discussed in the documentation files.  Do you have them?  If you
have a copy of this page you probably do.  They are
in a separate archive from the executables (they are in the Documentation and
Sources archives, which you should definitely fetch).  Input file formats
are discussed in  main.html , in  sequence.html ,  distance.html ,
 contchar.html ,  discrete.html , and the documentation files for the
individual programs.
  "Where can I find out how to infer
phylogenies?" 
 There are now a few books.  For molecular data you could use one of these:
 
At the upper-undergraduate level:
 
  Graur, D. and W.-H. Li.  2000.   Fundamentals of Molecular
     Evolution.  Sinauer Associates, Sunderland, Massachusetts. (or the earlier edition
     by Li and Graur).
  Page, R. D. P. and E. C. Holmes.  1998.   Molecular Evolution: 
     A Phylogenetic Approach.   Blackwell, Oxford.
 
 
and as graduate-level texts:
 
  Nei, M. and S. Kumar.  2000.   Molecular Evolution and
     Phylogenetics.  Oxford University Press, Oxford.
  Li, W.-H.  1999.   Molecular Evolution.   Sinauer Associates,
     Sunderland,   Massachusetts.
 
 
For more mathematically-oriented readers, there is the book
 
 Semple, C., and M. Steel. 2003.  Phylogenetics.  Oxford Lecture
Series in Mathematics and Its Applications, volume 24. Oxford
University Press, Oxford.
 
 
Best of all is of course my own book on phylogenies, which
covers the subject for many data types, at a
graduate course level: 
 
 Felsenstein, J.  2004.   Inferring Phylogenies .
Sinauer Associates, Sunderland, Massachusetts.
 
 
There are also some recent books that take a more practical hands-on approach,
and give some detailed information on how to use programs, including PHYLIP
programs.  These include:
 
 Hall, B. G.  2004.   Phylogenetic Trees Made Easy , 2nd edition.
Sinauer Associates, Sunderland, Massachusetts.
 Salemi, M., and A.-M. Vandamme (eds.) 2003.  The Phylogenetic Handbook.
   A Practical Approach to DNA and Protein Phylogeny.   Cambridge University
Press, Cambridge.
 
 
In addition, one of these three review articles may help:
 
 Swofford, D. L., G. J. Olsen, P. J. Waddell, and D. M. Hillis.  1996.
Phylogenetic inference.  pp. 407-514 in  Molecular Systematics , 2nd ed.,
ed.  D. M. Hillis, C. Moritz, and B. K. Mable.  Sinauer Associates, Sunderland,
Massachusetts.
 Felsenstein, J. 1988. Phylogenies from molecular sequences: inference and
reliability.   Annual Review of Genetics   22:  521-565.
 Felsenstein, J. 1988. Phylogenies and quantitative
characters.  Annual Review of Ecology and Systematics   19:  445-471.
 
 
A useful article introducing the inference of phylogenies at a more
elementary level is:
 
 Baldauf, S. L.  2003.  Phylogeny for the faint of heart: a tutorial.  Trends
in Genetics   19:  345-351.
 
 
I have already mentioned above that there is an excellent guide to using PHYLIP 3.6
for molecular analyses available.  It is by Jarno Tuimala:
 
 Tuimala, J.  2004.  A Primer to Phylogenetic Analysis using Phylip Package.
  2nd edition.  Center for Scientific Computing, Espoo, Finland.
 
and it is
available as a PDF  here .
 
 
 Questions about distribution and citation: 
 
 
  "If I copied PHYLIP from a friend without you knowing, should I try
to keep you from finding out?" 
 No.  It is to your advantage and mine for you to
let me know.  If you did not get PHYLIP "officially" from me  or  from  someone
authorized  by me, but copied a friend's version, you are not in my database of
users.   You  may also  have  an  old  version  which  has   since   been
substantially  improved.  I  don't  mind  you  "bootlegging"
PHYLIP (it's free anyway), but
you should realize that you may have copied an outdated version. If you are reading this
Web page, 
you can get  the  latest  version  just  as  quickly over Internet.
It will help both of us if you get
onto my mailing list.  If you are on it, then I will give your  name  to  other
nearby  users  when  they ask for the names of nearby users, and they are urged to contact you and
update  your  copy.   (I  benefit  by  getting  a  better  feel  for  how  many
distributions  there have been, and having a better mailing list to use to give
other users local people to contact).  Use the registration form which
can be accessed through our web site's registration page.
  "How do I make a citation  to  the  PHYLIP  package in  the  paper I am
writing?"  
 One way is like this:
 
Felsenstein, J.  2004.  PHYLIP (Phylogeny Inference Package) version 3.6.
 Distributed by the author.  Department of Genome Sciences, University of
Washington, Seattle. 
 
or if the editor for whom you are writing insists that the citation must be  to
a  printed  publication,  you  could cite a notice for version 3.2 published in
Cladistics:
 
Felsenstein, J.  1989.  PHYLIP - Phylogeny Inference Package (Version 3.2).
 Cladistics   5:  164-166.
 
 
For a while a printed version of the PHYLIP documentation was available and one
could  cite that.  This is no longer true.  Other than that, this is difficult,
because I have never written a paper announcing  PHYLIP!   My  1985b  paper  in
Evolution on the bootstrap method contains a
one-paragraph Appendix describing the availability of this  package,  and  that
can  also  be  cited  as  a  reference  for  the  package, although it was
distributed since 1980 while the bootstrap paper is 1985.   A paper  on  PHYLIP
is needed mostly to give people something to cite, as word-of-mouth, references
in other people's papers, and electronic newsgroup  postings  have  spread  the
word about PHYLIP's existence quite effectively.
  "Can I make copies of PHYLIP available to the students in
my class?" 
 Generally, yes.  Read the Copyright notice near the front of
this main documentation page.  If you charge money for PHYLIP,
or use it in a service for which you charge money, you will need
to negotiate a royalty.  But you can make it freely available
and you do not need to get any special permission from us to do so.
  "How many copies of PHYLIP have been distributed?" 
 On
27 September, 1996 we reached 5,000 registered installations worldwide.
(By now we are well over 15,000 but have lost count for
the moment).  Of course there are
many more people who have got copies from friends.  PHYLIP is the  most  widely
distributed  phylogeny  package.
In recent years  magnetic  tape  distribution, diskette distribution and e-mail distribution of
PHYLIP have disappeared (as I
insist people use the network if they can).  But all this has
been  more  than  offset  by, first, an explosion of distributions by anonymous ftp
over Internet, and then a bigger explosion of World Wide Web distributions and
registrations (about 8 registrations per day at the moment).
 
 
 Questions about documentation 
 
 
  "Where can I get a printed version of  the  PHYLIP  documents?" 
 For  the
moment,  you  can  only  get  a  printed  version by printing it yourself.  For
versions 3.1 to 3.3 a printed version was sold by Christopher Meacham  and  Tom
Duncan,  then  at  the  University Herbarium of the University of California at
Berkeley.  But they have had to discontinue this as it was too much work.   You
should  be  able to print out the documentation files on almost any printer and
make yourself a printed version of whichever of them you need.
  "Why have I been dropped from your newsletter mailing list?" 
 You haven't.
The  newsletter  was  dropped.  It simply was too hard to mail it out to such a
large mailing list.  The last issue of the newsletter  was  Number  9  in  May,
1987.  The Listserver News Bulletins that we tried for a while have also been dropped
as too hard to keep up to date.  I am hoping that our World Wide Web site will take their place.
 
 
 
 Additional Frequently Asked Questions, or: 
"Why didn't it occur to you to ...  
 
  ... allow the options to be set on the command line?" 
 We could in Unix and Linux, or somewhat differently in Windows.  But
there are so many options that this would be difficult, especially
when the options require additional information to be supplied such as
rates of evolution for many categories of sites.  You may be asking this
question because you want to automate the operation of PHYLIP programs
using batch files (command files) to run in background.  If that is the
issue, see the section of this main documentation page on
"Running the programs in background or under control of a command file".
It explains how to set the options using input redirection and a file
that has the menu responses as keystrokes.
  ... write these programs in Java?" 
 Well, we might.  It is not completely clear which of two contenders,
C++ and Java, will become more widespread, and which one will gradually
fade away.  Whichever one is more successful, we will probably want to use
for future versions of PHYLIP.  As the C compilers that are used to
compile PHYLIP are usually also able to compile C++, we will be moving in
that direction, but with constant worrying about whether to convert PHYLIP
to Java instead.
  ... forgot about all those inferior systems and just develop PHYLIP for Unix?" 
 This is self-answering, since the same people first said I should 
just develop it for Apple II's, then just for CP/M Z-80's, then just
for IBM PCDOS, then just for Macintoshes or for Sun 
workstations, and then for Windows.  If I had listened to them and done any one of these, I would 
have had a very hard time adapting the package to any of the other ones once 
these folks changed their mind (and most of them did)!
  ... write these programs in Pascal?" 
 These programs started out
in Pascal in 1980.  In 1993 we released both Pascal and C versions.  The
present version (3.6) and
future versions will be C-only.  I make fewer mistakes in Pascal and do
like the language better than C, but C has overtaken Pascal and Pascal
compilers are starting to be hard to find on some machines.  Also C is a
bit better standardized which makes the number of modifications a user
has to make to adapt the programs to their system much less.
  ... write these programs in PROLOG
(or Ada, or Modula-2, or SIMULA, or BCPL, or PL/I, or APL, or LISP)?" 
 These are all languages I have considered.  All
have advantages, but they are not really widespread (as are C, C++, and Java).
  ... include in the package a program to do the Distance Wagner method, (or
successive approximations character weighting)?" 
 In most cases where I have not
included other methods, it is because I decided that they had no substantial
advantages over methods that were included (such as the programs Fitch, 
Kitsch, Neighbor, the  T  option of Mix and Dollop, and the " ? " ancestral
states option of the discrete characters parsimony programs).
  ... include in the package ordination methods and more
clustering algorithms?" 
 Because this is  not  a clustering package, it's a
package for phylogeny estimation.  Those are different tasks with different
objectives and mostly different methods.  Mary Kuhner and Jon Yamato have,
however,
included in Neighbor an option for UPGMA clustering, which will be very
similar to Kitsch in results.
  ... include in the package a program to do nucleotide sequence 
alignment?" 
 Well, yes, I should
have, and this is scheduled to be in future releases.  But multiple sequence
alignment programs, in the era after Sankoff, Morel, and Cedergren's 1973
classic paper, need to use substantial computer horsepower to estimate the
alignment and the tree together (but see Karl Nicholas's program
 GeneDoc  or Ward Wheeler and David Gladstein's  MALIGN , as
well as more approximate methods of tree-based alignment used in
 ClustalW ,  TreeAlign , or  POY ).
 
 
 
 (Fortunately) obsolete questions  
 
(The following four questions, once
common, have finally disappeared, I am pleased to report).
 "Why didn't it occur to you to ...  
 
  ... let me log in to your computer in Seattle
and copy the files out over a phone line?" 
 No thanks.  It would cost you for a lot of
long-distance telephone time, plus a half hour of my time and yours in which
I had to explain to you how to log in and do the copying.
  ... send me a listing of your program?" 
 Damn it, it's not "a program",
it's 35 programs, in a great many files.  What were you
thinking of doing, having 1800-line programs typed in by slaves at your
end?  If you were going to go to all that trouble why not try network
transfer?  If you have these then you can print out all the
listings you want to and add them to the huge stack of printed output in
the corner of your office.
  ... write a magnetic tape in our computer center's favorite format
(inverted Lithuanian EBCDIC at 998 bpi)?" 
 Because the ANSI standard
format is the most widely used one, and even though your computer center
may pretend it can't read a tape written this way, if you sniff around
you will find a utility to read it.  It's just a  lot  easier for me to
let you do that work.  If I tried to put the tape into your format, I
would probably get it wrong anyway.
  ... give us a version of these in FORTRAN?" 
 Because the
programs are  far  easier to write and debug in C or Pascal, and cannot
easily be
rewritten into FORTRAN (they make extensive use of recursive calls and
of records and pointers).  In any case, C is widely available.  If you don't
have a C compiler or don't know
how to use it, you are going to have to learn a language like C or
Pascal sooner or later, and the sooner the better.
 
 
    
 
 New Features in This Version  
 
Version 3.6 has many new features:
  Faster (well, less, slow) likelihood programs.
 The DNA and protein likelihood and distance programs allow
for rate variation between sites using a gamma distribution of
rates among sites, or using a gamma distribution plus a given
fraction of sites which are assumed invariant.
 A new multistate discrete characters parsimony program, Pars, that
handles unordered multistate characters.
 The Dnapars and Pars parsimony programs can infer multifurcating
trees, which sensibly reduces the number of tied trees they find.
 A new protein sequence likelihood program, Proml,
and also a version, Promlk which assumes a molecular clock.
 A new restriction sites and restriction fragments distance program,
Restdist, that can also be used to compute distances for RAPD and
AFLP data.  It also allows for gamma-distributed rate variation among
DNA sites.
 In the DNA likelihood programs, you can now specify different
categories of rates of change (such as rates for first, second, and
third positions of a coding sequence) and assign them to specific sites.
This is in addition to the ability of the program to use the Hidden Markov
Model mechanism to allow rates of change to vary across sites in a way that
does not ask you to assign which rate goes with which site.
 The input files for many of the programs are now
simpler, in that they do not contain options information such as specification
of weights and categories.  That information is now provided in separate
files with default names such as  weights  and  categories .
 The DNA likelihood programs can now evaluate multifurcating
user trees (option  U ).
 All programs that read in user-defined trees now do so from a separate
file, whose default name is  intree , rather than requiring them to
be in the input file as before.
 The DNA likelihood programs can infer the sequence at ancestral
nodes in the interior of the tree.
 Dnapars can now do transversion parsimony.
 The bootstrapping program Seqboot now can, instead of producing a
large file containing multiple data sets, be asked instead
to produce a weights file with multiple sets of weights.  Many
programs in this release can analyze those multiple weights together with
the original data set, which saves disk space.
 The bootstrapping program Seqboot can pass weights and categories
information through to a multiple weights file or a multiple categories
file.
 Seqboot can also convert sequence files from Interleaved to
Sequential form, or back.
 Seqboot can convert a PHYLIP molecular sequences or discrete characters
morphology data file into the NEXUS format, which is used by a number of
other phylogeny programs such as MacClade, MrBayes and PAUP*.
 Seqboot can also carry out a number of different methods of
permuting the order of characters in a data set.  This could be used to
carry out the Incongruence Length Difference (or Partition Homogeneity)
method of testing homogeneity of data sets.
 Seqboot can also write a sequence data file into one version of
an XML format for sequence alignments,
for use by programs that need XML input
(none of the current PHYLIP programs can yet use this format, but it
may be useful in the future).
 Retree can now write tree out into a preliminary version of a new XML tree
file format which is in the process of being defined.
 The Kishino-Hasegawa-Templeton (KHT) test which compares user-defined
trees (option U) is now joined by the Shimodaira-Hasegawa (SH) test
(Shimodaira and Hasegawa, 1999) which corrects for comparisons among
multiple tests.  This avoids a statistical problem with multiple user trees.
 Contrast can now carry out an analysis that takes into account
within-species variation, according to a model similar (but not
identical) to that introduced by Michael Lynch (1990).  This enables
analysis of individuals sampled from the species, in a way that properly
takes sampling error into account.
 A new program, Treedist, computes the Robinson-Foulds symmetric
difference distance among trees.  This measures the number of branches in
the trees that are present in one but not the other.
It also can compute the Branch Score distance defined by Kuhner and Felsenstein
(1994) which takes branch lengths into account.
 Fitch and Kitsch now have an option to make trees by the
minimum evolution distance matrix method.
 The protein parsimony program Protpars now allows you to choose among
a number of different genetic codes such as mitochondrial codes.
 The consensus tree program Consense
can compute the M l  family of consensus tree methods, which
generalize the Majority Rule consensus tree method. It can
also compute our extended Majority Rule consensus (which is
Majority Rule with some additional groups added to resolve the
tree more completely), and it can also compute the original
Majority Rule consensus tree method which does not add these
extra groups.  It can also
compute the Strict consensus.
 The tree-drawing programs Drawgram and Drawtree have a number of new
options of kinds of file they can produce, including Windows Bitmap files,
files for the Idraw and FIG X windows drawing programs, the POV ray-tracer,
and even VRML Virtual Reality Markup Language files that will enable you
to wander around the tree using a VRML plugin for your browser, such as
Cosmo Player or Cortona.
 Drawtree now uses my new Equal Daylight Algorithm to draw unrooted
trees.  This gives a much better-looking tree.  Of course, competing programs
such as TREEVIEW and PAUP draw trees that look just as good - because they
too have started to use my method (with my encouragement).  Drawtree also
can use another algorithm, the n-body method.
 The tree-drawing programs can now produce trees across multiple
pages, which is handy for looking at trees with very large numbers
of tips, and for producing giant diagrams by pasting together
multiple sheets of paper.
 
 
There are many more, lesser features added as well.
 
    
 
 Coming Attractions, Future Plans  
 
There are some obvious deficiencies in this version.  Some of these
holes will be filled in the next few releases (leading to version
4.0).  They include:
 
 Obviously we need to start thinking about a more visual mouse/windows
interface, but only if that can be used on X windows, Macintoshes, and
Windows.
 Program Penny and its relatives will improved so as to run faster
and find all most parsimonious trees more quickly.
 An "evolutionary clock" version of Contml will be done, and the same 
may also be done for Restml.
 We are gradually generalizing the tree structures in the programs to
infer multifurcating trees as well as bifurcating ones.
We should be able to have any program read any tree and know what to do
with it, without the user having to fret about whether an unrooted tree was
fed to a program that needs a rooted tree.
 In general, we need more support for protein sequences, including a
codon model of change, allowing for different rates for synonymous
and nonsynonymous changes.
 We also need more support for combining runs from multiple loci,
allowing for different rates of evolution at the different loci.
 We will be expanding our use and production of XML data set files and
XML tree files.
 A program to align molecular sequences on a predefined User Tree may
ultimately be included.  This will allow alignment and phylogeny
reconstruction to procede iteratively by successive runs of two programs, one
aligning on a tree and the other finding a better tree based on that alignment.
In the shorter run a simple two-sequence alignment program may be included.
 An interactive "likelihood explorer" for DNA sequences will be written.
This will allow, either with or without the assumption of a molecular
clock, trees to be varied interactively so that the user can get a much
better feel for the shape of the likelihood surface.  Likelihood will be
able to be plotted against branch lengths for any branch.
 If possible we will allow use of Hidden Markov Models for correcting for
purine/pyrimidine richness variations among species, within the framework of
the maximum likelihood programs.  That the maximum likelihood programs do not
allow for base composition variation is their major limitation at the moment.
 The Hidden Markov Model (regional rates) option of Dnaml and Dnamlk will
be generalized to allow
for rates at sites to gradually change as one moves along the tree,
in an attempt to implement Fitch and Markowitz's (1970) notion of "covarions".
 A more sophisticated compatibility program should be included, if I can
find one.
 We are economizing on the size of the source code, and enforcing some
standardization of it, by putting frequently used routines in separate
files which can be linked into various programs.  This will enforce
a rather complete standardization of our code.
 We will move our code to an object-oriented
language, most likely C++.  One could describe the language that version
3.4 was written in as "Pascal", version 3.5 as "Pascal written in C",
version 3.6 as "C written in C", and maybe version 4.0 as "C++ written
in C" and then 4.1 as "C++ written in C++".  At least that scenario
is one possibility.
 
 
Much of the future development of the package will be in the DNA and protein
likelihood programs and the distance matrix programs.  This is for several
reasons.  First, I am more interested in those problems.  Second, collection of
molecular data is increasing rapidly, and those programs have the most promise
for future development
for those data.
 
    
 
 Endorsements  
 
Here are some comments people have made in print about PHYLIP.  Explanatory
material in square brackets is my own.  They fall naturally into three groups:
 
 From the pages of  Cladistics : 
 
 
"Under no circumstances can we recommend PHYLIP/WAG [their name for the
Wagner parsimony option of Mix]."
 
Luckow, M. and R. A. Pimentel (1985)
 
 
 
 
"PHYLIP has not proven very effective in implementing parsimony (Luckow and
Pimentel, 1985)."
 
J. Carpenter (1987a)
 
 
 
 
"... PHYLIP.  This is the computer program where every newsletter concerning
it is mostly bug-catching, some of which have been put there by previous
corrections.  As Platnick (1987) documents, through dint of much labor useful
results may be attained with this program, but I would suggest an
easier way: FORMAT b:"
 
J. Carpenter (1987b)
 
 
 
 
"PHYLIP is bug-infested and both less effective and orders of
magnitude slower than other programs ...."
 
"T. N. Nayenizgani" [J. S. Farris] (1990)
 
 
 
 
"Hennig86 [by J. S. Farris] provides such substantial improvements over
previously available programs (for both mainframes and microcomputers) that
it should now become the tool of choice for practising systematists."
 
N. Platnick (1989)
 
 
 
 ... in the pages of other journals: 
 
 
"The availability, within PHYLIP of distance, compatibility, maximum likelihood,
and generalized `invariants' algorithms (Cavender and Felsenstein, 1987) sets
it apart from other packages .... One of the strengths of PHYLIP is its
documentation ...."
 
Michael J. Sanderson (1990)
 
 (Sanderson also criticizes PHYLIP for slowness and inflexibility of its
parsimony algorithms, and compliments other packages on their strengths). 
 
 
 
"This package of programs has gradually become a basic necessity to anyone
working seriously on various aspects of phylogenetic inference .... The package
includes more programs than any other known phylogeny package.  But it is not
just a collection of cladistic and related programs.  The package has great
value added to the whole, and for this it is unique and of extreme
importance .... its various strengths are in the great array of methods
provided ...."
 
Bernard R. Baum (1989)
 
 
(note also W. Fink's critical remarks (1986) on version 2.8 of PHYLIP).
 
 
 
 ... and in the comments made by users when they register: 
 
 
"a program on phylogeny --
PHYLOGENY INTERFERENCE PACKAGE (PHYLIP).  We would therefore like to ask ..."
 
 
[names withheld] (in 1994)
 
 
 
"I am struglling with your clever programs."
 
 
[name withheld] (in 1995)
 
 
 
"I'm famously computer illiterate - I look forward to many frustrating hours trying to run this program"
 
 
Desmond Maxwell (in 1998)
 
 
 
"I am a brave man.  PHYLIP is a brave program.  We'll do fine together."
 
 
Christopher Winchell (in 2000)
 
 
 
"The Mahabarata of phylogenetics looks better than ever."
 
 
Ross Crozier (in 2001)
 
 
"I love phylip.  Tastes great and less filling!"
 
 
Byron Adams (in 2002)
 
    
 
 References for the Documentation Files  
 
In the documentation files that follow I frequently refer to papers
in the literature.  In order to centralize the references they are given
in this section.  If you want to find further papers beyond these, my
book (Felsenstein, 2004) lists more than 1,000 further references.
 
Adams, E. N.  1972.  Consensus techniques and the comparison of
taxonomic trees.   Systematic Zoology   21:  390-397.
 
Adams, E. N.  1986.  N-trees as nestings: complexity, similarity, and
consensus.   Journal of Classification   3:  299-317.
 
Archie, J. W.  1989.  A randomization test for phylogenetic information in
systematic data.   Systematic Zoology   38:  239-252.
 
Barry, D., and J. A. Hartigan.  1987.  Statistical analysis of hominoid
molecular evolution.   Statistical Science    2:  191-210.
 
Baum, B. R.  1989.  PHYLIP: Phylogeny Inference Package. Version 3.2. (Software
review).   Quarterly Review of Biology   64:  539-541.
 
Bron, C., and J. Kerbosch.  1973.  Algorithm 457: Finding all cliques
of an undirected graph.   Communications of the Association for Computing Machinery   16:  575-577.
 
Camin, J. H., and R. R. Sokal.  1965.  A method for deducing branching
sequences in phylogeny.   Evolution   19:  311-326.
 
Carpenter, J.  1987a.  A report on the Society for the Study of Evolution
workshop "Computer Programs for Inferring Phylogenies".   Cladistics   3: 
363-375.
 
Carpenter, J.  1987b.  Cladistics of cladists.   Cladistics   3:  363-375.
 
Cavalli-Sforza, L. L., and A. W. F. Edwards.  1967.  Phylogenetic
analysis: models and estimation procedures.   Evolution   32:  550-570
(also  American Journal of Human Genetics   19:  233-257).
 
Cavender, J. A. and J. Felsenstein.  1987.  Invariants of phylogenies in a
simple case with discrete states.   Journal of Classification   4:  57-71.
 
Churchill, G.A.  1989.  Stochastic models for heterogeneous DNA sequences.
 Bulletin of Mathematical Biology   51:  79-94.
 
Conn, E. E. and P. K. Stumpf.  1963.   Outlines of Biochemistry.   John Wiley
and Sons, New York.
 
Day, W. H. E.  1983.  Computationally difficult parsimony problems in
phylogenetic systematics.   Journal of Theoretical Biology   103: 
429-438.
 
Dayhoff, M. O. and R. V. Eck.  1968.   Atlas of Protein Sequence
and Structure 1967-1968.   National Biomedical Research Foundation,
Silver Spring, Maryland.
 
Dayhoff, M. O., R. M. Schwartz, and B. C. Orcutt.  1979.  A model of
evolutionary change in proteins.  pp. 345-352 in  Atlas of
Protein Sequence and Structure, volume 5, supplement 3, 1978,  ed.
M. O. Dayhoff.  National Biomedical Research Foundation, Silver Spring, Maryland
.
 
Dayhoff, M. O.  1979.   Atlas of Protein Sequence and Structure, Volume 5,
Supplement 3, 1978.   National Biomedical Research Foundation, Washington, D.C.
 
DeBry, R. W. and N. A. Slade.  1985.  Cladistic analysis of restriction
endonuclease cleavage maps within a maximum-likelihood framework.
 Systematic Zoology   34:   21-34.
 
Dempster, A. P., N. M. Laird, and D. B. Rubin.  1977.  Maximum
likelihood from incomplete data via the EM algorithm.   Journal of the Royal Statistical Society B   39:  1-38.
 
Eck, R. V., and M. O. Dayhoff.  1966.   Atlas of Protein Sequence and
Structure 1966.   National Biomedical Research Foundation, Silver
Spring, Maryland.
 
Edwards, A. W. F., and L. L. Cavalli-Sforza.  1964.  Reconstruction of
evolutionary trees.  pp. 67-76 in  Phenetic and Phylogenetic
Classification,  ed. V. H. Heywood and J. McNeill. Systematics
Association Volume No. 6. Systematics Association, London.
 
Estabrook, G. F., C. S. Johnson, Jr., and F. R. McMorris.  1976a.  A
mathematical foundation for the analysis of character
compatibility.   Mathematical Biosciences   23:  181-187.
 
Estabrook, G. F., C. S. Johnson, Jr., and F. R. McMorris.  1976b.  An
algebraic analysis of cladistic characters.   Discrete Mathematics   16:  141-147.
 
Estabrook, G. F., F. R. McMorris, and C. A. Meacham.  1985.  Comparison of
undirected phylogenetic trees based on subtrees of four evolutionary units.
 Systematic Zoology   34:  193-200.
 
Faith, D. P.  1990.  Chance marsupial relationships.   Nature  345:  393-394.
 
Faith, D. P. and P. S. Cranston.  1991.  Could a cladogram this short have
arisen by chance alone?: On permutation tests for cladistic
structure.   Cladistics   7:  1-28.
 
Farris, J. S.  1977.  Phylogenetic analysis under Dollo's Law.   Systematic Zoology   26:  77-88.
 
Farris, J. S.  1978a.  Inferring phylogenetic trees from chromosome
inversion data.   Systematic Zoology   27:  275-284.
 
Farris, J. S.  1981.  Distance data in phylogenetic analysis.  pp. 3-23
in  Advances in Cladistics: Proceedings of the first meeting of the
Willi Hennig Society,  ed. V. A. Funk and D. R. Brooks.  New York
Botanical Garden, Bronx, New York.
 
Farris, J. S.  1983.  The logical basis of phylogenetic analysis.  pp. 1-47
in  Advances in Cladistics, Volume 2, Proceedings of the Second Meeting of
the Willi Hennig Society.   ed. Norman I. Platnick and V. A. Funk.  Columbia
University Press, New York.
 
Farris, J. S.  1985.  Distance data revisited.   Cladistics   1:  67-85.
 
Farris, J. S.  1986.  Distances and statistics.   Cladistics   2:  144-157. 
 
Farris, J. S. ["T. N. Nayenizgani"].  1990.  The systematics association
enters its golden years (review of  Prospects in Systematics , ed. D.
Hawksworth).   Cladistics   6:  307-314.
 
Felsenstein, J.  1973a.  Maximum likelihood and minimum-steps methods
for estimating evolutionary trees from data on discrete characters.
 Systematic Zoology   22:  240-249.
 
Felsenstein, J.  1973b.  Maximum-likelihood estimation of evolutionary
trees from continuous characters.   American Journal of Human Genetics   25: 
471-492.
 
Felsenstein, J.  1978a.  The number of evolutionary trees.   Systematic Zoology   27:  27-33.
 
Felsenstein, J.  1978b.  Cases in which parsimony and compatibility
methods will be positively misleading.   Systematic Zoology   27: 
401-410.
 
Felsenstein, J.  1979.  Alternative methods of phylogenetic inference
and their interrelationship.   Systematic Zoology   28:  49-62.
 
Felsenstein, J.  1981a.  Evolutionary trees from DNA sequences: a
maximum likelihood approach.   Journal of Molecular Evolution   17:  368-376.
 
Felsenstein, J.  1981b.  A likelihood approach to character weighting
and what it tells us about parsimony and compatibility.   Biological Journal of the Linnean Society   16:  183-196.
 
Felsenstein, J.  1981c.  Evolutionary trees from gene frequencies and
quantitative characters: finding maximum likelihood estimates.
 Evolution   35:  1229-1242.
 
Felsenstein, J.  1982.  Numerical methods for inferring evolutionary
trees.   Quarterly Review of Biology   57:  379-404.
 
Felsenstein, J.  1983b.  Parsimony in systematics: biological and
statistical issues.  Annual Review of Ecology and Systematics   14:  313-333.
 
Felsenstein, J. 1984a.  Distance methods for inferring phylogenies: a
justification.  Evolution   38:  16-24.
 
Felsenstein, J.  1984b.  The statistical approach to inferring
evolutionary trees and what it tells us about parsimony and
compatibility.  pp. 169-191 in:  Cladistics: Perspectives in the
Reconstruction of Evolutionary History,  edited by T. Duncan and T. F.
Stuessy.  Columbia University Press, New York.
 
Felsenstein, J.  1985a.  Confidence limits on phylogenies with a molecular
clock.   Systematic Zoology   34:  152-161.
 
Felsenstein, J.  1985b.  Confidence limits on phylogenies: an approach
using the bootstrap.   Evolution   39:  783-791. 
 
Felsenstein, J.  1985c.  Phylogenies from gene frequencies: a statistical
problem.   Systematic Zoology   34:  300-311.
 
Felsenstein, J.  1985d.  Phylogenies and the comparative method.   American Naturalist   125:  1-12.
 
Felsenstein, J.  1986.  Distance methods: a reply to Farris.   Cladistics   2: 
130-144.
 
Felsenstein, J.  and E. Sober.  1986.  Parsimony and likelihood: an
exchange.   Systematic Zoology   35:  617-626.
 
Felsenstein, J.  1988a.  Phylogenies and quantitative characters.   Annual Review of Ecology and Systematics   19:  445-471.
 
Felsenstein, J.  1988b.  Phylogenies from molecular sequences: inference and 
reliability.    Annual Review of Genetics   22:  521-565.
 
Felsenstein, J.  1992.  Phylogenies from restriction sites, a
maximum likelihood approach.   Evolution   46:  159-173.
 
Felsenstein, J. and G. A. Churchill. 1996.
A hidden Markov model approach to variation among sites in rate of evolution
 Molecular Biology and Evolution   13:  93-104.
 
Felsenstein, J. 2004.  Inferring Phylogenies.  Sinauer Associates,
Sunderland, Massachusetts.
 
Fink, W. L.  1986.  Microcomputers and phylogenetic analysis.   Science   234:  1135-1139.
 
Fitch, W. M., and E. Markowitz.  1970.  An improved method for determining
codon variability in a gene and its application to the rate of fixation of
mutations in evolution.   Biochemical Genetics   4:  579-593.
 
Fitch, W. M., and E. Margoliash.  1967.  Construction of phylogenetic
trees.   Science   155:  279-284.
 
Fitch, W. M.  1971.  Toward defining the course of evolution: minimum
change for a specified tree topology.   Systematic Zoology   20:  406-416.
 
Fitch, W. M.  1975.  Toward finding the tree of maximum parsimony.  pp. 189-230
in  Proceedings of the Eighth International Conference on Numerical Taxonomy, 
ed. G. F. Estabrook.  W. H. Freeman, San Francisco.
 
Fitch, W. M. and E. Markowitz.  1970.  An improved method for determining
codon variability and its application to the rate of fixation of mutations
in evolution.   Biochemical Genetics   4:  579-593.
 
George, D. G.,  L. T. Hunt, and W. C. Barker.  1988.  Current methods in
sequence comparison and analysis.  pp. 127-149 in Macromolecular Sequencing
and Synthesis, ed. D. H. Schlesinger.  Alan R. Liss, New York.
 
Gilmour, R.  2000.  Taxonomic markup language: applying XML to systematic data.
 Bioinformatics   16:  406-407.
 
Gomberg, D.  1966.  "Bayesian" post-diction in an evolution process.
unpublished manuscript: University of Pavia, Italy.
 
Graham, R. L., and L. R. Foulds.  1982.  Unlikelihood that minimal
phylogenies for a realistic biological study can be constructed in
reasonable computational time.   Mathematical Biosciences   60:  133-142.
 
Hasegawa, M. and T. Yano.  1984a.  Maximum likelihood method of phylogenetic
inference from DNA sequence data.   Bulletin of the Biometric Society of Japan   No. 5:  1-7.
 
Hasegawa, M.  and T. Yano.  1984b.  Phylogeny and classification of
Hominoidea as inferred from DNA sequence data.   Proceedings of the Japan Academy   60 B:  389-392.
 
Hasegawa, M., Y. Iida, T. Yano, F. Takaiwa, and M. Iwabuchi.  1985a.
Phylogenetic relationships among eukaryotic kingdoms as inferred from
ribosomal RNA sequences.  Journal of Molecular Evolution  22: 32-38.
 
Hasegawa, M., H. Kishino, and T. Yano.  1985b.  Dating of the human-ape
splitting by a molecular clock of mitochondrial DNA.  Journal of Molecular
Evolution  22: 160-174.
 
Hendy, M. D., and D. Penny.  1982.  Branch and bound algorithms to
determine minimal evolutionary trees.   Mathematical Biosciences   59:  277-290.
 
Higgins, D. G. and P. M. Sharp.  1989.  Fast and sensitive
multiple sequence alignments on a microcomputer.   Computer Applications in the Biological Sciences (CABIOS)   5:  151-153.
 
Hochbaum, D. S. and A. Pathria.  1997.  Path costs in evolutionary
tree reconstruction.   Journal of Computational Biology   4:  163-175.
 
Holmquist, R., M. M. Miyamoto, and M. Goodman.  1988.  Higher-primate
phylogeny - why can't we decide?   Molecular Biology and Evolution   5:  201-216.
 
Inger, R. F.  1967.  The development of a phylogeny of frogs. 
 Evolution   21:  369-384.
 
Jin, L. and M. Nei.  1990.  Limitations of the evolutionary parsimony method
of phylogenetic analysis.   Molecular Biology and Evolution   7:  82-102.
 
Jones, D. T., W. R. Taylor and J. M. Thornton. 1992. The rapid generation of
mutation data matrices from protein sequences.  Computer Applications
in the Biosciences (CABIOS)   8:  275-282.
 
Jukes, T. H. and C. R. Cantor.  1969.  Evolution of protein molecules.  pp. 
21-132 in Mammalian Protein Metabolism, ed. H. N. Munro.  Academic Press, New 
York.
 
Kidd, K. K. and L. A. Sgaramella-Zonta.  1971.  Phylogenetic analysis: concepts
and methods.   American Journal of Human Genetics   23:  235-252.
 
Kim, J.  and M. A. Burgman.  1988.  Accuracy of phylogenetic-estimation 
methods using simulated allele-frequency data.   Evolution   42:  596-602.
 
Kimura, M.  1980.  A simple model for estimating evolutionary rates of base 
substitutions through comparative studies of nucleotide sequences.   Journal of Molecular Evolution   16:  111-120.
 
Kimura, M.  1983.  The Neutral Theory of Molecular Evolution.  Cambridge
University Press, Cambridge.
 
Kingman, J. F. C.  1982a.  The coalescent.   Stochastic Processes and Their Applications   13:  235-248.
 
Kingman, J. F. C.  1982b.  On the genealogy of large populations.   Journal of Applied Probability   19A:  27-43.
 
Kishino, H. and M. Hasegawa.  1989. Evaluation of the maximum likelihood
estimate of the evolutionary tree topologies from DNA sequence data, and the
branching order in Hominoidea.   Journal of Molecular Evolution   29:  170-179.
 
Kluge, A. G., and J. S. Farris.  1969.  Quantitative phyletics and the
evolution of anurans.   Systematic Zoology   18:  1-32.
 
Kosiol, C., and N. Goldman.  2005.  Different versions of the Dayhoff rate
matrix.   Molecular Biology and Evolution   22:  193-199.
 
Kuhner, M. K. and J. Felsenstein.  1994.  A simulation comparison of
phylogeny algorithms under equal and unequal evolutionary rates.
 Molecular Biology and Evolution   11:  459-468 (Erratum  12:  525  1995).
 
Künsch, H. R.  1989.  The jackknife and the bootstrap for general stationary
observations.   Annals of Statistics   17:  1217-1241.
 
Lake, J. A.  1987.  A rate-independent technique for analysis of nucleic acid
sequences: evolutionary parsimony.   Molecular Biology and Evolution   4:  167-191.
 
Lake, J. A.  1994.  Reconstructing evolutionary trees from DNA and protein
sequences: paralinear distances.
 Proceedings of the Natonal Academy of Sciences, USA   91:  1455-1459.
 
Le Quesne, W. J.  1969.  A method of selection of characters in
numerical taxonomy.   Systematic Zoology   18:  201-205.
 
Le Quesne, W. J.  1974.  The uniquely evolved character concept and its
cladistic application.   Systematic Zoology   23:  513-517.
 
Lewis, H. R., and C. H. Papadimitriou.  1978.  The efficiency of
algorithms.   Scientific American   238:  96-109 (January issue)
 
Lockhart, P. J., M. A. Steel, M. D. Hendy, and D. Penny.  1994.
Recovering evolutionary trees under a more realistic model of sequence
evolution.   Molecular Biology and Evolution   11:  605-612.
 
Luckow, M.  and D. Pimentel.  1985.  An empirical comparison of
numerical Wagner computer programs.   Cladistics   1:  47-66.
 
Lynch, M.  1990.  Methods for the analysis of comparative data in evolutionary
biology.   Evolution   45:  1065-1080.
 
Maddison, D. R.  1991.  The discovery and importance of multiple islands of
most-parsimonious trees.   Systematic Zoology   40:  315-328.
 
Margush, T. and F. R. McMorris.  1981.  Consensus n-trees.   Bulletin of Mathematical Biology   43:  239-244.
 
Nelson, G.  1979.  Cladistic analysis and synthesis: principles and definitions,
with a historical note on Adanson's  Familles des Plantes 
(1763-1764).   Systematic Zoology   28:  1-21.
 
Nei, M.  1972.  Genetic distance between populations.   American Naturalist   106:  283-292.
 
Nei, M.  and W.-H. Li.  1979.  Mathematical model for studying genetic variation
in terms of restriction endonucleases.   Proceedings of the National Academy of Sciences, USA   76:  5269-5273.
 
Page, R. D. M.  1989.  Comments on component-compatibility in historical
biogeography.   Cladistics   5:  167-182.
 
Penny, D. and M. D. Hendy.  1985.  Testing methods of evolutionary tree 
construction.   Cladistics   1:  266-278.
 
Platnick, N.  1987.   An empirical comparison of microcomputer parsimony
programs.   Cladistics   3:  121-144.
 
Platnick, N.  1989.  An empirical comparison of microcomputer parsimony
programs. II.   Cladistics   5:  145-161.
 
Reynolds, J. B., B. S. Weir, and C. C. Cockerham.  1983.  Estimation of the 
coancestry coefficient: basis for a short-term genetic 
distance.   Genetics   105:  767-779.
 
Robinson, D. F. and L. R. Foulds.  1979.  Comparison of weighted
labelled trees.  pp. 119-126 in  Combinatorial Mathematics VI.  Proceedings
of the Sixth Australian Conference on Combinatorial Mathematics, Armidale,
Australia, August, 1978,   ed. A. F. Horadam and W. D. Wallis.  Lecture Notes in Mathematics, No. 748.  Springer-Verlag, Berlin.
 
Robinson, D. F. and L. R. Foulds.  1981.  Comparison of phylogenetic trees.
 Mathematical Biosciences   53:  131-147.
 
Rohlf, F. J.  and M. C. Wooten.  1988.  Evaluation of the restricted maximum 
likelihood method for estimating phylogenetic trees using simulated allele-
frequency data.   Evolution   42:  581-595.
 
Rzhetsky, A., and M. Nei.  1992.  Statistical properties of the ordinary
least-squares, generalized least-squares, and minimum-evolution methods
of phylogenetic inference.  Journal of Molecular Evolution   35: 
367-375 .
 
Saitou, N., and M. Nei.  1987.  The neighbor-joining method: a new method for
reconstructing phylogenetic trees.   Molecular Biology and Evolution   4:  406-425.
 
Sanderson, M. J.  1990.  Flexible phylogeny reconstruction: a review of
phylogenetic inference packages using parsimony.   Systematic Zoology   39:  414-420.
 
Sankoff, D. D., C. Morel, R. J. Cedergren.  1973.  Evolution of 5S RNA and
the nonrandomness of base replacement.   Nature New Biology   245:  232-234.
 
Shimodaira, H. and M. Hasegawa.  1999.  Multiple comparisons of log-likelihoods
with applications to phylogenetic inference.   Molecular Biology and
Evolution   16:  1114-1116.
 
Shimodaira, H.  2002.  An approximately unbiased test of phylogenetic
tree selection.   Systematic Biology   51:  492--508.
 
Sokal, R. R. and P. H. A. Sneath.  1963.   Principles of Numerical Taxonomy. 
W. H. Freeman, San Francisco.
 
Smouse, P. E. and W.-H. Li.  1987.  Likelihood analysis of mitochondrial
restriction-cleavage patterns for the human-chimpanzee-gorilla trichotomy.
 Evolution   41:  1162-1176.
 
Sober, E.  1983a.  Parsimony in systematics: philosophical issues.   Annual Review of Ecology and Systematics   14:  335-357.
 
Sober, E.  1983b.  A likelihood justification of parsimony.   Cladistics   1:  209-233.
 
Sober, E.  1988.   Reconstructing the Past: Parsimony, Evolution,
and Inference.   MIT Press, Cambridge, Massachusetts.
 
Sokal, R. R., and P. H. A. Sneath.  1963.   Principles of Numerical
Taxonomy.   W. H. Freeman, San Francisco.
 
Steel, M. A., P. J. Lockhart, and D. Penny.  1993.  Confidence in evolutionary trees from biological sequence data.   Nature   364:  440-442.
 
Steel, M. A.  1994.  Recovering a tree from the Markov leaf colourations
it generates under a Markov model.   Applied Mathematics Letters 
 7:  19-23.
 
Studier, J. A.  and K. J. Keppler.  1988.  A note on the neighbor-joining
algorithm of Saitou and Nei.   Molecular Biology and Evolution  5:  729-731.
 
Swofford, D. L. and G. J. Olsen.  1990.  Phylogeny reconstruction.  Chapter
11, pages 411-501 in  Molecular Systematics,  ed. D. M. Hillis and C. Moritz.
Sinauer Associates, Sunderland, Massachusetts.
 
Swofford, D. L., G. J. Olsen, P. J. Waddell, and D. M. Hillis.  1996.
Phylogenetic inference.  pp. 407-514 in  Molecular Systematics , 2nd ed.,
ed.  D. M. Hillis, C. Moritz, and B. K. Mable.  Sinauer Associates, Sunderland,
Massachusetts.
 
Templeton, A. R.  1983.  Phylogenetic inference from restriction endonuclease
cleavage site maps with particular reference to the evolution of humans and the
apes.  Evolution   37:  221-244.
 
Thompson, E. A.  1975.   Human Evolutionary Trees.   Cambridge University
Press, Cambridge.
 
Veerassamy, S., A. Smith and E. R. M. Tillier.  2003.
A transition probability model for amino acid substitutions from Blocks.
 Journal of Computational Biology   10:  997-1010.
 
Wu, C. F. J.  1986.  Jackknife, bootstrap and other resampling plans in 
regression analysis.   Annals of Statistics   14:  1261-1295.
 
Yang, Z. 1993.  Maximum-likelihood estimation of phylogeny from DNA sequences
when substitution rates differ over sites.   Molecular Biology and
Evolution   10:  1396-1401.
 
Yang, Z. 1994.  Maximum likelihood phylogenetic estimation from DNA sequences
with variable rates over sites: approximate methods.   Journal of Molecular
Evolution   39:  306-314.
 
Yang, Z.  1995.  A space-time process model for the evolution of DNA sequences.
 Genetics   139:  993-1005.
 
Zharkikh, A. and W.-H. Li.  1995.  Estimation of confidence in phylogeny:
the complete-and-partial bootstrap technique.   Molecular Biology and
Evolution   4:  44-63.

 
 
    
 Credits  
 
Over the years various granting agencies have contributed to the
support of the PHYLIP project (at first without knowing it).  They are:
 
 
  Years 
 Agency 
 Grant or Contract Number 
 
  1999-2003 
 NSF 
 BIR-9527687 
 
  1999-2002 
 NIH NIGMS 
 R01 GM51929-04 
 
  1999-2001 
 NIH NIMH 
 R01 HG01989-01 
 
  1995-1999 
 NIH NIGMS 
 R01 GM51929-01 
 
  1992-1995  
 National Science Foundation 
 DEB-9207558 
 
  1992-1994 
 NIH NIGMS Shannon Award 
 2 R55 GM41716-04 
 
  
1989-1992 
 NIH NIGMS 
 1 R01-GM41716-01 
 
  
1990-1992 
 National Science Foundation 
 BSR-8918333 
 
  
1987-1990 
 National Science Foundation 
 BSR-8614807 
 
  1979-1987 
 U.S. Department of Energy 
 DE-AM06-76RLO2225 TA DE-AT06-76EV71005 
 
 
 
I am particularly grateful to program administrators William Moore,
Irene Eckstrand, Peter Arzberger, and Conrad Istock, who have
gone beyond the call of duty to make sure that PHYLIP continued.
 
Booby prizes for funding are awarded to:
  The people at the U.S. Department of Energy who, in 1987, decided they
were "not interested in phylogenies",
 The members of the Systematics Panel of NSF who twice (in 1989 and 1992)
positively recommended that my applications  not  be funded.  I am very
grateful to program director William Moore for courageously overruling
their decision the first time.  The 1992 NSF Systematics Panel could claim
no credit for PHYLIP whatsoever.
 The members of the 1992 Genetics Study Section of NIH who rated my
proposal in the 53rd percentile (I don't know if that's 53rd from
the top or the bottom, but does it matter?), thus denying it funding.  I am,
however, grateful to the NIGMS administrators, especially Irene Eckstrand,
who supported giving me
a "Shannon award" partially funding my work for a period in spite of this
rating.
 The reviewers at NSF Population Biology in early 2003 who gave my 
proposal too anemic a rating: ( "The work has the potential to define the field for many years to come .... All agreed that the proposal is somewhat vague. There was also some concern that the proposed work is too ambitious." ) 
 The reviewers at the Genetics Study Section of the NIH in early 2004
who said such nice things about my proposal ( "One is likely to look back
in 20 years and marvel that these questions were not actively pursued by more
theoreticians"  and  "The first inclination is to give this PI
all the money he wants!" ), but then
proceeded to give my proposal a 30.2 percentile rating.  Next time you can
tone down the praise as long as you improve the ratings, OK?
 
 
The original Camin-Sokal parsimony program and the polymorphism parsimony 
program were written by me in 1977 and 1978.  They were Pascal versions of 
earlier FORTRAN programs I wrote in 1966 and 1967 using the same algorithm to 
infer phylogenies under the Camin-Sokal and polymorphism parsimony 
criteria.  Harvey Motulsky worked for me as a programmer in 1971 and wrote 
FORTRAN programs to carry out the Camin-Sokal, Dollo, and polymorphism 
methods (he is better-known these days as the author of the scientific
data analysis package GraphPad).  But most of the early work on PHYLIP other
than my own was by Jerry 
Shurman and Mark Moehring.  Jerry Shurman worked for me in the summers of 
1979 and 1980, and Mark Moehring worked for me in the summers of 1980 and 
1981.  Both wrote original versions of many of the other programs, based on 
the original versions of my Camin-Sokal parsimony program and POLYM.  These 
formed the basis of Version 1 of the Package, first distributed in October, 
1980. 
 
Version 2, released in the spring of 1982, involved a fairly complete rewrite 
by me of many of those programs.  Hisashi Horino for
version 3.3 reworked some parts of the programs Clique and Consense
to make their output more comprehensible, and has added some code to the
tree-drawing programs Drawgram and Drawtree as well.  He also worked on
some of the Drawtree and Drawgram driver code.
 
My more recent part-time programmers Akiko Fuseki, Sean Lamont,
Andrew Keeffe, Daniel Yek, Dan Fineman, Patrick Colacurcio,
Mike Palczewski, Doug Buxton, Ian Robertson, and Marissa LaMadrid gave
me substantial help with the 3.6 releases, and their excellent work is
greatly appreciated.  Akiko, in over 10 years of excellent work, did much of the hard work of adding
new features and changing old ones in the 3.4 and 3.5 releases,
centralized many of the C routines in support files, and is responsible for the
new versions of Dnapars and Pars. Andrew
prepared the Macintosh version, wrote Retree, added the ray-tracing
and PICT code to the Draw... programs and has since done much other work.  Sean
was central to the conversion to
C, and tested it extensively.  My (then) postdoctoral fellow
Mary Kuhner and her associate Jon Yamato created Neighbor, the
neighbor-joining and UPGMA program, for the current release, for which I am
also grateful (Naruya Saitou and Li Jin kindly encouraged us to use some of the
code from their own implementation of this method).
 
I am very grateful to over 300
users for algorithmic suggestions, complaints about features (or lack of
features), and information about the behavior of their operating systems
and compilers.  A list of some of their names will be found at
 the
credits page on the PHYLIP web site  which is at
 http://evolution.gs.washington.edu/phylip/credits.html 
 
A major contribution to this package has been made by others
writing programs or parts of programs.  Chris Meacham contributed the
important program Factor, long demanded by users, and the even more
important ones PLOTREE and PLOTGRAM.  Important parts of the code in
Drawgram and Drawtree were taken over from those two programs.
Kent Fiala wrote
function "reroot" to do outgroup-rooting, which was an essential part of many
programs in earlier versions.  Someone at the Western Australia Institute of
Technology suggested the name PHYLIP (by writing it the label on the
outside of a magnetic tape).  Perhaps it was the late Julian Ford
(I've lost the relevant letter).
 
The distribution of the package also owes much to Buz Wilson and Willem Ellis, 
who put a lot of effort into the early distributions of the PCDOS and
Macintosh versions respectively.  Christopher Meacham and Tom Duncan for three
versions distributed a printed version of these documentation files (they are no
longer able to do so), and I am
very grateful to them for those efforts.  William H.E. Day and F. James Rohlf
have been very helpful in setting up the listserver news bulletin service which
succeeded the PHYLIP newsletter for a time.
 
I also wish to thank the people who have made computer resources available to
me, mostly in the loan of use of microcomputers.  These include Jeremy
Field, Clem Furlong, Rick Garber, Dan Jacobson, Rochelle Kochin, Monty Slatkin,
Jim Archie, Jim Thomas, and George Gilchrist.
 
I should also note the computers used to develop this package:
These include a CDC 6400, two DECSystem 1090s, my trusty old SOL-20, my
old Osborne-1, a VAX 11/780, a VAX 8600, a MicroVAX I, a DECstation
3100, my old Toshiba 1100+, my 
DECstation 5000/200, a DECstation 5000/125, a Compudyne 486DX/33, a
Trinity Genesis 386SX, a Zenith Z386, a Mac Classic, a DEC Alphastation 400
4/233, a Pentium 120, a Pentium 200, a PowerMac 6100, and a Macintosh G3.
(One of the reasons
we have been successful in achieving compatibility between different computer
systems is that I have had to run them myself under so many different operating
systems and compilers).
 
    
 
 Other Phylogeny Programs Available Elsewhere  
 
A comprehensive list of phylogeny programs is maintained at the PHYLIP
web site on the Phylogeny Programs pages:
 
 
  
 http://evolution.gs.washington.edu/phylip/software.html    
 
Here we will simply mention some of the major general-purpose programs.  For
many more and much more, see those web pages.
 
 PAUP*     A comprehensive program with parsimony, likelihood, and
distance matrix methods.  It competes with PHYLIP to be responsible for
the most trees published.  Written by David Swofford and distributed by
Sinauer Associates of Sunderland, Massachusetts.
It is described in
 a web page .
at  http://www.sinauer.com/detail.php?id=8060 .
Current prices are $100 for the Macintosh version, $85 for the
Windows version, and $150 for Unix versions for many kinds of workstations.
 
 MacClade      An interactive Macintosh program to
rearrange trees and watch the changes in the fit of the trees to
data as judged by parsimony.  MacClade has a great many features including
a spreadsheet data editor and many different descriptive statistics
for different kinds of data.  It is particularly designed to export and
import data to and from PAUP*.
MacClade is available for $100 from Sinauer Associates, of Sunderland,
Massachusetts.  It is described in a web page at
 
 http://www.sinauer.com/detail.php?id=4707  .
MacClade is also described on its  
Web page , at  http://phylogeny.arizona.edu/macclade/macclade.html .
 
 MrBayes    The leading program for Bayesian inference of
phylogenies.  It uses Markov Chain Monte Carlo inference to assess
support for clades and to infer posterior distrubutions of parameters.
Produced by John Huelsenbeck and Fredrik Ronquist, it is available at
 its web site  at
 http://morphbank.ebc.uu.se/mrbayes/  as a Macintosh or Windows
executable, or in source code in C.
 
 MEGA     A Windows and DOS program by Sudhir Kumar of Arizona State University
(written together with Koichiro Tamura and Masatoshi Nei while he was a
student in Nei's lab at Pennsylvania
State University).  It can carry out parsimony and distance matrix methods
for DNA sequence data.  Version 2.1 for Windows
can be downloaded from  
the MEGA web site 
at  http://www.megasoftware.net .
 
 PAML     Ziheng Yang of the Department of Genetics and Biometry at
University College, London has written this package of programs to
carry out likelihood analysis of DNA and protein sequence data.  
It is one of the only packages able to use the codon model for protein
sequence data which takes the genetic code reasonably fully into account.
PAML is
particularly strong in the options for coping with variability of rates
of evolution from site to site, though it is less able than some other
packages to search effectively for the best tree.  It is available as
C source code and as Macintosh and Windows executables from its web site at
 
 http://abacus.gene.ucl.ac.uk/software/paml.html  .
 
 TREE-PUZZLE     This package by Korbinian Strimmer and Arndt von Haeseler
was begun when they were at the Uviversität Munchen in Germany.
TREE-PUZZLE can carry out likelihood
methods for DNA and protein data, searching by the strategy of
"quartet puzzling" which they invented.  It can also compute distances.
It superimposes trees estimated
from many quartets of species.  TREE-PUZZLE is available for Unix, Macintoshes,
or Windows from their web site at
  http://www.tree-puzzle.de/  .
 
 DAMBE     A package written by Xuhua Xia of the
Department of Biology of the University of Ottawa (formerly of the
University of Hong Kong).
Its initials stand for Data Analysis in Molecular Biology and Evolution.
DAMBE is a general-purpose package for DNA and protein sequence phylogenies.
It can read and
convert a number of file formats, and has many features for
descriptive statistics, and can compute a number of commonly-used
distance matrix measures and infer phylogenies by parsimony, distance,
or likelihood methods, including bootstrapping and jackknifing.  There are
a number of kinds of statistical tests of trees available and it
can also display phylogenies.  DAMBE includes a copy of ClustalW as well;
DAMBE consists of Windows executables.  It is available from its
web site at  
 http://aix1.uottawa.ca/~xxia/software/software.htm  .
 
 Hennig86     A fast parsimony program by J. S. Farris of the
Naturhistoriska Riksmuseet in Stockholm, Sweden for discrete characters
data (it can handle DNA if its states are recoded to be digits).
Reputed to be faster than PAUP*.
The program is distributed as an executable and costs $50, plus $5
mailing costs ($10 outside of of the U.S.). The user's name should be stated,
as copies are personalized as a copy-protection measure. It is
distributed by Arnold Kluge, Amphibians and Reptiles, Museum of Zoology,
University of
Michigan, Ann Arbor, Michigan 48109-1079, U.S.A. ( akluge  (at)  umich.edu ) and
by Diana Lipscomb at George Washington University ( BIODL  (at)  gwuvm.gwu.edu ).
 
 RnA     J. S. Farris's very fast program which uses parsimony
to carry out jackknifing resampling of DNA sequence data.  This would be
nearly equivalent in properties to bootstrapping if the jackknifing were
sampling random halves of the data, but Farris prefers to have each
jackknife sample delete a fraction 1/ e  of the data, which will give
most groups too much support (he would disagree with this statement).
RnA is available from Arnold Kluge, Amphibians and Reptiles, Museum of Zoology,
University of
Michigan, Ann Arbor, Michigan 48109-1079, U.S.A. ( akluge  (at)  umich.edu )
and Diana Lipscomb
at George Washington University ( BIODL  (at)  gwuvm.gwu.edu ) who may be
contacted for details.  The cost is about $30 US. 
 
 NONA     Pablo Goloboff, of the Instituto Miguel Lillo in
Tucumán, Argentina has written this very fast parsimony program, capable
of some relevant forms of weighted parsimony, which can handle either
DNA sequence data or discrete characters.  It is available with
some companion programs
from  
 http://www.cladistics.com/aboutNona.htm  
There is a 30 day free trial, after which
NONA must be purchased separately by sending a check for $40.00 to
either directly to the the author, or to: James M. Carpenter, Attn: NONA,
Division of Invertebrate Zoology, American Museum of Natural History,
Central Park West at 79th Street, New York, NY 10024.
 
 TNT    This program, by Pablo Goloboff, J. S. Farris, and Kevin Nixon,
is for searching large data sets for most parsimonious trees. 
The authors are respectively at the Instituto Miguel Lillo in Tucuman,
Argentina, the Naturhistoriska Riksmuseet in Stockholm, Sweden, and the
Hortorium, Cornell University, Ithaca, New York.
TNT is described
as faster than other methods, though not faster than NONA for small to
medium data sets.  It is distributed as
Windows, Linux, and MacOS X executables. The Windows version has a free
demonstration mode which allows up to 10 sessions, each up to 10 hours long.
To receive the password for the program and, one should send a check for $80.00
US made out to Jim Carpenter, sending it to Carpenter at Division of
Invertebrate Zoology, American Museum of Natural History, Central Park West at
79th Street, New York, NY 10024, USA. Tell him also the exact user name you
will use with the program (as the password must match an encrypted version of
this). The program and some support files including documentation are
available from  its download
area  at  http://www.zmuc.dk/public/phylogeny/tnt 
(see the ReadMe! web page there).
 
These are only a few of the over 256 different phylogeny packages that
are now available (as of July, 2006 - the number keeps increasing).  The
others are described (and web links and ftp addresses provided) at my
Phylogeny Programs web pages at the address given above.
 
    
 
 How You Can Help Me  
 
Simply let me know of any problems you have had adapting the
programs to your computer.  I can often make "transparent" changes that, by
making the code avoid the wilder, woolier, and less standard parts of
C, not only help others who have your machine but even improve the
chance of the programs functioning on new machines.  I would like fairly
detailed information on what gave trouble, on what operating system,
machine, and (if relevant) compiler, and what had to be done to make the
programs work.  I am sometimes able to do some over-the-telephone
trouble-shooting, particularly
if I don't have to pay for the call, but electronic mail is a the best
way for me to be asked about problems, as you can include your
input and output files so I can see what is going on (please do  not 
send them as Attachments, but as part of the body of a message).  I'd really
like these programs to be
able to run with only routine changes on  absolutely everything , down to
and possibly including the Amana Touchmatic Radarange Microwave Oven
which was an Intel 8080 system (in fact, early versions of this package did
run successfully on Intel 8080 systems running the CP/M operating system).
A PalmPilot version is contemplated too.
 
I would also like to know timings of programs from the package, when
run on the three test input files provided above, for various computer and
compiler combinations, so that I can provide this information in the
section on speeds of this document.
 
For the phylogeny plotting programs Drawgram and Drawtree,
I am particularly interested in knowing what has to be done
to adapt them for other graphic file formats.
 
You can also be helpful to PHYLIP users in your part of the world by
helping them get the latest version of PHYLIP from our web site
and by helping them with any
problems they may have in getting PHYLIP working on their data.
 
Your help is appreciated.  I am always happy to hear suggestions
for features and programs that ought to be incorporated in the package,
but please do not be upset if I turn out to have already considered the
particular possibility you suggest and decided against it.
 
    
 
 In Case of Trouble  
 
 Read The (documentation) Files Meticulously  ("RTFM").  If that doesn't solve the
problem, please check the Frequently Asked Questions web page at the
PHYLIP web site:
 
 
  
http://evolution.gs.washington.edu/phylip/faq.html   
 
and the PHYLIP Bugs web page at that site:
 
 
  
http://evolution.gs.washington.edu/phylip/bugs.html   
 
If none of these answers your question, get in touch with me.  My electronic mail address
is given below.  If you do ask about a problem, please specify the program
name, version of the package, computer operating system, and
send me your data file so I can test the problem.  Do  not 
send your data file as an e-mail Attachment but instead
as the body of a message. I read the e-mail on a Unix system, which makes
it impossible to read some formats of attachments without
running around to other machines and moving the files there. This
is one of my least favorite activities, so please do not use attachments.
Also it will help if you 
have the relevant output and documentation files so that you
can refer to them in any correspondence.  I can also be reached by telephone
by calling me in my office: 
+1-(206)-543-0150, or at home: +1-(206)-526-9057 (how's  that  for user
support!).  If I cannot be reached at either place, a message can be left at
the office of
the Department of Genome Sciences, +1-(206)-221-7377 but I prefer strongly that I not
call you, as in any phone consultation the least you can do is pay the phone
bill.  Better yet, use electronic mail.
 
Particularly if you are in a part of the world distant from me, you may also 
want to try to get in touch with other users of PHYLIP nearby.  I can also,
if requested, provide a list of nearby users.
 
 
   
Joe Felsenstein 
Department of Genome Sciences 
University of Washington 
Box 357730 
Seattle, Washington 98195-7730, U.S.A.
   
 
 
Electronic mail addresses:       joe  (at)  gs.washington.edu 
  
 
 
