# Supplementary material for: Probabilistic Phylogenetic Inference with Insertions and Deletions
Source: PLoS Comput Biol. 2008 Sep 19;4(9):e1000172. doi: 10.1371/journal.pcbi.1000172 (PMC2527138; doi:10.1371/journal.pcbi.1000172)
Supplement: Dataset S1 — Supplemental Material (24.89 MB GZ) [file pcbi.1000172.s001.gz › erate-supplement-R2/src/phylip3.66-erate/doc/kitsch.html]

kitsch


version 3.66

# Kitsch -- Fitch-Margoliash and Least Squares Methods with Evolutionary Clock

© Copyright 1986-2006 by the University of
Washington. Written by Joseph Felsenstein. Permission is granted to copy
this document provided that no fee is charged for it and that this copyright
notice is not removed.

This program carries out the Fitch-Margoliash and Least Squares methods,
plus a variety of others of the same family, with the assumption that all
tip species are contemporaneous, and that there is an evolutionary clock
(in effect, a molecular clock). This means that branches of the tree cannot
be of arbitrary length, but are constrained so that the total
length from the root of
the tree to any species is the same. The quantity minimized is the same
weighted sum of squares described in the Distance Matrix Methods documentation
file.

The options are set using the menu:

|  |
| --- |
| ``` Fitch-Margoliash method with contemporary tips, version 3.6  Settings for this run:   D      Method (F-M, Minimum Evolution)?  Fitch-Margoliash   U                 Search for best tree?  Yes   P                                Power?  2.00000   -      Negative branch lengths allowed?  No   L         Lower-triangular data matrix?  No   R         Upper-triangular data matrix?  No   S                        Subreplicates?  No   J     Randomize input order of species?  No. Use input order   M           Analyze multiple data sets?  No   0   Terminal type (IBM PC, ANSI, none)?  ANSI   1    Print out the data at start of run  No   2  Print indications of progress of run  Yes   3                        Print out tree  Yes   4       Write out trees onto tree file?  Yes    Y to accept these or type the letter for one to change ``` |

Most of the options are described in the Distance Matrix Programs documentation
file.

The D (methods) option allows choice between the Fitch-Margoliash
criterion and the Minimum Evolution method (Kidd and Sgaramella-Zonta, 1971;
Rzhetsky and Nei, 1993). Minimum Evolution (not to be confused with
parsimony) uses the Fitch-Margoliash criterion to fit branch lengths to each
topology, but then chooses topologies based on their total branch length
(rather than the goodness of fit sum of squares). There is no
constraint on negative branch lengths in the Minimum Evolution method;
it sometimes gives rather strange results, as it can like solutions
that have large negative branch lengths, as these reduce the total
sum of branch lengths!

Note that the User Trees (used by option U) must be
rooted trees (with a bifurcation at their base). If you take a user
tree from Fitch and try to evaluate it in Kitsch, it must first be
rooted. This can be done using Retree. Of the options
available in Fitch, the O option is
not available, as Kitsch estimates a rooted tree which cannot be
rerooted, and the G option is not
available, as global rearrangement is the default condition anyway. It
is also not possible to specify that specific branch lengths of a user tree
be retained when it is read into Kitsch, unless all of them are present. In
that case the tree should be properly clocklike. Readers who wonder why
we have not provided the feature of holding some of the user tree branch
lengths constant while iterating others are invited to tell us how they
would do it. As you consider particular possible patterns of branch
lengths you will find that the matter is not at all simple.

If you use a User Tree (option U) with branch lengths with Kitsch, and the
tree is not clocklike, when two branch lengths give conflicting positions
for a node, Kitsch will use the first of them and ignore the other. Thus
the user tree:

```
     ((A:0.1,B:0.2):0.4,(C:0.06,D:0.01):43);
```

is nonclocklike, so it will be treated as if it were actually the tree:

```
     ((A:0.1,B:0.1):0.4,(C:0.06,D:0.06):44);
```

The input is exactly the same as described in the Distance Matrix Methods
documentation file. The output is a rooted tree, together with the sum of
squares, the number of tree topologies searched, and, if the power P is at
its default value of 2.0, the Average Percent Standard Deviation is also
supplied. The lengths of the branches of the tree are given in a table,
that also shows for each branch the time at the upper end of the
branch. "Time" here really means cumulative branch length from the root, going
upwards (on the printed diagram, rightwards). For each branch, the
"time" given is for the node at the right (upper) end of the branch. It
is important to realize that the branch lengths are not exactly proportional to
the lengths drawn on the printed tree diagram! In particular, short
branches are exaggerated in the length on that diagram so that they are
more visible.

The method may be considered as providing an estimate of the
phylogeny. Alternatively, it can be considered as a phenetic clustering of
the tip species. This method minimizes an objective function, the sum of
squares,
not only setting the levels of the clusters so as to do so, but rearranging
the hierarchy of clusters to try to find alternative clusterings that
give a lower overall sum of squares. When the power option P is set to a
value of *P = 0.0*, so that we are minimizing a simple sum of squares
of the differences between the observed distance matrix and the expected one,
the method is very close in spirit to Unweighted Pair Group Arithmetic Average
Clustering (UPGMA), also called Average-Linkage Clustering. If the topology of
the tree is fixed and there turn out to be no branches of negative length, its
result should be the same as UPGMA in that case. But since it tries
alternative topologies and (unless
the N option is set) it combines nodes that otherwise could result in a reversal
of levels, it is possible for it to give a different, and better, result than
simple sequential clustering. Of course UPGMA itself is available as an
option in program Neighbor.

The U (User Tree) option requires a bifurcating tree, unlike Fitch, which
requires an unrooted tree with a trifurcation at its base. Thus the tree
shown below would be written:

     ((D,E),(C,(A,B)));

If a tree with a trifurcation at the base is by mistake fed into the U option
of Kitsch then some of its species (the entire rightmost furc, in fact) will be
ignored and too small a tree read in. This should result in an error message
and the program should stop. It is important to understand the
difference between the User Tree formats for Kitsch and Fitch. You may want
to use Retree to convert a user tree that is suitable for Fitch into one
suitable for Kitsch or vice versa.

An important use of this method will be to do a formal statistical test of
the evolutionary clock hypothesis. This can be done by comparing the sums
of squares achieved by Fitch and by Kitsch, BUT SOME CAVEATS ARE
NECESSARY. First, the assumption is that the observed distances are truly
independent, that no original data item contributes to more than one of them
(not counting the two reciprocal distances from i to j and from j to i). THIS
WILL NOT HOLD IF THE DISTANCES ARE OBTAINED FROM GENE FREQUENCIES, FROM
MORPHOLOGICAL CHARACTERS, OR FROM MOLECULAR SEQUENCES. It may be invalid even
for immunological distances and levels of DNA hybridization, provided that the
use of common standard for all members of a row or column allows an error in
the measurement of the standard to affect all these distances
simultaneously. It will also be invalid if the numbers have been collected in
experimental groups, each measured by taking differences from a common standard
which itself is measured with error. Only if the numbers in different cells
are measured from independent standards can we depend on the statistical
model. The details of the test and the assumptions are discussed in my review
paper on distance methods (Felsenstein, 1984a). For further and sometimes
irrelevant controversy on these matters see the papers by Farris (1981,
1985, 1986) and myself (Felsenstein, 1986, 1988b).

A second caveat is that the distances must be expected to rise linearly with
time, not according to any other curve. Thus it may be necessary to transform
the distances to achieve an expected linearity. If the distances have an upper
limit beyond which they could not go, this is a signal that linearity may
not hold. It is also VERY important to choose the power *P* at a value
that results in the standard deviation of the variation of the observed from the
expected distances being the *P/2*-th power of the expected distance.

To carry out the test, fit the same data with both Fitch and Kitsch,
and record the two sums of squares. If the topology has turned out the
same, we have *N = n(n-1)/2* distances which have been fit with
*2n-3*
parameters in Fitch, and with *n-1* parameters in Kitsch. Then the
difference between *S(K)* and *S(F)* has *d1 = n-2*
degrees of freedom. It is
statistically independent of the value of *S(F)*, which has
*d2 = N-(2n-3)*
degrees of freedom. The ratio of mean squares

```
      [S(K)-S(F)]/d1
     ----------------
          S(F)/d2
```

should, under the
evolutionary clock, have an F distribution with *n-2* and
*N-(2n-3)* degrees of
freedom respectively. The test desired is that the F ratio is in the upper
tail (say the upper 5%) of its distribution. If the S (subreplication)
option is in
effect, the above degrees of freedom must be modified by noting that
N is not *n(n-1)/2* but is the sum of the numbers of replicates of all
cells in the distance matrix read in, which may be either square or
triangular. A further explanation of the
statistical test of the clock is given in a paper of mine (Felsenstein, 1986).

The program uses a similar tree construction method to the other programs
in the package and, like them, is not guaranteed to give the best-fitting
tree. The assignment of the branch lengths for a given topology is a
least squares fit, subject to the constraints against negative branch lengths,
and should not be able to be improved upon. Kitsch runs more quickly than
Fitch.

The constant
available for modification at the beginning of the program is
"epsilon", which defines a small quantity needed in
some of the calculations. There is no feature saving multiply trees
tied for best,
because exact ties are not expected, except in cases where it should be
obvious from the tree printed out what is the nature of the tie (as when an
interior branch is of length zero).

---

### TEST DATA SET

|  |
| --- |
| ```     7 Bovine      0.0000  1.6866  1.7198  1.6606  1.5243  1.6043  1.5905 Mouse       1.6866  0.0000  1.5232  1.4841  1.4465  1.4389  1.4629 Gibbon      1.7198  1.5232  0.0000  0.7115  0.5958  0.6179  0.5583 Orang       1.6606  1.4841  0.7115  0.0000  0.4631  0.5061  0.4710 Gorilla     1.5243  1.4465  0.5958  0.4631  0.0000  0.3484  0.3083 Chimp       1.6043  1.4389  0.6179  0.5061  0.3484  0.0000  0.2692 Human       1.5905  1.4629  0.5583  0.4710  0.3083  0.2692  0.0000 ``` |

---

### TEST SET OUTPUT FILE (with all numerical options on)

|  |
| --- |
| ```    7 Populations  Fitch-Margoliash method with contemporary tips, version 3.66                    __ __             2                   \  \   (Obs - Exp) Sum of squares =  /_ /_  ------------                                 2                    i  j      Obs  negative branch lengths not allowed   Name                       Distances ----                       ---------  Bovine        0.00000   1.68660   1.71980   1.66060   1.52430   1.60430               1.59050 Mouse         1.68660   0.00000   1.52320   1.48410   1.44650   1.43890               1.46290 Gibbon        1.71980   1.52320   0.00000   0.71150   0.59580   0.61790               0.55830 Orang         1.66060   1.48410   0.71150   0.00000   0.46310   0.50610               0.47100 Gorilla       1.52430   1.44650   0.59580   0.46310   0.00000   0.34840               0.30830 Chimp         1.60430   1.43890   0.61790   0.50610   0.34840   0.00000               0.26920 Human         1.59050   1.46290   0.55830   0.47100   0.30830   0.26920               0.00000                                              +-------Human                                               +-6                                      +----5 +-------Chimp                                          !    !                                  +---4    +---------Gorilla                                    !   !         +------------------------3   +--------------Orang             !                        !    +----2                        +------------------Gibbon       !    !  --1    +-------------------------------------------Mouse        !    +------------------------------------------------Bovine       Sum of squares =      0.107  Average percent standard deviation =   5.16213  From     To            Length          Height ----     --            ------          ------     6   Human           0.13460         0.81285    5      6            0.02836         0.67825    6   Chimp           0.13460         0.81285    4      5            0.07638         0.64990    5   Gorilla         0.16296         0.81285    3      4            0.06639         0.57352    4   Orang           0.23933         0.81285    2      3            0.42923         0.50713    3   Gibbon          0.30572         0.81285    1      2            0.07790         0.07790    2   Mouse           0.73495         0.81285    1   Bovine          0.81285         0.81285 ``` |
