# Supplementary material for: Probabilistic Phylogenetic Inference with Insertions and Deletions
Source: PLoS Comput Biol. 2008 Sep 19;4(9):e1000172. doi: 10.1371/journal.pcbi.1000172 (PMC2527138; doi:10.1371/journal.pcbi.1000172)
Supplement: Dataset S1 — Supplemental Material (24.89 MB GZ) [file pcbi.1000172.s001.gz › erate-supplement-R2/src/phylip3.66-erate/doc/retree.html]

retree


version 3.66

# Retree -- Interactive Tree Rearrangement

© Copyright 1993-2006 by The University of
Washington. Written by Joseph Felsenstein. Permission is granted to copy
this document provided that no fee is charged for it and that this copyright
notice is not removed.

Retree is a tree editor. It reads in a tree,
or allows the user to construct one, and
displays this tree on the screen. The
user then can specify how the tree is to
be rearranged, rerooted or written out to a file.

The input trees are in one file (with default file name
intree), the
output trees are written into another (outtree). The user
can reroot, flip branches, change names of species, change or remove
branch lengths, and move around to look at various parts of the tree if it is
too large to fit on the screen. The trees can be multifurcating at any
level, although the user is warned that many PHYLIP programs still cannot
handle multifurcations above the root, or even at the root.

A major use for this program will be to change rootedness of trees so that
a rooted tree derived from one program can be fed in as an unrooted tree to
another (you are asked about this when you give the command to write out
the tree onto the tree output file). It will also be useful for specifying
the length of a branch in
a tree where you want a program like Dnaml, Dnamlk, Fitch, or Contml to
hold that branch length constant (see the L suboption of the User Tree
option in those programs. It will also be useful for changing the order
of species for purely cosmetic reasons for Drawgram and Drawtree, including
using the Midpoint method of rooting the tree. It can also be used to write out
a tree file in the Nexus format used by Paup and MacClade or in our XML tree
file format.

This program uses graphic characters that show the tree to best
advantage on some computer systems.
Its graphic characters will work best on MSDOS systems or MSDOS windows in
Windows, and to
any system whose screen or terminals emulate ANSI standard terminals
such as old Digitial VT100 terminals,
Telnet programs,
or VT100-compatible windows in the X windowing system.
For any other screen types, (such as Macintosh windows) there is a generic
option which does
not make use of screen graphics characters. The program will work well
in those cases, but the tree it displays will look a bit uglier.

As we will see below, the initial menu of the program allows you to choose
among three screen types (PCDOS, Ansi, and none).
If you want to avoid having to make this choice every time, you can change
some of the constants in the file phylip.h to have the terminal type
initialize itself in the proper way, and recompile. We have tried to
have the default values be correct for PC, Macintosh, and Unix
screens. If the setting is "none" (which is necessary on
Macintosh MacOS 9 screens), the special graphics
characters will not be used to indicate nucleotide states, but only letters
will be used for the four nucleotides. This is less easy to look at.

The constants that need attention are ANSICRT and IBMCRT.
Currently these are both set to "false" on Macintosh MacOS 9 systems,
to "true" on MacOS X and on Unix/Linux
systems, and IBMCRT is set to "true" on Windows systems. If your system
has an ANSI compatible terminal, you might want to find the
definition of ANSICRT in phylip.h and set it to "true", and
IBMCRT to "false".

The user interaction starts with the program presenting a menu. The
menu looks like this:

|  |
| --- |
| ``` Tree Rearrangement, version 3.6  Settings for this run:   U          Initial tree (arbitrary, user, specify)?  User tree from tree file   N   Format to write out trees (PHYLIP, Nexus, XML)?  PHYLIP   0                     Graphics type (IBM PC, ANSI)?  ANSI   W       Width of terminal screen, of plotting area?  80, 80   L                        Number of lines on screen?  24  Are these settings correct? (type Y or the letter for one to change) ``` |

The 0 (Graphics type) option is the usual
one and is described in the main documentation file. The U (initial tree)
option allows the user to choose whether
the initial tree is to be arbitrary, interactively specified by the user, or
read from a tree file. Typing U causes the program to change among the
three possibilities in turn. Usually we will want to use a User Tree from
a file. It requires that you have available a tree file
with the tree topology of the initial tree. If you wish to set up some other
particular tree you can either use the "specify" choice in the initial tree
option (which is somewhat clumsy to use) or rearrange a User Tree of an
arbitrary tree into the shape you want by using
the rearrangement commands given below.

The L (screen Lines) option allows the user to change the height of the
screen (in lines of characters) that is assumed to be available on the
display. This may be particularly helpful when displaying large trees
on displays that have more than 24 lines per screen, or on workstation
or X-terminal screens that can emulate the ANSI terminals with more than
24 lines.

The N (output file format) option allows the user to specify that the tree files that
are written by the program will be in one of three formats:

1. The **PHYLIP** default file format (the Newick standard) used by the
   programs in this package.- The **Nexus** format defined by
     David Swofford and by Wayne Maddison and David Maddison for their programs
     PAUP and MacClade. A tree file written in Nexus format should be directly
     readable by those programs (They also have options to read a regular
     PHYLIP tree file as well).- An **XML** tree file format which we have defined.

The XML tree file format is fairly simple. The tree file, which
may have multiple trees, is enclosed in a pair of
<PHYLOGENIES> ... </PHYLOGENIES> tags.
Each tree is included in tags
<PHYLOGENY> ... </PHYLOGENY>. Each branch of the tree is enclosed in a pair of tags
<CLADE> ... </CLADE>, which enclose the branch and all its descendants.
If the branch has a length, this is given by the LENGTH attribute of the
CLADE tag, so that the pair of tags looks like this:

```
<CLADE LENGTH="0.09362"> ... </CLADE>
```

A tip of the tree is at the end of a branch (and hence that
branch is enclosed in a pair of
<CLADE> ... </CLADE> tags). Its name is enclosed by <NAME> ... </NAME>
tags. Here is an XML tree:

|  |
| --- |
| ``` <phylogenies>   <phylogeny>     <clade>       <clade length="0.87231"><name>Mouse</name></clade>       <clade length="0.49807"><name>Bovine</name></clade>       <clade length="0.39538">         <clade length="0.25930"><name>Gibbon</name></clade>         <clade length="0.10815">           <clade length="0.24166"><name>Orang</name></clade>           <clade length="0.04405">             <clade length="0.12322"><name>Gorilla</name></clade>             <clade length="0.06026">               <clade length="0.13846"><name>Chimp</name></clade>               <clade length="0.0857"><name>Human</name></clade>             </clade>           </clade>         </clade>       </clade>     </clade>   </phylogeny> </phylogenies> ``` |

The indentation is for readability but is not part of the XML tree
standard, which ignores that kind of white space.

What programs can read an XML tree? None right now, not even PHYLIP programs!
But soon our lab's LAMARC package will have programs that can read an XML tree.
XML is rapidly becoming the standard for representing and interchanging
complex data -- it is time to have an XML tree standard. Certain extensions
are obvious (to represent the bootstrap proportion for a branch, use
BOOTP=0.83 in the CLADE tag, for example).

There are other proposals for an XML tree standard. They have
many similarities to this one, but are not identical to it. At the
moment there is no mechanism in place for deciding between them other
than seeing which get widely used. Here are links to other
proposals:

|  |  |  |
| --- | --- | --- |
| Taxonomic Markup Language | `http://www.albany.edu/~gilmr/pubxml/`.  and preprint at  `xml.coverpages.org/gilmour-TML.pdf` | published in the paper by  Ron Gilmour (2000). |
| Andrew Rambaut's  BEAST XML phylogeny format | See page 9 of PDF of BEAST manual at   `http://evolve.zoo.ox.ac.uk/beast/` | An XML format for phylogenies is sketchly described there. |
| treeml | `http://www.nomencurator.org/InfoVis2003/download/treeml.dtd`  (see also example: )   `http://www.cs.umd.edu/hcil/iv03contest/datasets/treeml-sample.xml` | Jean-Daniel Fekete's DTD  for a tree XML file |

The W (screen and window Width) option specifies the width in characters
of the area which the trees will be plotted to fit into. This is by
default 80 characters so that they will fit on a normal width terminal. The
actual width of the display on the terminal (normally 80 characters) will
be regarded as a window displaying part of the tree. Thus you could
set the "plotting area" to 132 characters, and inform the program that
the screen width is 80 characters. Then the program will display only part
of the tree at any one time. Below we will show how to move the "window"
and see other parts of the tree.

After the initial menu is displayed and the choices are made,
the program then sets up an initial tree and displays it. Below it will be a
one-line menu of possible commands. Here is what the tree and the menu
look like (this is the tree specified by the example input tree given at
the bottom of this page, as it displays when the terminal type is "none"):

|  |
| --- |
| ```                                       ,>>1:Human                                    ,>22                                 ,>21  `>>2:Chimp                                 !  !                              ,>20  `>>>>>3:Gorilla                              !  !                  ,>>>>>>>>>>19  `>>>>>>>>4:Orang                  !           !               ,>18           `>>>>>>>>>>>5:Gibbon               !  !               !  !              ,>>>>>>>>6:Barbary Ma               !  `>>>>>>>>>>>>>23               !                 !  ,>>>>>7:Crab-e. Ma      ,>>>>>>>17                 `>24      !        !                    !  ,>>8:Rhesus Mac      !        !                    `>25      !        !                       `>>9:Jpn Macaq   ,>16        !   !  !        `>>>>>>>>>>>>>>>>>>>>>>>>>10:Squir. Mon   !  !   !  !                                ,>11:Tarsier ** 7 lines below screen **  NEXT? (Options: R . U W O T F D B N H J K L C + ? X Q) (? for Help) ``` |

The tree that was read in had no branch lengths on its branches. The
absence of a branch length is indicated by drawing the branch with ">"
characters (>>>>>>>). When branches have branch lengths, they are
drawn with "-" characters (-------) and their
lengths on the screen are approximately proportional to the branch length.

If you type "?" you will get a single screen showing a description of each
of these commands in a few words. Here are slightly more detailed
descriptions of the commands:

R: ("Rearrange"). This command asks for the number of a node which is to be removed from the tree. It and everything to the right of it on the tree is to be removed (by breaking the branch immediately below it). (This is also everything "above" it on the tree when the tree grows upwards, but as the tree grows from left to right on the screen we use "right" rather than "above"). The command also asks whether that branch is to be inserted At a node or Before a node. The first will insert it as an additional branch coming out of an existing node (creating a more multifurcating tree), and the second will insert it so that a new internal node is created in the tree, located in the branch that precedes the node (to the left of it), with the branch that is inserted coming off from that new node. In both cases the program asks you for the number of a node at (or before) which that group is to be inserted. If an impossible number is given, the program refuses to carry out the rearrangement and asks for a new command. The rearranged tree is displayed: it will often have a different number of steps than the original. If you wish to undo a rearrangement, use the Undo command, for which see below. .: (dot) This command simply causes the current tree to be redisplayed. It is of use when the tree has partly disappeared off of the top of the screen owing to too many responses to commands being printed out at the bottom of the screen. =: (toggle display of branch lengths). This option is available whenever the tree has a full set of branch lengths. It toggles on and off whether the tree displayed on the screen is shown with the relative branch lengths roughly correct. (It cannot be better than roughly correct because the display is in units of length of whole character widths on the screen). It does not actually remove any branch lengths from the tree: if the tree showing on the screen seems to have no branch lengths after use of the "=" option, if it were written out at that point, it would still have a full] set of branch lengths. U: ("Undo"). This command reverses the effect of the most recent rearrangement, outgroup re-rooting, or flipping of branches. It returns to the previous tree topology. It will be of great use when rearranging the tree and when one -- it permits you to abandon the new one and return to the previous one without remembering its topology in detail. Some operations, such as the simultaneous removal of lengths from all branches, cannot be reversed. W: ("Write"). This command writes out the current tree onto a tree output file. If the file already has been written to by this run of Retree, it will ask you whether you want to replace the contents of the file, add the tree to the end of the file, or not write out the tree to the file. It will also ask you whether you want the tree to written out as Rooted or Unrooted. If you choose Unrooted, it will write the outermost split of the tree as a three-way split with the three branches being those that issue from one of the nodes. This node will be the left (upper) interior node which is next to the root, or the other one if there is no interior node to the left (above) the root. The tree is written in the standard format used by PHYLIP (a subset of the Newick standard), in the Nexus format, or in an XML tree file format. A normal PHYLIP tree is in the proper format to serve as the User-Defined Tree for setting up the initial tree in a subsequent run of the program. However, some programs also require a line in the tree input file that gives the number of trees in the file. You may have to add this line using an editor such as vi, Emacs, Windows Notepad, or MacOS's Simpletext. O: ("Outgroup"). This asks for the number of a node which is to be the outgroup. The tree will be redisplayed with that node as the left descendant of the root fork. Note that it is possible to use this to make a multi-species group the outgroup (i.e., you can give the number of an interior node of the tree as the outgroup, and the program will re-root the tree properly with that on the left of the bottom fork. M: ("Midpoint root"). This reroots a tree that has a complete set of branches using the Midpoint rooting method. That rooting method finds the centroid of the tree -- the point that is equidistant from the two farthest points of the tree, and roots the tree there. This is the point in the middle of the longest path from one tip to another in the tree. This has the effect of making the two farthest tips stick out an equal distance to the right. Note that as the tree is rerooted, the scale may change on the screen so that it looks like it ahas suddenly gotted a bit longer. It will not have actually changed in total length. This option is not in the menu if the tree does not have a full set of branch lengths. T: ("Transpose"). This asks for a node number and then flips the two branches at that node, so that the left-right order of branches at that node is changed. This also does not actually change the tree topology but it does change the appearance of the tree. However, unlike the F option discussed below, the individual subtrees defined by those branches do not have the order of any branches reversed in them. F: ("Flip"). This asks for a node number and then flips the entire subtree at that node, so that the left-right order of branches in the whole subtree is changed. This does not actually change the tree topology but it does change the appearance of the tree. Note that it works differently than the F option in the programs Move, Dnamove, and Dolmove, which is actually like the T option mentioned above. B: ("Branch length"). This asks you for the number of a node which is at the end of a branch length, then asks you whether you want to enter a branch length for that branch, change the branch length for that branch (if there is one already) or remove the branch length from the branch. N: ("Name"). This asks you which species you want to change the name for (referring to it by the number for that branch), then gives you the option of either removing the name, typing a new name, or leaving the name as is. Be sure not to try to enter a parentheses ("(" or ")"), a colon (":"), a comma (",") or a semicolon (";") in a name, as those may be mistaken for structural information about the tree when the tree file is read by another program. H, J, K, or L.: These are the movement commands for scrolling the "window" across a tree. H moves the "window" leftwards (though not beyond column 1, J moves it down, K up, and L right. The "window" will move 20 columns or rows at a time, and the tree will be redrawn in the new "window". Note that this amount of movement is not a full screen. C: ("Clade"). The C command instructs the program to print out only that part of the tree (the "clade") from a certain node on up. The program will prompt you for the number of this node. Remember that thereafter you are not looking at the whole tree. To go back to looking at the whole tree give the C command again and enter "0" for the node number when asked. Most users will not want to use this option unless forced to, as much can be accomplished with the window movement commands H, J, K, and L. +: ("next tree"). This causes the program to read in the next tree in the input file, if there is one. Currently the program does not detect gracefully that it has come to the end of the input tree file, and may crash with a "segmentation fault" if it does. However usually it will not lose any tree file that it has written. On Unix or Linux systems the crash may produce a useless "core dump" (a big file named "core") which you will want to delete. ?: ("Help"). Prints a one-screen summary of what the commands do, a few words for each command. X: ("Exit"). Exit from program. If the current tree has not yet been saved into a file, the program will first ask you whether it should be saved. Q: ("Quit"). A synonym for X. Same as the eXit command.

The program was written by Andrew Keeffe, using some code from Dnamove, which
he also wrote.

Below is a test tree file. We have already showed (above), what the
resulting tree display looks like when the terminal type is "none".
For ANSI or IBM PC screens it will look better, using the graphics characters
of those screens, which we do not attempt to show here.

---

### TEST INPUT TREE FILE

|  |
| --- |
| ``` ((((((((Human,Chimp),Gorilla),Orang),Gibbon),(Barbary_Ma,(Crab-e._Ma, (Rhesus_Mac,Jpn_Macaq)))),Squir._Mon),((Tarsier,Lemur),Bovine)),Mouse); ``` |
