# Supplementary material for: Probabilistic Phylogenetic Inference with Insertions and Deletions
Source: PLoS Comput Biol. 2008 Sep 19;4(9):e1000172. doi: 10.1371/journal.pcbi.1000172 (PMC2527138; doi:10.1371/journal.pcbi.1000172)
Supplement: Dataset S1 — Supplemental Material (24.89 MB GZ) [file pcbi.1000172.s001.gz › erate-supplement-R2/src/phylip3.66-erate/doc/dnapars.html]

main


version 3.66

# Dnapars -- DNA Parsimony Program

© Copyright 1986-2006 by The University of
Washington. Written by Joseph Felsenstein. Permission is granted to copy
this document provided that no fee is charged for it and that this copyright
notice is not removed.

This program carries out unrooted parsimony (analogous to Wagner
trees) (Eck and Dayhoff, 1966; Kluge and Farris, 1969) on DNA
sequences. The method of Fitch (1971) is used to count the number of
changes of base needed on a given tree.
The assumptions of this method are analogous to those of MIX:

1. Each site evolves independently.- Different lineages evolve independently.- The probability of a base substitution at a given site is
       small over the lengths of time involved in
       a branch of the phylogeny.- The expected amounts of change in different branches of the phylogeny
         do not vary by so much that two changes in a high-rate branch
         are more probable than one change in a low-rate branch.- The expected amounts of change do not vary enough among sites that two
           changes in one site are more probable than one change in another.

That these are the assumptions of parsimony methods has been documented
in a series of papers of mine: (1973a, 1978b, 1979, 1981b, 1983b, 1988b). For
an opposing view arguing that the parsimony methods make no substantive
assumptions such as these, see the papers by Farris (1983) and Sober (1983a,
1983b, 1988), but also read the exchange between Felsenstein and Sober (1986).

Change from an occupied site to a deletion is counted as one
change. Reversion from a deletion to an occupied site is allowed and is also
counted as one change. Note that this in effect assumes that a deletion
N bases long is N separate events.

Dnapars can handle both bifurcating and multifurcating trees. In doing its
search for most parsimonious trees, it adds species not only by creating new
forks in the middle of existing branches, but it also tries putting them at
the end of new branches which are added to existing forks. Thus it searches
among both bifurcating and multifurcating trees. If a branch in a tree
does not have any characters which might change in that branch in the most
parsimonious tree, it does not save that tree. Thus in any tree that
results, a branch exists only if some character has a most parsimonious
reconstruction that would involve change in that branch.

It also saves a number of trees tied for best (you can alter the number
it saves using the V option in the menu). When rearranging trees, it
tries rearrangements of all of the saved trees. This makes the algorithm
slower than earlier versions of Dnapars.

The input data is standard. The first line of the input file contains the
number of species and the number of sites.

Next come the species data. Each
sequence starts on a new line, has a ten-character species name
that must be blank-filled to be of that length, followed immediately
by the species data in the one-letter code. The sequences must either
be in the "interleaved" or "sequential" formats
described in the Molecular Sequence Programs document. The I option
selects between them. The sequences can have internal
blanks in the sequence but there must be no extra blanks at the end of the
terminated line. Note that a blank is not a valid symbol for a deletion.

The options are selected using an interactive menu. The menu looks like this:

|  |
| --- |
| ``` DNA parsimony algorithm, version 3.6  Setting for this run:   U                 Search for best tree?  Yes   S                        Search option?  More thorough search   V              Number of trees to save?  10000   J   Randomize input order of sequences?  No. Use input order   O                        Outgroup root?  No, use as outgroup species  1   T              Use Threshold parsimony?  No, use ordinary parsimony   N           Use Transversion parsimony?  No, count all steps   W                       Sites weighted?  No   M           Analyze multiple data sets?  No   I          Input sequences interleaved?  Yes   0   Terminal type (IBM PC, ANSI, none)?  ANSI   1    Print out the data at start of run  No   2  Print indications of progress of run  Yes   3                        Print out tree  Yes   4          Print out steps in each site  No   5  Print sequences at all nodes of tree  No   6       Write out trees onto tree file?  Yes    Y to accept these or type the letter for one to change ``` |

The user either types "Y" (followed, of course, by a carriage-return)
if the settings shown are to be accepted, or the letter or digit corresponding
to an option that is to be changed.

The S (search) option controls how, and how much, rearrangement is done on the
tied trees that are saved by the program. If the "More thorough search"
option (the default) is chosen, the program will save multiple tied trees,
without collapsing internal branches that have no evidence of change on them.
It will subsequently rearrange on all parts of each of those trees.
If the "Less thorough search" option is chosen, before saving,
the program will collapse
all branches that have no evidence that there is any change on that branch.
This leads to less attempted rearrangement. If the "Rearrange on
one best tree" option is chosen, only the first of the tied trees is used for
rearrangement. This is faster but less thorough. If your trees are likely
to have large multifurcations, do not use the default "More thorough search"
option as it could result in too large a number of trees being saved.

The N option allows you to choose transversion parsimony, which counts only
transversions (changes between one of the purines A or G and one of the
pyrimidines C or T). This setting is turned off by default.

The Weights (W) option
takes the weights from a file whose default name is "weights". The weights
follow the format described in the main documentation file, with integer
weights from 0 to 35 allowed by using the characters 0, 1, 2, ..., 9 and
A, B, ... Z.

The User tree (option U) is read from a file whose default name is intree.
The trees can be multifurcating. They must be preceded in the file by a
line giving the number of trees in the file.

The options J, O, T, M, and 0 are the usual ones. They are described in the
main documentation file of this package. Option I is the same as in
other molecular sequence programs and is described in the documentation file
for the sequence programs.

The M (multiple data sets option) will ask you whether you want to
use multiple sets of weights (from the weights file) or multiple data sets.
The ability to use a single data set with multiple weights means that
much less disk space will be used for this input data. The bootstrapping
and jackknifing tool Seqboot has the ability to create a weights file with
multiple weights.

The O (outgroup) option will have no effect if the U (user-defined tree)
option is in effect.
The T (threshold) option allows a continuum of methods
between parsimony and compatibility. Thresholds less than or equal to 1.0 do
not have any meaning and should
not be used: they will result in a tree dependent only on the input
order of species and not at all on the data!

Output is standard: if option 1 is toggled on, the data is printed out,
with the convention that "." means "the same as in the first species".
Then comes a list of equally parsimonious trees.
Each tree has branch lengths. These are computed using an algorithm
published by Hochbaum and Pathria (1997) which I first heard of from
Wayne Maddison who invented it independently of them. This algorithm
averages the number of reconstructed changes of state over all sites a
over all possible most parsimonious placements of the changes of state
among branches. Note that it does not correct in any way for multiple
changes that overlay each other.

If option 2 is
toggled on a table of the
number of changes of state required in each character is also
printed. If option 5 is toggled
on, a table is printed
out after each tree, showing for each branch whether there are known to be
changes in the branch, and what the states are inferred to have been at the
top end of the branch. This is a reconstruction of the ancestral sequences
in the tree. If you choose option 5, a menu item "." appears which gives you
the opportunity to turn off dot-differencing so that complete ancestral
sequences are shown. If the inferred state is a "?" or one of the IUB
ambiguity symbols, there will be multiple
equally-parsimonious assignments of states; the user must work these out for
themselves by hand. A "?" in the reconstructed states means that in
addition to one or more bases, a deletion may or may not be present. If
option 6 is left in its default state the trees
found will be written to a tree file, so that they are available to be used
in other programs.

If the U (User Tree) option is used and more than one tree is supplied, the
program also performs a statistical test of each of these trees against the
best tree. This test, which is a version of the test proposed by
Alan Templeton (1983) and evaluated in a test case by me (1985a). It is
closely parallel to a test using log likelihood differences
due to Kishino and Hasegawa (1989), and
uses the mean and variance of
step differences between trees, taken across sites. If the mean
is more than 1.96 standard deviations different then the trees are declared
significantly different. The program
prints out a table of the steps for each tree, the differences of
each from the best one, the variance of that quantity as determined by
the step differences at individual sites, and a conclusion as to
whether that tree is or is not significantly worse than the best one.
If the U (User Tree) option is used and more than one tree is supplied,
and the program is not told to assume autocorrelation between the
rates at different sites, the
program also performs a statistical test of each of these trees against the
one with highest likelihood. If there are two user trees, this
is a version of the test proposed by
Alan Templeton (1983) and evaluated in a test case by me (1985a). It is
closely parallel to a test using log likelihood differences
due to Kishino and Hasegawa (1989)
It uses the mean and variance of the differences in the number
of steps between trees, taken across sites. If the two
trees' means are more than 1.96 standard deviations different,
then the trees are
declared significantly different.

If there are more than two trees, the test done is an extension of
the KHT test, due to Shimodaira and Hasegawa (1999). They pointed out
that a correction for the number of trees was necessary, and they
introduced a resampling method to make this correction. In the version
used here the variances and covariances of the sums of steps across
sites are computed for all pairs of trees. To test whether the
difference between each tree and the best one is larger than could have
been expected if they all had the same expected number of steps,
numbers of steps for all trees are sampled with these covariances and equal
means (Shimodaira and Hasegawa's "least favorable hypothesis"),
and a P value is computed from the fraction of times the difference between
the tree's value and the lowest number of steps exceeds that actually
observed. Note that this sampling needs random numbers, and so the
program will prompt the user for a random number seed if one has not
already been supplied. With the two-tree KHT test no random numbers
are used.

In either the KHT or the SH test the program
prints out a table of the number of steps for each tree, the differences of
each from the lowest one, the variance of that quantity as determined by
the differences of the numbers of steps at individual sites,
and a conclusion as to
whether that tree is or is not significantly worse than the best one.

Option 6 in the menu controls whether the tree estimated by the program
is written onto a tree file. The default name of this output tree file
is "outtree". If the U option is in effect, all the user-defined
trees are written to the output tree file. If the program finds multiple
trees tied for best, all of these are written out onto the output tree
file. Each is followed by a numerical weight in square brackets (such as
[0.25000]). This is needed when we use the trees to make a consensus
tree of the results of bootstrapping or jackknifing, to avoid overrepresenting
replicates that find many tied trees.

The program is a straightforward relative of MIX
and runs reasonably quickly, especially with many sites and few species.

---

### TEST DATA SET

|  |
| --- |
| ```    5   13 Alpha     AACGUGGCCAAAU Beta      AAGGUCGCCAAAC Gamma     CAUUUCGUCACAA Delta     GGUAUUUCGGCCU Epsilon   GGGAUCUCGGCCC ``` |

---

### CONTENTS OF OUTPUT FILE (if all numerical options are on)

|  |
| --- |
| ``` DNA parsimony algorithm, version 3.66   5 species,  13  sites   Name            Sequences ----            ---------  Alpha        AACGUGGCCA AAU Beta         ..G..C.... ..C Gamma        C.UU.C.U.. C.A Delta        GGUA.UU.GG CC. Epsilon      GGGA.CU.GG CCC   One most parsimonious tree found:                                               +-----Epsilon                   +----------------------------3     +------------2                            +-------Delta        |            |     |            +----------------Gamma        |     1----Beta         |     +---------Alpha        requires a total of     19.000    between      and       length   -------      ---       ------      1           2       0.217949      2           3       0.487179      3      Epsilon      0.096154      3      Delta        0.134615      2      Gamma        0.275641      1      Beta         0.076923      1      Alpha        0.173077  steps in each site:          0   1   2   3   4   5   6   7   8   9      *-----------------------------------------     0|       2   1   3   2   0   2   1   1   1    10|   1   1   1   3                          From    To     Any Steps?    State at upper node                              ( . means same as in the node below it on tree)            1                AABGTCGCCA AAY    1      2         yes    V.KD...... C..    2      3         yes    GG.A..T.GG .C.    3   Epsilon     maybe   ..G....... ..C    3   Delta        yes    ..T..T.... ..T    2   Gamma        yes    C.TT...T.. ..A    1   Beta        maybe   ..G....... ..C    1   Alpha        yes    ..C..G.... ..T ``` |
