# Supplementary material for: Probabilistic Phylogenetic Inference with Insertions and Deletions
Source: PLoS Comput Biol. 2008 Sep 19;4(9):e1000172. doi: 10.1371/journal.pcbi.1000172 (PMC2527138; doi:10.1371/journal.pcbi.1000172)
Supplement: Dataset S1 — Supplemental Material (24.89 MB GZ) [file pcbi.1000172.s001.gz › erate-supplement-R2/src/phylip3.66-erate/doc/penny.html]

penny


version 3.66

# Penny - Branch and bound to find all most parsimonious trees

© Copyright 1986-2006 by the University of
Washington. Written by Joseph Felsenstein. Permission is granted to copy
this document provided that no fee is charged for it and that this copyright
notice is not removed.

Penny is a program that will find all of the most parsimonious trees
implied by your data. It does so not by examining all possible trees,
but by using the more sophisticated "branch and bound" algorithm, a
standard computer science search strategy first applied to
phylogenetic inference by Hendy and Penny (1982). (J. S. Farris
[personal communication, 1975] had also suggested that this strategy,
which is well-known in computer science, might
be applied to phylogenies, but he did not publish this suggestion).

There is, however, a price to be paid for the certainty that one has
found all members of the set of most parsimonious trees. The problem of
finding these has been shown (Graham and Foulds, 1982; Day, 1983) to be
NP-complete, which is equivalent to saying that there is no
fast algorithm that is guaranteed to solve the problem in all cases
(for a discussion of NP-completeness, see the Scientific American
article by Lewis and Papadimitriou, 1978). The result is that this
program, despite its algorithmic sophistication, is VERY SLOW.

The program should be slower than the other tree-building programs
in the package, but useable up to about ten species. Above this it will
bog down rapidly, but exactly when depends on the data and on how much
computer time you have (it may be more effective in the hands of someone
who can let a microcomputer grind all night than for someone who
has the "benefit" of paying for time on the campus mainframe computer). IT
IS VERY IMPORTANT FOR YOU TO GET A FEEL FOR HOW LONG THE PROGRAM
WILL TAKE ON YOUR DATA. This can be done by running it on subsets
of the species, increasing the number of species in the run until you
either are able to treat the full data set or know that the program
will take unacceptably long on it. (Making a plot of the logarithm of run
time against species number may help to project run times).

## The Algorithm

The search strategy used by Penny starts by making a tree consisting of the
first two species (the first three if the tree is to be unrooted). Then
it tries to add the next species in all possible places (there are three
of these). For each of the resulting trees it evaluates the number of
steps. It adds the next species to each of these, again in all
possible spaces. If this process would continue it would simply
generate all possible trees, of which there are a very large number even
when the number of species is moderate (34,459,425 with 10 species). Actually
it does not do this, because the trees are generated in a
particular order and some of them are never generated.

Actually the order in which trees are generated is not quite as
implied above, but is a "depth-first search". This
means that first one adds the third species in the first possible
place, then the fourth species in its first possible place, then
the fifth and so on until the first possible tree has been produced. Its
number of steps is evaluated. Then one "backtracks" by trying the
alternative placements of the last species. When these are exhausted
one tries the next placement of the next-to-last species. The
order of placement in a depth-first search is like this for a
four-species case (parentheses enclose monophyletic groups):

     Make tree of first two species     (A,B)  
          Add C in first place     ((A,B),C)  
               Add D in first place     (((A,D),B),C)  
               Add D in second place     ((A,(B,D)),C)  
               Add D in third place     (((A,B),D),C)  
               Add D in fourth place     ((A,B),(C,D))  
               Add D in fifth place     (((A,B),C),D)  
          Add C in second place: ((A,C),B)  
               Add D in first place     (((A,D),C),B)  
               Add D in second place     ((A,(C,D)),B)  
               Add D in third place     (((A,C),D),B)  
               Add D in fourth place     ((A,C),(B,D))  
               Add D in fifth place     (((A,C),B),D)  
          Add C in third place     (A,(B,C))  
               Add D in first place     ((A,D),(B,C))  
               Add D in second place     (A,((B,D),C))  
               Add D in third place     (A,(B,(C,D)))  
               Add D in fourth place     (A,((B,C),D))  
               Add D in fifth place     ((A,(B,C)),D)  

Among these fifteen trees you will find all of the four-species
rooted bifurcating trees, each exactly once (the parentheses each enclose
a monophyletic group). As displayed above, the backtracking
depth-first search algorithm is just another way of producing all
possible trees one at a time. The branch and bound algorithm
consists of this with one change. As each tree is constructed,
including the partial trees such as (A,(B,C)), its number of steps
is evaluated. In addition a prediction is made as to how many
steps will be added, at a minimum, as further species are added.

This is done by counting how many binary characters which are invariant in the
data up the species most recently added will ultimately show variation when
further species
are added. Thus if 20 characters vary among species A, B, and C and their
root, and if tree ((A,C),B) requires 24 steps, then if there are 8 more
characters which will be seen to vary when species D is added, we can
immediately say that no matter how we add D, the resulting tree can have no less
than 24 + 8 = 32 steps. The point of all this is that if a previously-found
tree such as ((A,B),(C,D)) required only 30 steps, then we know that
there is no point in even trying to add D to ((A,C),B). We have
computed the bound that enables us to cut off a whole line of inquiry
(in this case five trees) and avoid going down that particular branch
any farther.

The branch-and-bound algorithm thus allows us to find all most parsimonious
trees without generating all possible trees. How much of a saving this
is depends strongly on the data. For very clean (nearly "Hennigian")
data, it saves much time, but on very messy data it will still take
a very long time.

The algorithm in the program differs from the one outlined here
in some essential details: it investigates possibilities in the
order of their apparent promise. This applies to the order of addition
of species, and to the places where they are added to the tree. After
the first two-species tree is constructed, the program tries adding
each of the remaining species in turn, each in the best possible place it
can find. Whichever of those species adds (at a minimum) the most
additional steps is taken to be the one to be added next to the tree. When
it is added, it is added in turn to places which cause the fewest
additional steps to be added. This sounds a bit complex, but it is done
with the intention of eliminating regions of the search of all possible
trees as soon as possible, and lowering the bound on tree length as quickly
as possible.

The program keeps a list of all the most parsimonious
trees found so far. Whenever
it finds one that has fewer steps than
these, it clears out the list and
restarts the list with that tree. In the process the bound tightens and
fewer possibilities need be investigated. At the end the list contains
all the shortest trees. These are then printed out. It should be
mentioned that the program Clique for finding all largest cliques
also works by branch-and-bound. Both problems are NP-complete but for
some reason Clique runs far faster. Although their worst-case behavior
is bad for both programs, those worst cases occur far more frequently
in parsimony problems than in compatibility problems.

## Controlling Run Times

Among the quantities available to be set at the
beginning of a run of Penny, two (howoften and howmany) are of particular
importance. As Penny goes along it will keep count of how many
trees it has examined. Suppose that howoften is 100 and howmany is 1000,
the default settings. Every time 100 trees have been examined, Penny
will print out a line saying how many multiples of 100 trees have now been
examined, how many steps the most parsimonious tree found so far has,
how many trees of with that number of steps have been found, and a very
rough estimate of what fraction of all trees have been looked at so far.

When the number of these multiples printed out reaches the number howmany
(say 1000), the whole algorithm aborts and prints out that it has not
found all most parsimonious trees, but prints out what is has got so far
anyway. These trees need not be any of the most parsimonious trees: they are
simply the most parsimonious ones found so far. By setting the product
(howoften times howmany) large you can make
the algorithm less likely to abort, but then you risk getting bogged
down in a gigantic computation. You should adjust these constants so that
the program cannot go beyond examining the number of trees you are reasonably
willing to wait for. In their initial setting the program will
abort after looking at 100,000 trees. Obviously you may want to adjust
howoften in order to get more or fewer lines of intermediate notice of how
many trees have been looked at so far. Of course, in small cases you may
never even reach the first multiple of howoften and nothing will be printed out
except some headings and then the final trees.

The indication of the approximate percentage of trees searched so far will
be helpful in judging how much farther you would have to go to get the full
search. Actually, since that fraction is the fraction of the set of all
possible trees searched or ruled out so far, and since the search becomes
progressively more efficient, the approximate fraction printed out will
usually be an underestimate of how far along the program is, sometimes a
serious underestimate.

A constant that affects the result is
"maxtrees", which controls the
maximum number of trees that can be stored. Thus if
"maxtrees" is 25, and 32 most parsimonious trees are found,
only the first 25 of these are stored and printed out. If "maxtrees"
is increased, the program does not run any slower but requires a little
more intermediate storage space. I recommend that
"maxtrees" be kept as large as you can, provided you are willing to
look at an output with that many trees on it! Initially,
"maxtrees" is set to 100 in the distribution copy.

## Methods and Options

The counting of the length of trees is done by an algorithm nearly
identical to the corresponding algorithms in Mix, and thus the remainder
of this document will be nearly identical to the Mix document. Mix
is a general parsimony program which carries out the Wagner and
Camin-Sokal parsimony methods in mixture, where each character can have
its method specified. The program defaults to carrying out Wagner
parsimony.

The Camin-Sokal parsimony method explains the data by assuming that
changes 0 --> 1 are allowed but not changes 1 --> 0. Wagner parsimony
allows both kinds of changes. (This under the assumption that 0 is the
ancestral state, though the program allows reassignment of the ancestral
state, in which case we must reverse the state numbers 0 and 1
throughout this discussion). The criterion is to find the tree which
requires the minimum number of changes. The Camin-Sokal method is due
to Camin and Sokal (1965) and the Wagner method to Eck and Dayhoff
(1966) and to Kluge and Farris (1969).

Here are the assumptions of these two methods:

1. Ancestral states are known (Camin-Sokal) or unknown (Wagner).- Different characters evolve independently.- Different lineages evolve independently.- Changes 0 --> 1 are much more probable than changes 1 --> 0
         (Camin-Sokal) or equally probable (Wagner).- Both of these kinds of changes are a priori improbable over the
           evolutionary time spans involved in the differentiation of the
           group in question.- Other kinds of evolutionary event such as retention of polymorphism
             are far less probable than 0 --> 1 changes.- Rates of evolution in different lineages are sufficiently low that
               two changes in a long segment of the tree are far less probable
               than one change in a short segment.

That these are the assumptions of parsimony methods has been documented
in a series of papers of mine: (1973a, 1978b, 1979, 1981b,
1983b, 1988b). For an opposing view arguing that the parsimony methods
make no substantive
assumptions such as these, see the papers by Farris (1983) and Sober (1983a,
1983b), but also read the exchange between Felsenstein and Sober (1986).

The input for Penny is the standard input for discrete characters
programs, described above in the documentation file for the
discrete-characters programs. States "?", "P", and "B" are allowed.

Most of the options are selected using a menu:

|  |
| --- |
| ``` Penny algorithm, version 3.6  branch-and-bound to find all most parsimonious trees  Settings for this run:   X                     Use Mixed method?  No   P                     Parsimony method?  Wagner   F        How often to report, in trees:  100   H        How many groups of  100 trees:  1000   O                        Outgroup root?  No, use as outgroup species  1   S           Branch and bound is simple?  Yes   T              Use Threshold parsimony?  No, use ordinary parsimony   A   Use ancestral states in input file?  No   W                       Sites weighted?  No   M           Analyze multiple data sets?  No   0   Terminal type (IBM PC, ANSI, none)?  ANSI   1    Print out the data at start of run  No   2  Print indications of progress of run  Yes   3                        Print out tree  Yes   4     Print out steps in each character  No   5     Print states at all nodes of tree  No   6       Write out trees onto tree file?  Yes  Are these settings correct? (type Y or the letter for one to change) ``` |

The options X, O, T, A, and M are the usual Mixed Methods, Outgroup,
Threshold, Ancestral
States, and Multiple Data Sets options. They are described in the Main
documentation file and in the Discrete Characters Programs documentation
file. The O option is only acted upon if the final tree is unrooted.

The option P toggles between the Camin-Sokal parsimony criterion
and the Wagner parsimony criterion. Options F and H reset the
variables howoften (F) and howmany (H). The user is prompted for the new
values. By setting these larger the program will report its progress less
often (howoften) and will run longer (howmany times howoften). These values
default to 100 and 1000 which
guarantees a search of 100,000 trees, but these can be changed. Note that
option F in this program is not the Factors option available in some of
the other programs in this section of the package.

The A (Ancestral states) option works in the usual way, described in the
Discrete Characters Programs documentation file. If
the A option is not used, then the program will assume 0 as the
ancestral state for those characters following the Camin-Sokal method,
and will assume that the ancestral state is unknown for those characters
following Wagner parsimony. If any characters have unknown ancestral
states, and if the resulting tree is rooted (even by outgroup),
a table will be printed out
showing the best guesses of which are the ancestral states in each
character.

The S (Simple) option alters a step in Penny which reconsiders the
order in which species are added to the tree. Normally the decision as to
what species to add to the tree next is made as the first tree is being
constructed; that ordering of species is not altered subsequently. The
S option causes it to be continually reconsidered. This will probably
result in a substantial increase in run time, but on some data sets of
intermediate messiness it may help. It is included in case it might prove
of use on some data sets. The Simple option, in which the ordering is
kept the same after being established by trying alternatives during
the construction of the first tree, is the default. Continual reconsideration
can be selected as an alternative.

The F (Factors)
option is not available in this program, as it would have no effect on
the result even if that information were provided in the input file.

The final output is standard: a set of trees, which
will be printed as rooted or unrooted
depending on which is appropriate, and if the user elects to see them,
tables of the number of changes
of state required in each character. If the Wagner option is in force for a
character, it may not be possible to unambiguously locate the places on
the tree where the changes occur, as there may be multiple possibilities. A
table is available to be printed out after each tree, showing for each branch
whether
there are known to be changes in the branch, and what the states are inferred
to have been at the top end of the branch. If the inferred state is a "?"
there will be multiple equally-parsimonious assignments of states; the user
must work these out for themselves by hand.

If the Camin-Sokal parsimony method (option C or S)
is invoked and the A option is also used, then the program will
infer, for any character whose ancestral state is unknown ("?") whether the
ancestral state 0 or 1 will give the fewest state changes. If these are
tied, then it may not be possible for the program to infer the
state in the internal nodes, and these will all be printed as ".". If this
has happened and you want to know more about the states at the internal
nodes, you will find helpful to use Move to display the tree and examine
its interior states, as the algorithm in Move shows all that can be known
in this case about the interior states, including where there is and is not
amibiguity. The algorithm in Penny gives up more easily on displaying these
states.

If the A option is not used, then the program will assume 0 as the
ancestral state for those characters following the Camin-Sokal method,
and will assume that the ancestral state is unknown for those characters
following Wagner parsimony. If any characters have unknown ancestral
states, and if the resulting tree is rooted (even by outgroup),
a table will be printed out
showing the best guesses of which are the ancestral states in each
character. You will find it useful to understand the difference between
the Camin-Sokal parsimony criterion with unknown ancestral state and the Wagner
parsimony criterion.

If option 6 is left in its default state the trees
found will be written to a tree file, so that they are available to be used
in other programs. If the program finds multiple
trees tied for best, all of these are written out onto the output tree
file. Each is followed by a numerical weight in square brackets (such as
[0.25000]). This is needed when we use the trees to make a consensus
tree of the results of bootstrapping or jackknifing, to avoid overrepresenting
replicates that find many tied trees.

At the beginning of the program are a series of constants,
which can be changed to help adapt the program to different computer systems.
Two are the initial values of howmany and howoften,
constants "often" and "many". Constant "maxtrees"
is the maximum number of tied trees that will be stored.

---

### TEST DATA SET

|  |
| --- |
| ```     7    6 Alpha1    110110 Alpha2    110110 Beta1     110000 Beta2     110000 Gamma1    100110 Delta     001001 Epsilon   001110 ``` |

---

### TEST SET OUTPUT (with all numerical options turned on)

|  |
| --- |
| ``` Penny algorithm, version 3.66  branch-and-bound to find all most parsimonious trees   7 species,   6 characters Wagner parsimony method   Name         Characters ----         ----------  Alpha1       11011 0 Alpha2       11011 0 Beta1        11000 0 Beta2        11000 0 Gamma1       10011 0 Delta        00100 1 Epsilon      00111 0   requires a total of              8.000      3 trees in all found     +-----------------Alpha1       !     !        +--------Alpha2     --1        !     !  +-----4     +--Epsilon      !  !     !  +--6     !  !     +--5  +--Delta        +--2        !        !        +-----Gamma1          !        !           +--Beta2           +-----------3                    +--Beta1         remember: this is an unrooted tree!   steps in each character:          0   1   2   3   4   5   6   7   8   9      *-----------------------------------------     0!       1   1   1   2   2   1              From    To     Any Steps?    State at upper node                              ( . means same as in the node below it on tree)            1                11011 0   1    Alpha1       no     ..... .   1       2         no     ..... .   2       4         no     ..... .   4    Alpha2       no     ..... .   4       5         yes    .0... .   5       6         yes    0.1.. .   6    Epsilon      no     ..... .   6    Delta        yes    ...00 1   5    Gamma1       no     ..... .   2       3         yes    ...00 .   3    Beta2        no     ..... .   3    Beta1        no     ..... .     +-----------------Alpha1       !   --1  +--------------Alpha2       !  !     !  !           +--Epsilon      +--2        +--6        !  +-----5  +--Delta           !  !     !        +--4     +-----Gamma1             !           !        +--Beta2              +--------3                    +--Beta1         remember: this is an unrooted tree!   steps in each character:          0   1   2   3   4   5   6   7   8   9      *-----------------------------------------     0!       1   1   1   2   2   1              From    To     Any Steps?    State at upper node                              ( . means same as in the node below it on tree)            1                11011 0   1    Alpha1       no     ..... .   1       2         no     ..... .   2    Alpha2       no     ..... .   2       4         no     ..... .   4       5         yes    .0... .   5       6         yes    0.1.. .   6    Epsilon      no     ..... .   6    Delta        yes    ...00 1   5    Gamma1       no     ..... .   4       3         yes    ...00 .   3    Beta2        no     ..... .   3    Beta1        no     ..... .     +-----------------Alpha1       !     !           +-----Alpha2     --1  +--------2     !  !        !  +--Beta2        !  !        +--3     +--4           +--Beta1           !        !           +--Epsilon         !        +--6        +--------5  +--Delta                    !                 +-----Gamma1        remember: this is an unrooted tree!   steps in each character:          0   1   2   3   4   5   6   7   8   9      *-----------------------------------------     0!       1   1   1   2   2   1              From    To     Any Steps?    State at upper node                              ( . means same as in the node below it on tree)            1                11011 0   1    Alpha1       no     ..... .   1       4         no     ..... .   4       2         no     ..... .   2    Alpha2       no     ..... .   2       3         yes    ...00 .   3    Beta2        no     ..... .   3    Beta1        no     ..... .   4       5         yes    .0... .   5       6         yes    0.1.. .   6    Epsilon      no     ..... .   6    Delta        yes    ...00 1   5    Gamma1       no     ..... . ``` |
