# Supplementary material for: Probabilistic Phylogenetic Inference with Insertions and Deletions
Source: PLoS Comput Biol. 2008 Sep 19;4(9):e1000172. doi: 10.1371/journal.pcbi.1000172 (PMC2527138; doi:10.1371/journal.pcbi.1000172)
Supplement: Dataset S1 — Supplemental Material (24.89 MB GZ) [file pcbi.1000172.s001.gz › erate-supplement-R2/src/phylip3.66-erate/doc/neighbor.html]

neighbor


version 3.66

# Neighbor -- Neighbor-Joining and UPGMA methods

© Copyright 1991-2006 by the University of
Washington. Written by Joseph Felsenstein. Permission is granted to copy
this document provided that no fee is charged for it and that this copyright
notice is not removed.

This program implements the Neighbor-Joining method of Saitou and Nei (1987)
and the UPGMA method of clustering. The program was written by Mary Kuhner
and Jon Yamato, using some code from program Fitch. An important part of the
code was translated
from FORTRAN code from the neighbor-joining program written by Naruya Saitou
and by Li Jin, and is used with the kind permission of Drs. Saitou and Jin.

Neighbor constructs a tree by successive clustering of lineages, setting
branch lengths as the lineages join. The tree is not rearranged
thereafter. The tree does not assume an evolutionary clock, so that it
is in effect an unrooted tree. It should be somewhat similar to the tree
obtained by Fitch. The program cannot evaluate a User tree, nor can it prevent
branch lengths from becoming negative. However the algorithm is far faster
than Fitch or Kitsch. This will make it particularly effective in their place
for large studies or for bootstrap or jackknife resampling studies which
require runs on multiple data sets.

The UPGMA option constructs a tree by successive (agglomerative) clustering
using an average-linkage method of clustering. It has some relationship
to Kitsch, in that when the tree topology turns out the same, the
branch lengths with UPGMA will turn out to be the same as with the P = 0
option of Kitsch.

The options for Neighbor are selected through the menu, which looks like
this:

|  |
| --- |
| ``` Neighbor-Joining/UPGMA method version 3.6  Settings for this run:   N       Neighbor-joining or UPGMA tree?  Neighbor-joining   O                        Outgroup root?  No, use as outgroup species  1   L         Lower-triangular data matrix?  No   R         Upper-triangular data matrix?  No   S                        Subreplicates?  No   J     Randomize input order of species?  No. Use input order   M           Analyze multiple data sets?  No   0   Terminal type (IBM PC, ANSI, none)?  ANSI   1    Print out the data at start of run  No   2  Print indications of progress of run  Yes   3                        Print out tree  Yes   4       Write out trees onto tree file?  Yes     Y to accept these or type the letter for one to change ``` |

Most of the input options
(L, R, S, J, and M) are as given in the Distance Matrix Programs
documentation file,
that file, and their input format is the same as given there.
The O (Outgroup) option
is described in the main
documentation file of this package. It is not available when the
UPGMA option is selected. The Jumble option (J) does not allow
multiple jumbles (as most of the other programs that have it do),
as there is no objective way of choosing which of the multiple
results is best, there being no explicit criterion for optimality of the tree.

Option N chooses between the Neighbor-Joining and UPGMA methods. Option
S is the usual Subreplication option. Here, however, it is present only
to allow Neighbor to read the input data: the number of replicates is
actually ignored, even though it is read in. Note that this means that
one cannot use it to have missing data in the input file, if Neighbor is
to be used.

The output consists of an tree (rooted if UPGMA, unrooted if Neighbor-Joining)
and the lengths of the
interior segments. The Average Percent Standard Deviation is not
computed or printed out. If the tree found by Neighbor is fed into Fitch
as a User Tree, it will compute this quantity if one also selects the
N option of Fitch to ensure that none of the branch lengths is re-estimated.

As Neighbor runs it prints out an account of the successive clustering
levels, if you allow it to. This is mostly for reassurance and can be
suppressed using menu option 2. In this printout of cluster levels
the word "OTU" refers to a tip species, and the word "NODE" to an
interior node of the resulting tree.

The constants available for modification at the beginning of the
program are "namelength" which gives the length of a
species name, and the usual boolean
constants that initiliaze the terminal type. There is no feature saving
multiply trees tied for best,
partly because we do not expect exact ties except in cases where the branch
lengths make the nature of the tie obvious, as when a branch is of zero
length.

The major advantage of Neighbor is its speed: it requires a time only
proportional to the cube of the number of species. It is significantly
faster than version 3.5 of this program. By contrast Fitch
and Kitsch require a time that rises as the fourth power of the number
of species. Thus Neighbor is well-suited to bootstrapping studies and
to analysis of very large trees. Our simulation studies (Kuhner
and Felsenstein, 1994) show that, contrary to statements in the
literature by others, Neighbor does not get as accurate an estimate of
the phylogeny as does Fitch. However it does nearly as well, and in
view of its speed this will make it a quite useful program.

---

### TEST DATA SET

|  |
| --- |
| ```     7 Bovine      0.0000  1.6866  1.7198  1.6606  1.5243  1.6043  1.5905 Mouse       1.6866  0.0000  1.5232  1.4841  1.4465  1.4389  1.4629 Gibbon      1.7198  1.5232  0.0000  0.7115  0.5958  0.6179  0.5583 Orang       1.6606  1.4841  0.7115  0.0000  0.4631  0.5061  0.4710 Gorilla     1.5243  1.4465  0.5958  0.4631  0.0000  0.3484  0.3083 Chimp       1.6043  1.4389  0.6179  0.5061  0.3484  0.0000  0.2692 Human       1.5905  1.4629  0.5583  0.4710  0.3083  0.2692  0.0000 ``` |

---

### OUTPUT FROM TEST DATA SET (with all numerical options on)

|  |
| --- |
| ```    7 Populations  Neighbor-Joining/UPGMA method version 3.66    Neighbor-joining method   Negative branch lengths allowed   Name                       Distances ----                       ---------  Bovine        0.00000   1.68660   1.71980   1.66060   1.52430   1.60430               1.59050 Mouse         1.68660   0.00000   1.52320   1.48410   1.44650   1.43890               1.46290 Gibbon        1.71980   1.52320   0.00000   0.71150   0.59580   0.61790               0.55830 Orang         1.66060   1.48410   0.71150   0.00000   0.46310   0.50610               0.47100 Gorilla       1.52430   1.44650   0.59580   0.46310   0.00000   0.34840               0.30830 Chimp         1.60430   1.43890   0.61790   0.50610   0.34840   0.00000               0.26920 Human         1.59050   1.46290   0.55830   0.47100   0.30830   0.26920               0.00000     +---------------------------------------------Mouse        !    !                        +---------------------Gibbon       1------------------------2    !                        !  +----------------Orang        !                        +--5    !                           ! +--------Gorilla      !                           +-4    !                             ! +--------Chimp        !                             +-3    !                               +------Human        !    +------------------------------------------------------Bovine       remember: this is an unrooted tree!  Between        And            Length -------        ---            ------    1          Mouse           0.76891    1             2            0.42027    2          Gibbon          0.35793    2             5            0.04648    5          Orang           0.28469    5             4            0.02696    4          Gorilla         0.15393    4             3            0.03982    3          Chimp           0.15167    3          Human           0.11753    1          Bovine          0.91769 ``` |
