# Supplementary material for: Probabilistic Phylogenetic Inference with Insertions and Deletions
Source: PLoS Comput Biol. 2008 Sep 19;4(9):e1000172. doi: 10.1371/journal.pcbi.1000172 (PMC2527138; doi:10.1371/journal.pcbi.1000172)
Supplement: Dataset S1 — Supplemental Material (24.89 MB GZ) [file pcbi.1000172.s001.gz › erate-supplement-R2/src/phylip3.66-erate/doc/fitch.html]

fitch


version 3.66

# Fitch -- Fitch-Margoliash and Least-Squares Distance Methods

© Copyright 1986-2006 by the University of
Washington. Written by Joseph Felsenstein. Permission is granted to copy
this document provided that no fee is charged for it and that this copyright
notice is not removed.

This program carries out Fitch-Margoliash, Least Squares,
and a number of similar methods as described in the documentation
file for distance methods.

The options for Fitch are selected through the menu, which looks like
this:

|  |
| --- |
| ``` Fitch-Margoliash method version 3.6  Settings for this run:   D      Method (F-M, Minimum Evolution)?  Fitch-Margoliash   U                 Search for best tree?  Yes   P                                Power?  2.00000   -      Negative branch lengths allowed?  No   O                        Outgroup root?  No, use as outgroup species  1   L         Lower-triangular data matrix?  No   R         Upper-triangular data matrix?  No   S                        Subreplicates?  No   G                Global rearrangements?  No   J     Randomize input order of species?  No. Use input order   M           Analyze multiple data sets?  No   0   Terminal type (IBM PC, ANSI, none)?  ANSI   1    Print out the data at start of run  No   2  Print indications of progress of run  Yes   3                        Print out tree  Yes   4       Write out trees onto tree file?  Yes    Y to accept these or type the letter for one to change ``` |

Most of the input options
(U, P, -, O, L, R, S, J, and M) are as given in
the documentation page for distance matrix programs, and their input format is
the same as given there. The
U (User Tree) option has one additional feature when the N (Lengths)
option is used. This menu option will appear only if the U (User Tree)
option is selected. If N (Lengths) is set to "Yes" then if any branch
in the user tree has a branch length, that branch will not have its
length iterated. Thus you can prevent all branches from having their
lengths changed by giving them all lengths in the user tree, or hold
only one length unchanged by giving only that branch a length (such
as, for example, 0.00). You may find program Retree useful for
adding and removing branch lengths from a tree. This option can
also be used to compute the Average Percent Standard Deviation for a
tree obtained from Neighbor, for comparison with trees obtained by
Fitch or Kitsch.

The D (methods) option allows choice between the Fitch-Margoliash
criterion and the Minimum Evolution method (Kidd and Sgaramella-Zonta, 1971;
Rzhetsky and Nei, 1993). Minimum Evolution (not to be confused with
parsimony) uses the Fitch-Margoliash criterion to fit branch lengths to each
topology, but then chooses topologies based on their total branch length
(rather than the goodness of fit sum of squares). There is no
constraint on negative branch lengths in the Minimum Evolution method;
it sometimes gives rather strange results, as it can like solutions
that have large negative branch lengths, as these reduce the total
sum of branch lengths!

Another input option available in Fitch that is not available in Kitsch
or Neighbor
is the G (Global) option. G is the
Global search option. This causes, after the last species is added to
the tree, each possible group to be removed and re-added. This improves the
result, since the position of every species is reconsidered. It
approximately triples the run-time of the program. It is not an option in
Kitsch because it is the default and is always in force there. The
O (Outgroup) option is described in the main
documentation file of this package. The O option has no effect if the
tree is a user-defined tree (if the U option is in effect). The
U (User Tree) option requires an unrooted tree; that is, it require
that the tree have a trifurcation at its base:

     ((A,B),C,(D,E));

The output consists of an unrooted tree and the lengths of the
interior segments. The sum of squares is printed out, and if *P = 2.0*
Fitch and Margoliash's "average percent standard deviation" is also computed
and printed out. This is the sum of squares, divided by
N-2, and
then square-rooted and then multiplied by 100 (n is the number of
species on the tree):

     APSD = ( SSQ / (N-2) )1/2 x 100.

where *N* is the total number of off-diagonal distance measurements that
are in the (square) distance matrix. If the S (subreplication) option
is in force it is instead the sum of the numbers of replicates in all the
non-diagonal
cells of the distance matrix. But if the L or R option is also in effect, so
that the distance matrix read in is lower- or upper-triangular, then the
sum of replicates is only over those cells actually read in. If S is not in
force, the number of replicates in
each cell is assumed to be 1, so that *N* is *n(n-1)*,
where *n* is the number
of species. The *APSD* gives an indication of the average percentage
error. The number of trees examined is also printed out.

The constants
available for modification at the beginning of the program are:
"smoothings", which gives the number of passes through
the algorithm which adjusts the lengths of the segments of the tree so
as to minimize the sum of squares, "delta", which controls the size of
improvement in sum of squares that is used to control the number of
iterations improving branch lengths,
and "epsilonf",
which defines a small quantity needed in
some of the calculations. There is no feature saving multiply trees
tied for best,
partly because we do not expect exact ties except in cases where the branch
lengths make the nature of the tie obvious, as when a branch is of zero
length.

The algorithm can be slow. As the number of species
rises, so does the number of distances from each species to the others. The
speed of this algorithm will thus rise as the fourth power of the
number of species, rather than as the third power as do most of the
others. Hence it is expected to get very slow as the number of species
is made larger.

---

### TEST DATA SET

|  |
| --- |
| ```     7 Bovine      0.0000  1.6866  1.7198  1.6606  1.5243  1.6043  1.5905 Mouse       1.6866  0.0000  1.5232  1.4841  1.4465  1.4389  1.4629 Gibbon      1.7198  1.5232  0.0000  0.7115  0.5958  0.6179  0.5583 Orang       1.6606  1.4841  0.7115  0.0000  0.4631  0.5061  0.4710 Gorilla     1.5243  1.4465  0.5958  0.4631  0.0000  0.3484  0.3083 Chimp       1.6043  1.4389  0.6179  0.5061  0.3484  0.0000  0.2692 Human       1.5905  1.4629  0.5583  0.4710  0.3083  0.2692  0.0000 ``` |

---

### OUTPUT FROM TEST DATA SET (with all numerical options on)

|  |
| --- |
| ```    7 Populations  Fitch-Margoliash method version 3.66                    __ __             2                   \  \   (Obs - Exp) Sum of squares =  /_ /_  ------------                                 2                    i  j      Obs  Negative branch lengths not allowed   Name                       Distances ----                       ---------  Bovine        0.00000   1.68660   1.71980   1.66060   1.52430   1.60430               1.59050 Mouse         1.68660   0.00000   1.52320   1.48410   1.44650   1.43890               1.46290 Gibbon        1.71980   1.52320   0.00000   0.71150   0.59580   0.61790               0.55830 Orang         1.66060   1.48410   0.71150   0.00000   0.46310   0.50610               0.47100 Gorilla       1.52430   1.44650   0.59580   0.46310   0.00000   0.34840               0.30830 Chimp         1.60430   1.43890   0.61790   0.50610   0.34840   0.00000               0.26920 Human         1.59050   1.46290   0.55830   0.47100   0.30830   0.26920               0.00000     +---------------------------------------------Mouse        !    !                                +------Human        !                             +--5    !                           +-4  +--------Chimp        !                           ! !    !                        +--3 +---------Gorilla      !                        !  !    1------------------------2  +-----------------Orang        !                        !    !                        +---------------------Gibbon       !    +------------------------------------------------------Bovine       remember: this is an unrooted tree!  Sum of squares =     0.01375  Average percent standard deviation =     1.85418  Between        And            Length -------        ---            ------    1          Mouse             0.76985    1             2              0.41983    2             3              0.04986    3             4              0.02121    4             5              0.03695    5          Human             0.11449    5          Chimp             0.15471    4          Gorilla           0.15680    3          Orang             0.29209    2          Gibbon            0.35537    1          Bovine            0.91675 ``` |
