# Supplementary material for: Probabilistic Phylogenetic Inference with Insertions and Deletions
Source: PLoS Comput Biol. 2008 Sep 19;4(9):e1000172. doi: 10.1371/journal.pcbi.1000172 (PMC2527138; doi:10.1371/journal.pcbi.1000172)
Supplement: Dataset S1 — Supplemental Material (24.89 MB GZ) [file pcbi.1000172.s001.gz › erate-supplement-R2/src/phylip3.66-erate/doc/discrete.html]

discrete


version 3.66

# DOCUMENTATION FOR (0,1) DISCRETE CHARACTER PROGRAMS

© Copyright 1986-2006 by the University of
Washington. Written by Joseph Felsenstein. Permission is granted to copy
this document provided that no fee is charged for it and that this copyright
notice is not removed.

These programs are intended for the use of morphological
systematists who are dealing with discrete characters,
or by molecular evolutionists dealing with presence-absence data on
restriction sites. One of the programs (Pars) allows multistate
characters, with up to 8 states, plus the unknown state symbol "?".
For the others, the characters
are assumed to be coded into a series of (0,1) two-state characters. For
most of the programs there are two other states possible, "P", which
stands for the state of Polymorphism for both states (0 and 1), and "?",
which stands for the state of ignorance: it is the state "unknown", or
"does not apply". The state "P" can also be denoted by "B", for "both".

There is a method invented by Sokal and Sneath (1963) for linear
sequences of character states, and fully developed for branching sequences
of character states
by Kluge and Farris (1969) for recoding a multistate character
into a series of two-state (0,1) characters. Suppose we had a character
with four states whose character-state tree had the rooted form:

```
               1 ---> 0 ---> 2
                      |
                      |
                      V
                      3
```

so that 1 is the ancestral state and 0, 2 and 3 derived states. We can
represent this as three two-state characters:

```
                Old State           New States
                --- -----           --- ------
                    0                  001
                    1                  000
                    2                  011
                    3                  101
```

The three new states correspond to the three arrows in the above character
state tree. Possession of one of the new states corresponds to whether or not
the old state had that arrow in its ancestry. Thus the first new state
corresponds to the bottommost arrow, which only state 3 has in its ancestry,
the second state to the rightmost of the top arrows, and the third state to
the leftmost top arrow. This coding will guarantee that the number of times
that states arise on the tree (in programs Mix, Move, Penny and Boot)
or the number of polymorphic states in a tree segment (in the Polymorphism
option of Dollop, Dolmove, Dolpenny and Dolboot) will correctly
correspond to what would have been the case had our programs been able to take
multistate characters into account. Although I have shown the above character
state tree as rooted, the recoding method works equally well on unrooted
multistate characters as long as the connections between the states are known
and contain no loops.

However, in the default option of programs Dollop, Dolmove, Dolpenny
and Dolboot the multistate recoding does not necessarily work properly, as it
may lead the program to reconstruct nonexistent state combinations such as
010. An example of this problem is given in my paper on alternative
phylogenetic methods (1979).

If you have multistate character data where the states are connected in a
branching "character state tree" you may want to do the binary recoding
yourself. Thanks to Christopher Meacham, the package contains
a program, Factor, which will do the recoding itself. For details see
the documentation file for Factor.

We now also have the program Pars, which can do parsimony for unordered
character states.

## COMPARISON OF METHODS

The methods used in these programs make different assumptions about
evolutionary rates, probabilities of different kinds of events, and our
knowledge about the characters or about the character state trees.
Basic references on these assumptions are my 1979, 1981b and 1983b
papers, particularly the latter. The
assumptions of each method are briefly described in the documentation
file for the corresponding program. In most cases my assertions about what are
the assumptions of these methods are challenged by others, whose papers I also
cite at that point. Personally, I believe that they are wrong and I am
right. I must emphasize the importance of
understanding the assumptions underlying the methods you are using. No
matter how fancy the algorithms, how maximum the likelihood or how
minimum the number of steps, your results can only be as good as the
correspondence between biological reality and your assumptions!

## INPUT FORMAT

The input format is as described in the general documentation file. The
input starts with a line containing the number of
species and the number of characters.

In Pars, each character can have up to 8 states plus a "?" state. In any
character, the first 8 symbols encountered will be taken to represent
these states. Any of the digits 0-9, letters A-Z and a-z, and even symbols
such as + and -, can be used (and in fact which 8 symbols are used can
be different in different characters).

In the other discrete characters programs the allowable states are,
0, 1, P, B, and ?. Blanks
may be included between the states (i. e. you can have a
species whose data is DISCOGLOSS0 1 1 0 1 1 1). It is possible for
extraneous information to follow the end of the character state data on
the same line. For example, if there were 7 characters in the data set,
a line of species data could read "DISCOGLOSS0110111 Hello there").

The discrete character data can continue to a new line whenever needed.
The characters are not in the "aligned" or "interleaved" format used by the
molecular sequence programs: they have the name and entire set of characters
for one species, then the name and entire set of characters for the next
one, and so on. This is known as the sequential format. Be particularly
careful when you use restriction sites
data, which can be in either the aligned or the sequential format for use in
Restml but must be in the sequential format for these discrete character
programs.

For Pars the discrete character data can be in either Sequential or
Interleaved format; the latter is the default.

Errors in the input data will often be detected by the programs, and this will
cause them to issue an error message such as 'BAD OUTGROUP NUMBER: ' together
with information as to which species, character, or in this case outgroup
number is the incorrect one. The program will them terminate; you will have
to look at the data and figure out what went wrong and fix it. Often an error
in the data causes a lack of synchronization between what is in the data file
and what the program thinks is to be there. Thus a missing character may
cause the program to read part of the next species name as a character and
complain about its value. In this type of case you should look for the error
earlier in the data file than the point about which the program is
complaining.

## OPTIONS GENERALLY AVAILABLE

Specific information on options will be given in the documentation
file associated with each program. However, some options occur in many
programs. Options are selected from the menu in each
program.

- The A (Ancestral states) option. This indicates that we are
  specifying the ancestral states for each character. In the menu the
  ancestors (A) option must be selected.
  An ancestral states input file is read, whose default name is
  ancestors. It contains
  a line or lines giving the ancestral states for each character.
  These may be 0, 1 or ?, the latter
  indicating that the ancestral state is unknown.

  An example is:

  001??11

  The ancestor information can be continued to a new line and can have blanks
  between any of the characters in the same way that species character data
  can.
  In the program Clique the ancestor is instead to be included as a
  regular species and
  no A option is available.

  - The F (Factors) option. This is used in programs Move, Dolmove,
    and Factor. It specifies which binary characters correspond
    to which multistate characters. To use the F option you
    choose the F option in the program menu. After that the program
    will read a factors file (default name factors
    Which consists of a line or lines containing a symbol
    for each binary character. The
    symbol can be anything, provided that it is the same for binary characters
    that correspond to the same multistate character, and changes between
    multistate characters. A good practice is to make it the lower-order digit
    of the number of the multistate character.

    For example, if there were 20 binary characters that had been generated by
    nine multistate characters having respectively 4, 3, 3, 2, 1, 2, 2, 2, and 1
    binary factors you would make the factors file be:

    11112223334456677889

    although it could equivalently be:

    aaaabbbaaabbabbaabba

    All that is important is that the symbol
    for each binary character change only when adjacent binary characters
    correspond to different mutlistate characters. The factors
    file contents
    can continue to a new line at any time except during the initial characters
    filling out the length of a species name.

    - The J (Jumble) option. This causes the species to be entered into the
      tree in a random order rather than in their order in the input file. The
      program prompts you for a random number seed. This option is described in
      the main documentation file.

      - The M (Multiple data sets) option. This has also been described in the
        main documentation file. It is not to be confused with the M option specified
        in the input file, which is the Mixture of methods option (yes, I know
        this is confusing).

        - The O (outgroup) option. This has also already been discussed in the
          general documentation file. It specifies the number of the particular species
          which will be used as the outgroup in rerooting the final tree when it is
          printed out. It will not have any effect if the tree is already rooted or is
          a user-defined tree. This option is not available in Dollop, Dolmove,
          or Dolpenny, which always infer a rooted tree, or Clique, which
          requires you to work out the rerooting by hand. The menu selection will
          cause you to be prompted for the number of the outgroup.

          - The T (threshold) option. This sets a threshold such that if the
            number of steps counted in a character is higher than the threshold, it
            will be taken to be the threshold value rather than the actual number of
            steps. This option has already been described in the main documentation
            file. The user is prompted for the threshold value. My 1981 paper
            (Felsenstein, 1981b)
            explains the logic behind the Threshold option, which is an attarctive
            alternative to successive weighting of characters.

            - The U (User tree) option. This has already been described in the
              main documentation file. For all of these programs user trees are to be
              specified as bifurcating trees, even in the cases where the tree that
              is inferred by the programs is to be regarded as unrooted.

              - The W (Weights) option. This allows us to specify weights on the
                characters, including the possibility of omitting characters from the
                analysis. It has already been described in the main documentation file.

                - The X (miXture) option. In the programs Mix, Move, and Penny
                  the user can specify for each character which parsimony method is
                  in effect. This is done by selecting menu option X (not M) and having
                  an input mixture file, whose default name is mixture.
                  It contains a line or lines with and one letter for
                  each character. These letters are C or S if the character is to
                  be reconstructed according to Camin-Sokal parsimony, W or ? if the
                  character is to be reconstructed according to Wagner parsimony. So if
                  there are 20 characters the line giving the mixture might look like this:

                  ```
                  WWWCC WWCWC
                  ```

                  Note that blanks in the seqence of characters (after the first ones that
                  are as long as the species names) will be ignored, and the information
                  can go on to a new line at any point. So this could equally well have been
                  specified by

                  ```
                  WW
                  CCCWWCWC
                  ```

In Pars you can, using the menu, turn off this dot-differencing
convention and see all states at all hypothetical ancestral nodes of the tree.

On the line in that table corresponding to each branch of the tree will also
be printed "yes", "no" or "maybe" as an answer to the question of whether this
branch is of nonzero length. If there is no evidence that any character has
changed in that branch, then "no" will be printed. If there is definite
evidence that one has changed, then "yes" will be printed. If the matter is
ambiguous, then "maybe" will be printed. You should keep in mind that all of
these conclusions assume that we are only interested in the assignment of
states that requires the least amount of change. In reality, the confidence
limit on tree topology usually includes many different topologies, and
presumably also then the confidence limits on amounts of change in branches
are also very broad.

In addition to the table showing numbers of events, a table may be printed out
showing which ancestral state causes the fewest events for each
character. This will not always be done, but only when the tree is rooted and
some ancestral states are unknown. This can be used to infer states of
occurred and making it easy for the user to reconstruct all the alternative
patterns of the characters states in the hypothetical ancestral nodes.
In Pars you can, using the menu, turn off this dot-differencing
convention and see all states at all hypothetical ancestral nodes of the tree.

On the line in that table corresponding to each branch of the tree will also
be printed "yes", "no" or "maybe" as an answer to the question of whether this
branch is of nonzero length. If there is no evidence that any character has
changed in that branch, then "no" will be printed. If there is definite
evidence that one has changed, then "yes" will be printed. If the matter is
ambiguous, then "maybe" will be printed. You should keep in mind that all of
these conclusions assume that we are only interested in the assignment of
states that requires the least amount of change. In reality, the confidence
limit on tree topology usually includes many different topologies, and
presumably also then the confidence limits on amounts of change in branches
are also very broad.

In addition to the table showing numbers of events, a table may be printed out
showing which ancestral state causes the fewest events for each
character. This will not always be done, but only when the tree is rooted and
some ancestral states are unknown. This can be used to infer states of
ancestors. For example, if you use the O (Outgroup) and A (Ancestral states)
options together, with at least some of the ancestral states being given as
"?", then inferences will be made for those characters, as the outgroup makes
the tree rooted if it was not already.

In programs Mix and Penny, if you are using the Camin-Sokal parsimony option
with ancestral state "?" and it turns out that the program cannot decide
between ancestral states 0 and 1, it will fail to even attempt reconstruction
of states of the hypothetical ancestors, printing them all out as "." for
occurred and making it easy for the user to reconstruct all the alternative
patterns of the characters states in the hypothetical ancestral nodes.
In Pars you can, using the menu, turn off this dot-differencing
convention and see all states at all hypothetical ancestral nodes of the tree.

On the line in that table corresponding to each branch of the tree will also
be printed "yes", "no" or "maybe" as an answer to the question of whether this
branch is of nonzero length. If there is no evidence that any character has
changed in that branch, then "no" will be printed. If there is definite
evidence that one has changed, then "yes" will be printed. If the matter is
ambiguous, then "maybe" will be printed. You should keep in mind that all of
these conclusions assume that we are only interested in the assignment of
states that requires the least amount of change. In reality, the confidence
limit on tree topology usually includes many different topologies, and
presumably also then the confidence limits on amounts of change in branches
are also very broad.

In addition to the table showing numbers of events, a table may be printed out
showing which ancestral state causes the fewest events for each
character. This will not always be done, but only when the tree is rooted and
some ancestral states are unknown. This can be used to infer states of
occurred and making it easy for the user to reconstruct all the alternative
patterns of the characters states in the hypothetical ancestral nodes.
In Pars you can, using the menu, turn off this dot-differencing
convention and see all states at all hypothetical ancestral nodes of the tree.

On the line in that table corresponding to each branch of the tree will also
be printed "yes", "no" or "maybe" as an answer to the question of whether this
branch is of nonzero length. If there is no evidence that any character has
changed in that branch, then "no" will be printed. If there is definite
evidence that one has changed, then "yes" will be printed. If the matter is
ambiguous, then "maybe" will be printed. You should keep in mind that all of
these conclusions assume that we are only interested in the assignment of
states that requires the least amount of change. In reality, the confidence
limit on tree topology usually includes many different topologies, and
presumably also then the confidence limits on amounts of change in branches
are also very broad.

In addition to the table showing numbers of events, a table may be printed out
showing which ancestral state causes the fewest events for each
character. This will not always be done, but only when the tree is rooted and
some ancestral states are unknown. This can be used to infer states of
ancestors. For example, if you use the O (Outgroup) and A (Ancestral states)
options together, with at least some of the ancestral states being given as
"?", then inferences will be made for those characters, as the outgroup makes
the tree rooted if it was not already.

In programs Mix and Penny, if you are using the Camin-Sokal parsimony option
with ancestral state "?" and it turns out that the program cannot decide
between ancestral states 0 and 1, it will fail to even attempt reconstruction
of states of the hypothetical ancestors, printing them all out as "." for
those characters. This is done for internal bookkeeping reasons -- to
reconstruct their changes would require a fair amount of additional code and
additional data structures. It is not too hard to reconstruct the internal
states by hand, trying the two possible ancestral states one after the
other. A similar comment applies to the use of ancestral state "?" in the
Dollo or Polymorphism parsimony methods (programs Dollop and Dolpenny) which
also can result in a similar hesitancy to print the estimate of the states of
the hypothetical ancestors. In all of these cases the program will print "?"
rather than "no" when it describes whether there are any changes in a branch,
since there might or might not be changes in those characters which are not
reconstructed.

For further information see the documentation files for the
individual programs.
