# Supplementary material for: Probabilistic Phylogenetic Inference with Insertions and Deletions
Source: PLoS Comput Biol. 2008 Sep 19;4(9):e1000172. doi: 10.1371/journal.pcbi.1000172 (PMC2527138; doi:10.1371/journal.pcbi.1000172)
Supplement: Dataset S1 — Supplemental Material (24.89 MB GZ) [file pcbi.1000172.s001.gz › erate-supplement-R2/src/phylip3.66-erate/doc/drawgram.html]

drawgram


version 3.66

# Drawgram

© Copyright 1990-2006 by The University of
Washington. Written by Joseph Felsenstein. Permission is granted to copy
this document provided that no fee is charged for it and that this copyright
notice is not removed.

Drawgram interactively plots a cladogram- or phenogram-like rooted tree
diagram, with many options including orientation of tree and branches,
style of tree, label sizes and angles, tree depth, margin sizes, stem
lengths, and placement of nodes in the tree. Particularly if you can
use your computer to preview the plot, you can very
effectively adjust the details of the plotting to get just the kind of
plot you want.

To understand the working of Drawgram and Drawtree, you should first
read the Tree Drawing Programs web page
in this documentation.

As with Drawtree, to run Drawgram you need a compiled copy of the
program, a font file, and a tree file. The tree file has a default name
of intree. The font file has a default name of "fontfile". If there is
no file of that name, the program will ask you for the name of a font file
(we provide ones that have the names font1 through font6).
Once you decide on a favorite one of these, you could make a copy of it
and call it fontfile, and it will then be used by default.
Note that the program will get confused if the input tree file has the number
of trees on the first line of the file, so that numbr may have to be removed.

Once these choices have been made you will see the central menu of the
program, which looks like this:

|  |
| --- |
| ``` Rooted tree plotting program version 3.6  Here are the settings:  0  Screen type (IBM PC, ANSI):  ANSI  P       Final plotting device:  Postscript printer  V           Previewing device:  X Windows display  H                  Tree grows:  Horizontally  S                  Tree style:  Phenogram  B          Use branch lengths:  (no branch lengths available)  L             Angle of labels:  90.0  R      Scale of branch length:  Automatically rescaled  D       Depth/Breadth of tree:  0.53  T      Stem-length/tree-depth:  0.05  C    Character ht / tip space:  0.3333  A             Ancestral nodes:  Centered  F                        Font:  Times-Roman  M          Horizontal margins:  1.65 cm  M            Vertical margins:  2.16 cm  #              Pages per tree:  one page per tree   Y to accept these or type the letter for one to change ``` |

These are the settings that control the appearance of the tree, which
has already been read in. You can either accept these as is, in which
case you would answer Y to the question and press the Return or Enter
key, or you can answer N if you want to change one, or simply type the
character corresponding to the one you want to change (if you answer N it
will just immediately ask you for that number anyway).

For a first run, particularly if previewing is available, you might accept
these default values and see what the result looks like. The program
will then tell you it is about to preview the tree and ask you to press
Return or Enter when you are ready to see this (you will probably have
to press it twice). If you are on a Windows system (and have its graphics
selected as your previewing option), on a Unix or Linux system and are
using X windows for previewing, or are on a Macintosh system, a new
window will open with the preview in it. If you are using the Tektronix
preview option the preview will appear in the window where the menu was.

On X Windows, Macintosh, and Windows you can resize the preview window,
though for some of these you may have to ask the system to redraw the
preview to see it at the new window size.

Once you are finished looking at the preview, you will want to
specify whether the program should make the final plot or change some of
the settings. This is done differently on the different previews:

- In **X Windows** you should make the menu window the active
  window. You may need to move the mouse over it, or click in it, or
  click on its top bar. You do not need to try to close the preview
  window yourself, and usually if you do this will cause trouble.- In **Windows** use the File menu in the preview window
    and choose either the Change Parameters menu item, or if
    you are ready to make the final plot, choose the Plot menu item.- On a **Macintosh** system, you can simply use the little box in
      the corner of the preview window to close it. The text window for the
      menu will then be active.- In **PC graphics** press on the Enter key. The screen with the
        preview should disappear and the settings menu reappear.- With a **Tektronix** preview, you may need to change your screen
          from a Tektronix-compatible mode to see the menu again.

Except with the Macintosh preview, the program will
now ask you if the tree is now ready to be plotted. If you answer Y (for
Yes) (or choose this option in the File menu of the preview
window in the case of Windows) the program will usually write a plot file
(with some plot options it will draw the tree on the screen). Then it will
terminate.

But if you do not say that you are ready to plot the tree,
it will go back to the above menu, allow
you to change more options, and go through the whole process again. The
easiest way to learn the meaning of the options is to try them,
particularly if previewing is available. Below I will describe them one
by one; you may prefer to skip reading this unless you are puzzled about
one of them.

## THE OPTIONS

O: This is an option that allows you to change the menu window to be an ANSI terminal or an IBM PC terminal. Generally you will not want to change this. P: This allows you to choose the Plotting device or file format. We have discussed the possible choices in the draw programs documentation web page. V: This allows you to change the type of preView window (or even turn off previewing. We have discussed the different possible choices in the draw programs documentation web page. H: Whether the tree grows Horizontally or vertically. The horizontal growth will be from left to right. This option is self explanatory. The other options are designed so that when we switch this direction of growth the tree still looks the same, except for orientation and overall size. This option is toggled, that is, when it is chosen the orientation changes, going back and forth between Vertical and Horizontal. The default orientation is Horizontal. S: The Style of the tree. There are six styles possible: Cladogram, Phenogram, Curvogram, Eurogram, Swoopogram, and Circular Tree. These are chosen by the letters C, P, V, E, S and O. These take a little explaining. In spite of the words "cladogram" and "phenogram", there is no implication of the extent to which you consider these diagrams as being genealogies or phenetic clustering diagrams. The names refer to pictorial style, not your own intended final use for the diagram. The six styles can be described as follows (assuming a vertically growing tree): Cladogram: nodes are connected to other nodes and to tips by straight lines going directly from one to the other. This gives a V-shaped appearance. The default settings if there are no branch lengths are designed to yield a V-shaped tree with a 90-degree angle at the base. Phenogram: nodes are connected to other nodes and to other tips by a horizontal and then a vertical line. This gives a particularly precise idea of horizontal levels. Curvogram: nodes are connected to other nodes and to tips by a curve which is one fourth of an ellipse, starting out horizontally and then curving upwards to become vertical. This pattern was suggested by Joan Rudd. Eurogram: so-called because it is a version of cladogram diagram popular in Europe. Nodes are connected to other nodes and to tips by a diagonal line that goes outwards and goes at most one-third of the way up to the next node, then turns sharply straight upwards and is vertical. Unfortunately it is nearly impossible to guarantee, when branch lengths are used, that the angles of divergence of lines are the same. Swoopogram: this option connects two nodes or a node and a tip using two curves that are actually each one-quarter of an ellipse. The first part starts out vertical and then bends over to become horizontal. The second part, which is at least two-thirds of the total, starts out horizontal and then bends up to become vertical. The effect is that two lineages split apart gradually, then more rapidly, then both turn upwards. Circular Tree: This is a style introduced by David Swofford in PAUP\*. The tree grows outward from a central point, being essentially a Phenogram style tree in polar coordinates. The tips form a 360-degree circle. The "vertical" lines run outward radially from the center, and the "horizontal" lines are arcs of circles centered on it. You should experiment with these and decide which you want -- it depends very much on the effect you want. B: Whether the tree has Branch lengths that are being used in the diagram. If the tree that was read in had a full set of branch lengths, it will be assumed as a default that you want to use them in the diagram, but you can specify that they are not to be used. If the tree does not have a full set of branch lengths then this will be indicated, and if you try to use branch lengths the program will refuse to allow you to do so. Note that when you change option B, the node position option A may change as well. L: The angle of the Labels. The angle is always calculated relative to a vertical tree; whether the tree is horizontal or vertical, if the labels are at an angle of 90 degrees they run parallel to direction of tree growth. The default value is 90 degrees. The option allows you to choose any angle from 0 to 90 degrees. R: How the branch lengths will be translated into distances on the output device. Note that when branch lengths have not been provided, there are implicit branch lengths specified by the type of tree being drawn. This option will toggle back and forth between automatic adjustment of branch lengths so that the diagram will just fit into the margins, and you specifying how many centimeters there will be per unit branch length. This is included so that you can plot different trees to a common scale, showing which ones have longer or shorter branches than others. Note that if you choose too large a value for centimeters per unit branch length, the tree will be so big it will overrun the plotting area and may cause failure of the diagram to display properly. Too small a value will cause the tree to be a nearly invisible dot. D: The ratio between the Depth and the breadth of the tree. It is initially set near 0.5, to approximate a V-shaped tree, but you may want to try a larger value to get a longer and narrower tree. Depth and breadth are described as if the tree grew vertically, so that depth is always measured from the root to the tips (not including the length of the labels). T: The length of the sTem of the tree as a fraction of the depth of the tree. You may want to either lengthen the stem or remove it entirely by giving a value of zero. C: The Character height, measured as a fraction of the tip spacing. If the labels are rotated to a shallow angle, the character height will be automatically adjusted in hopes of avoiding collision of labels at different tips. This option allows you to change the size of the labels yourself. On output devices where line thicknesses can be varied, the thickness of the tree lines will automatically be adjusted to be proportional to the character height, which is an additional reason you may want to change character height. A: Controls the positions of the Ancestral (interior) nodes. This can greatly affect the appearance of the tree. The vertical positions (these descriptions assume a a tree growing vertically) are not under your control except insofar as you specify the use or non-use of branch lengths. If you choose to change this option you will see the menu: | | | --- | | ``` Should interior node positions: be Intermediate between their immediate descendants, Weighted average of tip positions Centered among their ultimate descendants iNnermost of immediate descendants or so that tree is V-shaped (type I, W, C, N or V): ``` | The five methods (Intermediate, Weighted, Centered, Innermost, and V-shaped) are different horizontal positionings of the interior nodes. It will be helpful to you to try these out and see which you like best. Intermediate places the node halfway between its immediate descendants (horizontally), Weighted places it closer to that descendant who is closer vertically as well, and Centered centers the node below the horizontal positions of the tips that are descended from that node. You may want to choose that option that prevents lines from crossing each other. V-shaped is another option, one designed, if there are no branch lengths being used, to yield a v-shaped tree of regular appearance. With branch lengths it will not necessarily do so. "Innermost" is the most unusual option: it chooses a center for the tree, and always places interior nodes below the innermost of their immediate descendants. This leads to a tree that has vertical lines in the center, like a tree with a trunk. If the tree you are plotting has a full set of lengths, then when it is read in the node position option is automatically set to "intermediate", which is the setting with the least likelihood of lines in the tree crossing. If it does not have lengths the option is set to "V-shaped". If you change option B which tells the program whether to try to use the branch lengths, then the node position option will automatically be reset to the appropriate one of these defaults. This may be confusing if you do not realise that it is happening. F: Allows you to select the name of the Font that you will use for the species names. This is allowed for some of the plotter drivers (this menu item does not appear for the others). You can select the name of any font that is available for your plotter, for example "Courier-Bold" or "Helvetica". The label will then be printed using that font rather than being drawn line-by-line as it is in the default Hershey font. In the preview of the tree, the Hershey font is always used (which means that it may look different from the final font). The size of the characters in the species names is scaled according to the label heights you have selected in the menu, whether plotter fonts or the Hershey font are used. Note that for some plotter drivers (particular Xfig and PICT) fonts can be used only if the species labels are horizontal or vertical (at angles of 0 degrees or 90 degrees). M: The horizontal and vertical Margins in centimeters. You can enter new margins (you must enter new values for both horizontal and vertical margins, though these need not be different from the old values). For the moment I do not allow you to specify left and right margins separately, or top and bottom margins separately. In a future release I hope to do so. #: The number of pages per tree. Defaults to one, but if you need a physically large tree you may want to choose a larger number. For example, to make a big tree for a poster, choose a larger number of pages horizontally and vertically (the program will ask you for these numbers), get out your scissors and paste or tape, and go to work.

I recommend that you try all of these options (particularly if you can
preview the trees). It is of particular use to try combinations of the
style of tree (option S) with the different methods of placing
interior nodes (option A). You will find that a wide variety of
effects can be achieved.

I would appreciate suggestions for improvements in Drawgram, but please
be aware that the source code is already very large and I may not be
able to implement all suggestions.
