# Supplementary material for: Probabilistic Phylogenetic Inference with Insertions and Deletions
Source: PLoS Comput Biol. 2008 Sep 19;4(9):e1000172. doi: 10.1371/journal.pcbi.1000172 (PMC2527138; doi:10.1371/journal.pcbi.1000172)
Supplement: Dataset S1 — Supplemental Material (24.89 MB GZ) [file pcbi.1000172.s001.gz › erate-supplement-R2/src/phylip3.66-erate/doc/protdist.html]

 

protdist


version 3.66

# Protdist -- Program to compute distance matrix from protein sequences

© Copyright 1993, 2000-2006 by the University of Washington. Permission
is granted to copy
this document provided that no fee is charged for it and that this copyright
notice is not removed.

This program uses protein sequences to compute a distance matrix, under
four different models of amino acid replacement. It can also
compute a table of similarity between the amino acid sequences.
The distance for each
pair of species estimates the total branch length between the two species, and
can be used in the distance matrix programs Fitch, Kitsch or Neighbor. This
is an alternative to use of the sequence data itself in the
parsimony program Protpars.

The program reads in protein sequences and writes an output file containing
the distance matrix or similarity table. The five models of amino acid
substitution are
one which is based on the Jones, Taylor and Thornton (1992) model of
amino acid change, the PMB model (Veerassamy, Smith and Tillier, 2003)
which is derived from the Blocks database of conserved protein motifs,
the DCMut model (Kosiol and Goldman, 2005) based on the PAM matrices of
Margaret Dayhoff,
one due to
Kimura (1983, p.75) which approximates it based simply on the fraction of
similar amino acids, and one based on a model in which the amino acids are
divided up into groups, with change occurring based on the genetic code
but with greater difficulty of changing between groups. The program correctly
takes into account a variety of sequence ambiguities.

The five methods are:

(1) The Dayhoff PAM matrix. This uses the DCMut model (Kosiol and Goldman,
2005) which is a version of the PAM model of Margaret Dayhoff.
The PAM model is an empirical one that
scales probabilities of change from one amino acid to another in
terms of a unit which is an expected 1% change between two amino acid
sequences. The PAM 001 matrix is used to make a transition probability
matrix which allows prediction of the probability of changing from any
one amino acid to any other, and also predicts equilibrium amino acid
composition. The program assumes that these probabilities are correct
and bases its computations of distance on them. The distance that is
computed is scaled in units of expected fraction of amino acids
changed. This is a unit such that 1.0 is 100 PAM's.

(2) The Jones-Taylor-Thornton model. This is similar to the Dayhoff
PAM model, except that it is based on a recounting of the number of
observed changes in amino acids by Jones, Taylor, and Thornton (1992).
They used a much larger sample of protein sequences than did Dayhoff.
The distance is scaled in units of the expected fraction of amino acids
changed (100 PAM's). Because its sample is so much larger this
model is to be preferred over the original Dayhoff PAM model. It
is the default model in this program.

(3) The PMB (Probability Matrix from Blocks) model. This is derived using the
Blocks database of conserved protein motifs. It is described in
a paper by Veerassamy, Smith and Tillier (2003). Elisabeth Tillier
kindly made the matrices available for this model.

(4) Kimura's distance. This is a rough-and-ready distance formula for
approximating PAM distance by simply measuring the fraction of amino
acids, p, that differs between two sequences and computing the
distance as (Kimura, 1983)

     D = - loge ( 1 - p - 0.2 p2 ).

This is very quick to do but has some obvious limitations. It does not
take into account which amino acids differ or to what amino acids
they change, so some information is lost. The units of the distance
measure are fraction of amino acids differing, as also in the case of
the PAM distance. If the fraction of amino acids differing gets larger
than about 0.8541 the distance becomes infinite. Note that this can happen
with bootstrapped sequences even when the original sequences are below
this level of difference.

(5) The Categories distance. This is my own concoction. I imagined
a nucleotide sequence changing according to Kimura's 2-parameter model,
with the exception that some changes of amino acids are less likely than
others. The amino acids are grouped into a series of categories. Any
base change that does not change which category the amino acid is in is
allowed, but if an amino acid changes category this is allowed only a
certain fraction of the time. The fraction is called the "ease" and
there is a parameter for it, which is 1.0 when all changes are allowed
and near 0.0 when changes between categories are nearly impossible.

In this option I have allowed the user to select the Transition/Transversion
ratio, which of several genetic codes to use, and which categorization
of amino acids to use. There are three of them, a somewhat random sample:

(a): The George-Hunt-Barker (1988) classification of amino acids, (b): A classification provided by my colleague Ben Hall when I asked him for one, (c): One I found in an old "baby biochemistry" book (Conn and Stumpf, 1963), which contains most of the biochemistry I was ever taught, and all that I ever learned.

Interestingly enough, all of them are consisten with the same linear
ordering of amino acids, which they divide up in different ways. For the
Categories model I have set as default the George/Hunt/Barker classification
with the "ease" parameter set to 0.457 which is approximately the value
implied by the empirical rates in the Dayhoff PAM matrix.

The method uses, as I have noted, Kimura's (1980) 2-parameter model of DNA
change. The Kimura "2-parameter" model allows
for a difference between transition and transversion rates. Its transition
probability matrix for a short interval of time is:

```
              To:     A        G        C        T
                   ---------------------------------
               A  | 1-a-2b     a         b       b
       From:   G  |   a      1-a-2b      b       b
               C  |   b        b       1-a-2b    a
               T  |   b        b         a     1-a-2b
```

where *a* is *u dt*, the product of the rate of transitions per unit time and *dt*
is the length *dt* of the time interval, and *b* is *v dt*, the product of half the
rate of transversions (i.e., the rate of a specific transversion)
and the length dt of the time interval.

Each distance that is calculated is an estimate, from that particular pair of
species, of the divergence time between those two species. The Kimura
distance is straightforward to compute. The other two are considerably
slower, and they look at all positions, and find that distance which
makes the likelihood highest. This likelihood is in effect the length of
the internal branch in a two-species tree that connects these two
species. Its likelihood is just the product, under the model, of the
probabilities of each position having the (one or) two amino acids that
are actually found. This is fairly slow to compute.

The computation proceeds from an eigenanalysis (spectral decomposition)
of the transition probability matrix. In the case of the PAM 001 matrix
the eigenvalues and eigenvectors are precomputed and are hard-coded
into the program in over 400 statements. In the case of the Categories
model the program computes the eigenvalues and eigenvectors itself, which
will add a delay. But the delay is independent of the number of species
as the calculation is done only once, at the outset.

The actual algorithm for estimating the distance is in both cases a
bisection algorithm which tries to find the point at which the derivative
os the likelihood is zero. Some of the kinds of ambiguous amino acids
like "glx" are correctly taken into account. However, gaps are treated
as if they are unkown nucleotides, which means those positions get dropped
from that particular comparison. However, they are not dropped from the
whole analysis. You need not eliminate regions containing gaps, as long
as you are reasonably sure of the alignment there.

Note that there is an
assumption that we are looking at all positions, including those
that have not changed at all. It is important not to restrict attention
to some positions based on whether or not they have changed; doing that
would bias the distances by making them too large, and that in turn
would cause the distances
to misinterpret the meaning of those positions that
had changed.

The program can now correct distances for unequal rates of change at different
amino acid positions. This correction, which was introduced for DNA
sequences by Jin and Nei (1990), assumes that the distribution of rates
of change among amino acid positions follows a Gamma distribution. The
user is asked for the value of a parameter that determines the amount of
variation of rates among amino acid positions. Instead of the more
widely-known coefficient alpha, Protdist uses the coefficient of variation
(ratio of the standard deviation to the mean) of rates among amino acid
positions. . So if there is 20% variation in rates, the CV is
is 0.20. The square of the C.V. is also the reciprocal of the
better-known "shape parameter", alpha, of the Gamma
distribution, so in this case the shape parameter alpha = 1/(0.20\*0.20)
= 25. If you want to achieve a particular value of alpha, such as 10,
you will want to use a CV of 1/sqrt(10) = 1/3.162 = 0.3162.

In addition to the five distance calculations, the program can also
compute a table of similarities between amino acid sequences. These values
are the fractions of amino acid positions identical between the sequences.
The diagonal values are 1.0000. No attempt is made to count similarity
of nonidentical amino acids, so that no credit is given for having
(for example) different hydrophobic amino acids at the corresponding
positions in the two sequences. This option has been requested by many
users, who need it for descriptive purposes. It is not intended that
the table be used for inferring the tree.

## INPUT FORMAT AND OPTIONS

Input is fairly standard, with one addition. As usual the first line of the
file gives the number of species and the number of sites. There follows the
character W if the Weights option is being used.

Next come the species data. Each
sequence starts on a new line, has a ten-character species name
that must be blank-filled to be of that length, followed immediately
by the species data in the one-letter code. The sequences must either
be in the "interleaved" or "sequential" formats
described in the Molecular Sequence Programs document. The I option
selects between them. The sequences can have internal
blanks in the sequence but there must be no extra blanks at the end of the
terminated line. Note that a blank is not a valid symbol for a deletion.

After that are the lines (if any) containing the information for the
W option, as described below.

The options are selected using an interactive menu. The menu looks like this:

|  |
| --- |
| ``` Protein distance algorithm, version 3.6  Settings for this run:   P  Use JTT, PMB, PAM, Kimura, categories model?  Jones-Taylor-Thornton matrix   G  Gamma distribution of rates among positions?  No   C           One category of substitution rates?  Yes   W                    Use weights for positions?  No   M                   Analyze multiple data sets?  No   I                  Input sequences interleaved?  Yes   0                 Terminal type (IBM PC, ANSI)?  ANSI   1            Print out the data at start of run  No   2          Print indications of progress of run  Yes  Are these settings correct? (type Y or the letter for one to change) ``` |

The user either types "Y" (followed, of course, by a carriage-return)
if the settings shown are to be accepted, or the letter or digit corresponding
to an option that is to be changed.

The P option selects one of the five distance methods, or the
similarity table. It toggles among these
six methods. The default method, if none is specified, is the
Jones-Taylor-Thornton model. If the Categories distance is selected
another menu option, T, will appear allowing the user
to supply the Transition/Transversion ratio that should be assumed
at the underlying DNA level, and another one, C, which allows the
user to select among various nuclear and mitochondrial genetic codes.i
The transition/transversion ratio can be any number from 0.5 upwards.

The G option chooses Gamma distributed rates of evolution across amino
acid psoitions. The program will pronmpt you for the Coefficient of Variation
of rates. As is noted above, thi is 1/sqrt(alpha) if alpha is the more
familiar "shape coefficient" of the Gamma distribution. If the G option
is not selected, the program defaults to having no variation of rates
among sites.

The C option allows user-defined rate categories. The user is prompted
for the number of user-defined rates, and for the rates themselves,
which cannot be negative but can be zero. These numbers, which must be
nonnegative (some could be 0),
are defined relative to each other, so that if rates for three categories
are set to 1 : 3 : 2.5 this would have the same meaning as setting them
to 2 : 6 : 5.
The assignment of rates to
sites is then made by reading a file whose default name is "categories".
It should contain a string of digits 1 through 9. A new line or a blank
can occur after any character in this string. Thus the categories file
might look like this:

```
122231111122411155
1155333333444
```

If both user-assigned rate categories and Gamma-distributed rates
are allowed, the program assumes that the
actual rate at a site is the product of the user-assigned category rate
and the Gamma-distributed rate. This allows you to specify that
certain sites have higher or lower rates of change while also allowing the
program to allow variation of rates in addition to that.

The options M and 0 are the usual ones. They are described in the
main documentation file of this package. Option I is the same as in
other molecular sequence programs and is described in the documentation file
for the sequence programs.

The W (Weights) option is invoked in the usual way, with only weights 0
and 1 allowed. It selects a set of sites to be analyzed, ignoring the
others. The sites selected are those with weight 1. If the W option is
not invoked, all sites are analyzed.

## OUTPUT FORMAT

As the
distances are computed, the program prints on your screen or terminal
the names of the species in turn,
followed by one dot (".") for each other species for which the distance to
that species has been computed. Thus if there are ten species, the first
species name is printed out, followed by one dot, then on the next line
the next species name is printed out followed by two dots, then the
next followed by three dots, and so on. The pattern of dots should form
a triangle. When the distance matrix has been written out to the output
file, the user is notified of that.

The output file contains on its first line the number of species. The
distance matrix is then printed in standard
form, with each species starting on a new line with the species name, followed
by the distances to the species in order. These continue onto a new line
after every nine distances. The distance matrix is square
with zero distances on the diagonal. In general the format of the distance
matrix is such that it can serve as input to any of the distance matrix
programs.

If the similarity table is selected, the table that is produced is not
in a format that can be used as input to the distance matrix programs.
it has a heading, and the species names are also put at the tops of the
columns of the table (or rather, the first 8 characters of each species
name is there, the other two characters omitted to save space). There
is not an option to put the table into a format that can be read by
the distance matrix programs, nor is there one to make it into a table
of fractions of difference by subtracting the similarity values from 1.
This is done deliberately to make it more difficult for the use to
use these values to construct trees. The similarity values are
not corrected for multiple changes, and their use to construct trees
(even after converting them to fractions of difference) would be
wrong, as it would lead to severe conflict between the distant
pairs of sequences and the close pairs of sequences.

If the option to print out the data is selected, the output file will
precede the data by more complete information on the input and the menu
selections. The output file begins by giving the number of species and the
number of characters, and the identity of the distance measure that is
being used.

In the Categories model of substitution,
the distances printed out are scaled in terms of expected numbers of
substitutions, counting both transitions and transversions but not
replacements of a base by itself, and scaled so that the average rate of
change is set to 1.0. For the Dayhoff PAM and Kimura models the
distance are scaled in terms of the expected numbers of amino acid
substitutions per site. Of course, when a branch is twice as
long this does not mean that there will be twice as much net change expected
along it, since some of the changes may occur in the same site and overlie or
even reverse each
other. The branch lengths estimates here are in terms of the expected
underlying numbers of changes. That means that a branch of length 0.26
is 26 times as long as one which would show a 1% difference between
the protein (or nucleotide) sequences at the beginning and end of the
branch. But we
would not expect the sequences at the beginning and end of the branch to be
26% different, as there would be some overlaying of changes.

One problem that can arise is that two or more of the species can be so
dissimilar that the distance between them would have to be infinite, as
the likelihood rises indefinitely as the estimated divergence time
increases. For example, with the Kimura model, if the two sequences
differ in 85.41% or more of their positions then the estimate of divergence
time would be infinite. Since there is no way to represent an infinite
distance in the output file, the program regards this as an error, issues a
warning message indicating which pair of species are causing the problem, and
computes a distance of -1.0.

## PROGRAM CONSTANTS

The constants that are available to be changed by the user at the beginning
of the program include
"namelength", the length of species names in
characters, and "epsilon", a parameter which controls the accuracy of the
results of the iterations which estimate the distances. Making "epsilon"
smaller will increase run times but result in more decimal places of
accuracy. This should not be necessary.

The program spends most of its time doing real arithmetic. Any software or
hardware changes that speed up that arithmetic will speed it up by a nearly
proportional amount.

---

### TEST DATA SET

(Note that although these may look like DNA sequences, they are being
treated as protein sequences consisting entirely of alanine, cystine,
glycine, and threonine).

|  |
| --- |
| ```    5   13 Alpha     AACGTGGCCACAT Beta      AAGGTCGCCACAC Gamma     CAGTTCGCCACAA Delta     GAGATTTCCGCCT Epsilon   GAGATCTCCGCCC ``` |

---

### CONTENTS OF OUTPUT FILE (with all numerical options on )

(Note that when the numerical options are not on, the output file produced is
in the correct format to be used as an input file in the distance matrix
programs).

|  |
| --- |
| ```   Jones-Taylor-Thornton model distance  Name            Sequences ----            ---------  Alpha        AACGTGGCCA CAT Beta         ..G..C.... ..C Gamma        C.GT.C.... ..A Delta        G.GA.TT..G .C. Epsilon      G.GA.CT..G .CC   Alpha       0.000000  0.331834  0.628142  1.036660  1.365098 Beta        0.331834  0.000000  0.377406  1.102689  0.682218 Gamma       0.628142  0.377406  0.000000  0.979550  0.866781 Delta       1.036660  1.102689  0.979550  0.000000  0.227515 Epsilon     1.365098  0.682218  0.866781  0.227515  0.000000 ``` |
