# Supplementary material for: Probabilistic Phylogenetic Inference with Insertions and Deletions
Source: PLoS Comput Biol. 2008 Sep 19;4(9):e1000172. doi: 10.1371/journal.pcbi.1000172 (PMC2527138; doi:10.1371/journal.pcbi.1000172)
Supplement: Dataset S1 — Supplemental Material (24.89 MB GZ) [file pcbi.1000172.s001.gz › erate-supplement-R2/src/phylip3.66-erate/doc/seqboot.html]

seqboot


version 3.66

# Seqboot -- Bootstrap, Jackknife, or Permutation Resampling of Molecular Sequence, Restriction Site, Gene Frequency or Character Data

© Copyright 1991-2006 by the University of Washington.
Written by Joseph Felsenstein. Permission is granted to copy
this document provided that no fee is charged for it and that this copyright
notice is not removed.

Seqboot is a general bootstrapping and data set translation tool. It is intended to allow you to
generate multiple data sets that are resampled versions of the input data
set. Since almost all programs in the package can analyze these multiple
data sets, this allows almost anything in this package to be bootstrapped,
jackknifed, or permuted. Seqboot can handle molecular sequences,
binary characters, restriction sites, or gene frequencies. It
can also convert data sets between Sequential and Interleaved
format, and into the NEXUS format or into a new XML sequence alignment format.

To carry out a bootstrap (or jackknife, or permutation test) with some method
in the package, you may need to use three programs. First, you need to run
Seqboot to take the original data set and produce a large number of
bootstrapped or jackknifed data
sets (somewhere between 100 and 1000 is usually adequate).
Then you need to find the phylogeny estimate for
each of these, using the particular method of interest. For example, if
you were using Dnapars you would first run Seqboot and make a file with 100
bootstrapped data sets. Then you would give this file the proper name to
have it be the input file for Dnapars. Running Dnapars with the M (Multiple
Data Sets) menu choice and informing it to expect 100 data sets, you
would generate a big output file as well as a treefile with the trees from
the 100 data sets. This treefile could be renamed so that it would serve
as the input for Consense. When Consense is run the majority rule consensus
tree will result, showing the outcome of the analysis.

This may sound tedious, but the run of Consense is fast, and that of
Seqboot is fairly fast, so that it will not actually take any longer than
a run of a single bootstrap program with the same original data and the same
number of replicates. This is not very hard and allows bootstrapping or
jackknifing on many of the methods in
this package. The same steps are necessary with all of them. Doing things
this way some of the intermediate files (the tree file from the Dnapars
run, for example) can be used to summarize the results of the bootstrap in
other ways than the majority rule consensus method does.

If you are using the Distance Matrix programs, you will have to add one extra
step to this, calculating distance matrices from each of the replicate data
sets, using Dnadist or Gendist. So (for example) you would run Seqboot, then
run Dnadist using the output of Seqboot as its input, then run (say) Neighbor
using the output of Dnadist as its input, and then run Consense using the
tree file from Neighbor as its input.

The resampling methods available are:

- **The bootstrap.** Bootstrapping was invented by Bradley Efron in 1979,
  and its use in phylogeny estimation was introduced by me (Felsenstein, 1985b;
  see also Penny and Hendy, 1985).
  It involves creating a new data set by sampling *N* characters randomly
  with replacement, so that the resulting data set has the same size as the
  original, but some characters have been left out and others are duplicated.
  The random variation of the results from analyzing these bootstrapped
  data sets can be shown statistically to be typical of the variation that
  you would get from collecting new data sets. The method assumes that the
  characters evolve independently, an assumption that may not be realistic
  for many kinds of data.

  - **The partial bootstrap.**. This is the bootstrap where fewer than
    the full number of characters are sampled. The user is asked for the
    fraction of characters to be sampled. It is primarily useful in carrying out
    Zharkikh and Li's (1995) Complete And Partial Bootstrap method, and
    Shimodaira's (2002) AU method, both of which correct the bias of bootstrap
    P values.

    - **Block-bootstrapping.** One pattern of departure from indeopendence
      of character evolution is correlation of evolution in adjacent characters.
      When this is thought to have occurred, we can correct for it by samopling,
      not individual characters, but blocks of adjacent characters. This is
      called a block bootstrap and was introduced by Künsch (1989). If the
      correlations are believed to extend over some number of characters, you
      choose a block size, *B*, that is larger than this, and choose
      *N/B* blocks of size *B*. In its implementation here the
      block bootstrap "wraps around" at the end of the characters (so that if a
      block starts in the last  *B-1* characters, it continues by wrapping
      around to the first character after it reaches the last character). Note also
      that if you have a DNA sequence data set of an exon of a coding region, you
      can ensure that equal numbers of first, second, and third coding positions
      are sampled by using the block bootstrap with *B = 3*.

      - **Partial block-bootstrapping.** Similar to partial bootstrapping
        except sampling blocks rather than single characters.

        - **Delete-half-jackknifing.**. This alternative to the bootstrap involves
          sampling a random half of the characters, and including them in the data
          but dropping the others. The resulting data sets are half the size of the
          original, and no characters are duplicated. The random variation from
          doing this should be very similar to that obtained from the bootstrap.
          The method is advocated by Wu (1986). It was mentioned by me in my
          bootstrapping paper (Felsenstein, 1985b), and has been available for many
          years in this program as an option.
          Note that, for the
          present, block-jackknifing is not available,
          because I cannot figure out how to do it straightforwardly when the block size
          is not a divisor of the number of characters.

          - **Delete-fraction jackknifing.** Jackknifing is advocated by
            Farris et. al. (1996) but as deleting a fraction 1/e (1/2.71828). This
            retains too many characters and will lead to overconfidence in the
            resulting groups when there are conflicting characters. However it is
            made available here as an option, with the user asked to supply the fraction
            of characters that are to be retained.

            - **Permuting species within characters.** This method of resampling (well, OK,
              it may not be best to call it resampling) was introduced by Archie (1989)
              and Faith (1990; see also Faith and Cranston, 1991). It involves permuting the
              columns of the data matrix
              separately. This produces data matrices that have the same number and kinds
              of characters but no taxonomic structure. It is used for different purposes
              than the bootstrap, as it tests not the variation around an estimated tree
              but the hypothesis that there is no taxonomic structure in the data: if
              a statistic such as number of steps is significantly smaller in the actual
              data than it is in replicates that are permuted, then we can argue that there
              is some taxonomic structure in the data (though perhaps it might be just the
              presence of aa pair of sibling species).

              - **Permuting characters.** This simply permutes the order of the
                characters, the same reordering being applied to all species.
                For many methods of tree inference this will make no difference to the
                outcome (unless one has rates of evolution correlated among adjacent sites).
                It is included as a possible step in carrying out a permutation test of
                homogeneity of characters (such as the Incongruence Length Difference test).

                - **Permuting characters separately for each species.** This is a
                  method introduced by Steel, Lockhart, and Penny (1993) to permute data so as
                  to destroy all phylogenetic structure, while keeping the base composition of
                  each species the same as before. It shuffles the character order separately
                  for each species.

                  - **Rewriting.** This is not a resampling or permutation method -- it
                    simply rewrites the data set into a different format. That format
                    can be the PHYLIP format. For molecular sequences and discrete morphological
                    character it can also be the NEXUS format. For molecular sequences one
                    other format is available, a new (and nonstandard) XML format of our own
                    devising. When the PHYLIP format is chosen the data set is coverted between
                    Interleaved and Sequential format. If it was read in as Interleaved sequences,
                    it will be written out as Sequential format, and vice versa. The NEXUS
                    format for molecular sequences is always written as interleaved sequences.
                    The XML format is different from (though similar to) a number of other
                    XML sequence alignment formats. An example will be found below.
                    Here is a table to links to those other XML alignment formats:

                    |  |  |  |
                    | --- | --- | --- |
                    | Andrew Rambaut's  BEAST XML format | `http://evolve.zoo.ox.ac.uk/beast/introXML.html`  and  `http://evolve.zoo.ox.ac.uk/beast/reference/index.html` | A format for alignments. There  is also a format for phylogenies  described there. |
                    | MSAML | `http://xml.coverpages.org/msaml-desc-dec.html` | Defined by Paul Gordon of  University of Calgary. See his  big list of molecular biology  XML projects. |
                    | BSML | `http://www.bsml.org/resources/default.asp` | Bioinformatic Sequence  Markup Language  includes a multiple sequence  alignment XML format |

The data input file is of standard form for molecular sequences (either in
interleaved or sequential form), restriction sites, gene frequencies, or
binary morphological characters.

When the program runs it first asks you for a random number seed. This should
be an integer greater than zero (and probably less than 32767) and which is
of the form 4n+1, that is, it leaves a remainder of 1 when divided by 4. This
can be judged by looking at the last two digits of the integer (for instance
7651 is not of form 4n+1 as 51, when divided by 4, leaves the remainder 3).
The random number seed is used to start the random number generator.
If the randum number seed is not odd, the program will request it again.
Any odd number can be used, but may result in a random number sequence that
repeats itself after less than the full one billion numbers. Usually this
is not a problem. As the random numbers appear to be unpredictable,
there is no such thing as a "good" seed -- the numbers produced from one
seed are statistically indistinguishable from those produced by another, and
it is not true that the numbers produced from one seed (say 4533) are similar
to those produced from a nearby seed (say 4537).

Then the program shows you a menu to allow you to choose options. The menu
looks like this:

|  |
| --- |
| ``` Bootstrapping algorithm, version 3.6  Settings for this run:   D      Sequence, Morph, Rest., Gene Freqs?  Molecular sequences   J  Bootstrap, Jackknife, Permute, Rewrite?  Bootstrap   %    Regular or altered sampling fraction?  regular   B      Block size for block-bootstrapping?  1 (regular bootstrap)   R                     How many replicates?  100   W              Read weights of characters?  No   C                Read categories of sites?  No   S     Write out data sets or just weights?  Data sets   I             Input sequences interleaved?  Yes   0      Terminal type (IBM PC, ANSI, none)?  ANSI   1       Print out the data at start of run  No   2     Print indications of progress of run  Yes    Y to accept these or type the letter for one to change ``` |

The user selects options by typing one of the letters in the left column,
and continues to do so until all options are correctly set. Then the
program can be run by typing Y.

It is important to select the correct data type (the D selection). Each
time D is typed the program will change data type, proceeding successively
through Molecular Sequences, Discrete Morphological Characters, Restriction
Sites, and Gene Frequencies. Some of these will cause additional entries
to appear in the menu. If Molecular Sequences or Restriction Sites settings
and chosen the I (Interleaved)
option appears in the menu (and as Molecular Sequences are also the default,
it therefore appears in the first menu). It is the usual
I option discussed in the Molecular Sequences document file and in the main
documentation files for the package, and is on by default.

If the Restriction Sites option is chosen the menu option E appears, which
asks whether the input file contains a third number on the first line of
the file, for the number of restriction enzymes used to detect these sites.
This is necessary because data sets for Restml need this third number, but
other programs do not, and Seqboot needs to know what to expect.

If the Gene Frequencies option is chosen an menu option A appears which allows
the user to specify that all alleles at each locus are in the input file.
The default setting is that one allele is absent at each locus.
Note that for sampling methods such as the bootstrap and jackknife,
whole loci are sampled, not individual alleles.

The J option allows the user to select Bootstrapping, Delete-Half-Jackknifing,
the Archie-Faith permutation of species within characters, permutation of
character order, shuffling character order separately within each species,
or Rewriting. It changes
successively among these each time J is typed.

The P menu option appears if the data are molecular
sequences and the J option is used to choose the Rewrite option. It
gives you the choice between our normal PHYLIP format or a new (and
nonstandard) XML sequence alignment format. This encloses the alignment
between <ALIGNMENT> ... </ALIGNMENT> tags. Each sequence
between <SEQUENCE> ... </SEQUENCE> tags, has a TYPE
attribute of the sequence which is either "dna", "rna" or "protein".
This is set by default to "dna" but can be changed by the
user in an S Sequence type menu option. Each
sequence has its name, enclosed between <NAME> ... </NAME>
tags, and the data itself, enclosed between <DATA> ... </DATA>
tags. The XML option is not available unless the data are molecular
sequences. It is a new format -- no programs yet read it.
In other cases the P menu option does not appear and the
PHYLIP output format is assumed. Here is a simple example of this
XML sequence alignment format, for the (silly) data set used in
our main documentation file:

|  |
| --- |
| ``` <alignment>    <sequence type="dna">       <name>Archaeopt</name>       <data>CGATGCTTAC CGC</data>    </sequence>     <sequence type="dna">       <name>Hesperorni</name>       <data>CGTTACTCGT TGT</data>    </sequence>     <sequence type="dna">       <name>Baluchithe</name>       <data>TAATGTTAAT TGT</data>    </sequence>     <sequence type="dna">       <name>B. virgini</name>       <data>TAATGTTCGT TGT</data>    </sequence>     <sequence type="dna">       <name>Brontosaur</name>       <data>CAAAACCCAT CAT</data>    </sequence>     <sequence type="dna">       <name>B.subtilis</name>       <data>GGCAGCCAAT CAC</data>    </sequence>  </alignment> ``` |

For the gene frequencies and restriction sites data types, this Rewrite
option does not change the data set. The option will be useful mostly to
write the data out in a standard format, in cases where the input file
is messy-looking.

The B option selects the Block Bootstrap. When you select option B the program
will ask you to enter the block length. When the block length is 1,
this means that we are doing regular bootstrapping rather than
block-bootstrapping.

The % option allows the user control over what fraction of the characters
are sampled in the bootstrap and jackknife methods. Normally the
bootstrap samples a number of times equal to the number of characters,
and the jackknife samples half that number. This option permits you to
specify a smaller fraction of characters to be sampled. Note that doing
so is "statistically incorrect", but it is available here for whatever other
purposes you may have in mind. Note that the fraction you will be asked to
enter is the fraction of characters sampled, not the fraction left out.
If you specify 100 as the fraction of sites retained and are using
the jackknife, the data set
will simply be rewritten. Note (as mentioned below) that this can be
used together with the W (Weights) option to rewrite a data set while
omitting a particular set of sites.

The R option allows the user to set the number of replicate data sets.
This defaults to 100. Most statisticians would be happiest with 1000 to
10,000 replicates in a bootstrap, but 100 gives a rough picture. You
will have to decide this based on how long a running time of the
tree programs you are willing to tolerate. (The time needed to do the
sampling in this program is not much of an issue).

The W (Weights) option allows weights to be read
from a file whose default name is "weights". The weights
follow the format described in the main documentation file.
Weights can only be 0 or 1, and act to select
the characters (or sites) that will be used in the resampling, the others
being ignored and always omitted from the output data sets.
If you use W together with the S (just weights)
option, you write a file of weights (whose default name is "outweights").
In that file, any character whose original weight is 0 will have weight
0, the other weights varying according to the resampling. Note that if
you write out data sets rather than weights (not using the S option),
this output weights file is not written, as the characters are
written different numbers of times in the data output file
Note that with restriction sites, the weights are not used by
some of the programs. Writing out files of weights will not be
helpful with those programs. For the moment, with all gene frequencies
programs the weights are also not used.

Note that it is possible to use Seqboot to rewrite a data set while
omitting certain sites. This can be done, not with the rewrite choice in
option J, but with its jackknife choice. Choose the delete-half
jackknife, but then use the % option to set the fraction of sites
sampled to 100%. Also use the W option to read a set of weights that
select which sites to retain (those with weights 1 instead of 0).
Use the R option to set the number of replicates to 1. The program will
write one data set, with all the sites that have weights 1, in order.

The C (Categories) option can be used with molecular sequence programs to
allow assignment of sites or amino acid positions to user-defined rate
categories. The assignment of rates to
sites is then made by reading a file whose default name is "categories".
It should contain a string of digits 1 through 9. A new line or a blank
can occur after any character in this string. Thus the categories file
might look like this:

```
122231111122411155
1155333333444
```

The only use of the Categories information in Seqboot is that they
are sampled along with the sites (or amino acid positions) and are
written out onto a file whose default name is "outcategories",
which has one set of categories information for each bootstrap
or jackknife replicate.

In the discrete characters data type, three more options appear in the
menu. These are N (aNcestors), X (miXture of methods), and F (Factors)
options. They
may be useful with the program Mix, which allows input of ancestors
information and information specifying the mixture of parsimony methods
to be used. Factors information is also read and used by
programs Move, Dolmove, and Clique, in calculating how many multistate
characters are compatible with a tree.
The mixture, ancestors, and factors information for the characters
are specified in input files whose default names are
"ancestors", "mixture", and "factors". Seqboot produces output files that
properly
reflect what the resampling implies for these files. The corresponding
output files have default file names "outancestors", "outmixture", and
"outfactors".

For futher description of the mixture, ancestors, and factors file
formats and contents
see the Discrete Characters Programs documentation file.

The S option is a particularly important one. It is used whether to
produce multiple output files or multiple weights. If your
data set is large, a file with (say) 1000 such data sets can be very
large and may use up too much space on your system. If you choose
the S option, the program will instead produce a weights file with
multiple sets of weights. The default name of this file is "outweights".
Except for some programs that cannot handle multiple sets of
weights,
PHYLIP programs have an M (multiple data sets) option that asks the
user whether to use multiple data sets or multiple sets of weights.
If the latter is selected when running those programs, they
read one data set, but analyze it multiple times, each time reading a new
set of weights. As both bootstrapping and jackknifing can be thought of
as reweighting the characters, this accomplishes the same thing (the
multiple weights option is not available for the various kinds of permutation).
As the file with multiple sets of weights is much smaller than a file with
multiple data sets, this can be an attractive way to save file space.
When multiple sets of weights is chosen, they reflect the sampling as
well as any set of weights that was read in, so that you can use
Seqboot's W option as well.

The 0 (Terminal type) option is the usual one.

## Saving time by combining results of separate runs

Often runs of distance programs, or of phylogeny programs, on large numbers
of bootstrap replicates are very time-consuming. If you have multiple
computers, you can save time by splitting up these runs among multiple
machines. For example, if you have 1000 replicate data sets (or weights) from
bootstrapping, you could divide these into ten files of 100 data sets (or
you could simply use Seqboot ten times with different random number seeds). If
these are run on ten separate computers, the execution time is speeded up
by as much as a factor of 10. Each input file of 100 data sets results in
an output tree file. These can be concatenated end-to-end using a word
processor program or using a command such as the Unix/Linux cat
command. Make sure that these files are not turned into Microsoft Word
format when this is done. The consensus tree program Consense will
hande the concatenated tree file properly.

If a distance matrix method is being used, you can also produce the distance
matrices on different machines, and concatenate them end-to-end to
produce an input file of distance matrices for Fitch, Kitsch, or Neighbor.
This is particularly relevant for Neighbor, which in most cases makes trees more
quickly than the distance matrices can be produced.

## Input File

The data files read by Seqboot are the standard ones for the various kinds of
data. For molecular sequences the sequences may be either interleaved or
sequential, and similarly for restriction sites. Restriction sites data
may either have or not have the third argument, the number of restriction
enzymes used. Discrete morphological
characters are always assumed to be in sequential format. Gene frequencies
data start with the number of species and the number of loci, and then
follow that by a line with the number of alleles at each locus. The data for
each locus may either have one entry for each allele, or omit one allele at
each locus. The details of the formats are given in the main documentation
file, and in the documentation files for the groups of programs.

## Output

The output file will contain the data sets generated by the resampling
process. Note that, when Gene Frequencies data is used or when
Discrete Morphological characters with the Factors option are used,
the number of characters in each data set may vary. It may also vary
if there are an odd number of characters or sites and the Delete-Half-Jackknife
resampling method is used, for then there will be a 50% chance of choosing
(n+1)/2 characters and a 50% chance of choosing (n-1)/2 characters.

The Factors option causes the characters to be resampled together. If
(say) three adjacent characters all have the same factors characters, so
that they all are understood to be recoding one multistate character, they
will be resampled together as a group. WARNING: at the moment the Factors
option does not work properly together with the Permute Characters option,
so I urge you not to try that combination of options.

The order of species in the data sets in the output file will vary
randomly. This is a precaution to help the programs that analyze these data
avoid any result which is sensitive to
the input order of species from showing up repeatedly
and thus appearing to have evidence in its favor.

The numerical options 1 and 2 in the menu also affect the output file.
If 1 is chosen (it is off by default) the program will print the original
input data set on the output file before the resampled data sets. I cannot
actually see why anyone would want to do this. Option 2 toggles the
feature (on by default) that prints out up to 20 times during the resampling
process a notification that the program has completed a certain number of
data sets. Thus if 100 resampled data sets are being produced, every 5
data sets a line is printed saying which data set has just been completed.
This option should be turned off if the program is running in background and
silence is desirable. At the end of execution the program will always (whatever
the setting of option 2) print
a couple of lines saying that output has been written to the output file.

## Size and Speed

The program runs moderately quickly, though more slowly when the Permutation
resampling method is used than with the others.

---

### TEST DATA SET

|  |
| --- |
| ```     5    6 Alpha     AACAAC Beta      AACCCC Gamma     ACCAAC Delta     CCACCA Epsilon   CCAAAC ``` |

---

### CONTENTS OF OUTPUT FILE

(If Replicates are set to 10 and seed to 4333)

|  |
| --- |
| ```     5     6 Alpha      ACAAAC Beta       ACCCCC Gamma      ACAAAC Delta      CACCCA Epsilon    CAAAAC     5     6 Alpha      AAAACC Beta       AACCCC Gamma      CCAACC Delta      CCCCAA Epsilon    CCAACC     5     6 Alpha      ACAAAC Beta       ACCCCC Gamma      CCAAAC Delta      CACCCA Epsilon    CAAAAC     5     6 Alpha      ACCAAA Beta       ACCCCC Gamma      ACCAAA Delta      CAACCC Epsilon    CAAAAA     5     6 Alpha      ACAAAC Beta       ACCCCC Gamma      ACAAAC Delta      CACCCA Epsilon    CAAAAC     5     6 Alpha      AAAACA Beta       AAAACC Gamma      AAACCA Delta      CCCCAC Epsilon    CCCCAA     5     6 Alpha      AAACCC Beta       CCCCCC Gamma      AAACCC Delta      CCCAAA Epsilon    AAACCC     5     6 Alpha      AAAACC Beta       AACCCC Gamma      AAAACC Delta      CCCCAA Epsilon    CCAACC     5     6 Alpha      AAAAAC Beta       AACCCC Gamma      CCAAAC Delta      CCCCCA Epsilon    CCAAAC     5     6 Alpha      AACCAC Beta       AACCCC Gamma      AACCAC Delta      CCAACA Epsilon    CCAAAC ``` |
