# Supplementary material for: Probabilistic Phylogenetic Inference with Insertions and Deletions
Source: PLoS Comput Biol. 2008 Sep 19;4(9):e1000172. doi: 10.1371/journal.pcbi.1000172 (PMC2527138; doi:10.1371/journal.pcbi.1000172)
Supplement: Dataset S1 — Supplemental Material (24.89 MB GZ) [file pcbi.1000172.s001.gz › erate-supplement-R2/src/phylip3.66-erate/doc/drawtree.html]

drawtree


version 3.66

# Drawtree

© Copyright 1990-2006 by The University of
Washington. Written by Joseph Felsenstein. Permission is granted to copy
this document provided that no fee is charged for it and that this copyright
notice is not removed.

Drawtree interactively plots an unrooted tree diagram, with many options
including orientation of tree and branches, label sizes and angles, margin
sizes. Particularly if you can use your computer screen to
preview the plot, you can very effectively adjust the details of the plotting
to get just the kind of plot you want.

To understand the working of Drawgram and Drawtree, you should first
read the Tree Drawing Programs web page
in this documentation.

As with Drawgram, to run Drawtree you need a compiled copy of the
program, a font file, and a tree file. The tree file has a default name
of intree. The font file has a default name of "fontfile". If there is
no file of that name, the program will ask you for the name of a font file
(we provide ones that have the names font1 through font6).
Once you decide on a favorite one of these, you could make a copy of it
and call it fontfile, and it will then be used by default.
Note that the program will get confused if the input tree file has the number
of trees on the first line of the file, so that numbr may have to be removed.

Once these choices have been made you will see the central menu of the
program, which looks like this:

|  |
| --- |
| ``` Unrooted tree plotting program version 3.6  Here are the settings:   0  Screen type (IBM PC, ANSI)?  ANSI  P       Final plotting device:  Postscript printer  V           Previewing device:  X Windows display  B          Use branch lengths:  (no branch lengths available)  L             Angle of labels:  branch points to Middle of label  R            Rotation of tree:  90.0  I     Iterate to improve tree:  Equal-Daylight algorithm  D  Try to avoid label overlap?  No  S      Scale of branch length:  Automatically rescaled  C   Relative character height:  0.3333  F                        Font:  Times-Roman  M          Horizontal margins:  1.65 cm  M            Vertical margins:  2.16 cm  #           Page size submenu:  one page per tree   Y to accept these or type the letter for one to change ``` |

These are the settings that control the appearance of the tree, which
has already been read in. You can either accept these as is, in which
case you would answer Y to the question and press the Return or Enter
key, or you can answer N if you want to change one, or simply type the
character corresponding to the one you want to change (if you answer N it
will just immediately ask you for that number anyway).

For a first run, particularly if previewing is available, you might accept
these default values and see what the result looks like. The program
will then tell you it is about to preview the tree and ask you to press
Return or Enter when you are ready to see this (you will probably have
to press it twice). If you are on a Windows system (and have its graphics
selected as your previewing option), on a Unix or Linux system and are
using X windows for previewing, or are on a Macintosh system, a new
window will open with the preview in it. If you are using the Tektronix
preview option the preview will appear in the window where the menu was.

On X Windows, Macintosh, and Windows you can resize the preview window,
though for some of these you may have to ask the system to redraw the
preview to see it at the new window size.

Once you are finished looking at the preview, you will want to
specify whether the program should make the final plot or change some of
the settings. This is done differently on the different previews:

- In **X Windows** you should make the menu window the active
  window. You may need to move the mouse over it, or click in it, or
  click on its top bar. You do not need to try to close the preview
  window yourself, and usually if you do this will cause trouble.- In **Windows** use the File menu in the preview window
    and choose either the Change Parameters menu item, or if
    you are ready to make the final plot, choose the Plot menu item.- On a **Macintosh** system, you can simply use the little box in
      the corner of the preview window to close it. The text window for the
      menu will then be active.- In **PC graphics** press on the Enter key. The screen with the
        preview should disappear and the settings menu reappear.- With a **Tektronix** preview, you may need to change your screen
          from a Tektronix-compatible mode to see the menu again.

Except with the Macintosh preview, the program will
now ask you if the tree is now ready to be plotted. If you answer Y (for
Yes) (or choose this option in the File menu of the preview
window in the case of Windows) the program will usually write a plot file
(with some plot options it will draw the tree on the screen). Then it will
terminate.

But if you do not say that you are ready to plot the tree,
it will go back to the above menu, allow
you to change more options, and go through the whole process again. The
easiest way to learn the meaning of the options is to try them,
particularly if previewing is available. Below I will describe them one
by one; you may prefer to skip reading this unless you are puzzled about
one of them.

## THE OPTIONS

O: This is an option that allows you to change the menu window to be an ANSI terminal or an IBM PC terminal. Generally you will not want to change this. P: This allows you to choose the Plotting device or file format. We have discussed the possible choices in the draw programs documentation web page. V: This allows you to change the type of preView window (or even turn off previewing. We have discussed the different possible choices in the draw programs documentation web page. B: Whether the tree has Branch lengths that are being used in the diagram. If the tree that was read in had a full set of branch lengths, it will be assumed as a default that you want to use them in the diagram, but you can specify that they are not to be used. If the tree does not have a full set of branch lengths then this will be indicated, and if you try to use branch lengths the program will refuse to allow you to do so. Note that there is no way to use negative branch lengths, so Drawtree automatically takes their absolute values, and thus will plot a branch that has length -0.1 as if it has length 0.1. L: The angle of the Labels. Initially the branches connected to the tips will point to the middles of the labels. If you want to change the way the labels are drawn, the program will offer you a choice between Fixed, Middle, Radial, and Along as the ways the angles of the labels are to be determined. If you choose Fixed (the default), you will be asked if you want labels to be at some fixed angle, between 90.0 and -90.0 degrees, you can specify that. You may have to try different angles to find one that keeps the labels from colliding: I have not guarded against this. However there are additional options. The other systems for determining angles of labels are Middle (M), Radial (R) and Along (A). Middle has the branch connected to that tip point to the midpoint of the label. Radial indicates that the labels are all aligned to as to point toward the root node of the tree. Along aligns them to have the same angle as the branch connected to that tip. This is particularly likely to keep the labels from colliding, but it may give a misleading impression that the final branch is long. Note that with the Radial option, if you do not like the point from which the labels appear to radiate, you might try re-rooting the tree (option 7). R: The rotation of the tree. This is initially 90.0 degrees. The angle is read out counterclockwise from the right side of the tree, so that increasing this angle will rotate the tree counterclockwise, and decreasing it will rotate it clockwise. The meaning of this angle is explained further under option A. As you rotate the tree, the appearance (and size) may change, but the labels will not rotate if they are drawn at a Fixed angle. A: The Angle through which the tree is plotted. This is by default 360.0 degrees. The tree is in the shape of an old-fashioned hand fan. The tree fans out from its root node, each of the subtrees being allocated part of this angle, a part proportional to how many tips the subtree contains. If the rotation of the tree is (say) 90.0 degrees (the default under option R), the fan starts at +270 degrees and runs clockwise around to -90 degrees (i.e., it starts at the bottom of the plot and runs clockwise around until it returns to the bottom. Thus the center of the fan runs from the root upwards (which is why we say it is rotated to 90.0 degrees). By changing option R we can change the direction of the fan, and by changing option A we can change the width of the fan without changing its center line. If you want the tree to fan out in a semicircle, a value of a bit greater than 180 degrees would be appropriate, as the tree will not completely fill the fan. Note that using either of the iterative improvement methods mentioned below is impossible if the angle is not 360 degrees. I: Whether the tree angles will be Iteratively improved. There are three methods available: Equal Arc: This method, invented by Christopher Meacham in PLOTREE, the predecessor to this program, starts from the root of the tree and allocates arcs of angle to each subtree proportional to the number of tips in it. This continues as one moves out to other nodes of the tree and subdivides the angle allocated to them into angles for each of that node's dependent subtrees. This method is fast, and never results in lines of the tree crossing. It is the method used to make a starting tree for the other two methods. Equal Daylight: This iteratively improves an initial tree by successively going to each interior node, looking at the subtrees (often there are 3 of them) visible from there, and swinging them so that the arcs of "daylight" visible between them are equal. This is not as fast as Equal Arc but should never result in lines crossing. It gives particularly good-looking trees, and it is the default method for this program. It will be described in a future paper by me. This method has also been licensed to David Swofford for use in his program PAUP\* N-Body: This assumes that there are electrical charges located at the ends of all the branches, and that they repel each other with a force that varies (as electrical repulsion would) as the inverse square of the distance between them. The tree adjusts its shape until the forces balance. This can be computationally slow, and can result in lines crossing. I find the trees inferior to Equal Daylight, but it is worth a try. D: Whether the program tries to avoiD overlap of the labels. We have left this off by default, because it is a rather feeble option that is frequently unsuccessful, and often make the trees look wierd. Nevertheless it may be worth a try. S: On what Scale the branch lengths will be translated into distances on the output device. Note that when branch lengths have not been provided, there are implicit branch lengths of 1.0 per branch. This option will toggle back and forth between automatic adjustment of branch lengths so that the diagram will just fit into the margins, and you specifying how many centimeters there will be per unit branch length. This is included so that you can plot different trees to a common scale, showing which ones have longer or shorter branches than others. Note that if you choose too large a value for centimeters per unit branch length, the tree will be so big it will overrun the plotting area and may cause failure of the diagram to display properly. Too small a value will cause the tree to be a nearly invisible dot. C: The Character height, measured as a fraction of a quantity which is the horizontal space available for the tree, divided by one less than the number of tips. You need not worry about exactly what this is: you can always change the value (which is initially 0.3333) to make the labels larger or smaller. On output devices where line thicknesses can be varied, the thickness of the tree lines will automatically be adjusted to be proportional to the character height, which is an additional reason you may want to change character height. F: Allows you to select the name of the Font that you will use for the species names. This is allowed for some of the plotter drivers (this menu item does not appear for the others). You can select the name of any font that is available for your plotter, for example "Courier-Bold" or "Helvetica". The label will then be printed using that font rather than being drawn line-by-line as it is in the default Hershey font. In the preview of the tree, the Hershey font is always used (which means that it may look different from the final font). The size of the characters in the species names is scaled according to the label heights you have selected in the menu, whether plotter fonts or the Hershey font are used. Note that for some plotter drivers (particular Xfig and PICT) fonts can be used only if the species labels are horizontal or vertical (at angles of 0 degrees or 90 degrees). M: The horizontal and vertical Margins in centimeters. You can enter new margins (you must enter new values for both horizontal and vertical margins, though these need not be different from the old values). For the moment I do not allow you to specify left and right margins separately, or top and bottom margins separately. In a future release I hope to do so. G: If iterative improvement is not turned on in option I (so that we are employing the Equal Arc method), this option appears in the menu. It controls whether the angles of lines will be "regularized". Regularization is off by default. It takes the angles of the branches coming out from each node, and changes them so that they are "rounded off". This process (which I will not fully describe) will make the lines vertical if they are close to vertical, horizontal if they are close to horizontal, 45 degrees if they are close to that, and so on. It will lead to a tree in which angles look very regular. You may or may not want that. If you are unhappy with the appearance of the tree when using this option, you could try rotating it slightly (option R) as that may cause some branches to change their angle by a large amount, by having the angles be "rounded off" to a different value. #: The number of pages per tree. Defaults to one, but if you need a physically large tree you may want to choose a larger number. For example, to make a big tree for a poster, choose a larger number of pages horizontally and vertically (the program will ask you for these numbers), get out your scissors and paste or tape, and go to work.

I recommend that you try all of these options (particularly if you can
preview the trees). It is of particular use to try trees with different
iteration methods (option I) and
with regularization (option G).
You will find that variety of effects can be achieved.

I would appreciate suggestions for improvements in Drawtree, but please
be aware that the source code is already very large and I may not be
able to implement all suggestions.
