# Supplementary material for: Probabilistic Phylogenetic Inference with Insertions and Deletions
Source: PLoS Comput Biol. 2008 Sep 19;4(9):e1000172. doi: 10.1371/journal.pcbi.1000172 (PMC2527138; doi:10.1371/journal.pcbi.1000172)
Supplement: Dataset S1 — Supplemental Material (24.89 MB GZ) [file pcbi.1000172.s001.gz › erate-supplement-R2/src/phylip3.66-erate/doc/factor.html]

factor


version 3.66

# Factor - Program to factor multistate characters.

© Copyright 1986-2006 by The University of Washington. Written by
Christopher Meacham and Joseph Felsenstein. Permission is granted
to copy this document provided that no fee is charged for it and that this
copyright notice is not removed.

This program factors a data set that contains multistate
characters, creating a data set consisting entirely of binary (0,1)
characters that, in turn, can be used as input to any of the other
discrete character programs in this package, except for PARS.
Besides this primary
function, Factor also provides an easy way of deleting characters from a
data set. The input format for Factor is very similar to the input
format for the other discrete character programs except for the
addition of character-state tree descriptions.

Note that this program has no way of converting an unordered multistate
character into binary characters.
Fortunately, PARS has joined the package, and it enables unordered
multistate characters, in which any state can change to any other in
one step, to be analyzed with parsimony.

Factor is really for a different case, that in which there are
multiple states related on a "character state tree", which specifies
for each state which other states it can change to. That graph of
states is assumed to be a tree, with no loops in it.

The first line of the input file should contain the number of
species and the number of multistate characters. This
first line is followed by the lines describing the character-state
trees, one description per line. The species information constitutes
the last part of the file. Any number of lines may be used for a single
species.

## FIRST LINE

The first line is free format with the number of species first,
separated by at least one blank (space) from the number of multistate
characters, which in turn is separated by at least one blank from the
options, if present.

## OPTIONS

The options are selected from a menu that looks like this:

|  |
| --- |
| ``` Factor -- multistate to binary recoding program, version 3.6  Settings for this run:   A      put ancestral states in output file?  No   F   put factors information in output file?  No   0       Terminal type (IBM PC, ANSI, none)?  (none)   1      Print indications of progress of run  Yes  Are these settings correct? (type Y or the letter for one to change) ``` |

The options particular to this program are:

A: Choosing the A (Ancestors) options toggles on and off the setting that causes a line to be written in the ancestors output file that describes the states of the ancestor as indicated by the character-state tree descriptions (see below). If the ancestral state is not specified by a particular character-state tree, a "?" signifying an unknown character state will be written. The multistate characters are factored in such a way that the ancestral state in the factored data set will always be "0". F: Choosing the F (Factors) option toggles on and off a setting that will cause a factors output file to be written (its default file name is "factors"). The line in this file will indicate to other programs which factors came from the same multistate character. Of the programs currently in the package only Seqboot, Move, and Dolmove use this information.

## CHARACTER-STATE TREE DESCRIPTIONS

The character-state trees are described in free format. The
character number of the multistate character is given first followed
by the description of the tree itself. Each description must be
completed on a single line. Each character that is to be factored must
have a description, and the characters must be described in the order
that they occur in the input, that is, in numerical order.

The tree is described by listing the pairs of character states that
are adjacent to each other in the character-state tree. The two
character states in each adjacent pair are separated by a colon (":").
If character fifteen has this character state tree for possible states
"A", "B", "C", and "D":

```
                         A ---- B ---- C
                                |
                                |
                                |
                                D
```

then the character-state tree description would be

```
                        15  A:B B:C D:B
```

Note that either symbol may appear first. The ancestral state is
identified, if desired, by putting it "adjacent" to a period. If we
wanted to root character fifteen at state C:

```
                         A <--- B <--- C
                                |
                                |
                                V
                                D
```

we could write

```
                      15  B:D A:B C:B .:C
```

Both the order in which the pairs are listed and the order of the
symbols in each pair are arbitrary. However, each pair may only appear
once in the list. Any symbols may be used for a character state in the
input except the character that signals the connection between two states (in
the distribution copy this is set to ":"), ".", and, of course, a
blank. Blanks are ignored
completely in the tree description so that even B:DA:BC:B.:C or
B : DA : BC : B. : C would be equivalent to the above example.
However, at least one blank must separate the character number from the
tree description.

## DELETING CHARACTERS FROM A DATA SET

If no description line appears in the input for a particular
character, then that character will be omitted from the output. If the
character number is given on the line, but no character-state tree is
provided, then the symbol for the character in the input will be copied
directly to the output without change. This is useful for characters
that are already coded "0" and "1". Characters can be deleted from a
data set simply by listing only those that are to appear in the output.

## TERMINATING THE LIST OF TREE DESCRIPTIONS

The last character-state tree description should be followed by a
line containing the number "999". This terminates processing of the
trees and indicates the beginning of the species information.

## SPECIES INFORMATION

The format for the species information is basically identical to
the other discrete character programs. The first ten character positions
are allotted to the species name (this value may be changed by altering
the value of the constant nmlngth at the beginning of the program). The
character states follow and may be continued to as many lines as
desired. There is no current method for indicating polymorphisms. It is
possible to either put blanks between characters or not.

There is a method for indicating uncertainty about states. There is
one character value that stands for "unknown". If this appears in
the input data then "?" is written out in all the corresponding
positions in the output file. The character value that designates
"unknown" is given in the constant unkchar at the beginning of the
program, and can be changed by changing that constant. It is set to
"?" in the distribution copy.

## OUTPUT

The first line of output will contain the number of species and
the number of binary characters in the factored data set.
The factored characters will be written for each species in the format
required for input by the other discrete programs in the package. The
maximum length of the output lines is 80 characters, but this maximum
length can be changed prior to compilation.

If the A (Ancestors) option was chosen, an output file whose default name
is ancestors will be written with the ancestors information.
If F (Factors) was chosen in the menu, am output file whose default name
is factors will be written containing the factors information.

ERRORS

The output should be checked for error messages. Errors will occur
in the character-state tree descriptions if the format is incorrect
(colons in the wrong place, etc.), if more than one root is specified,
if the tree contains loops (and hence is not a tree), and if the tree is
not connected, e.g.

```
                             A:B B:C D:E
```

describes

```
                  A ---- B ---- C          D ---- E
```

This "tree" is in two unconnected pieces. An error will also occur if a symbol
appears in the data set that is not in the tree description for that
character. Blanks at the end of lines when the species information
is continued to a new line will cause this kind of error.

## CONSTANTS AVAILABLE TO BE CHANGED

At the beginning of the program a number of
are available to be changed to accomodate larger data sets. These are
"maxstates", "maxoutput", "sizearray", "factchar" and "unkchar". The
constant "maxstates"
gives the maximum number of states per character (set at 20 in the
distribution copy). The constant "maxoutput"
gives the maximum width of a line in the output file (80 in the
distribution copy). The constant "sizearray"
must be less than the sum of squares
of the numbers of states in the characters. It is initially set to
set to 2000, so that although 20 states are allowed (at the initial
setting of maxstates) per character, there cannot be 20 states in all
of 100 characters.

Particularly important constants are "factchar" and "unkchar"
which are not numerical
values but a character. Initially set to the colon ":",
"factchar" is the character that will be used to separate states in the input of character
state trees. It can be changed by changing this
constant. (We could have used a hyphen ("-") but didn't because that would make the
minus-sign ("-") unavailable as a character state in +/- characters).
The constant "unkchar"
is the character value in the input data that
indicates that the state is unknown. It is set to "?" in the
distribution copy. If your computer is one that lacks the colon ":" in its
character set or uses a nonstandard character code such as EBCDIC, you
will want to change the constant "factchar".

## INPUT AND OUTPUT FILES

The input file for the program has the default file name "infile"
and the output file, the one that has the binary character state data,
has the name "outfile".

|  |  |
| --- | --- |
| ----SAMPLE INPUT----- | -----Comments (not part of input file) ----- |
| ```    4   6 1 A:B B:C         2 A:B B:.         4                 5 0:1 1:2 .:0     6 .:# #:$ #:%     999               Alpha     CAW00#  Beta      BBX01% Gamma     ABY12# Epsilon   CAZ01$ ``` | ```      4 species; 6 characters      A ---- B ---- C      B ---> A      Character 3 deleted; 4 unchanged      0 ---> 1 ---> 2      % <--- # ---> $      Signals end of trees      Species information begins ``` |
| ---SAMPLE OUTPUT----- | -----Comments (not part of output file) ----- |
| ```     4    8 Alpha     11100000 Beta      10001001 Gamma     00011100 Epsilon   11101010 ``` | ```      4 species; 8 factors      Chars. 1 and 2 come from old number 1      Char. 3 comes from old number 2      Char. 4 is old number 4      Chars. 5 and 6 come from old number 5      Chars. 7 and 8 come from old number 6 ``` |
