# Supplementary material for: Probabilistic Phylogenetic Inference with Insertions and Deletions
Source: PLoS Comput Biol. 2008 Sep 19;4(9):e1000172. doi: 10.1371/journal.pcbi.1000172 (PMC2527138; doi:10.1371/journal.pcbi.1000172)
Supplement: Dataset S1 — Supplemental Material (24.89 MB GZ) [file pcbi.1000172.s001.gz › erate-supplement-R2/src/phylip3.66-erate/doc/dnacomp.html]

dnacomp


version 3.66

# Dnacomp -- DNA Compatibility Program

© Copyright 1986-2006 by the University of
Washington. Written by Joseph Felsenstein. Permission is granted to copy
this document provided that no fee is charged for it and that this copyright
notice is not removed.

This program implements the compatibility method for DNA sequence
data. For a four-state character without a character-state tree, as in
DNA sequences, the usual clique theorems cannot be applied. The
approach taken in this program is to directly evaluate each tree
topology by counting how many substitutions are needed in each site,
comparing this to the minimum number that might be needed (one less than
the number of bases observed at that site), and then evaluating the
number of sites which achieve the minimum number. This is the
evaluation of the tree (the number of compatible sites), and the
topology is chosen so as to maximize that number.

Compatibility methods originated with Le Quesne's (1969) suggestion that
one ought to look for trees supported by the largest number of perfectly
fitting (compatible) characters. Fitch (1975) showed by counterexample that
one could not use the pairwise compatibility methods used in Clique to
discover the largest clique of jointly compatible characters.

The assumptions of this method are similar to those of Clique. In
a paper in the Biological Journal of the Linnean Society (1981b)
I discuss this matter extensively. In effect, the assumptions are that:

1. Each character evolves independently.- Different lineages evolve independently.- The ancestral base at each site is unknown.- The rates of change in most sites over the time spans involved
         in the the divergence of the group are very small.- A few of the sites have very high rates of change.- We do not know in advance which are the high and which the low
             rate sites.

That these are the assumptions of compatibility methods has been documented
in a series of papers of mine: (1973a, 1978b, 1979, 1981b,
1983b, 1988b). For an opposing
view arguing that arguments such as mine are invalid
and that parsimony (and perhaps compatibility) methods make no substantive
assumptions such as these, see the papers by Farris (1983) and Sober (1983a,
1983b, 1988), but also read the exchange between Felsenstein and Sober (1986).

There is, however, some reason to believe that the present criterion is not the
proper way to correct for the presence of some sites with high rates of
change in nucleotide sequence data. It can be argued that sites showing more
than two nucleotide states, even if those are compatible with the other sites,
are also candidates for sites with high rates of change. It might then be more
proper to use Dnapars with the Threshold option with a threshold value of 2.

Change from an occupied site to a gap is counted as one
change. Reversion from a gap to an occupied site is allowed and is also
counted as one change. Note that this in effect assumes that a gap
N bases long is N separate events. This may be an overcorrection. When
we have nonoverlapping gaps, we could instead code a gap as a
single event by changing all but the first "-" in the gap into "?" characters.
In this way only the first base of the gap causes the program to infer a
change.

The input data is standard. The first line of the input file contains the
number of species and the number of sites.

Next come the species data. Each
sequence starts on a new line, has a ten-character species name
that must be blank-filled to be of that length, followed immediately
by the species data in the one-letter code. The sequences must either
be in the "interleaved" or "sequential" formats
described in the Molecular Sequence Programs document. The I option
selects between them. The sequences can have internal
blanks in the sequence but there must be no extra blanks at the end of the
terminated line. Note that a blank is not a valid symbol for a deletion.

The options are selected using an interactive menu. The menu looks like this:

|  |
| --- |
| ``` DNA compatibility algorithm, version 3.6  Settings for this run:   U                 Search for best tree?  Yes   J   Randomize input order of sequences?  No. Use input order   O                        Outgroup root?  No, use as outgroup species  1   W                       Sites weighted?  No   M           Analyze multiple data sets?  No   I          Input sequences interleaved?  Yes   0   Terminal type (IBM PC, ANSI, none)?  ANSI   1    Print out the data at start of run  No   2  Print indications of progress of run  Yes   3                        Print out tree  Yes   4  Print steps & compatibility at sites  No   5  Print sequences at all nodes of tree  No   6       Write out trees onto tree file?  Yes  Are these settings correct? (type Y or the letter for one to change) ``` |

The user either types "Y" (followed, of course, by a carriage-return)
if the settings shown are to be accepted, or the letter or digit corresponding
to an option that is to be changed.

The options U, J, O, W, M, and 0 are the usual ones. They are described in the
main documentation file of this package. Option I is the same as in
other molecular sequence programs and is described in the documentation file
for the sequence programs.

The O (outgroup) option has no effect if the U (user-defined tree) option
is in effect. The user-defined trees (option U) fed in must be strictly
bifurcating, with a two-way split at their base.

The interpretation of weights (option W) in the case of a compatibility method
is that they count how many times the character (in this case the site) is
counted in the analysis. Thus a character can be dropped from the
analysis by assigning it zero weight. On the other hand, giving it a
weight of 5 means that in any clique it is in, it is counted as 5
characters when the size of the clique is evaluated. Generally, weights
other than 0 or 1 do not have much meaning when dealing with DNA sequences.

Output is standard: if option 1 is toggled on, the data is printed out,
with the convention that "." means "the same as in the first species".
Then comes a list of equally parsimonious trees, and (if option 2 is
toggled on) a table of the
number of changes of state required in each character. If option 5 is toggled
on, a table is printed
out after each tree, showing for each branch whether there are known to be
changes in the branch, and what the states are inferred to have been at the
top end of the branch. If the inferred state is a "?" or one of the IUB
ambiguity symbols, there will be multiple
equally-parsimonious assignments of states; the user must work these out for
themselves by hand. A "?" in the reconstructed states means that in
addition to one or more bases, a gap may or may not be present. If
option 6 is left in its default state the trees
found will be written to a tree file, so that they are available to be used
in other programs. If the program finds multiple
trees tied for best, all of these are written out onto the output tree
file. Each is followed by a numerical weight in square brackets (such as
[0.25000]). This is needed when we use the trees to make a consensus
tree of the results of bootstrapping or jackknifing, to avoid overrepresenting
replicates that find many tied trees.

If the U (User Tree) option is used and more than one tree is supplied,
the program also performs a statistical test of each of these trees against the
one with highest likelihood. If there are two user trees, the test
done is one which is due to Kishino and Hasegawa (1989), a version
of a test originally introduced by Templeton (1983). In this
implementation it uses the mean and variance of weighted
compatibility differences between trees, taken across sites. If the two
trees compatibilities are more than 1.96 standard deviations different then
the trees are declared significantly different.

If there are more than two trees, the test done is an extension of
the KHT test, due to Shimodaira and Hasegawa (1999). They pointed out
that a correction for the number of trees was necessary, and they
introduced a resampling method to make this correction. In the version
used here the variances and covariances of the sum of weighted
compatibilities of sites are computed for all pairs of trees. To
test whether the
difference between each tree and the best one is larger than could have
been expected if they all had the same expected compatibility,
compatibilities for all trees are sampled with these covariances and equal
means (Shimodaira and Hasegawa's "least favorable hypothesis"),
and a P value is computed from the fraction of times the difference between
the tree's value and the highest compatibility exceeds that actually
observed. Note that this sampling needs random numbers, and so the
program will prompt the user for a random number seed if one has not
already been supplied. With the two-tree KHT test no random numbers
are used.

In either the KHT or the SH test the program
prints out a table of the compatibility of each tree, the differences of
each from the highest one, the variance of that quantity as determined by
the compatibility differences at individual sites, and a conclusion as to
whether that tree is or is not significantly worse than the best one.

The algorithm is a straightforward modification of Dnapars, but with
some extra machinery added to calculate, as each species is added, how
many base changes are the minimum which could be required at that site. The
program runs fairly quickly.

The constants
which can be changed at the beginning of the program are:
the name length "nmlngth",
"maxtrees", the maximum number of trees which the program will store for output,
and "maxuser",
the maximum number of user trees that can be used in the paired sites test.

---

### TEST DATA SET

|  |
| --- |
| ```     5   13 Alpha     AACGUGGCCAAAU Beta      AAGGUCGCCAAAC Gamma     CAUUUCGUCACAA Delta     GGUAUUUCGGCCU Epsilon   GGGAUCUCGGCCC ``` |

### CONTENTS OF OUTPUT FILE (if all numerical options are turned on)

|  |
| --- |
| ``` DNA compatibility algorithm, version 3.66   5 species,  13  sites  Name            Sequences ----            ---------  Alpha        AACGUGGCCA AAU Beta         ..G..C.... ..C Gamma        C.UU.C.U.. C.A Delta        GGUA.UU.GG CC. Epsilon      GGGA.CU.GG CCC   One most parsimonious tree found:              +--Epsilon            +--4        +--3  +--Delta           !  !     +--2  +-----Gamma        !  !     1  +--------Beta         !     +-----------Alpha         remember: this is an unrooted tree!   total number of compatible sites is       11.0  steps in each site:          0   1   2   3   4   5   6   7   8   9      *-----------------------------------------     0|       2   1   3   2   0   2   1   1   1    10|   1   1   1   3                           compatibility (Y or N) of each site with this tree:        0123456789      *----------    0 ! YYNYYYYYY   10 !YYYN        From    To     Any Steps?    State at upper node                              ( . means same as in the node below it on tree)            1                AABGTSGCCA AAY    1      2        maybe   .....C.... ...    2      3         yes    V.KD...... C..    3      4         yes    GG.A..T.GG .C.    4   Epsilon     maybe   ..G....... ..C    4   Delta        yes    ..T..T.... ..T    3   Gamma        yes    C.TT...T.. ..A    2   Beta        maybe   ..G....... ..C    1   Alpha       maybe   ..C..G.... ..T ``` |
