# Supplementary material for: Probabilistic Phylogenetic Inference with Insertions and Deletions
Source: PLoS Comput Biol. 2008 Sep 19;4(9):e1000172. doi: 10.1371/journal.pcbi.1000172 (PMC2527138; doi:10.1371/journal.pcbi.1000172)
Supplement: Dataset S1 — Supplemental Material (24.89 MB GZ) [file pcbi.1000172.s001.gz › erate-supplement-R2/src/phylip3.66-erate/doc/treedist.html]

treedist


version 3.66

# Treedist -- distances between trees

© Copyright 2000-2006 by The University of
Washington. Written by Joseph Felsenstein. Permission is granted to copy
this document provided that no fee is charged for it and that this copyright
notice is not removed.

This program computes distances between trees. Two distances are computed,
the Branch Score Distance of Kuhner and Felsenstein (1994), and
the more widely known Symmetric Difference of Robinson and Foulds (1981).
The Branch Score Distance uses branch lengths, and can only be calculated
when the trees have lengths on all branches. The Symmetric Difference
does not use branch length information, only the tree topologies. It
must also be borne in mind that neither distance has any immediate
statistical interpretation -- we cannot say whether a larger distance is
significantly larger than a smaller one.

These distances are computed by considering all possible branches that could
exist on the
the two trees. Each branch divides the set of species into two groups --
the ones connected to one end of the branch and the ones connected to the
other. This makes a partition of the full set of species. (in Newick notation)

```
  ((A,C),(D,(B,E)))
```

has two internal branches. One induces the partition {A, C  |  B, D, E}
and the other induces the partition {A, C, D  |  B, E}. A different tree
with the same set of species,

```
  (((A,D),C),(B,E))
```

has internal branches that correspond to the two partitions {A, C, D  |  B, E}
and {A, D  |  B, C, E}. Note that the other branches, all of which are
external branches, induce partitions that separate one species from all the
others. Thus there are 5 partitions like this: {C  |  A, B, D, E} on each
of these trees. These are always present on all trees, provided that each
tree has each species at the end of its own branch.

In the case of the Branch Score distance, each partition that does exist on
a tree also has a branch length associated with it. Thus if the tree is

```
  (((A:0.1,D:0.25):0.05,C:0.01):0.2,(B:0.3,E:0.8):0.2)
```

The list of partitions and their branch lengths is:

|  |  |
| --- | --- |
| {A  |  B, C, D, E} | 0.1 |
| {D  |  A, B, C, E} | 0.25 |
| {A, D  |  B, C, E} | 0.05 |
| {C  |  A, B, D, E} | 0.01 |
| {A, D, C  |  B, E} | 0.4 |
| {B  |  A, C, D, E} | 0.3 |
| {E  |  A, B, C, D} | 0.8 |

Note that the tree is being treated as unrooted here, so that the branch
lengths on either side of the rootmost node are summed up to get a branch
length of 0.4.

The Branch Score Distance imagines us as having made a list of all
possible partitions, the ones shown above and also all 7 other possible
partitions, which correspond to branches that are not found in this tree.
These are assigned branch lengths of 0.
For two trees, we imagine constructing these lists, and then summing the
squared differences between the branch lengths. Thus if both trees have
branches {A, D  |  B, C, E}, the sum
contains the square of the difference between the branch lengths. If one
tree has the branch and the other doesn't, it contains the square of the
difference between the branch length and zero (in other words, the
square of that branch length). If both trees do not have a particular
branch, nothing is added to the sum because the difference is then between
0 and 0.

The Branch Score Distance takes this sum of squared differences and
computes its square root. Note that it has some desirable properties.
When small branches differ in tree topology, it is not very big.
When branches are both present but differ in length, it is affected.

The Symmetric Difference is simply a count of how many partitions there are,
among the two trees, that are on one tree and not on the other. In the
example above there are two partitions, {A, C  |  B, D, E} and {A, D  |  B, C, E},
each of which is present on only one of the two trees. The Symmetric
Difference between the two trees is therefore 2. When the two trees are
fully resolved bifurcating trees, their symmetric distance must be an even
number; it can range from 0 to twice the number of internal branches, which
for *n* species is 4n-10 (for 3 species or more).

Note the relationship between the two distances. If all trees have all their
branches have length 1.0, the Branch Score Distance is the square root of the
Symmetric Difference, as each branch that is present in one but not in the
other results in 1.0 being added to the sum of squared differences.

We have assumed that nothing is lost if the trees are treated as unrooted trees.
It is easy to define a counterpart to the Branch Score Distance and one to
the Symmetric Difference for these rooted trees.
Each branch then defines a set of species, namely the clade defined by that
branch. Thus if the first of the two trees above were considered as a rooted
tree it would define the three clades {A, C}, {B, D, E}, and {B, E}. The
Branch Score Distance is computed from the branch lengths for all possible
sets of species, with 0 put for each set that does not occur on that tree.
The table above will be nearly the same, but with two entries instead of
one for the sets on either side of the root, {A C D} and {B E}.
The
Symmetric Difference between two rooted trees is simply the count of the number
of clades that are defined by one but not by the other. For the second tree
the clades would be {A, D}, {B, C, E}, and {B, E}. The Symmetric Difference
between these two rooted trees would then be 4.

Although the examples we have discussed have involved fully
bifurcating trees, the input trees can have multifurcations.
This does not cause any complication for the Branch Score Distance.
For the Symmetric Difference, it can lead to distances that are odd numbers.

However, note one strong restriction. The trees should all have the same
list of species. If you use one set of species in the first two trees,
and another in the second two, and choose distances for adjacent pairs,
the distances will be incorrect and will depend on the order of these
pairs in the input tree file, in odd ways.

## INPUT AND OPTIONS

The program reads one or two input tree files. If there is one input tree
file, its default name is intree. If there are two their default
names are intree and intree2. The tree files may either
have the number of trees on their first line, or not. If the number of
trees is given, it is actually ignored and all trees in the tree file
are considered, even if there are more trees than indicated by the number.
There is no maximum number of trees that can be processed but, if you
feed in too many, there may be an error message about running out of
memory. The problem is particularly acute if you choose the option to
examine all possible pairs of trees in an input tree file, or all possible
pairs of trees one from each of two input tree files. Thus if there are
1,000 trees in the input tree file, keep in mind that all possible
pairs means 1,000,000 pairs to be examined!

On the other hand, there is a maximum of the number of species that can
be in any tree. At the moment it is about 10,000. If you need to handle
more species than that, you can recompile the program after changing the
value of the variable maxgrp to make it higher. It is about
twice the maximum number of species.

The options are selected from a menu, which looks like this:

|  |
| --- |
| ``` Tree distance program, version 3.66  Settings for this run:  D                         Distance Type:  Branch Score Distance  O                         Outgroup root:  No, use as outgroup species  1  R         Trees to be treated as Rooted:  No  T    Terminal type (IBM PC, ANSI, none):  ANSI  1  Print indications of progress of run:  Yes  2                 Tree distance submenu:  Distance between adjacent pairs  Are these settings correct? (type Y or the letter for one to change) ``` |

The D option chooses which distance measure to use. The Branch Score
Distance is the default. If it is in force, and any of the trees which are
read in have even one branch that fails to have a length, the program will
terminate with an error. If the Symmetric Difference option is chosen,
no check of branch lengths is made.

The O option allows you to root the trees using an outgroup. It is specified
by giving its number, where the species are numbered in the order they
appear in the first tree. Outgroup-rooting all the trees does not
affect the distances if the trees are treated as unrooted, and if it is done
and trees are
treated as rooted, the distances turn out to be the same as the unrooted
ones. Thus it is unlikely that you will find this option of interest.

The R option controls whether the Symmetric Distance that is computed is
to treat the trees as unrooted or rooted. Unrooted is the default.

The terminal type (0) and progress (1) options do not need description here.

Option 2 controls how many tree files are read in, which trees are to
be compared, and how the output is to be presented. It causes
another menu to appear:

|  |
| --- |
| ``` Tree Pairing Submenu:  A     Distances between adjacent pairs in tree file.  P     Distances between all possible pairs in tree file.  C     Distances between corresponding pairs in one tree file and another.  L     Distances between all pairs in one tree file and another.   Choose one: (A,P,C,L) ``` |

Option A computes the distances between successive pairs of trees in the
tree input file -- between trees 1 and 2, trees 3 and 4, trees
5 and 6, and so on. If there are an odd number of trees in the input tree
file the last tree will be ignored and a warning message printed to
remind the user that nothing was done with it.

Option P computes distances between all pairs of trees in the input tree
file. Thus with 10 trees 10 x 10 = 100 distances will be computed,
including distances between each tree and itself.

Option C takes input from two tree files and cmputes distances between
corresponding members of the two tree files. Thus distances will be
computed between tree 1 of the first tree file and tree 1 of the second one,
between tree 2 of the first file and tree 2 of the second one, and so on.
If the number of trees in the two files differs, the extra trees in the
file that has more of them are ignored and a warning is printed out.

Option L computes distances between all pairs of trees, where one tree is
taken from one tree file and the other from the other tree file. Thus if
the first tree file has 7 trees and the second has 5 trees, 7 x 5 = 35
different distances will be computed.

If option 2 is not selected, the program defaults to looking at one tree
file and computing distances of adjacent pairs (so that option A is
the default).

## OUTPUT

The results of the analysis are written onto an output file whose
default file name is outfile.

If any of the four types of analysis are selected, the program asks the
user how they want the results presented. Here is that menu for options
P or L:

|  |
| --- |
| ``` Distances output options:  F     Full matrix.  V     One pair per line, verbose.  S     One pair per line, sparse.   Choose one: (F,V,S) ``` |

The Full matrix (choice F) is a table showing all distances. It is
written onto the output file. The table is presented as groups of
10 columns. Here is the Full matrix for the 12 trees in the input
tree file which is given as an example at the end of this page.

|  |
| --- |
| ``` Tree distance program, version 3.66  Symmetric differences between all pairs of trees in tree file:             1     2     3     4     5     6     7     8     9    10        \------------------------------------------------------------     1 |   0     4     2    10    10    10    10    10    10    10       2 |   4     0     2    10     8    10     8    10     8    10       3 |   2     2     0    10    10    10    10    10    10    10       4 |  10    10    10     0     2     2     4     2     4     0       5 |  10     8    10     2     0     4     2     4     2     2       6 |  10    10    10     2     4     0     2     2     4     2       7 |  10     8    10     4     2     2     0     4     2     4       8 |  10    10    10     2     4     2     4     0     2     2       9 |  10     8    10     4     2     4     2     2     0     4      10 |  10    10    10     0     2     2     4     2     4     0      11 |   2     2     0    10    10    10    10    10    10    10      12 |  10    10    10     2     4     2     4     0     2     2             11    12        \------------     1 |   2    10       2 |   2    10       3 |   0    10       4 |  10     2       5 |  10     4       6 |  10     2       7 |  10     4       8 |  10     0       9 |  10     2      10 |  10     2      11 |   0    10      12 |  10     0 ``` |

The Full matrix is only available for analyses P and L (not for A or C).

Option V (Verbose) writes one distance per line. The Verbose
output is the default. Here it is for the example data set given below:

|  |
| --- |
| ``` Tree distance program, version 3.66  Symmetric differences between adjacent pairs of trees:  Trees 1 and 2:    4 Trees 3 and 4:    10 Trees 5 and 6:    4 Trees 7 and 8:    4 Trees 9 and 10:    4 Trees 11 and 12:    10 ``` |

Option S (Sparse or terse) is similar except that all that is
given on each line are the numbers of the two trees and the distance,
separated by blanks. This may be a convenient format if you want to
write a program to read these numbers in, and you want to spare yourself
the effort of having the program wade through the words on each line
in the Verbose output.
The first four lines of the Sparse output are titles that your program would
want to skip past. Here is the Sparse output for the example trees.

|  |
| --- |
| ``` 1 2 4 3 4 10 5 6 4 7 8 4 9 10 4 11 12 10 ``` |

## CREDITS AND FUTURE

Treedist was originally written by Dan Fineman, with fixes by Doug Buxton.
We also hope in the future to compute a distance based on
quartets shared and not shared by trees (implicit in the work of Estabrook, McMorris, and
Meacham, 1985). We will also implement the tree distance of Robinson and
Foulds (1979), which is like the Branch Score Distance but uses absolute
values of differences between branch lengths rather than sums of squares of
differences.

---

### TEST DATA SET 1

|  |
| --- |
| ``` (A,(B,(H,(D,(J,(((G,E),(F,I)),C)))))); (A,(B,(D,((J,H),(((G,E),(F,I)),C))))); (A,(B,(D,(H,(J,(((G,E),(F,I)),C)))))); (A,(B,(E,(G,((F,I),((J,(H,D)),C)))))); (A,(B,(E,(G,((F,I),(((J,H),D),C)))))); (A,(B,(E,((F,I),(G,((J,(H,D)),C)))))); (A,(B,(E,((F,I),(G,(((J,H),D),C)))))); (A,(B,(E,((G,(F,I)),((J,(H,D)),C))))); (A,(B,(E,((G,(F,I)),(((J,H),D),C))))); (A,(B,(E,(G,((F,I),((J,(H,D)),C)))))); (A,(B,(D,(H,(J,(((G,E),(F,I)),C)))))); (A,(B,(E,((G,(F,I)),((J,(H,D)),C))))); ``` |

The output from the setting in the D menu choice of the Symmetric Difference
for this test set is given above (it is the
Verbose output example).

---

### TEST DATA SET 2

This data set is the first part of the previous one, but with branch
lengths on the trees, to serve as an example for the Branch Score distance.

|  |
| --- |
| ``` (A:0.1,(B:0.1,(H:0.1,(D:0.1,(J:0.1,(((G:0.1,E:0.1):0.1,(F:0.1,I:0.1):0.1):0.1, C:0.1):0.1):0.1):0.1):0.1):0.1); (A:0.1,(B:0.1,(D:0.1,((J:0.1,H:0.1):0.1,(((G:0.1,E:0.1):0.1, (F:0.1,I:0.1):0.1):0.1,C:0.1):0.1):0.1):0.1):0.1); (A:0.1,(B:0.1,(D:0.1,(H:0.1,(J:0.1,(((G:0.1,E:0.1):0.1,(F:0.1,I:0.1):0.1):0.1, C:0.1):0.1):0.1):0.1):0.1):0.1); (A:0.1,(B:0.1,(E:0.1,(G:0.1,((F:0.1,I:0.1):0.1,((J:0.1,(H:0.1,D:0.1):0.1):0.1, C:0.1):0.1):0.1):0.1):0.1):0.1); (A:0.1,(B:0.1,(E:0.1,(G:0.1,((F:0.1,I:0.1):0.1,(((J:0.1,H:0.1):0.1,D:0.1):0.1, C:0.1):0.1):0.1):0.1):0.1):0.1); (A:0.1,(B:0.1,(E:0.1,((F:0.1,I:0.1):0.1,(G:0.1,((J:0.1,(H:0.1,D:0.1):0.1):0.1, C:0.1):0.1):0.1):0.1):0.1):0.1); (A:0.1,(B:0.1,(E:0.1,((F:0.1,I:0.1):0.1,(G:0.1,(((J:0.1,H:0.1):0.1,D:0.1):0.1, C:0.1):0.1):0.1):0.1):0.1):0.1); (A:0.1,(B:0.1,(E:0.1,((G:0.1,(F:0.1,I:0.1):0.1):0.1,((J:0.1,(H:0.1, D:0.1):0.1):0.1,C:0.1):0.1):0.1):0.1):0.1); (A:0.1,(B:0.1,(E:0.1,((G:0.1,(F:0.1,I:0.1):0.1):0.1,(((J:0.1,H:0.1):0.1, D:0.1):0.1,C:0.1):0.1):0.1):0.1):0.1); (A:0.1,(B:0.1,(E:0.1,(G:0.1,((F:0.1,I:0.1):0.1,((J:0.1,(H:0.1,D:0.1):0.1):0.1, C:0.1):0.1):0.1):0.1):0.1):0.1); (A:0.1,(B:0.1,(D:0.1,(H:0.1,(J:0.1,(((G:0.1,E:0.1):0.1,(F:0.1,I:0.1):0.1):0.1, C:0.1):0.1):0.1):0.1):0.1):0.1); (A:0.1,(B:0.1,(E:0.1,((G:0.1,(F:0.1,I:0.1):0.1):0.1,((J:0.1,(H:0.1, D:0.1):0.1):0.1,C:0.1):0.1):0.1):0.1):0.1); ``` |

---

### TEST SET 2 OUTPUT

This was run using the default Branch Score distance, and asking in
option 2 for the P (all pairs in file) setting and the F (Full matrix output)
setting.

|  |
| --- |
| ``` Tree distance program, version 3.66  Branch score distances between all pairs of trees in tree file:                   1           2           3           4           5           6           7        \------------------------------------------------------------------------------------     1 |         0         0.2    0.141421    0.316228    0.316228    0.316228    0.316228       2 |       0.2           0    0.141421    0.316228    0.282843    0.316228    0.282843       3 |  0.141421    0.141421           0    0.316228    0.316228    0.316228    0.316228       4 |  0.316228    0.316228    0.316228           0    0.141421    0.141421         0.2       5 |  0.316228    0.282843    0.316228    0.141421           0         0.2    0.141421       6 |  0.316228    0.316228    0.316228    0.141421         0.2           0    0.141421       7 |  0.316228    0.282843    0.316228         0.2    0.141421    0.141421           0       8 |  0.316228    0.316228    0.316228    0.141421         0.2    0.141421         0.2       9 |  0.316228    0.282843    0.316228         0.2    0.141421         0.2    0.141421      10 |  0.316228    0.316228    0.316228           0    0.141421    0.141421         0.2      11 |  0.141421    0.141421           0    0.316228    0.316228    0.316228    0.316228      12 |  0.316228    0.316228    0.316228    0.141421         0.2    0.141421         0.2                    8           9          10          11          12        \------------------------------------------------------------     1 |  0.316228    0.316228    0.316228    0.141421    0.316228       2 |  0.316228    0.282843    0.316228    0.141421    0.316228       3 |  0.316228    0.316228    0.316228           0    0.316228       4 |  0.141421         0.2           0    0.316228    0.141421       5 |       0.2    0.141421    0.141421    0.316228         0.2       6 |  0.141421         0.2    0.141421    0.316228    0.141421       7 |       0.2    0.141421         0.2    0.316228         0.2       8 |         0    0.141421    0.141421    0.316228           0       9 |  0.141421           0         0.2    0.316228    0.141421      10 |  0.141421         0.2           0    0.316228    0.141421      11 |  0.316228    0.316228    0.316228           0    0.316228      12 |         0    0.141421    0.141421    0.316228           0 ``` |
