# Supplementary material for: Probabilistic Phylogenetic Inference with Insertions and Deletions
Source: PLoS Comput Biol. 2008 Sep 19;4(9):e1000172. doi: 10.1371/journal.pcbi.1000172 (PMC2527138; doi:10.1371/journal.pcbi.1000172)
Supplement: Dataset S1 — Supplemental Material (24.89 MB GZ) [file pcbi.1000172.s001.gz › erate-supplement-R2/src/phylip3.66-erate/doc/dnadist.html]

dnadist


version 3.66

# Dnadist -- Program to compute distance matrix from nucleotide sequences

© Copyright 1986-2006 by the University of
Washington. Written by Joseph Felsenstein. Permission is granted to copy
this document provided that no fee is charged for it and that this copyright
notice is not removed.

This program uses nucleotide sequences to compute a distance matrix, under
four different models of nucleotide substitution. It can also
compute a table of similarity between the nucleotide sequences.
The distance for each
pair of species estimates the total branch length between the two species, and
can be used in the distance matrix programs Fitch, Kitsch or Neighbor. This
is an alternative to use of the sequence data itself in the maximum likelihood
program Dnaml or the parsimony program Dnapars.

The program reads in nucleotide sequences and writes an output file containing
the distance matrix, or else a table of similarity between sequences.
The four models of nucleotide substitution are those
of Jukes and Cantor (1969), Kimura (1980), the F84 model (Kishino and Hasegawa,
1989; Felsenstein and Churchill, 1996), and the model underlying the
LogDet distance (Barry and Hartigan, 1987; Lake, 1994;
Steel, 1994; Lockhart et. al., 1994).
All except the LogDet distance can be made to allow for
for unequal rates of substitution at different sites, as Jin and Nei
(1990) did for the Jukes-Cantor model.
The program correctly takes
into account a variety of sequence ambiguities, although in cases where they
exist it can be slow.

Jukes and Cantor's (1969) model assumes that there is independent change at
all sites, with equal probability. Whether a base changes is independent of
its identity, and when it changes there is an equal probability of ending up
with each of the other three bases. Thus the transition probability matrix
(this is a technical term from probability theory and has nothing to do with
transitions as opposed to transversions) for a short period of time dt is:

```
              To:    A        G        C        T
                   ---------------------------------
               A  | 1-3a      a         a       a
       From:   G  |  a       1-3a       a       a
               C  |  a        a        1-3a     a
               T  |  a        a         a      1-3a
```

where *a* is *u dt*, the product of the rate of substitution per unit time (*u*)
and the length *dt* of the time interval. For longer periods of time this
implies that the probability that two sequences will differ at a given site
is:

      p = 3/4 ( 1 - e- 4/3 u t)

and hence that if we observe *p*, we can compute an estimate of the branch
length *ut* by inverting this to get

     ut = - 3/4 loge ( 1 - 4/3 p )

The Kimura "2-parameter" model is almost as symmetric as this, but allows
for a difference between transition and transversion rates. Its transition
probability matrix for a short interval of time is:

```
              To:     A        G        C        T
                   ---------------------------------
               A  | 1-a-2b     a         b       b
       From:   G  |   a      1-a-2b      b       b
               C  |   b        b       1-a-2b    a
               T  |   b        b         a     1-a-2b
```

where *a* is *u dt*, the product of the rate of transitions per unit time and *dt*
is the length *dt* of the time interval, and *b* is *v dt*, the product of half the
rate of transversions (i.e., the rate of a specific transversion)
and the length dt of the time interval.

The F84 model incorporates different rates of transition
and transversion, but also allowing for different frequencies of the four
nucleotides. It is the model which is used in Dnaml, the maximum likelihood
nucelotide sequence phylogenies program in this package. You will find the
model described in the document for that program. The transition
probabilities for this model are given by Kishino and Hasegawa (1989),
and further explained in a paper by me and Gary Churchill (1996).

The LogDet distance allows a fairly general model of substitution. It
computes the distance from the determinant of the empirically observed
matrix of joint probabilities of nucleotides in the two species. An
explanation of it is available in the chapter by Swofford et, al. (1996).

The first three models are closely related. The Dnaml model reduces to
Kimura's
two-parameter model if we assume that the equilibrium frequencies of the four
bases are equal. The Jukes-Cantor model in turn is a special case of the
Kimura 2-parameter model where a = b. Thus each model is a special case of
the ones that follow it, Jukes-Cantor being a special case of both of the
others.

The Jin and Nei (1990) correction for variation in rate of evolution from
site to site can be adapted to all of the first three models.
It assumes that the rate of substitution varies from site to site
according to a gamma distribution, with a coefficient of variation that
is specified by the user. The user is asked for it when choosing this option
in the menu.

Each distance that is calculated is an estimate, from that particular pair of
species, of the divergence time between those two species. For the Jukes-
Cantor model, the estimate is computed using the formula for *ut* given above,
as long as the nucleotide symbols in the two sequences are all either A, C, G,
T, U, N, X, ?, or - (the latter four indicate a deletion or an unknown
nucleotide. This estimate is a maximum likelihood estimate for that
model. For the Kimura 2-parameter model, with only these nucleotide symbols,
formulas special to that estimate are also computed. These are also,
in effect, computing the maximum likelihood estimate for that model. In
the Kimura case it depends on the
observed sequences only through the sequence length and the observed number of
transition and transversion differences between those two sequences. The
calculation in that case is a maximum likelihood estimate and will differ
somewhat from the estimate obtained from the formulas in Kimura's original
paper. That formula was also a maximum likelihood estimate, but with the
transition/transversion ratio estimated empirically, separately for each pair
of sequences. In the present case, one overall preset transition/transversion
ratio is used which makes the computations harder but achieves greater
consistency between different comparisons.

For the
F84 model, or for any of the models where one or both sequences contain at
least one of the other ambiguity codons such as Y, R, etc., a maximum
likelihood calculation is also done using code which was originally written
for Dnaml. Its disadvantage is that it is slow. The resulting
distance is in effect a maximum likelihood estimate of the divergence time
(total branch length between) the two sequences. However the present
program will be much faster than versions earlier than 3.5, because I have
speeded up the iterations.

The LogDet model computes the distance from the determinant of the
matrix of co-occurrence of nucleotides in the two species, according to
the formula

```
   D  = - 1/4(loge(|F|) - 1/2loge(fA1fC1fG1fT1fA2fC2fG2fT2))
```

Where *F* is a matrix whose *(i,j)* element is the fraction
of sites at which base *i* occurs in one species and base *j*
occurs in the other. fji is the fraction of sites at
which species *i* has base *j*.
The LogDet distance cannot cope with ambiguity codes. It must have
completely defined sequences. One limitation of the LogDet distance is
that it may be infinite sometimes, if there are too many changes between
certain pairs of nucleotides. This can be particularly noticeable with
distances computed from bootstrapped sequences.

Note that there is an
assumption that we are looking at all sites, including those
that have not changed at all. It is important not to restrict attention
to some sites based on whether or not they have changed; doing that
would bias the distances by making them too large, and that in turn
would cause the distances
to misinterpret the meaning of those sites that
had changed.

For all of these
distance methods, the program allows us to specify
that "third position" bases have a different rate of substitution than first and
second positions, that introns have a different rate than exons, and so on. The
Categories option which does this allows us to make up to
9 categories of sites and specify different rates of change for them.

In addition to the four distance calculations, the program can also
compute a table of similarities between nucleotide sequences. These values
are the fractions of sites identical between the sequences.
The diagonal values are 1.0000. No attempt is made to count similarity
of nonidentical nucleotides, so that no credit is given for having
(for example) different purines at corresponding
sites in the two sequences. This option has been requested by many
users, who need it for descriptive purposes. It is not intended that
the table be used for inferring the tree.

## INPUT FORMAT AND OPTIONS

Input is fairly standard, with one addition. As usual the first line of the
file gives the number of species and the number of sites.

Next come the species data. Each
sequence starts on a new line, has a ten-character species name
that must be blank-filled to be of that length, followed immediately
by the species data in the one-letter code. The sequences must either
be in the "interleaved" or "sequential" formats
described in the Molecular Sequence Programs document. The I option
selects between them. The sequences can have internal
blanks in the sequence but there must be no extra blanks at the end of the
terminated line. Note that a blank is not a valid symbol for a deletion --
neither is dot (".").

The options are selected using an interactive menu. The menu looks like this:

|  |
| --- |
| ``` Nucleic acid sequence Distance Matrix program, version 3.6  Settings for this run:   D  Distance (F84, Kimura, Jukes-Cantor, LogDet)?  F84   G          Gamma distributed rates across sites?  No   T                 Transition/transversion ratio?  2.0   C            One category of substitution rates?  Yes   W                         Use weights for sites?  No   F                Use empirical base frequencies?  Yes   L                       Form of distance matrix?  Square   M                    Analyze multiple data sets?  No   I                   Input sequences interleaved?  Yes   0            Terminal type (IBM PC, ANSI, none)?  ANSI   1             Print out the data at start of run  No   2           Print indications of progress of run  Yes    Y to accept these or type the letter for one to change ``` |

The user either types "Y" (followed, of course, by a carriage-return)
if the settings shown are to be accepted, or the letter or digit corresponding
to an option that is to be changed.

The D option selects one of the four distance methods, or the
similarity table. It toggles among the
five methods. The default method, if none is specified, is the F84
model.

If the G (Gamma distribution) option is selected, the user will be
asked to supply the coefficient of variation of the rate of substitution
among sites. This is different from the parameters used by Nei and Jin but
related to them: their parameter *a* is also known as "alpha",
the shape parameter of the Gamma distribution. It is
related to the coefficient of variation by

     CV = 1 / a1/2

or

     a = 1 / (CV)2

(their parameter *b* is absorbed here by the requirement that time is scaled so
that the mean rate of evolution is 1 per unit time, which means that *a = b*).
As we consider cases in which the rates are less variable we should set *a*
larger and larger, as *CV* gets smaller and smaller.

The F (Frequencies) option appears when the Maximum Likelihood distance is
selected. This distance requires that the program be provided with the
equilibrium frequencies of the four bases A, C, G, and T (or U). Its default
setting is one which may save users much time. If you
want to use the empirical frequencies of the bases, observed in the input
sequences, as the base frequencies, you simply use the default setting of
the F option. These empirical
frequencies are not really the maximum likelihood estimates of the base
frequencies, but they will often be close to those values (what they are is
maximum likelihood estimates under a "star" or "explosion" phylogeny).
If you change the setting of the F option you will be prompted for the
frequencies of the four bases. These must add to 1 and are to be typed on
one line separated by blanks, not commas.

The T option in this program does not stand for Threshold,
but instead is the Transition/transversion option. The user is prompted for
a real number greater than 0.0, as the expected ratio of transitions to
transversions. Note
that this is not the ratio of the first to the second kinds of events,
but the resulting expected ratio of transitions to transversions. The exact
relationship between these two quantities depends on the frequencies in the
base pools. The default value of the T parameter if you do not use the T
option is 2.0.

The C option allows user-defined rate categories. The user is prompted
for the number of user-defined rates, and for the rates themselves,
which cannot be negative but can be zero. These numbers, which must be
nonnegative (some could be 0),
are defined relative to each other, so that if rates for three categories
are set to 1 : 3 : 2.5 this would have the same meaning as setting them
to 2 : 6 : 5.
The assignment of rates to
sites is then made by reading a file whose default name is "categories".
It should contain a string of digits 1 through 9. A new line or a blank
can occur after any character in this string. Thus the categories file
might look like this:

```
122231111122411155
1155333333444
```

If both user-assigned rate categories and Gamma-distributed rates
are allowed, the program assumes that the
actual rate at a site is the product of the user-assigned category rate
and the Gamma-distributed rate. This allows you to specify that
certain sites have higher or lower rates of change while also allowing the
program to allow variation of rates in addition to that.
(This may not always make
perfect biological sense: it would be more natural to assume some upper
bound to the rate, as we have discussed in the Felsenstein and Churchill
paper). Nevertheless you may want to use both types of rate variation.

The L option specifies that the output file is to have the distance
matrix in lower triangular form.

The W (Weights) option is invoked in the usual way, with only weights 0
and 1 allowed. It selects a set of sites to be analyzed, ignoring the
others. The sites selected are those with weight 1. If the W option is
not invoked, all sites are analyzed.
The Weights (W) option
takes the weights from a file whose default name is "weights". The weights
follow the format described in the main documentation file.

The M (multiple data sets) option will ask you whether you want to
use multiple sets of weights (from the weights file) or multiple data sets
from the input file.
The ability to use a single data set with multiple weights means that
much less disk space will be used for this input data. The bootstrapping
and jackknifing tool Seqboot has the ability to create a weights file with
multiple weights. Note also that when we use multiple weights for
bootstrapping we can also then maintain different rate categories for
different sites in a meaningful way. You should not use the multiple
data sets option without using multiple weights, you should not at the
same time use the user-defined rate categories option (option C).

The options 0 is the usual one. It is described in the
main documentation file of this package. Option I is the same as in
other molecular sequence programs and is described in the documentation file
for the sequence programs.

## OUTPUT FORMAT

As the
distances are computed, the program prints on your screen or terminal
the names of the species in turn,
followed by one dot (".") for each other species for which the distance to
that species has been computed. Thus if there are ten species, the first
species name is printed out, followed by nine dots, then on the next line
the next species name is printed out followed by eight dots, then the
next followed by seven dots, and so on. The pattern of dots should form
a triangle. When the distance matrix has been written out to the output
file, the user is notified of that.

The output file contains on its first line the number of species. The
distance matrix is then printed in standard
form, with each species starting on a new line with the species name, followed
by the distances to the species in order. These continue onto a new line
after every nine distances. If the L option is used, the matrix or distances
is in lower triangular form, so that only the distances to the other species
that precede each species are printed. Otherwise the distance matrix is square
with zero distances on the diagonal. In general the format of the distance
matrix is such that it can serve as input to any of the distance matrix
programs.

If the option to print out the data is selected, the output file will
precede the data by more complete information on the input and the menu
selections. The output file begins by giving the number of species and the
number of characters, and the identity of the distance measure that is
being used.

If the C (Categories) option is used a table of the relative rates of
expected substitution at each category of sites is printed, and a listing
of the categories each site is in.

There will then follow the equilibrium frequencies of the four bases.
If the Jukes-Cantor or Kimura distances are used, these will necessarily be
0.25 : 0.25 : 0.25 : 0.25. The output then shows
the transition/transversion ratio that was specified or
used by default. In the case of the Jukes-Cantor distance this will always
be 0.5. The transition-transversion parameter (as opposed to the ratio)
is also printed out: this is used within the program and can be ignored.
There then follow the data sequences, with the base sequences printed in
groups of ten bases along the lines of the Genbank and EMBL formats.

The distances printed out are scaled in terms of expected numbers of
substitutions, counting both transitions and transversions but not
replacements of a base by itself, and scaled so that the average rate of
change, averaged over all sites analyzed, is set to 1.0
if there are multiple categories of sites. This means that whether or not
there are multiple categories of sites, the expected fraction of change
for very small branches is equal to the branch length. Of course,
when a branch is twice as
long this does not mean that there will be twice as much net change expected
along it, since some of the changes may occur in the same site and overlie or
even reverse each
other. The branch lengths estimates here are in terms of the expected
underlying numbers of changes. That means that a branch of length 0.26
is 26 times as long as one which would show a 1% difference between
the nucleotide sequences at the beginning and end of the branch. But we
would not expect the sequences at the beginning and end of the branch to be
26% different, as there would be some overlaying of changes.

One problem that can arise is that two or more of the species can be so
dissimilar that the distance between them would have to be infinite, as
the likelihood rises indefinitely as the estimated divergence time
increases. For example, with the Jukes-Cantor model, if the two sequences
differ in 75% or more of their positions then the estimate of dovergence
time would be infinite. Since there is no way to represent an infinite
distance in the output file, the program regards this as an error, issues an
error message indicating which pair of species are causing the problem, and
stops. It might be that, had it continued running, it would have also run
into the same problem with other pairs of species. If the Kimura
distance is being used there may be no error message; the program may
simply give a large distance value (it is iterating towards
infinity and the value is just where the iteration stopped). Likewise
some maximum likelihood estimates may also become large for the same
reason (the sequences showing more divergence than is expected even with
infinite branch length). I hope in the future to add more warning
messages that would alert the user the this.

If the similarity table is selected, the table that is produced is not
in a format that can be used as input to the distance matrix programs.
it has a heading, and the species names are also put at the tops of the
columns of the table (or rather, the first 8 characters of each species
name is there, the other two characters omitted to save space). There
is not an option to put the table into a format that can be read by
the distance matrix programs, nor is there one to make it into a table
of fractions of difference by subtracting the similarity values from 1.
This is done deliberately to make it more difficult for the use to
use these values to construct trees. The similarity values are
not corrected for multiple changes, and their use to construct trees
(even after converting them to fractions of difference) would be
wrong, as it would lead to severe conflict between the distant
pairs of sequences and the close pairs of sequences.

## PROGRAM CONSTANTS

The constants that are available to be changed by the user at the beginning
of the program include
"maxcategories", the maximum number of site
categories, "iterations", which controls the number of times
the program iterates the EM algorithm that is used to do the maximum
likelihood distance, "namelength", the length of species names in
characters, and "epsilon", a parameter which controls the accuracy of the
results of the iterations which estimate the distances. Making "epsilon"
smaller will increase run times but result in more decimal places of
accuracy. This should not be necessary.

The program spends most of its time doing real arithmetic.
The algorithm, with separate and independent computations
occurring for each pattern, lends itself readily to parallel processing.

---

### TEST DATA SET

|  |
| --- |
| ```    5   13 Alpha     AACGTGGCCACAT Beta      AAGGTCGCCACAC Gamma     CAGTTCGCCACAA Delta     GAGATTTCCGCCT Epsilon   GAGATCTCCGCCC ``` |

---

### CONTENTS OF OUTPUT FILE (with all numerical options on)

(Note that when the options for displaying the input data are turned off,
the output is in a form suitable for use as an input file in the distance
matrix programs).

|  |
| --- |
| ``` Nucleic acid sequence Distance Matrix program, version 3.66   5 species,  13  sites    F84 Distance  Transition/transversion ratio =   2.000000  Name            Sequences ----            ---------  Alpha        AACGTGGCCA CAT Beta         ..G..C.... ..C Gamma        C.GT.C.... ..A Delta        G.GA.TT..G .C. Epsilon      G.GA.CT..G .CC   Empirical Base Frequencies:     A       0.24615    C       0.36923    G       0.21538   T(U)     0.16923  Alpha       0.000000  0.303900  0.857544  1.158927  1.542899 Beta        0.303900  0.000000  0.339727  0.913522  0.619671 Gamma       0.857544  0.339727  0.000000  1.631729  1.293713 Delta       1.158927  0.913522  1.631729  0.000000  0.165882 Epsilon     1.542899  0.619671  1.293713  0.165882  0.000000 ``` |
