# Supplementary material for: Probabilistic Phylogenetic Inference with Insertions and Deletions
Source: PLoS Comput Biol. 2008 Sep 19;4(9):e1000172. doi: 10.1371/journal.pcbi.1000172 (PMC2527138; doi:10.1371/journal.pcbi.1000172)
Supplement: Dataset S1 — Supplemental Material (24.89 MB GZ) [file pcbi.1000172.s001.gz › erate-supplement-R2/src/phylip3.66-erate/doc/dolpenny.html]

dolpenny


version 3.66

# Dolpenny - Branch and bound to find all most parsimonious trees for Dollo, polymorphism parsimony criteria

© Copyright 1986-2006 by the University of
Washington. Written by Joseph Felsenstein. Permission is granted to copy
this document provided that no fee is charged for it and that this copyright
notice is not removed.

Dolpenny is a program that will find all of the most parsimonious trees
implied by your data when the Dollo or polymorphism parsimony criteria are
employed. It does so not by examining all possible trees,
but by using the more sophisticated "branch and bound" algorithm, a
standard computer science search strategy first applied to
phylogenetic inference by Hendy and Penny (1982). (J. S. Farris
[personal communication, 1975] had also suggested that this strategy,
which is well-known in computer science, might
be applied to phylogenies, but he did not publish this suggestion).

There is, however, a price to be paid for the certainty that one has
found all members of the set of most parsimonious trees. The problem of
finding these has been shown (Graham and Foulds, 1982; Day, 1983) to be
NP-complete, which is equivalent to saying that there is no
fast algorithm that is guaranteed to solve the problem in all cases
(for a discussion of NP-completeness, see the Scientific American
article by Lewis and Papadimitriou, 1978). The result is that
this program, despite its algorithmic sophistication, is VERY SLOW.

The program should be slower than the other tree-building programs
in the package, but useable up to about ten species. Above this it will
bog down rapidly, but exactly when depends on the data and on how much
computer time you have (it may be more effective in the hands of someone
who can let a microcomputer grind all night than for someone who
has the "benefit" of paying for time on the campus mainframe
computer). IT IS VERY IMPORTANT FOR YOU TO GET A FEEL FOR HOW LONG THE
PROGRAM WILL TAKE ON YOUR DATA. This can be done by running it on subsets
of the species, increasing the number of species in the run until you
either are able to treat the full data set or know that the program
will take unacceptably long on it. (Making a plot of the logarithm of run
time against species number may help to project run times).

## The Algorithm

The search strategy used by Dolpenny starts by making a tree consisting of the
first two species (the first three if the tree is to be unrooted). Then
it tries to add the next species in all possible places (there are three
of these). For each of the resulting trees it evaluates the number of
losses. It adds the next species to each of these, again in all
possible spaces. If this process would continue it would simply
generate all possible trees, of which there are a very large number even
when the number of species is moderate (34,459,425 with 10 species). Actually
it does not do this, because the trees are generated in a
particular order and some of them are never generated.

Actually the order in which trees are generated is not quite as
implied above, but is a "depth-first search". This means that first
one adds the third species in the first possible
place, then the fourth species in its first possible place, then
the fifth and so on until the first possible tree has been produced. Its
number of steps is evaluated. Then one "backtracks" by trying the
alternative placements of the last species. When these are exhausted
one tries the next placement of the next-to-last species. The order of
placement in a depth-first search is like this for a
four-species case (parentheses enclose monophyletic groups):

     Make tree of first two species     (A,B)  
          Add C in first place     ((A,B),C)  
               Add D in first place     (((A,D),B),C)  
               Add D in second place     ((A,(B,D)),C)  
               Add D in third place     (((A,B),D),C)  
               Add D in fourth place     ((A,B),(C,D))  
               Add D in fifth place     (((A,B),C),D)  
          Add C in second place: ((A,C),B)  
               Add D in first place     (((A,D),C),B)  
               Add D in second place     ((A,(C,D)),B)  
               Add D in third place     (((A,C),D),B)  
               Add D in fourth place     ((A,C),(B,D))  
               Add D in fifth place     (((A,C),B),D)  
          Add C in third place     (A,(B,C))  
               Add D in first place     ((A,D),(B,C))  
               Add D in second place     (A,((B,D),C))  
               Add D in third place     (A,(B,(C,D)))  
               Add D in fourth place     (A,((B,C),D))  
               Add D in fifth place     ((A,(B,C)),D)  

Among these fifteen trees you will find all of the four-species
rooted bifurcating trees, each exactly once (the parentheses each enclose
a monophyletic group). As displayed above, the backtracking
depth-first search algorithm is just another way of producing all
possible trees one at a time. The branch and bound algorithm
consists of this with one change. As each tree is constructed,
including the partial trees such as (A,(B,C)), its number of losses
(or retentions of polymorphism)
is evaluated.

The point of this is that if a previously-found
tree such as ((A,B),(C,D)) required fewer losses, then we know that
there is no point in even trying to add D to ((A,C),B). We have
computed the bound that enables us to cut off a whole line of inquiry
(in this case five trees) and avoid going down that particular branch
any farther.

The branch-and-bound algorithm thus allows us to find all most parsimonious
trees without generating all possible trees. How much of a saving this
is depends strongly on the data. For very clean (nearly "Hennigian")
data, it saves much time, but on very messy data it will still take
a very long time.

The algorithm in the program differs from the one outlined here
in some essential details: it investigates possibilities in the
order of their apparent promise. This applies to the order of addition
of species, and to the places where they are added to the tree. After
the first two-species tree is constructed, the program tries adding
each of the remaining species in turn, each in the best possible place it
can find. Whichever of those species adds (at a minimum) the most
additional steps is taken to be the one to be added next to the tree. When
it is added, it is added in turn to places which cause the fewest
additional steps to be added. This sounds a bit complex, but it is done
with the intention of eliminating regions of the search of all possible
trees as soon as possible, and lowering the bound on tree length as quickly
as possible.

The program keeps a list of all the most parsimonious
trees found so far. Whenever
it finds one that has fewer losses than
these, it clears out the list and
restarts the list with that tree. In the process the bound tightens and
fewer possibilities need be investigated. At the end the list contains
all the shortest trees. These are then printed out. It should be
mentioned that the program Clique for finding all largest cliques
also works by branch-and-bound. Both problems are NP-complete but for
some reason Clique runs far faster. Although their worst-case behavior
is bad for both programs, those worst cases occur far more frequently
in parsimony problems than in compatibility problems.

## Controlling Run Times

Among the quantities available to be set at the
beginning of a run of Dolpenny, two (howoften and howmany) are of particular
importance. As Dolpenny goes along it will keep count of how many
trees it has examined. Suppose that howoften is 100 and howmany is 300,
the default settings. Every time 100 trees have been examined, Dolpenny
will print out a line saying how many multiples of 100 trees have now been
examined, how many steps the most parsimonious tree found so far has,
how many trees of with that number of steps have been found, and a very
rough estimate of what fraction of all trees have been looked at so far.

When the number of these multiples printed out reaches the number howmany
(say 1000), the whole algorithm aborts and prints out that it has not
found all most parsimonious trees, but prints out what is has got so far
anyway. These trees need not be any of the most parsimonious trees: they are
simply the most parsimonious ones found so far. By setting the product
(howoften X howmany) large you can make
the algorithm less likely to abort, but then you risk getting bogged
down in a gigantic computation. You should adjust these constants so that
the program cannot go beyond examining the number of trees you are reasonably
willing to pay for (or wait for). In their initial setting the program will
abort after looking at 100,000 trees. Obviously you may want to adjust
howoften in order to get more or fewer lines of intermediate notice of how
many trees have been looked at so far. Of course, in small cases you may
never even reach the first multiple of howoften and nothing will be printed out
except some headings and then the final trees.

The indication of the approximate percentage of trees searched so far will
be helpful in judging how much farther you would have to go to get the full
search. Actually, since that fraction is the fraction of the set of all
possible trees searched or ruled out so far, and since the search becomes
progressively more efficient, the approximate fraction printed out will
usually be an underestimate of how far along the program is, sometimes a
serious underestimate.

A constant that affects the result is "maxtrees",
which controls the maximum number of trees that can be stored. Thus if
"maxtrees"
is 25, and 32 most parsimonious trees are found, only the first 25 of these are
stored and printed out. If "maxtrees"
is increased, the program does not run any slower but requires a little
more intermediate storage space. I recommend
that "maxtrees"
be kept as large as you can, provided you are willing to
look at an output with that many trees on it! Initially,
"maxtrees" is set to 100 in the distribution copy.

## Methods and Options

The counting of the length of trees is done by an algorithm nearly
identical to the corresponding algorithms in Dollop, and thus the remainder
of this document will be nearly identical to the Dollop document. The
Dollo parsimony method was
first suggested in print in verbal form by Le Quesne (1974) and was
first well-specified by Farris (1977). The method is named after Louis
Dollo since he was one of the first to assert that in evolution it is
harder to gain a complex feature than to lose it. The algorithm
explains the presence of the state 1 by allowing up to one forward
change 0-->1 and as many reversions 1-->0 as are necessary to explain
the pattern of states seen. The program attempts to minimize the number
of 1-->0 reversions necessary.

The assumptions of this method are in effect:

1. We know which state is the ancestral one (state 0).- The characters are evolving independently.- Different lineages evolve independently.- The probability of a forward change (0-->1) is small over the
         evolutionary times involved.- The probability of a reversion (1-->0) is also small, but
           still far larger than the probability of a forward change, so
           that many reversions are easier to envisage than even one
           extra forward change.- Retention of polymorphism for both states (0 and 1) is highly
             improbable.- The lengths of the segments of the true tree are not so
               unequal that two changes in a long segment are as probable as
               one in a short segment.

That these are the assumptions is established in several of my
papers (1973a, 1978b, 1979, 1981b, 1983). For an opposing view arguing
that the parsimony methods make no substantive
assumptions such as these, see the papers by Farris (1983) and Sober (1983a,
1983b), but also read the exchange between Felsenstein and Sober (1986).

One problem can arise when using additive binary recoding to
represent a multistate character as a series of two-state characters. Unlike
the Camin-Sokal, Wagner, and Polymorphism methods, the Dollo
method can reconstruct ancestral states which do not exist. An example
is given in my 1979 paper. It will be necessary to check the output to
make sure that this has not occurred.

The polymorphism parsimony method was first used by me,
and the results published
(without a clear
specification of the method) by Inger (1967). The method was
published by Farris (1978a) and by me (1979). The method
assumes that we can explain the pattern of states by no more than one
origination (0-->1) of state 1, followed by retention of polymorphism
along as many segments of the tree as are necessary, followed by loss of
state 0 or of state 1 where necessary. The program tries to minimize
the total number of polymorphic characters, where each polymorphism is
counted once for each segment of the tree in which it is retained.

The assumptions of the polymorphism parsimony method are in effect:

1. The ancestral state (state 0) is known in each character.- The characters are evolving independently of each other.- Different lineages are evolving independently.- Forward change (0-->1) is highly improbable over the length of
         time involved in the evolution of the group.- Retention of polymorphism is also improbable, but far more
           probable that forward change, so that we can more easily
           envisage much polymorhism than even one additional forward
           change.- Once state 1 is reached, reoccurrence of state 0 is very
             improbable, much less probable than multiple retentions of
             polymorphism.- The lengths of segments in the true tree are not so unequal
               that we can more easily envisage retention events occurring in
               both of two long segments than one retention in a short
               segment.

That these are the assumptions of parsimony methods has been documented
in a series of papers of mine: (1973a, 1978b, 1979, 1981b,
1983b, 1988b). For an opposing view arguing that the parsimony methods
make no substantive
assumptions such as these, see the papers by Farris (1983) and Sober (1983a,
1983b), but also read the exchange between Felsenstein and Sober (1986).

The input format is the standard one, with "?", "P", "B" states
allowed. Most of the options are selected using a menu:

|  |
| --- |
| ``` Penny algorithm for Dollo or polymorphism parsimony, version 3.6  branch-and-bound to find all most parsimonious trees  Settings for this run:   P                     Parsimony method?  Dollo   H        How many groups of  100 trees:  1000   F        How often to report, in trees:  100   S           Branch and bound is simple?  Yes   T              Use Threshold parsimony?  No, use ordinary parsimony   A                 Use ancestral states?  No   W                       Sites weighted?  No   M           Analyze multiple data sets?  No   0   Terminal type (IBM PC, ANSI, none)?  ANSI   1    Print out the data at start of run  No   2  Print indications of progress of run  Yes   3                        Print out tree  Yes   4     Print out steps in each character  No   5     Print states at all nodes of tree  No   6       Write out trees onto tree file?  Yes  Are these settings correct? (type Y or the letter for one to change) ``` |

The P option toggles between the Polymorphism parsimony method and the
default Dollo parsimony method.

The options T, A, and M are the usual Threshold, Ancestral
States, and Multiple Data Sets options. They are described in the Main
documentation file and in the Discrete Characters Programs documentation
file.

Options F and H reset the
variables howoften (F) and howmany (H). The user is prompted for the new
values. By setting these larger the program will report its progress less
often (howoften) and will run longer (howmany times howoften). These values
default to 100 and 1000 which
guarantees a search of 100,000 trees, but these can be changed. Note that
option F in this program is not the Factors option available in some of
the other programs in this section of the package.

The use of the A
option allows implementation of the unordered Dollo parsimony and unordered
polymorphism parsimony methods which I have
described elsewhere (1984b). When the A option is used the ancestor is
not to be counted as one of the species. The O (outgroup) option is not
available since the tree produced is already rooted.

Setting T at or below
1.0 but above 0 causes the criterion to become compatibility rather than
polymorphism parsimony, although there is no advantage to using this
program instead of Penny to do a compatibility method. Setting
the threshold value higher brings about an intermediate between
the Dollo or polymorphism parsimony methods and the compatibility method,
so that there is some rationale for doing that.

Using a threshold value of 1.0 or lower, but above 0, one can
obtain a rooted (or, if the A option is used with ancestral states of
"?", unrooted) compatibility criterion, but there is no particular
advantage to using this program for that instead of MIX. Higher
threshold values are of course meaningful and provide
intermediates between Dollo and compatibility methods.

The S (Simple) option alters a step in Dolpenny which reconsiders the
order in which species are added to the tree. Normally the decision as to
what species to add to the tree next is made as the first tree is being
constructucted; that ordering of species is not altered subsequently. The
R option causes it to be continually reconsidered. This will probably
result in a substantial increase in run time, but on some data sets of
intermediate messiness it may help. It is included in case it might prove
of use on some data sets. The Simple option, in which the ordering is
kept the same after being established by trying alternatives during
the construction of the first tree, is the default. Continual reconsideration
can be selected as an alternative.

The Factors
option is not available in this program, as it would have no effect on
the result even if that information were provided in the input file.

The output format is also standard. It includes a rooted tree and,
if the user selects option 4, a table
of the numbers of reversions or retentions of polymorphism necessary
in each character. If any of the
ancestral states has been specified to be unknown, a table of
reconstructed ancestral states is also provided. When reconstructing
the placement of forward changes and reversions under the Dollo method,
keep in mind that each
polymorphic state in the input data will require one "last minute"
reversion. This is included in the tabulated counts. Thus if we have
both states 0 and 1 at a tip of the tree the program will assume that
the lineage had state 1 up to the last minute, and then state 0 arose in
that population by reversion, without loss of state 1.

A table is available to be printed out after each tree, showing for each
branch whether
there are known to be changes in the branch, and what the states are inferred
to have been at the top end of the branch. If the inferred state is a "?"
there will be multiple equally-parsimonious assignments of states; the user
must work these out for themselves by hand.

If the A option is used, then the program will
infer, for any character whose ancestral state is unknown ("?") whether the
ancestral state 0 or 1 will give the best tree. If these are
tied, then it may not be possible for the program to infer the
state in the internal nodes, and these will all be printed as ".". If this
has happened and you want to know more about the states at the internal
nodes, you will find helpful to use Dolmove to display the tree and examine
its interior states, as the algorithm in Dolmove shows all that can be known
in this case about the interior states, including where there is and is not
amibiguity. The algorithm in Dolpenny gives up more easily on displaying these
states.

If option 6 is left in its default state the trees
found will be written to a tree file, so that they are available to be used
in other programs. If the program finds multiple
trees tied for best, all of these are written out onto the output tree
file. Each is followed by a numerical weight in square brackets (such as
[0.25000]). This is needed when we use the trees to make a consensus
tree of the results of bootstrapping or jackknifing, to avoid overrepresenting
replicates that find many tied trees.

At the beginning of the program are a series of constants,
which can be changed to help adapt the program to different computer systems.
Two are the initial values of howmany and howoften,
constants "often" and "many". Constant "maxtrees"
is the maximum number of tied trees that will
be stored.

---

### TEST DATA SET

|  |
| --- |
| ```     7    6 Alpha1    110110 Alpha2    110110 Beta1     110000 Beta2     110000 Gamma1    100110 Delta     001001 Epsilon   001110 ``` |

---

### TEST SET OUTPUT (with all numerical options turned on)

|  |
| --- |
| ``` Penny algorithm for Dollo or polymorphism parsimony, version 3.66  branch-and-bound to find all most parsimonious trees   7 species,   6 characters Dollo parsimony method   Name         Characters ----         ----------  Alpha1       11011 0 Alpha2       11011 0 Beta1        11000 0 Beta2        11000 0 Gamma1       10011 0 Delta        00100 1 Epsilon      00111 0   requires a total of              3.000      3 trees in all found     +-----------------Delta        !   --2  +--------------Epsilon      !  !     +--3  +-----------Gamma1          !  !        +--6  +--------Alpha2             !  !           +--1     +--Beta2                 !  +--5              +--4  +--Beta1                    !                 +-----Alpha1        reversions in each character:          0   1   2   3   4   5   6   7   8   9      *-----------------------------------------     0!       0   0   1   1   1   0              From    To     Any Steps?    State at upper node                              ( . means same as in the node below it on tree)  root      2         yes    ..1.. .   2    Delta        yes    ..... 1   2       3         yes    ...11 .   3    Epsilon      no     ..... .   3       6         yes    1.0.. .   6    Gamma1       no     ..... .   6       1         yes    .1... .   1    Alpha2       no     ..... .   1       4         no     ..... .   4       5         yes    ...00 .   5    Beta2        no     ..... .   5    Beta1        no     ..... .   4    Alpha1       no     ..... .     +-----------------Delta        !   --2  +--------------Epsilon      !  !     +--3  +-----------Gamma1          !  !        +--6        +--Beta2              !  +-----5           !  !     +--Beta1              +--4              !     +--Alpha2                +-----1                    +--Alpha1        reversions in each character:          0   1   2   3   4   5   6   7   8   9      *-----------------------------------------     0!       0   0   1   1   1   0              From    To     Any Steps?    State at upper node                              ( . means same as in the node below it on tree)  root      2         yes    ..1.. .   2    Delta        yes    ..... 1   2       3         yes    ...11 .   3    Epsilon      no     ..... .   3       6         yes    1.0.. .   6    Gamma1       no     ..... .   6       4         yes    .1... .   4       5         yes    ...00 .   5    Beta2        no     ..... .   5    Beta1        no     ..... .   4       1         no     ..... .   1    Alpha2       no     ..... .   1    Alpha1       no     ..... .     +-----------------Delta        !   --2  +--------------Epsilon      !  !     +--3  +-----------Gamma1          !  !        !  !        +--Beta2           +--6     +--5           !  +--4  +--Beta1              !  !  !           +--1  +-----Alpha2                !              +--------Alpha1        reversions in each character:          0   1   2   3   4   5   6   7   8   9      *-----------------------------------------     0!       0   0   1   1   1   0              From    To     Any Steps?    State at upper node                              ( . means same as in the node below it on tree)  root      2         yes    ..1.. .   2    Delta        yes    ..... 1   2       3         yes    ...11 .   3    Epsilon      no     ..... .   3       6         yes    1.0.. .   6    Gamma1       no     ..... .   6       1         yes    .1... .   1       4         no     ..... .   4       5         yes    ...00 .   5    Beta2        no     ..... .   5    Beta1        no     ..... .   4    Alpha2       no     ..... .   1    Alpha1       no     ..... . ``` |
