# Supplementary material for: Probabilistic Phylogenetic Inference with Insertions and Deletions
Source: PLoS Comput Biol. 2008 Sep 19;4(9):e1000172. doi: 10.1371/journal.pcbi.1000172 (PMC2527138; doi:10.1371/journal.pcbi.1000172)
Supplement: Dataset S1 — Supplemental Material (24.89 MB GZ) [file pcbi.1000172.s001.gz › erate-supplement-R2/src/phylip3.66-erate/doc/dollop.html]

dollop


version 3.66

# Dollop -- Dollo and Polymorphism Parsimony Program

© Copyright 1986-2006 by the University of
Washington. Written by Joseph Felsenstein. Permission is granted to copy
this document provided that no fee is charged for it and that this copyright
notice is not removed.

This program carries out the Dollo and polymorphism parsimony methods. The
Dollo parsimony method was
first suggested in print in verbal form by Le Quesne (1974) and was
first well-specified by Farris (1977). The method is named after Louis
Dollo since he was one of the first to assert that in evolution it is
harder to gain a complex feature than to lose it. The algorithm
explains the presence of the state 1 by allowing up to one forward
change 0-->1 and as many reversions 1-->0 as are necessary to explain
the pattern of states seen. The program attempts to minimize the number
of 1-->0 reversions necessary.

The assumptions of this method are in effect:

1. We know which state is the ancestral one (state 0).- The characters are evolving independently.- Different lineages evolve independently.- The probability of a forward change (0-->1) is small over the
         evolutionary times involved.- The probability of a reversion (1-->0) is also small, but
           still far larger than the probability of a forward change, so
           that many reversions are easier to envisage than even one
           extra forward change.- Retention of polymorphism for both states (0 and 1) is highly
             improbable.- The lengths of the segments of the true tree are not so
               unequal that two changes in a long segment are as probable as
               one in a short segment.

One problem can arise when using additive binary recoding to
represent a multistate character as a series of two-state characters. Unlike
the Camin-Sokal, Wagner, and Polymorphism methods, the Dollo
method can reconstruct ancestral states which do not exist. An example
is given in my 1979 paper. It will be necessary to check the output to
make sure that this has not occurred.

The polymorphism parsimony method was first used by me, and the results
published (without a clear
specification of the method) by Inger (1967). The method was
independently published by Farris (1978a) and by me (1979). The method
assumes that we can explain the pattern of states by no more than one
origination (0-->1) of state 1, followed by retention of polymorphism
along as many segments of the tree as are necessary, followed by loss of
state 0 or of state 1 where necessary. The program tries to minimize
the total number of polymorphic characters, where each polymorphism is
counted once for each segment of the tree in which it is retained.

The assumptions of the polymorphism parsimony method are in effect:

1. The ancestral state (state 0) is known in each character.- The characters are evolving independently of each other.- Different lineages are evolving independently.- Forward change (0-->1) is highly improbable over the length of
         time involved in the evolution of the group.- Retention of polymorphism is also improbable, but far more
           probable that forward change, so that we can more easily
           envisage much polymorhism than even one additional forward
           change.- Once state 1 is reached, reoccurrence of state 0 is very
             improbable, much less probable than multiple retentions of
             polymorphism.- The lengths of segments in the true tree are not so unequal
               that we can more easily envisage retention events occurring in
               both of two long segments than one retention in a short
               segment.

That these are the assumptions of parsimony methods has been documented
in a series of papers of mine: (1973a, 1978b, 1979, 1981b,
1983b, 1988b). For an opposing view arguing that the parsimony methods
make no substantive
assumptions such as these, see the papers by Farris (1983) and Sober (1983a,
1983b), but also read the exchange between Felsenstein and Sober (1986).

The input format is the standard one, with "?", "P", "B" states
allowed. The options are selected using a menu:

|  |
| --- |
| ``` Dollo and polymorphism parsimony algorithm, version 3.6  Settings for this run:   U                 Search for best tree?  Yes   P                     Parsimony method?  Dollo   J     Randomize input order of species?  No. Use input order   T              Use Threshold parsimony?  No, use ordinary parsimony   A   Use ancestral states in input file?  No   W                       Sites weighted?  No   M           Analyze multiple data sets?  No   0   Terminal type (IBM PC, ANSI, none)?  ANSI   1    Print out the data at start of run  No   2  Print indications of progress of run  Yes   3                        Print out tree  Yes   4     Print out steps in each character  No   5     Print states at all nodes of tree  No   6       Write out trees onto tree file?  Yes  Are these settings correct? (type Y or the letter for one to change) ``` |

The options U, J, T, A, and M are the usual User Tree, Jumble,
Ancestral States, and Multiple Data Sets options, described either
in the main documentation file or in the Discrete Characters Programs
documentation file. The A (Ancestral States)
option allows implementation of the unordered Dollo parsimony and unordered
polymorphism parsimony methods which I have
described elsewhere (1984b). When the A option is used the ancestor is
not to be counted as one of the species. The O (outgroup) option is not
available since the tree produced is already rooted. Since the Dollo and
polymorphism methods produce a rooted
tree, the user-defined trees required by the U option have two-way forks
at each level.

The P (Parsimony Method) option is the one that toggles between polymorphism
parsimony and Dollo parsimony. The program defaults to Dollo parsimony.

The T (Threshold) option has already been described in
the Discrete Characters programs documentation file. Setting T at or below
1.0 but above 0 causes the criterion to become compatibility rather than
polymorphism parsimony, although there is no advantage to using this
program instead of MIX to do a compatibility method. Setting the
threshold value higher brings about an intermediate between
the Dollo or polymorphism parsimony methods and the compatibility method,
so that there is some rationale for doing that. Since the Dollo and
polymorphism methods produces a rooted
tree, the user-defined trees required by the U option have two-way forks
at each level.

Using a threshold value of 1.0 or lower, but above 0, one can
obtain a rooted (or, if the A option is used with ancestral states of
"?", unrooted) compatibility criterion, but there is no particular
advantage to using this program for that instead of MIX. Higher
threshold values are of course meaningful and provide
intermediates between Dollo and compatibility methods.

The X
(Mixed parsimony methods) option is not available in this program. The
Factors option is also not available in this program, as it would have no
effect on the result even if that information were provided in the input file.

Output is standard: a list of equally parsimonious trees, and, if the
user selects menu option 4, a table
of the numbers of reversions or retentions of polymorphism necessary
in each character. If any of the
ancestral states has been specified to be unknown, a table of
reconstructed ancestral states is also provided. When reconstructing
the placement of forward changes and reversions under the Dollo method,
keep in mind that each
polymorphic state in the input data will require one "last minute"
reversion. This is included in the tabulated counts. Thus if we have
both states 0 and 1 at a tip of the tree the program will assume that
the lineage had state 1 up to the last minute, and then state 0 arose in
that population by reversion, without loss of state 1.

If the user selects menu option 5, a table is printed out after each
tree, showing for each branch whether
there are known to be changes in the branch, and what the states are inferred
to have been at the top end of the branch. If the inferred state is a "?"
there may be multiple equally-parsimonious assignments of states; the user
must work these out for themselves by hand.

If the A option is used, then the program will
infer, for any character whose ancestral state is unknown ("?") whether the
ancestral state 0 or 1 will give the best tree. If these are
tied, then it may not be possible for the program to infer the
state in the internal nodes, and these will all be printed as ".". If this
has happened and you want to know more about the states at the internal
nodes, you will find helpful to use Dolmove to display the tree and examine
its interior states, as the algorithm in Dolmove shows all that can be known
in this case about the interior states, including where there is and is not
amibiguity. The algorithm in Dollop gives up more easily on displaying these
states.

If the U (User Tree) option is used and more than one tree is supplied, the
program also performs a statistical test of each of these trees against the
best tree. This test, which is a version of the test proposed by
Alan Templeton (1983) and evaluated in a test case by me (1985a). It is
closely parallel to a test using log likelihood differences
invented by Kishino and Hasegawa (1989), and uses the mean and variance of
step differences between trees, taken across characters. If the mean
is more than 1.96 standard deviations different then the trees are declared
significantly different. The program
prints out a table of the steps for each tree, the differences of
each from the highest one, the variance of that quantity as determined by
the step differences at individual characters, and a conclusion as to
whether that tree is or is not significantly worse than the best one. It
is important to understand that the test assumes that all the binary
characters are evolving independently, which is unlikely to be true for
many suites of morphological characters.

If there are more than two trees, the test done is an extension of
the KHT test, due to Shimodaira and Hasegawa (1999). They pointed out
that a correction for the number of trees was necessary, and they
introduced a resampling method to make this correction. In the version
used here the variances and covariances of the sums of steps across
characters are computed for all pairs of trees. To test whether the
difference between each tree and the best one is larger than could have
been expected if they all had the same expected number of steps,
numbers of steps for all trees are sampled with these covariances and equal
means (Shimodaira and Hasegawa's "least favorable hypothesis"),
and a P value is computed from the fraction of times the difference between
the tree's value and the lowest number of steps exceeds that actually
observed. Note that this sampling needs random numbers, and so the
program will prompt the user for a random number seed if one has not
already been supplied. With the two-tree KHT test no random numbers
are used.

In either the KHT or the SH test the program
prints out a table of the number of steps for each tree, the differences of
each from the lowest one, the variance of that quantity as determined by
the differences of the numbers of steps at individual characters,
and a conclusion as to
whether that tree is or is not significantly worse than the best one.

If option 6 is left in its default state the trees
found will be written to a tree file, so that they are available to be used
in other programs. If the program finds multiple
trees tied for best, all of these are written out onto the output tree
file. Each is followed by a numerical weight in square brackets (such as
[0.25000]). This is needed when we use the trees to make a consensus
tree of the results of bootstrapping or jackknifing, to avoid overrepresenting
replicates that find many tied trees.

At the beginning of the program is the constant
"maxtrees", the maximum number of trees which the program will store for
output.

The algorithm is a fairly simple adaptation of the one used in
the program Sokal, which was formerly in this package and has been
superseded by Mix. It requires two passes through each tree to count the
numbers of reversions.

---

### TEST DATA SET

|  |
| --- |
| ```      5    6 Alpha     110110 Beta      110000 Gamma     100110 Delta     001001 Epsilon   001110 ``` |

---

### TEST SET OUTPUT (with all numerical options on)

|  |
| --- |
| ``` Dollo and polymorphism parsimony algorithm, version 3.66  Dollo parsimony method   5 species,   6  characters   Name         Characters ----         ----------  Alpha        11011 0 Beta         11000 0 Gamma        10011 0 Delta        00100 1 Epsilon      00111 0   One most parsimonious tree found:     +-----------Delta      --3     !  +--------Epsilon      +--4        !  +-----Gamma           +--2           !  +--Beta               +--1              +--Alpha        requires a total of      3.000   reversions in each character:          0   1   2   3   4   5   6   7   8   9      *-----------------------------------------     0!       0   0   1   1   1   0              From    To     Any Steps?    State at upper node                              ( . means same as in the node below it on tree)  root      3         yes    ..1.. .   3    Delta        yes    ..... 1   3       4         yes    ...11 .   4    Epsilon      no     ..... .   4       2         yes    1.0.. .   2    Gamma        no     ..... .   2       1         yes    .1... .   1    Beta         yes    ...00 .   1    Alpha        no     ..... . ``` |
