# Supplementary material for: Probabilistic Phylogenetic Inference with Insertions and Deletions
Source: PLoS Comput Biol. 2008 Sep 19;4(9):e1000172. doi: 10.1371/journal.pcbi.1000172 (PMC2527138; doi:10.1371/journal.pcbi.1000172)
Supplement: Dataset S1 — Supplemental Material (24.89 MB GZ) [file pcbi.1000172.s001.gz › erate-supplement-R2/src/phylip3.66-erate/doc/contml.html]

contml


version 3.66

# Contml - Gene Frequencies and Continuous Characters Maximum Likelihood method

© Copyright 1986-2006 by the University of
Washington. Written by Joseph Felsenstein. Permission is granted to copy
this document provided that no fee is charged for it and that this copyright
notice is not removed.

This program estimates phylogenies by the restricted maximum likelihood method
based on the Brownian motion model. It is based on the model of Edwards and
Cavalli-Sforza (1964; Cavalli-Sforza and Edwards, 1967). Gomberg (1966),
Felsenstein (1973b, 1981c) and Thompson (1975) have done extensive further work
leading to efficient algorithms. Contml uses restricted maximum
likelihood estimation (REML), which is the criterion used by Felsenstein
(1973b). The actual algorithm is an iterative EM Algorithm (Dempster,
Laird, and Rubin, 1977) which is guaranteed to always give increasing
likelihoods. The algorithm is described in detail in a paper of mine
(Felsenstein, 1981c), which you should definitely consult if you are
going to use this program. Some simulation tests of it are given
by Rohlf and Wooten (1988) and Kim and Burgman (1988).

The default (gene frequency) mode treats the input as gene frequencies at a
series of loci, and
square-root-transforms the allele frequencies (constructing the frequency of
the missing allele at each locus first). This enables us to use the
Brownian motion model on the resulting coordinates, in an approximation
equivalent to using Cavalli-Sforza and Edwards's (1967) chord measure
of genetic distance and taking that to give distance between particles
undergoing pure Brownian motion. It assumes that each locus evolves
independently by pure genetic drift.

The alternative continuous characters mode (menu option C) treats the input
as a series of coordinates of each species in N dimensions. It assumes
that we have transformed the characters to remove correlations and to
standardize their variances.

## A word about microsatellite data

Many current users of Contml use it to analyze microsatellite data.
There are three ways to do this:

- Coding each copy number as an allele, and feeding in the
  frequencies of these alleles. As Contml's gene frequency mode assumes that
  all change is by genetic drift, this means that no copy number arises by
  mutation during the divergence of the populations. Since microsatellite
  loci have very high mutation rates, this is questionable.- Use some other
    program, one not in the PHYLIP package, to compute distances among the
    populations. Some of the programs that can do this are RSTCalc, poptrfdos,
    Microsat, and Populations. Links to them can be found at my Phylogeny
    Programs web site at 
    `http://evolution.gs.washington.edu/phylip/software.html`.

    Those distance measures allow for mutation during the divergence of the
    populations. But even they are not perfect -- they do not allow us to use
    all the information contained in the gene frequency differences of
    within a copy number allele. There is a need for a more complete
    statistical treatment of inference of phylogenies from microsatellite models,
    ones that take both mutation and genetic drift fully into account.- Alternatively, there is the Brownian motion approximation to mean population
      copy number. This is described in my book (Felsenstein, 2004, Chapter 15,
      pp. 242-245), and it is implicit also in the microsatellite distances.
      Each locus is coded as a single continuous character, the mean of the copy
      number in at that microsatellite locus in that species. Thus if the species
      (or population) has frequencies 0.10, 0.24, 0.60, and 0.06 of alleles that
      have 18, 19, 20, and 21 copies, it is coded as having

      0.10 X 18 + 0.24 X 19 + 0.60 X 20 + 0.06 X 21   =  19.62

      copies. These values can, I believe, be calculated by a spreadsheet program.
      Each microsatellite is represented by one character, and the continuous
      character mode of Contml is used (not the gene frequencies mode). This
      coding allows for mutation that changes copy number. It
      does not make complete use of all data, but neither does the treatment
      of microsatellite gene frequencies as changing only genetic drift.
      frequency

## The input file

The input file is as described in the continuous characters
documentation file above. Options are selected using a menu:

|  |
| --- |
| ``` Continuous character Maximum Likelihood method version 3.6  Settings for this run:   U                       Search for best tree?  Yes   C  Gene frequencies or continuous characters?  Gene frequencies   A   Input file has all alleles at each locus?  No, one allele missing at each   O                              Outgroup root?  No, use as outgroup species 1   G                      Global rearrangements?  No   J           Randomize input order of species?  No. Use input order   M                 Analyze multiple data sets?  No   0         Terminal type (IBM PC, ANSI, none)?  ANSI   1          Print out the data at start of run  No   2        Print indications of progress of run  Yes   3                              Print out tree  Yes   4             Write out trees onto tree file?  Yes    Y to accept these or type the letter for one to change ``` |

Option U is the usual User Tree option. Options C (Continuous Characters)
and A (All alleles present) have been described
in the Gene Frequencies and Continuous Characters Programs documentation
file. The options G, J, O and M are the usual Global Rearrangements, Jumble
order of species, Outgroup root, and Multiple Data Sets options.

The M (Multiple data sets) option does not allow multiple sets of weights
instead of multiple data sets, as there are no weights in this program.

The G and J options have no effect if the User Tree option is selected. User
trees are given with a trifurcation (three-way split) at the base. They
can start from any interior node. Thus the tree:

```
     A
     !
     *--B
     !
     *-----C
     !
     *--D
     !
     E
```

can be represented by any of the following:

```
     (A,B,(C,(D,E)));
     ((A,B),C,(D,E));
     (((A,B),C),D,E);
```

(there are of course 69 other representations as well obtained from these
by swapping the order of branches at an interior node).

## The output file

The output has a standard appearance. The topology of the tree
is given by an unrooted tree diagram. The lengths (in time or in
expected amounts of variance) are given in a table below the topology,
and a rough confidence interval given for each length. Negative lower
bounds on length indicate that rearrangements may be acceptable.

The units of length are amounts of expected accumulated variance (not
time). The
log likelihood (natural log) of each tree is also given, and it is
indicated how many topologies have been tried. The tree does not
necessarily have all tips contemporary, and the log likelihood may be
either positive or negative (this simply corresponds to whether the
density function does or does not exceed 1) and a negative log
likelihood does not indicate any error. The log likelihood allows
various formal likelihood ratio hypothesis tests. The description of
the tree includes approximate standard errors on the lengths of segments
of the tree. These are calculated by considering only the curvature of
the likelihood surface as the length of the segment is varied, holding
all other lengths constant. As such they are most probably underestimates of
the variance, and hence may give too much confidence in the given tree.

One should use caution in interpreting the likelihoods that are printed
out. If the model is wrong, it will not be possible to use the
likelihoods to make formal statistical statements. Thus, if gene
frequencies are being analyzed, but the gene frequencies change not only
by genetic drift, but also by mutation, the model is not correct. It
would be as well-justified in this case to use Gendist to compute the
Nei (1972) genetic distance and then use Fitch, Kitsch or Neighbor to make a
tree. If continuous characters are being analyzed, but if the
characters have not been transformed to new coordinates that evolve
independently and at equal rates, then the model is also violated and no
statistical analysis is possible. Doing such a transformation is not
easy, and usually not even possible.

If the U (User Tree) option is used and more than one tree is supplied,
the program also performs a statistical test of each of these trees against the
one with highest likelihood. If there are two user trees, the test
done is one which is due to Kishino and Hasegawa (1989), a version
of a test originally introduced by Templeton (1983). In this
implementation it uses the mean and variance of
log-likelihood differences between trees, taken across loci. If the two
trees means are more than 1.96 standard deviations different then the trees are
declared significantly different. This use of the empirical variance of
log-likelihood differences is more robust and nonparametric than the
classical likelihood ratio test, and may to some extent compensate for the
any lack of realism in the model underlying this program.

If there are more than two trees, the test done is an extension of
the KHT test, due to Shimodaira and Hasegawa (1999). They pointed out
that a correction for the number of trees was necessary, and they
introduced a resampling method to make this correction. The version
used here is a multivariate normal approximation to their test; it is
due to Shimodaira (1998). The variances and covariances of the sum of
log likelihoods across loci are computed for all pairs of trees. To test
whether the difference between each tree and the best one is larger than
could have been expected if they all had the same expected log-likelihood,
log-likelihoods for all trees are sampled with these covariances and equal
means (Shimodaira and Hasegawa's "least favorable hypothesis"),
and a P value is computed from the fraction of times the difference between
the tree's value and the highest log-likelihood exceeds that actually
observed. Note that this sampling needs random numbers, and so the
program will prompt the user for a random number seed if one has not
already been supplied. With the two-tree KHT test no random numbers
are used.

In either the KHT or the SH test the program
prints out a table of the log-likelihoods of each tree, the differences of
each from the highest one, the variance of that quantity as determined by
the log-likelihood differences at individual sites, and a conclusion as to
whether that tree is or is not significantly worse than the best one.

One problem which sometimes arises is that the program is fed two species
(or populations) with identical transformed gene frequencies: this can
happen if sample sizes are small and/or many loci are monomorphic. In
this case the program "gets its knickers in a twist" and can divide by
zero, usually causing a crash. If you suspect that this has happened,
check for two species with identical coordinates. If you find them,
eliminate one from the problem: the two must always show up as being at the
same point on the tree anyway.

The constants
available for modification at the beginning of the
program include "epsilon1",
a small quantity used in the iterations of branch lengths,
"epsilon2", another not quite so small quantity used to check
whether gene frequencies that were fed in for all alleles do not add up to 1,
"smoothings", the number of passes through a
given tree in the iterative likelihood maximization for a given topology,
"maxtrees", the maximum number of user trees that will be used for the
Kishino-Hasegawa-Templeton test, and
"namelength", the length of species names.
There is no provision in this program for saving multiple trees that are
tied for having the highest likelihood, mostly because an exact tie is
unlikely anyway.

The algorithm does not run as quickly as the discrete character
methods but is not enormously slower. Like them, its execution time
should rise as the cube of the number of species.

### TEST DATA SET

This data set was compiled by me from the compilation of human gene
frequencies by Mourant (1976). It appeared in a paper of mine
(Felsenstein, 1981c) on maximum likelihood phylogenies from gene
frequencies. The names of the loci and alleles are given in that
paper.

|  |
| --- |
| ```     5    10 2 2 2 2 2 2 2 2 2 2 European   0.2868 0.5684 0.4422 0.4286 0.3828 0.7285 0.6386 0.0205 0.8055 0.5043 African    0.1356 0.4840 0.0602 0.0397 0.5977 0.9675 0.9511 0.0600 0.7582 0.6207 Chinese    0.1628 0.5958 0.7298 1.0000 0.3811 0.7986 0.7782 0.0726 0.7482 0.7334 American   0.0144 0.6990 0.3280 0.7421 0.6606 0.8603 0.7924 0.0000 0.8086 0.8636 Australian 0.1211 0.2274 0.5821 1.0000 0.2018 0.9000 0.9837 0.0396 0.9097 0.2976 ``` |

---

### TEST SET OUTPUT (WITH ALL NUMERICAL OPTIONS TURNED ON)

|  |
| --- |
| ``` Continuous character Maximum Likelihood method version 3.66      5 Populations,   10 Loci  Numbers of alleles at the loci: ------- -- ------- -- --- -----     2   2   2   2   2   2   2   2   2   2  Name                 Gene Frequencies ----                 ---- -----------    locus:         1         2         3         4         5         6                  7         8         9        10  European     0.28680   0.56840   0.44220   0.42860   0.38280   0.72850              0.63860   0.02050   0.80550   0.50430 African      0.13560   0.48400   0.06020   0.03970   0.59770   0.96750              0.95110   0.06000   0.75820   0.62070 Chinese      0.16280   0.59580   0.72980   1.00000   0.38110   0.79860              0.77820   0.07260   0.74820   0.73340 American     0.01440   0.69900   0.32800   0.74210   0.66060   0.86030              0.79240   0.00000   0.80860   0.86360 Australian   0.12110   0.22740   0.58210   1.00000   0.20180   0.90000              0.98370   0.03960   0.00000   0.00000     +--------------African      !     !      +-------American     1------2     !      !       +--------------------------------------------Australian   !      +-------3     !              +Chinese      !     +European     remember: this is an unrooted tree!  Ln Likelihood =    40.99979  Between     And             Length      Approx. Confidence Limits -------     ---             ------      ------- ---------- ------   1       African        0.03718970   (  0.01198514,  0.07616999)   1          2           0.01711139   ( -0.00170355,  0.04620978)   2       American       0.02037005   (  0.00000023,  0.05187314)   2          3           0.02047369   ( -0.00095607,  0.05361605)   3       Australian     0.11225916   (  0.03498079,  0.23177459)   3       Chinese        0.00187759   ( -0.01534330,  0.02851067)   1       European       0.00000000   ( -0.01077301,  0.01666107) ``` |
