# Supplementary material for: Probabilistic Phylogenetic Inference with Insertions and Deletions
Source: PLoS Comput Biol. 2008 Sep 19;4(9):e1000172. doi: 10.1371/journal.pcbi.1000172 (PMC2527138; doi:10.1371/journal.pcbi.1000172)
Supplement: Dataset S1 — Supplemental Material (24.89 MB GZ) [file pcbi.1000172.s001.gz › erate-supplement-R2/src/phylip3.66-erate/doc/mix.html]

mix


version 3.66

# Mix - Mixed method discrete characters parsimony

© Copyright 1986-2006 by the University of
Washington. Written by Joseph Felsenstein. Permission is granted to copy
this document provided that no fee is charged for it and that this copyright
notice is not removed.

Mix is a general parsimony program which carries out the Wagner and
Camin-Sokal parsimony methods in mixture, where each character can have
its method specified separately. The program defaults to carrying out Wagner
parsimony.

The Camin-Sokal parsimony method explains the data by assuming that
changes 0 --> 1 are allowed but not changes 1 --> 0. Wagner parsimony
allows both kinds of changes. (This under the assumption that 0 is the
ancestral state, though the program allows reassignment of the ancestral
state, in which case we must reverse the state numbers 0 and 1
throughout this discussion). The criterion is to find the tree which
requires the minimum number of changes. The Camin-Sokal method is due
to Camin and Sokal (1965) and the Wagner method to Eck and Dayhoff
(1966) and to Kluge and Farris (1969).

Here are the assumptions of these two methods:

1. Ancestral states are known (Camin-Sokal) or unknown (Wagner).- Different characters evolve independently.- Different lineages evolve independently.- Changes 0 --> 1 are much more probable than changes 1 --> 0
         (Camin-Sokal) or equally probable (Wagner).- Both of these kinds of changes are a priori improbable over the
           evolutionary time spans involved in the differentiation of the
           group in question.- Other kinds of evolutionary event such as retention of polymorphism
             are far less probable than 0 --> 1 changes.- Rates of evolution in different lineages are sufficiently low that
               two changes in a long segment of the tree are far less probable
               than one change in a short segment.

That these are the assumptions of parsimony methods has been documented
in a series of papers of mine: (1973a, 1978b, 1979, 1981b,
1983b, 1988b). For an opposing view arguing that the parsimony methods
make no substantive
assumptions such as these, see the papers by Farris (1983) and Sober (1983a,
1983b), but also read the exchange between Felsenstein and Sober (1986).

## INPUT FORMAT

The input for Mix is the standard input for discrete characters
programs, described above in the documentation file for the
discrete-characters programs. States "?", "P", and "B" are allowed.

The options are selected using a menu:

|  |
| --- |
| ``` Mixed parsimony algorithm, version 3.6  Settings for this run:   U                 Search for best tree?  Yes   X                     Use Mixed method?  No   P                     Parsimony method?  Wagner   J     Randomize input order of species?  No. Use input order   O                        Outgroup root?  No, use as outgroup species  1   T              Use Threshold parsimony?  No, use ordinary parsimony   A   Use ancestral states in input file?  No   W                       Sites weighted?  No   M           Analyze multiple data sets?  No   0   Terminal type (IBM PC, ANSI, none)?  ANSI   1    Print out the data at start of run  No   2  Print indications of progress of run  Yes   3                        Print out tree  Yes   4     Print out steps in each character  No   5     Print states at all nodes of tree  No   6       Write out trees onto tree file?  Yes  Are these settings correct? (type Y or the letter for one to change) ``` |

The options U, X, J, O, T, A, and M are the usual User Tree, miXed
methods, Jumble, Outgroup,
Ancestral States, and Multiple Data Sets options, described either
in the main documentation file or in the Discrete Characters Programs
documentation file. The
user-defined trees supplied if you use the U option must be given as rooted
trees with two-way splits (bifurcations). The O option is acted upon only if
the final tree is unrooted and is not a user-defined tree. One of the
important uses of the the O option is to root the tree so that if there are
any characters in which the ancestral states have not been specified, the
program will print out a table showing which ancestral states require the
fewest steps. Note that when any of the characters has Camin-Sokal parsimony
assumed for it, the tree is rooted and the O option will have no effect.

The option P toggles between the Camin-Sokal parsimony criterion
and the default Wagner parsimony criterion. Option X invokes
mixed-method parsimony. If the A option is invoked, the ancestor is not
to be counted as one of the species.

The F (Factors)
option is not available in this program, as it would have no effect on
the result even if that information were provided in the input file.

## OUTPUT FORMAT

Output is standard: a list of equally parsimonious trees, which will be printed
as rooted or unrooted depending on which is appropriate, and, if the
user chooses, a table of the
number of changes of state required in each character. If the Wagner option is
in force for a character, it may not be possible to unambiguously locate the
places on the tree where the changes occur, as there may be multiple
possibilities. If the user selects menu option 5, a table is printed out
after each tree, showing for each
branch whether there are known to be changes in the branch, and what the states
are inferred to have been at the top end of the branch. If the inferred state
is a "?" there will be multiple equally-parsimonious assignments of states; the
user must work these out for themselves by hand.

If the Camin-Sokal parsimony method
is invoked and the Ancestors option is also used, then the program will
infer, for any character whose ancestral state is unknown ("?") whether the
ancestral state 0 or 1 will give the fewest state changes. If these are
tied, then it may not be possible for the program to infer the
state in the internal nodes, and these will all be printed as ".". If this
has happened and you want to know more about the states at the internal
nodes, you will find helpful to use Move to display the tree and examine
its interior states, as the algorithm in Move shows all that can be known
in this case about the interior states, including where there is and is not
amibiguity. The algorithm in Mix gives up more easily on displaying these
states.

If the A option is not used, then the program will assume 0 as the
ancestral state for those characters following the Camin-Sokal method,
and will assume that the ancestral state is unknown for those characters
following Wagner parsimony. If any characters have unknown ancestral
states, and if the resulting tree is rooted (even by outgroup),
a table will also be printed out
showing the best guesses of which are the ancestral states in each
character. You will find it useful to understand the difference between
the Camin-Sokal parsimony criterion with unknown ancestral state and the Wagner
parsimony criterion.

If the U (User Tree) option is used and more than one tree is supplied, the
program also performs a statistical test of each of these trees against the
best tree. This test, which is a version of the test proposed by
Alan Templeton (1983) and evaluated in a test case by me (1985a). It is
closely parallel to a test using log likelihood differences
invented by Kishino and Hasegawa (1989), and uses the mean and variance of
step differences between trees, taken across characters. If the mean
is more than 1.96 standard deviations different then the trees are declared
significantly different. The program
prints out a table of the steps for each tree, the differences of
each from the highest one, the variance of that quantity as determined by
the step differences at individual sites, and a conclusion as to
whether that tree is or is not significantly worse than the best one. It
is important to understand that the test assumes that all the binary
characters are evolving independently, which is unlikely to be true for
many suites of morphological characters.

If the U (User Tree) option is used and more than one tree is supplied, the
program also performs a statistical test of each of these trees against the
best tree. This test, which is a version of the test proposed by
Alan Templeton (1983) and evaluated in a test case by me (1985a). It is
closely parallel to a test using log likelihood differences
invented by Kishino and Hasegawa (1989), and uses the mean and variance of
step differences between trees, taken across characters. If the mean
is more than 1.96 standard deviations different then the trees are declared
significantly different. The program
prints out a table of the steps for each tree, the differences of
each from the highest one, the variance of that quantity as determined by
the step differences at individual characters, and a conclusion as to
whether that tree is or is not significantly worse than the best one. It
is important to understand that the test assumes that all the binary
characters are evolving independently, which is unlikely to be true for
many suites of morphological characters.

If there are more than two trees, the test done is an extension of
the KHT test, due to Shimodaira and Hasegawa (1999). They pointed out
that a correction for the number of trees was necessary, and they
introduced a resampling method to make this correction. In the version
used here the variances and covariances of the sums of steps across
characters are computed for all pairs of trees. To test whether the
difference between each tree and the best one is larger than could have
been expected if they all had the same expected number of steps,
numbers of steps for all trees are sampled with these covariances and equal
means (Shimodaira and Hasegawa's "least favorable hypothesis"),
and a P value is computed from the fraction of times the difference between
the tree's value and the lowest number of steps exceeds that actually
observed. Note that this sampling needs random numbers, and so the
program will prompt the user for a random number seed if one has not
already been supplied. With the two-tree KHT test no random numbers
are used.

In either the KHT or the SH test the program
prints out a table of the number of steps for each tree, the differences of
each from the lowest one, the variance of that quantity as determined by
the differences of the numbers of steps at individual characters,
and a conclusion as to
whether that tree is or is not significantly worse than the best one.

If option 6 is left in its default state the trees
found will be written to a tree file, so that they are available to be used
in other programs. If the program finds multiple
trees tied for best, all of these are written out onto the output tree
file. Each is followed by a numerical weight in square brackets (such as
[0.25000]). This is needed when we use the trees to make a consensus
tree of the results of bootstrapping or jackknifing, to avoid overrepresenting
replicates that find many tied trees.

At the beginning of the program is a constant, maxtrees,
the maximum number of trees which the program will store for output.

The program is descended from earlier programs Sokal and Wagner which have
long since been removed from the PHYLIP package, since Mix has all their
capabilites and more.

---

### TEST DATA SET

|  |
| --- |
| ```      5    6 Alpha     110110 Beta      110000 Gamma     100110 Delta     001001 Epsilon   001110 ``` |

---

### TEST SET OUTPUT (with all numerical options on)

|  |
| --- |
| ``` Mixed parsimony algorithm, version 3.66  5 species, 6 characters  Wagner parsimony method   Name         Characters ----         ----------  Alpha        11011 0 Beta         11000 0 Gamma        10011 0 Delta        00100 1 Epsilon      00111 0        4 trees in all found              +--Epsilon         +-----4        !     +--Gamma        +--2     !  !     +--Delta      --1  +-----3     !        +--Beta         !     +-----------Alpha         remember: this is an unrooted tree!   requires a total of      9.000  steps in each character:          0   1   2   3   4   5   6   7   8   9      *-----------------------------------------     0!       2   2   2   1   1   1              From    To     Any Steps?    State at upper node                              ( . means same as in the node below it on tree)            1                1?011 0   1       2         no     ..... .   2       4        maybe   .0... .   4    Epsilon      yes    0.1.. .   4    Gamma        no     ..... .   2       3         yes    ...00 .   3    Delta        yes    001.. 1   3    Beta        maybe   .1... .   1    Alpha       maybe   .1... .        +--------Gamma           !     +--2     +--Epsilon      !  !  +--4     !  +--3  +--Delta      --1     !     !     +-----Beta         !     +-----------Alpha         remember: this is an unrooted tree!   requires a total of      9.000  steps in each character:          0   1   2   3   4   5   6   7   8   9      *-----------------------------------------     0!       1   2   1   2   2   1              From    To     Any Steps?    State at upper node                              ( . means same as in the node below it on tree)            1                1?011 0   1       2         no     ..... .   2    Gamma       maybe   .0... .   2       3        maybe   ...?? .   3       4         yes    001.. .   4    Epsilon     maybe   ...11 .   4    Delta        yes    ...00 1   3    Beta        maybe   .1.00 .   1    Alpha       maybe   .1... .        +--------Epsilon      +--4     !  !  +-----Gamma        !  +--2   --1     !  +--Delta        !     +--3     !        +--Beta         !     +-----------Alpha         remember: this is an unrooted tree!   requires a total of      9.000  steps in each character:          0   1   2   3   4   5   6   7   8   9      *-----------------------------------------     0!       2   2   2   1   1   1              From    To     Any Steps?    State at upper node                              ( . means same as in the node below it on tree)            1                1?011 0   1       4        maybe   .0... .   4    Epsilon      yes    0.1.. .   4       2         no     ..... .   2    Gamma        no     ..... .   2       3         yes    ...00 .   3    Delta        yes    0.1.. 1   3    Beta         yes    .1... .   1    Alpha       maybe   .1... .        +--------Gamma        +--2     !  !  +-----Epsilon      !  +--4   --1     !  +--Delta        !     +--3     !        +--Beta         !     +-----------Alpha         remember: this is an unrooted tree!   requires a total of      9.000  steps in each character:          0   1   2   3   4   5   6   7   8   9      *-----------------------------------------     0!       2   2   2   1   1   1              From    To     Any Steps?    State at upper node                              ( . means same as in the node below it on tree)            1                1?011 0   1       2        maybe   .0... .   2    Gamma        no     ..... .   2       4        maybe   ?.?.. .   4    Epsilon     maybe   0.1.. .   4       3         yes    ...00 .   3    Delta        yes    0.1.. 1   3    Beta         yes    110.. .   1    Alpha       maybe   .1... . ``` |
