# Supplementary material for: Probabilistic Phylogenetic Inference with Insertions and Deletions
Source: PLoS Comput Biol. 2008 Sep 19;4(9):e1000172. doi: 10.1371/journal.pcbi.1000172 (PMC2527138; doi:10.1371/journal.pcbi.1000172)
Supplement: Dataset S1 — Supplemental Material (24.89 MB GZ) [file pcbi.1000172.s001.gz › erate-supplement-R2/src/phylip3.66-erate/doc/contrast.html]

Contrast


version 3.66

# Contrast -- Computes contrasts for comparative method

```

```

© Copyright 1991-2006 by the University of
Washington. Written by Joseph Felsenstein. Permission is granted to copy
this document provided that no fee is charged for it and that this copyright
notice is not removed.

This program implements the contrasts calculation described in my 1985
paper on the comparative method (Felsenstein, 1985d). It reads in a
data set of the standard quantitative characters sort, and also a
tree from the treefile. It then forms the contrasts between species
that, according to that tree, are statistically independent. This is
done for each character. The contrasts are all standardized by
branch lengths (actually, square roots of branch lengths).

The method is explained in the 1985 paper. It assumes
a Brownian motion model. This model was introduced by Edwards and
Cavalli-Sforza (1964; Cavalli-Sforza and Edwards, 1967)
as an approximation to the evolution of gene frequencies. I have
discussed (Felsenstein, 1973b, 1981c, 1985d, 1988b) the difficulties
inherent in using it as a model for the evolution of quantitative
characters. Chief among these is that the characters do not necessarily evolve
independently or at equal rates. This program allows one to evaluate this,
if there is independent information on the phylogeny. You can
compute the variance of the contrasts for each character, as a measure of
the variance accumulating per unit branch length. You can also test
covariances of characters.

The input file is as described in the continuous characters
documentation file above, for the case of continuous quantitative
characters (not gene frequencies). Options are selected using a menu:

|  |
| --- |
| ``` Continuous character comparative analysis, version 3.6  Settings for this run:   W        Within-population variation in data?  No, species values are means   R     Print out correlations and regressions?  Yes   C                        Print out contrasts?  No   M                     Analyze multiple trees?  No   0         Terminal type (IBM PC, ANSI, none)?  ANSI   1          Print out the data at start of run  No   2        Print indications of progress of run  Yes    Y to accept these or type the letter for one to change ``` |

Option W makes the program expect not means of the phenotypes in each
species, but phenotypes of individual specimens. The details of
the input file format in that case are given below. In that case the
program estimates the covariances of the phenotypic change, as well as
covariances of within-species phenotypic variation. The model used is
similar to (but not identical to) that of Lynch (1990). The
algorithms used differ from the ones he
gives in that paper. They will be described in a forthcoming paper by
me. In the case that has within-species samples contrasts are used by
the program, but it does not make sense to write them out to an
output file for direct analysis. They are of two kinds, contrasts
within species and contrasts between species. The former are
affected only by the within-species phenotypic covariation, but the
latter are affected by both within- and between-species covariation.
Contrast infers these two kinds of covariances and writes the
estimates out.

M is similar to the usual multiple data sets input option, but is used here
to allow multiple trees to be read from the treefile, not multiple
data sets to be read from the input file. In this way you can
use bootstrapping on the data that estimated these trees, get
multiple bootstrap estimates of the tree, and then use the M
option to make multiple analyses of the contrasts and the
covariances, correlations, and regressions. In this way (Felsenstein,
1988b) you can assess the effect of the inaccuracy of the trees on
your estimates of these statistics.

R allows you to turn off or on the printing out of the statistics.
If it is off only the contrasts will be printed out (unless option
1 is selected). With only the contrasts printed out, they are in
a simple array that is in a form that many statistics packages should
be able to read. The contrasts are rows, and each row has one contrast
for each character. Any multivariate statistics package should be able
to analyze these (but keep in mind that the contrasts have, by virtue
of the way they are generated, expectation zero, so all regressions
must pass through the origin). If the W option has been set to
analyze within-species as well as between-species variation, the R
option does not appear in the menu as the regression and correlation
statistics should always be computed in that case.

As usual, the tree file has the default name intree. It
should contain the desired tree or trees. These can be
either in bifurcating form, or may have the bottommost fork be a
trifurcation (it should not matter which of these ways you present the tree).
The tree must, of course, have branch lengths. These cannot be negative.
Trees from some distance methods, particularly Neighbor-Joining, are
sometimes inferred to have negative branch lengths, so be sure to
choose options in those programs that prevent negative branch lengths.

If you have a molecular data set (for example) and also, on the same
species, quantitative measurements, here is how you can allow for the
uncertainty of your estimate of the tree. Use Seqboot to generate multiple
data sets from your molecular data. Then, whichever method you use to
analyze it (the relevant ones are those that produce estimates of the
branch lengths: Dnaml, Dnamlk, Fitch, Kitsch, and Neighbor -- the latter
three require you to use Dnadist to turn the bootstrap data sets into
multiple distance matrices), you should use the Multiple Data Sets
option of that program. This will result in a tree file with many
trees on it. Then use this tree file with the input file containing
your continuous quantitative characters, choosing the Multiple Trees
(M) option. You will get one set of contrasts and statistics for each
tree in the tree file. At the moment there is no overall summary:
you will have to tabulate these by hand. A similar process can be
followed if you have restriction sites data (using Restml) or
gene frequencies data.

The statistics that are printed out include the covariances between
all pairs of characters, the regressions of each character on each
other (column j is regressed on row i), and the correlations between
all pairs of characters. In assessing degress of freedom it is
important to realize that each contrast was taken to have
expectation zero, which is known because each contrast could as
easily have been computed xi-xj instead of xj-xi. Thus there is no
loss of a degree of freedom for estimation of a mean. The degrees
of freedom is thus the same as the number of contrasts, namely one
less than the number of species (tips). If you feed these contrasts
into a multivariate statistics program make sure that it knows that
each variable has expectation exactly zero.

## Within-species variation

With the W option selected, Contrast analyzes data sets with variation within
species, using a model like that proposed by Michael Lynch (1990).
The method is described in vague terms in my book (Felsenstein, 2004, pp. 441).
If you select the W option for within-species variation, the data
set should have this structure (on the left are the data, on the right
my comments:

|  |  |
| --- | --- |
| ```    10    5               Alpha        2            2.01 5.3 1.5  -3.41 0.3  1.98 4.3 2.1  -2.98 0.45 Gammarus     3  6.57 3.1 2.0  -1.89 0.6  7.62 3.4 1.9  -2.01 0.7  6.02 3.0 1.9  -2.03 0.6 ... ``` | ```    number of species, number of characters    name of 1st species, # of individuals    data for individual #1    data for individual #2    name of 2nd species, # of individuals    data for individual #1    data for individual #2    data for individual #3    (and so on) ``` |

The covariances, correlations, and regressions for the "additive"
(between-species evolutionary variation) and "environmental" (within-species
phenotypic variation) are
printed out (the maximum likelihood estimates of each).
The program also estimates the within-species phenotypic variation in the
case where the between-species evolutionary covariances are forced to be
zero. The log-likelihoods of these two cases are compared and a
likelihood ratio test (LRT) is carried out. The program prints the result
of this test as a chi-square variate, and gives the number of degrees of
freedom of the LRT. You have to look up the chi-square variable on a
table of the chi-square distribution. The A option is available (if
the W option is invoked) to allow you to turn off the doing of this test
if you want to.

The log-likelihood of the data under the models with and without
between-species For the moment the program cannot handle the case where
within-species variation is to be taken into account but where only species
means are available. (It can handle cases where some species have only one
member in their sample).

We hope to fix this soon. We are also on our way to
incorporating full-sib, half-sib, or clonal groups within species, so as
to do one analysis for within-species genetic and between-species
phylogenetic variation.

The data set used as an example below is the example from a
paper by Michael Lynch (1990), his characters having been log-transformed.
In the case where there is only one specimen per species, Lynch's model
is identical to our model of within-species variation (for
multiple individuals per species it is not a subcase of his model).

---

### TEST SET INPUT

|  |
| --- |
| ```     5   2 Homo        4.09434  4.74493 Pongo       3.61092  3.33220 Macaca      2.37024  3.36730 Ateles      2.02815  2.89037 Galago     -1.46968  2.30259 ``` |

---

### TEST SET INPUT TREEFILE

|  |
| --- |
| ``` ((((Homo:0.21,Pongo:0.21):0.28,Macaca:0.49):0.13,Ateles:0.62):0.38,Galago:1.00); ``` |

---

### TEST SET OUTPUT (with all numerical options and option C on )

|  |
| --- |
| ``` Continuous character contrasts analysis, version 3.66     5 Populations,    2 Characters  Name                       Phenotypes ----                       ----------  Homo         4.09434   4.74493 Pongo        3.61092   3.33220 Macaca       2.37024   3.36730 Ateles       2.02815   2.89037 Galago      -1.46968   2.30259   Contrasts (columns are different characters) --------- -------- --- --------- -----------     0.74593   2.17989    1.58474   0.71761    1.19293   0.86790    3.35832   0.89706  Covariance matrix ---------- ------      3.9423    1.7028     1.7028    1.7062  Regressions (columns on rows) ----------- -------- -- -----      1.0000    0.4319     0.9980    1.0000  Correlations ------------      1.0000    0.6566     0.6566    1.0000 ``` |
