# Supplementary material for: Probabilistic Phylogenetic Inference with Insertions and Deletions
Source: PLoS Comput Biol. 2008 Sep 19;4(9):e1000172. doi: 10.1371/journal.pcbi.1000172 (PMC2527138; doi:10.1371/journal.pcbi.1000172)
Supplement: Dataset S1 — Supplemental Material (24.89 MB GZ) [file pcbi.1000172.s001.gz › erate-supplement-R2/src/phylip3.66-erate/doc/contchar.html]

contchar


version 3.66

# Gene Frequencies and Continuous Character Data Programs

© Copyright 1986-2006 by the University of
Washington. Written by Joseph Felsenstein. Permission is granted to copy
this document provided that no fee is charged for it and that this copyright
notice is not removed.

The programs in this group
use gene frequencies and quantitative character values. One (Contml)
constructs maximum likelihood estimates of the phylogeny, another
(Gendist) computes genetic distances for use in the distance matrix
programs, and the third (Contrast) examines correlation of traits as
they evolve along a given phylogeny.

When the gene frequencies data are used in Contml or Gendist, this
involves the following assumptions:

1. Different lineages evolve independently.- After two lineages split, their characters change
     independently.- Each gene frequency changes by genetic drift, with or without mutation
       (this varies from method to method).- Different loci or characters drift independently.

How these assumptions affect the methods will be seen in my papers on
inference of phylogenies from gene frequency and continuous character
data (Felsenstein, 1973b, 1981c, 1985c).

The input formats are fairly similar to the discrete-character
programs, but with one difference. When Contml is used in the gene-frequency
mode (its usual, default mode), or when Gendist is used,
the first line contains the number of species (or
populations) and the number of loci and the options information.
There then follows a line which
gives the numbers of alleles at each locus, in order. This must be
the full number of alleles, not the number of alleles which will be input:
i. e. for a two-allele locus the number should be 2, not 1. There
then follow the species (population) data, each species beginning
on a new line. The first 10 characters are taken as the name, and
thereafter the values of the individual characters are read free-format,
preceded and separated by blanks. They can go to a new line if desired,
though of course not in the middle of a number. Missing data is not
allowed - an important limitation. In the default configuration, for
each locus, the numbers should be
the frequencies of all but one allele. The menu option A (All) signals that
the frequencies of all alleles are provided in the input data -- the
program will then automatically ignore the last of them. So without the
A option, for a
three-allele locus there should be two numbers, the frequencies of
two of the alleles (and of course it must always be the same
two!). Here is a typical data set without the A option:

|  |
| --- |
| ```      5    3 2 3 2 Alpha      0.90 0.80 0.10 0.56 Beta       0.72 0.54 0.30 0.20 Gamma      0.38 0.10 0.05  0.98 Delta      0.42 0.40 0.43 0.97 Epsilon    0.10 0.30 0.70 0.62 ``` |

whereas here is what it would have to look like if the A option were
invoked:

|  |
| --- |
| ```      5    3 2 3 2 Alpha      0.90 0.10 0.80 0.10 0.10 0.56 0.44 Beta       0.72 0.28 0.54 0.30 0.16 0.20 0.80 Gamma      0.38 0.62 0.10 0.05 0.85  0.98 0.02 Delta      0.42 0.58 0.40 0.43 0.17 0.97 0.03 Epsilon    0.10 0.90 0.30 0.70 0.00 0.62 0.38 ``` |

The first line has the number of species (or populations) and the number
of loci. The second line has the number of alleles for each of the 3 loci.
The species lines have names (filled out to 10 characters with blanks)
followed by the gene frequencies of the 2 alleles for the first locus, the
3 alleles for the second locus, and the 2 alleles for the third locus.
You can start a new line after any of these allele frequencies, and
continue to give the frequencies on that line (without repeating the
species name).

If all alleles of a locus are given, it is important to have them add up
to 1. Roundoff of the frequencies may cause the program to conclude that
the numbers do not sum to 1, and stop with an error message.

While many compilers may be more tolerant, it is probably wise to
make sure that each number, including the first, is preceded by a blank,
and that there are digits both preceding and following any decimal
points.

Contml and Contrast also treat quantitative characters (the
continuous-characters mode in Contml, which is option C). It is assumed
that each character is evolving according to a Brownian motion model, at the
same rate, and independently. In
reality it is almost always impossible to guarantee this. The issue is
discussed at length
in my review article in Annual Review of Ecology and Systematics (Felsenstein,
1988a), where I point out the difficulty of transforming the characters so
that they are not only genetically independent but have independent selection
acting on them. If you are going to use Contml to model evolution of
continuous characters, then you should at least make some attempt to remove
genetic correlations between the characters (usually all one can do is remove
phenotypic correlations by transforming the characters so that there is no
within-population covariance and so that the within-population
variances of the characters are equal -- this is equivalent to using
Canonical Variates). However, this will only guarantee that one has
removed phenotypic covariances between characters. Genetic covariances
could only be removed by knowing the coheritabilities of the characters,
which would require genetic experiments, and selective covariances
(covariances due to covariation of selection pressures) would require
knowledge of the sources and extent of selection pressure in all variables.

Contrast is a program designed to infer, for a given phylogeny that is
provided to the program, the covariation between characters in a data
set. Thus we have a program in this set that allow us to take information
about the covariation and rates of evolution of characters and make an
estimate of the phylogeny (Contml), and a program that takes an estimate of the
phylogeny and infers the variances and covariances of the character
changes. But we have no program that infers both the phylogenies and
the character covariation from the same data set.

In the quantitative characters mode, a typical small data set would be:

|  |
| --- |
| ```      5   6 Alpha      0.345 0.467 1.213  2.2  -1.2 1.0 Beta       0.457 0.444 1.1    1.987 -0.2 2.678 Gamma      0.6 0.12 0.97 2.3  -0.11 1.54 Delta      0.68  0.203 0.888 2.0  1.67 Epsilon    0.297  0.22 0.90 1.9 1.74 ``` |

Note that in the latter case, there is no line giving the numbers
of alleles at each locus. In this latter case no square-root
transformation of the coordinates is done: each is assumed to give
directly the position on the Brownian motion scale.

For further discussion of options and modifiable constants in Contml,
Gendist, and Contrast see the documentation files for those programs.
