# Supplementary material for: Probabilistic Phylogenetic Inference with Insertions and Deletions
Source: PLoS Comput Biol. 2008 Sep 19;4(9):e1000172. doi: 10.1371/journal.pcbi.1000172 (PMC2527138; doi:10.1371/journal.pcbi.1000172)
Supplement: Dataset S1 — Supplemental Material (24.89 MB GZ) [file pcbi.1000172.s001.gz › erate-supplement-R2/src/phylip3.66-erate/doc/restdist.html]

restdist


version 3.66

# Restdist -- Program to compute distance matrix from restriction sites or fragments

© Copyright 2000-2006 by the University of
Washington. Written by Joseph Felsenstein. Permission is granted to copy
this document provided that no fee is charged for it and that this copyright
notice is not removed.

Restdist reads the same restriction sites format as Restml and
computes a restriction sites distance. It can also compute a restriction
fragments distance. The original restriction fragments and restriction
sites distance methods were introduced by Nei and Li (1979). Their original
method for restriction fragments is
also available in this program, although its default methods
are my modifications of the original Nei and Li methods.

These two distances assume that the restriction sites are accidental byproducts
of random change of nucleotide sequences. For my restriction sites distance
the DNA sequences are assumed to be changing according to the Kimura
2-parameter model of DNA change (Kimura, 1980). The user can set the
transition/transversion rate for the model. For my restriction fragments
distance there is
there is an implicit assumption of a Jukes-Cantor (1969) model of change,
The user can also set the
parameter of a correction for unequal rates of evolution between sites in
the DNA sequences, using a Gamma distribution of rates among sites.
The Jukes-Cantor model is also implicit in the restriction fragments
distance of Nei and Li(1979). It
does not allow us to correct for a Gamma distribution of rates among
sites.

## Restriction Sites Distance

The restriction sites distances use data coded for the presence of absence of
individual restriction sites (usually as + and - or 0 and 1). My
distance is based on the proportion, out of all sites observed in one species
or the other, which are present in both species. This is done to correct for
the ascertainment of sites, for the fact that we are not aware of many sites
because they do not appear in any species.

My distance starts by computing from the particular pair of species the fraction

```
                 n++
   f =  ---------------------
         n++ + 1/2 (n+- + n-+)
```

where *n++* is the number of sites contained in both species,
*n+-* is the number of sites contained in the first of the
two species but not in the second, and *n-+* is the
number of sites contained in the second of the two species but not in the
first. This is the fraction of sites that are present in one species which are
present in both. Since the number of sites present in the two species will
often differ, the denominator is the average of the number of sites found in
the two species.

If each restriction site is *s* nucleotides long, the probability
that a restriction site is present in the other species, given that it is
present in a species, is

```
      Qs,
```

where *Q* is the probability that a nucleotide has no net change as one
goes from the one species to the other. It may have changed in between; we
are interested in the probability that that nucleotide site is in the same base
in both species, irrespective of what has happened in between.

The distance is then computed by finding the branch length of a two-species
tree (connecting these two species with a single branch) such
that *Q* equals the *s*-th root of *f*. For this the
program computes *Q* for various values of branch length, iterating them
by a Newton-Raphson algorithm until the two quantities are equal.

The resulting distance should be numerically close to the original
restriction sites distance of Nei and Li (1979) when divergence is small.
Theirs computes the probability of retention of a site in a way that assumes
that the site is present in the common ancestor of the two species. Ours
does not make this assumption. It is inspired by theirs,
but differs in this detail. Their distance also assumes a Jukes-Cantor (1969)
model of base change, and does not allow for transitions being more frequent
than transversions. In this sense mine generalizes theres somewhat.
Their distance does include, as mine does as well, a correction for
Gamma distribution of rate of change among nucleotide sites.

I have made their original distance available here

## Restriction Fragments Distance

For restriction fragments data we use a different distance. If we
average over all restriction fragment lengths, each at its own
expected frequency, the probability that the fragment will still be
in existence after a certain amount of branch length, we must take into
account the probability that the two restriction sites at the ends of
the fragment do not mutate, and the probability that no new
restriction site occurs within the fragment in that amount of branch
length. The result for a restriction site length of *s* is:

```
                Q2s
          f = --------
               2 - Qs
```

(The details of the derivation are given in my book
*Inferring Phylogenies* (Felsenstein, 2004).
Given the observed fraction of restriction sites retained, *f*,
we can solve a quadratic equation from the above expression for
*Qs*. That makes it easy to obtain a value of *Q*,
and the branch length can then be estimated by adjusting it so the
probability of a base not changing is equal to that value.

Alternatively, if we use the Nei and Li (1979) restriction fragments
distance, this involves solving for *g* in the nonlinear
equation

```
       g  =  [ f (3 - 2g) ]1/4
```

and then the distance is given by

```
       d  =  - (2/r) loge(g)
```

where *r* is the length of the restriction site.

Comparing these two restriction fragments distances in a case
where their underlying DNA model is the same (which is when the
transition/transversion ratio of the modified model is set to
0.5), you will find
that they are very close to each other, differing very little at
small distances, with the modified distance become smaller than
the Nei/Li distance at larger distances. It will therefore matter
very little which one you use.

## A Comment About RAPDs and AFLPs

Although these distances are designed for restriction sites
and restriction fragments data, they can be applied to RAPD and
AFLP data as well. RAPD (Randomly Amplified Polymorphic DNA)
and AFLP (Amplified Fragment Length Polymorphism) data consist
of presence or absence of individual bands on a gel. The bands
are segments of DNA with PCR primers at each end. These
primers are defined sequences of known length (often about
10 nucleotides each). For AFLPs the reolevant length is the primer
length, plus three nucleotides. Mutation in these sequences makes them no
longer be primers, just as in the case of restriction sites.
Thus a pair of 10-nucleotide primers will behave much the same
as a 20-nucleotide restriction site, for RAPDs (26 for AFLPs). You can use
the restriction sites distance as the distance between RAPD or AFLP patterns
if you set the proper value for the total length of the site to the
total length of the primers (plus 6 in the case of AFLPs).
Of course there are many possible sources of noise in these data,
including confusing fragments of similar length for each other
and having primers near each other in the genome, and these are
not taken into account in the statistical model used here.

## INPUT FORMAT AND OPTIONS

The input is fairly standard, with one addition. As usual the first line of
the file gives the number of species and the number of sites, but there is also
a third number, which is the number of different restriction enzymes that were
used to detect the restriction sites. Thus a data set with 10 species and
35 different sites, representing digestion with 4 different enzymes, would
have the first line of the data file look like this:

```
   10   35    4
```

The site data are in standard form. Each species starts with a species name
whose maximum length is given by the constant "nmlngth"
(whose value in the
program as distributed is 10 characters). The name should, as usual, be padded
out to that length with blanks if necessary. The sites data then follows, one
character per site (any blanks will
be skipped and ignored). Like the DNA and protein sequence data, the
restriction sites data may be either in the "interleaved" form or the
"sequential" form. Note that if you are analyzing restriction sites
data with the programs Dollop or Mix or other discrete character
programs, at the moment those programs do not use the "aligned" or
"interleaved" data format. Therefore you may want to avoid that format
when you have restriction sites data that you will want to feed into
those programs.

The presence of a site is indicated by a "+" and the absence by a "-". I have
also allowed the use of "1" and "0" as synonyms for "+" and "-", for
compatibility with Mix and Dollop which do not allow "+" and "-". If the
presence of
the site is unknown (for example, if the DNA containing it has been deleted so
that one
does not know whether it would have contained the site) then the state "?" can
be used to indicate that the state of this site is unknown.

The options are selected using an interactive menu. The menu looks like this:

|  |
| --- |
| ``` Restriction site or fragment distances, version 3.6  Settings for this run:   R           Restriction sites or fragments?  Sites   N        Original or modified Nei/Li model?  Modified   G  Gamma distribution of rates among sites?  No   T            Transition/transversion ratio?  2.000000   S                              Site length?  6.0   L                  Form of distance matrix?  Square   M               Analyze multiple data sets?  No   I              Input sequences interleaved?  Yes   0       Terminal type (IBM PC, ANSI, none)?  ANSI   1       Print out the data at start of run?  No   2     Print indications of progress of run?  Yes    Y to accept these or type the letter for one to change ``` |

The user either types "Y" (followed, of course, by a carriage-return)
if the settings shown are to be accepted, or the letter or digit corresponding
to an option that is to be changed.

The R option toggles between a restriction sites distance, which
is the default setting, and a restriction fragments distance. In
both cases, another option appears, the N (Nei/Li) option.
This allows the user to choose the original Nei and Li (1979)
restriction sites or fragments distances rather than my modified versions,
which are the defaults.

If the G (Gamma distribution) option is selected, the user will be
asked to supply the coefficient of variation of the rate of substitution
among sites. This is different from the parameters used by Nei and Jin, who
introduced Gamma distribution of rates in DNA distances, but
related to their parameters: their parameter *a* is also known as
"alpha", the shape parameter of the Gamma distribution. It is
related to the coefficient of variation by

     CV = 1 / a1/2

or

     a = 1 / (CV)2

(their parameter *b* is absorbed here by the requirement that time is scaled so
that the mean rate of evolution is 1 per unit time, which means that *a = b*).
As we consider cases in which the rates are less variable we should set *a*
larger and larger, as *CV* gets smaller and smaller.

The Gamma distribution option is not available when using the
original Nei/Li restriction fragments distance.

The T option is the Transition/transversion option. The user is prompted for
a real number greater than 0.0, as the expected ratio of transitions to
transversions. Note
that this is the resulting expected ratio of transitions to transversions.
The default value of the T parameter if you do not use the T
option is 2.0. The T option is not available when you choose the original
Nei/Li restriction fragment distance, which assumes a Jukes-Cantor (1969)
model of DNA change, for which the transition/transversion ratio is
in effect fixed at 0.5.

The S option selects the site length. This is set to a default
value of 6. It can be set to any positive integer. While in
the Restml program there is an upper limit on the restriction
site length (set by memory limitations), in Restdist there is
no effective limit on the size of the restriction sites. A value
of 20, which might be appropriate in many cases for RAPD or AFLP
data, is typically not practical in Restml, but it is useable in
Restdist.

Option L specifies that the output file will have a square matrix
of distances. It can be used to change to lower-triangular
data matrices. This will usually not be
necessary, but if the distance matrices are going to be very
large, this alternative can reduce their size by half. The
programs which are to use them should then of course be informed
that they can expect lower-triangular distance matrices.

The M, I, and 0 options are the usual Multiple data set,
Interleaved input, and screen terminal type options. These are
described in the main documentation file.

Option 1 specifies that the input data will be written out
on the output file before the distances. This is off by
default. If it is done, it will make the output file unusable
as input to our distance matrix programs.

Option 2 turns off or on the indications of the progress of the run.
The program prints out a row of dots (".") indicating the
calculation of individual distances. Since the distance matrix
is symmetrical, the program only computes the distances for the
upper triangle of the distance matrix, and then duplicates
the distance to the other corner of the matrix. Thus the rows of
dots start out of full length, and then egt shorter and shorter.

## OUTPUT FORMAT

The output file contains on its first line the number of species. The
distance matrix is then printed in standard
form, with each species starting on a new line with the species name, followed
by the distances to the species in order. These continue onto a new line
after every nine distances. If the L option is used, the matrix or distances
is in lower triangular form, so that only the distances to the other species
that precede each species are printed. Otherwise the distance matrix is square
with zero distances on the diagonal. In general the format of the distance
matrix is such that it can serve as input to any of the distance matrix
programs.

If the option to print out the data is selected, the output file will
precede the data by more complete information on the input and the menu
selections. The output file begins by giving the number of species and the
number of characters.

The distances printed out are scaled in terms of expected numbers of
substitutions per DNA site, counting both transitions and transversions but not
replacements of a base by itself, and scaled so that the average rate of
change, averaged over all sites analyzed, is set to 1.0. Thus when the
G option is used, the rate of change at one site may be higher than
at another, but their mean is expected to be 1.

## PROGRAM CONSTANTS

The constants available to be changed are "initialv" and
"iterationsr". The constant "initialv" is the starting
value of the distance in the iterations. This will typically not need to
be changed. The constant "iterationsr" is the number of
times that the Newton-Raphson method which is used to solve the
equations for the distances is iterated. The program can be
speeded up by reducing the number of iterations from the default
value of 20, but at the possible risk of computing the distance
less accurately.

## FUTURE OF THE PROGRAM

The present program does not compute the original distance of Nei and Li (1979)
for restriction sites (though it does have an option to compute their original
distance for restriction fragments). I hope to add their restriction
sites distance in the near future.

---

### TEST DATA SET

|  |
| --- |
| ```    5   13   2 Alpha     ++-+-++--+++- Beta      ++++--+--+++- Gamma     -+--+-++-+-++ Delta     ++-+----++--- Epsilon   ++++----++--- ``` |

---

### CONTENTS OF OUTPUT FILE (with all numerical options on)

(Note that when the options for displaying the input data are turned off,
the output is in a form suitable for use as an input file in the distance
matrix programs).

|  |
| --- |
| ```     5 Species,   13 Sites  Name            Sites ----            -----  Alpha        ++-+-++--+ ++- Beta         ++++--+--+ ++- Gamma        -+--+-++-+ -++ Delta        ++-+----++ --- Epsilon      ++++----++ ---   Alpha       0.000000  0.022368  0.107681  0.082634  0.095581 Beta        0.022368  0.000000  0.107681  0.082634  0.056895 Gamma       0.107681  0.107681  0.000000  0.192466  0.207319 Delta       0.082634  0.082634  0.192466  0.000000  0.015949 Epsilon     0.095581  0.056895  0.207319  0.015949  0.000000 ``` |
