# Supplementary material for: Probabilistic Phylogenetic Inference with Insertions and Deletions
Source: PLoS Comput Biol. 2008 Sep 19;4(9):e1000172. doi: 10.1371/journal.pcbi.1000172 (PMC2527138; doi:10.1371/journal.pcbi.1000172)
Supplement: Dataset S1 — Supplemental Material (24.89 MB GZ) [file pcbi.1000172.s001.gz › erate-supplement-R2/src/phylip3.66-erate/doc/dnainvar.html]

dnainvar


version 3.66

# Dnainvar -- Program to compute Lake's and Cavender's phylogenetic invariants from nucleotide sequences

© Copyright 1986-2006 by the University of Washington.
Written by Joseph Felsenstein. Permission is granted to copy
this document provided that no fee is charged for it and that this copyright
notice is not removed.

This program reads in nucleotide sequences for four species and computes
the phylogenetic invariants discovered by James Cavender (Cavender and
Felsenstein, 1987) and James Lake (1987). Lake's method is also
called by him "evolutionary parsimony". I prefer Cavender's more
mathematically precise term "invariants", as the method bears somewhat
more relationship to likelihood methods than to parsimony. The invariants
are mathematical
formulas (in the present case linear or quadratic) in the EXPECTED
frequencies of site patterns which are zero for all trees of a given
tree topology, irrespective of branch lengths. We can consider at a given
site that if there
are no ambiguities, we could have for four species the nucleotide patterns
(considering the same site across all four species) AAAA, AAAC, AAAG, ...
through TTTT, 256 patterns in all.

The invariants are formulas in the expected pattern frequencies, not
the observed pattern frequencies. When they are computed using the
observed pattern frequencies, we will
usually find that they are not precisely zero even when the model is correct
and we have the correct tree topology. Only as the number of nucleotides
scored becomes infinite will the observed pattern frequencies approach their
expectations; otherwise, we must do a statistical test of the invariants.

Some explanation of invariants will be found in the above papers, and also
in my recent review article on statistical aspects of inferring phylogenies
(Felsenstein, 1988b). Although invariants have some important advantages,
their validity also depends on symmetry assumptions that may not be satisfied.
In the discussion below suppose that the possible unrooted phylogenies are
I: ((A,B),(C,D)), II: ((A,C),(B,D)), and III: ((A,D),(B,C)).

## Lake's Invariants, Their Testing and Assumptions

Lake's invariants are fairly simple to describe: the patterns involved are
only those in which there are two purines and two pyrimidines at a site.
Thus a site with AACT would affect the invariants, but a site with AAGG would
not. Let us use (as Lake does)
the symbols 1, 2, 3, and 4, with the proviso that 1 and 2
are either both of the purines or both of the pyrimidines; 3 and 4 are the
other two nucleotides. Thus 1 and 2 always differ by a transition; so do
3 and 4. Lake's invariants, expressed in terms of expected frequencies, are
the three quantities:

(1)      P(1133) + P(1234) - P(1134) - P(1233),

(2)      P(1313) + P(1324) - P(1314) - P(1323),

(3)      P(1331) + P(1342) - P(1341) - P(1332),

He showed that invariants (2) and (3) are zero under Topology I, (1) and (3)
are zero under topology II, and (1) and (2) are zero under Topology III. If,
for example, we see a site with pattern ACGC, we can start by setting 1=A.
Then 2 must be G. We can then set 3=C (so that 4 is T). Thus its pattern
type, making those substitutions, is 1323. P(1323) is the expected
probability of the type of pattern which includes ACGC, TGAG, GTAT, etc.

Lake's invariants are easily tested with observed frequencies. For example,
the first of them is a test of whether there are as many sites of types 1133
and 1234 as there are of types 1134 and 1233; this is easily tested with a
chi-square test or, as in this program, with an exact binomial test. Note
that with several invariants to test, we risk overestimating the significance
of results if we simply accept the nominal 95% levels of significance
(Li and Guoy, 1990).

Lake's invariants assume that each site is evolving independently, and that
starting from any base a transversion is equally likely to end up at each of
the two possible bases (thus, an A undergoing a transversion is equally likely
to end up as a C or a T, and similarly for the other four bases from which one
could start. Interestingly, Lake's results do not assume that rates of
evolution are the same at all sites. The result that the total of 1133 and 1234
is expected to be the same as the total of 1134 and 1233 is unaffected by the
fact that we may have aggregated the counts over classes of sites evolving at
different rates.

## Cavender's Invariants, Their Testing and Assumptions

Cavender's invariants (Cavender and Felsenstein, 1987) are for the case of
a character with two states. In the nucleic acid case we can classify
nucleotides into two states, R and Y (Purine and Pyrimidine) and then use the
two-state results. Cavender starts, as before, with the pattern frequencies.
Coding purines as R and pyrimidines as Y, the patterns types are RRRR, RRRY,
and so on until YYYY, a total of 16 types. Cavender found quadratic functions
of the expected frequencies of these 16 types that were expected to be zero
under a given phylogeny, irrespective of branch lengths. Two invariants
(called K and L) were found for each tree topology. The L invariants are
particularly easy to understand. If we have the tree topology ((A,B),(C,D)),
then in the case of two symmetric states, the event that A and B have the same
state should be independent of whether C and D have the same state, as the
events determining these happen in different parts of the tree. We can set
up a contingency table:

```
                                 C = D         C =/= D
                           ------------------------------
                          |
                   A = B  |   YYYY, YYRR,     YYYR, YYRY,
                          |   RRRR, RRYY      RRYR, RRRY
                          |
                 A =/= B  |   YRYY, YRRR,     YRYR, YRRY,
                          |   RYYY, RYRR      RYYR, RYRY
```

and we expect that the events C = D and A = B will be independent. Cavender's
L invariant for this tree topology is simply the negative of the crossproduct
difference,

      P(A=/=B and C=D) P(A=B and C=/=D) - P(A=B and C=D) P(A=/=B and C=/=D).

One of these L invariants is defined for each of the three tree topologies.
They can obviously be tested simply by doing a chi-square test on the
contingency table. The one corresponding to the correct topology should be
statistically indistinguishable from zero. Again, there is a possible
multiple tests problem if all three are tested at a nominal value of 95%.

The K invariants are differences between the L invariants. When one of the
tables is expected to have crossproduct difference zero, the other two are
expected to be nonzero, and also to be equal. So the difference of their
crossproduct differences can be taken; this is the K invariant. It is not
so easily tested.

The assumptions of Cavender's invariants are different from those of
Lake's. One obviously need not assume anything about the frequencies of, or
transitions among, the two different purines or the two different
pyrimidines. However one does need to assume independent events at each site,
and one needs to assume that the Y and R states are symmetric, that the
probability per unit time that a Y changes into an R is the same as the
probability that an R changes into a Y, so that we expect equal frequencies
of the two states. There is also an assumption that all sites are changing
between these two states at the same expected rate. This assumption is not
needed for Lake's invariants, since expectations of sums are equal to
sums of expectations, but for Cavender's it is, since products of expectations
are not equal to expectations of products.

It is helpful to have both sorts of invariants available; with further work we
may appreciate what other invaraints there are for various models of nucleic
acid change.

## INPUT FORMAT

The input data for Dnainvar is standard. The first line of the input file
contains the
number of species (which must always be 4 for this version of Dnainvar)
and the number of sites.

Next come the species data. Each
sequence starts on a new line, has a ten-character species name
that must be blank-filled to be of that length, followed immediately
by the species data in the one-letter code. The sequences must either
be in the "interleaved" or "sequential" formats
described in the Molecular Sequence Programs document. The I option
selects between them. The sequences can have internal
blanks in the sequence but there must be no extra blanks at the end of the
terminated line. Note that a blank is not a valid symbol for a deletion.

The options are selected using an interactive menu. The menu looks like this:

|  |
| --- |
| ``` Nucleic acid sequence Invariants method, version 3.6  Settings for this run:   W                       Sites weighted?  No   M           Analyze multiple data sets?  No   I          Input sequences interleaved?  Yes   0   Terminal type (IBM PC, ANSI, none)?  ANSI   1    Print out the data at start of run  No   2  Print indications of progress of run  Yes   3      Print out the counts of patterns  Yes   4              Print out the invariants  Yes    Y to accept these or type the letter for one to change ``` |

The user either types "Y" (followed, of course, by a carriage-return)
if the settings shown are to be accepted, or the letter or digit corresponding
to an option that is to be changed.

The options W, M and 0 are the usual ones. They are described in the
main documentation file of this package. Option I is the same as in
other molecular sequence programs and is described in the documentation file
for the sequence programs.

## OUTPUT FORMAT

The output consists first (if option 1 is selected) of a reprinting of the
input data, then (if option 2 is on) tables
of observed pattern frequencies and pattern type frequencies. A table will
be printed out, in alphabetic order AAAA through TTTT of all the patterns
that appear among the sites and the number of times each appears. This table
will be invaluable for computation of any other invariants. There follows
another table, of pattern types, using the 1234 notation, in numerical
order 1111 through 1234, of the number of times each type of pattern appears.
In this computation all sites at which there are any ambiguities or deletions
are omitted. Cavender's invariants could actually be computed from sites
that have only Y or R ambiguities; this will be done in the next release of
this program.

If option 3 is on the invariants are then printed out,
together with their statistical
tests. For Lake's invariants the two sums which are expected to be equal are
printed out, and then the result of an one-tailed exact binomial test which
tests whether the difference is expected to be this positive or more. The P
level is given (but remember the multiple-tests problem!).

For Cavender's L invariants the contingency tables are given. Each is tested
with a one-tailed chi-square test. It is possible that the expected numbers
in some categories could be too small for valid use of this test; the program
does not check for this. It is also possible that the chi-square could be
significant but in the wrong direction; this is not tested in the current
version of the program. To check it beware of a chi-square greater than 3.841
but with a positive invariant. The invariants themselves are computed, as the
difference of cross-products. Their absolute magnitudes are not important,
but which one is closest to zero may be indicative. Significantly nonzero
invariants should be negative if the model is valid. The K invariants, which
are simply differences among the L invariants, are also printed out without
any test on them being conducted. Note that it is possible to use the
bootstrap utility Seqboot to create multiple data sets, and from the
output from sunning all of these get the empirical variability of these
quadratic invariants.

## PROGRAM CONSTANTS

The constants
that are defined at the beginning of the program include
"maxsp",
which must always be 4 and should not be changed.

The program is very fast, as it has rather little work to do; these methods
are just a little bit beyond the reach of hand tabulation. Execution speed
should never be a limiting factor.

## FUTURE OF THE PROGRAM

In a future version I hope to allow for Y and R codes in the calculation of
the Cavender invariants, and to check for significantly negative cross-product
differences in them, which would indicate violation of the model. By
then there should be more known about invariants for larger number of species,
and any such advances will also be incorporated.

---

### TEST DATA SET | | | --- | | ``` 4 13 Alpha AACGTGGCCAAAT Beta AAGGTCGCCAAAC Gamma CATTTCGTCACAA Delta GGTATTTCGGCCT ``` | --- TEST SET OUTPUT (run with all numerical options turned on) | | | --- | | ``` Nucleic acid sequence Invariants method, version 3.66 4 species, 13 sites Name Sequences ---- --------- Alpha AACGTGGCCA AAT Beta ..G..C.... ..C Gamma C.TT.C.T.. C.A Delta GGTA.TT.GG CC. Pattern Number of times AAAC 1 AAAG 2 AACC 1 AACG 1 CCCG 1 CCTC 1 CGTT 1 GCCT 1 GGGT 1 GGTA 1 TCAT 1 TTTT 1 Symmetrized patterns (1, 2 = the two purines and 3, 4 = the two pyrimidines or 1, 2 = the two pyrimidines and 3, 4 = the two purines) 1111 1 1112 2 1113 3 1121 1 1132 2 1133 1 1231 1 1322 1 1334 1 Tree topologies (unrooted): I: ((Alpha,Beta),(Gamma,Delta)) II: ((Alpha,Gamma),(Beta,Delta)) III: ((Alpha,Delta),(Beta,Gamma)) Lake's linear invariants (these are expected to be zero for the two incorrect tree topologies. This is tested by testing the equality of the two parts of each expression using a one-sided exact binomial test. The null hypothesis is that the first part is no larger than the second.) Tree Exact test P value Significant? I 1 - 0 = 1 0.5000 no II 0 - 0 = 0 1.0000 no III 0 - 0 = 0 1.0000 no Cavender's quadratic invariants (type L) using purines vs. pyrimidines (these are expected to be zero, and thus have a nonsignificant chi-square, for the correct tree topology) They will be misled if there are substantially different evolutionary rate between sites, or different purine:pyrimidine ratios from 1:1. Tree I: Contingency Table 2 8 1 2 Quadratic invariant = 4.0 Chi-square = 0.23111 (not significant) Tree II: Contingency Table 1 5 1 6 Quadratic invariant = -1.0 Chi-square = 0.01407 (not significant) Tree III: Contingency Table 1 2 6 4 Quadratic invariant = 8.0 Chi-square = 0.66032 (not significant) Cavender's quadratic invariants (type K) using purines vs. pyrimidines (these are expected to be zero for the correct tree topology) They will be misled if there are substantially different evolutionary rate between sites, or different purine:pyrimidine ratios from 1:1. No statistical test is done on them here. Tree I: -9.0 Tree II: 4.0 Tree III: 5.0 ``` |
