# Supplementary material for: Probabilistic Phylogenetic Inference with Insertions and Deletions
Source: PLoS Comput Biol. 2008 Sep 19;4(9):e1000172. doi: 10.1371/journal.pcbi.1000172 (PMC2527138; doi:10.1371/journal.pcbi.1000172)
Supplement: Dataset S1 — Supplemental Material (24.89 MB GZ) [file pcbi.1000172.s001.gz › erate-supplement-R2/src/phylip3.66-erate/doc/pars.html]

pars


version 3.66

# Pars - Discrete character parsimony

© Copyright 1986-2006 by the University of
Washington. Written by Joseph Felsenstein. Permission is granted to copy
this document provided that no fee is charged for it and that this copyright
notice is not removed.

Pars is a general parsimony program which carries out the Wagner
parsimony method with multiple states. Wagner parsimony
allows changes among all states. The criterion is to find the tree which
requires the minimum number of changes.
The Wagner method was originated by Eck and Dayhoff (1966) and by Kluge and
Farris (1969). Here are its assumptions:

1. Ancestral states are unknown unknown.- Different characters evolve independently.- Different lineages evolve independently.- Changes to all other states are equally probable (Wagner).- These changes are a priori improbable over the
           evolutionary time spans involved in the differentiation of the
           group in question.- Other kinds of evolutionary event such as retention of polymorphism
             are far less probable than these state changes.- Rates of evolution in different lineages are sufficiently low that
               two changes in a long segment of the tree are far less probable
               than one change in a short segment.

That these are the assumptions of parsimony methods has been documented
in a series of papers of mine: (1973a, 1978b, 1979, 1981b,
1983b, 1988b). For an opposing view arguing that the parsimony methods
make no substantive
assumptions such as these, see the papers by Farris (1983) and Sober (1983a,
1983b), but also read the exchange between Felsenstein and Sober (1986).

## INPUT FORMAT

The input for Pars is the standard input for discrete characters
programs, described above in the documentation file for the
discrete-characters programs, except that multiple states (up to 8 of them)
are allowed. Any characters other than "?" are allowed as states, up to a
maximum of 8 states. In fact, one can
use different symbols in different columns of the data matrix,
although it is rather unlikely that you would want to do that.
The symbols you can use are:

- The digits 0-9,- The letters A-Z and a-z,- The symbols "!\"#$%&'()\*+,-./:;<=>?@\[\\]^\_`\{|}~  
      (of these, probably only + and - will be of interest to most users).

But note that these do *not* include blank (" "). Blanks in the
input data are simply skipped by the program, so that they can be used to
make characters into groups for ease of viewing.
The "?" (question mark) symbol has special meaning. It is allowed in the
input but is not available as the symbol of a state. Rather, it means that
the state is unknown.

Pars can handle both bifurcating and multifurcating trees. In doing its
search for most parsimonious trees, it adds species not only by creating new
forks in the middle of existing branches, but it also tries putting them at
the end of new branches which are added to existing forks. Thus it searches
among both bifurcating and multifurcating trees. If a branch in a tree
does not have any characters which might change in that branch in the most
parsimonious tree, it does not save that tree. Thus in any tree that
results, a branch exists only if some character has a most parsimonious
reconstruction that would involve change in that branch.

It also saves a number of trees tied for best (you can alter the number
it saves using the V option in the menu). When rearranging trees, it
tries rearrangements of all of the saved trees. This makes the algorithm
slower than earlier programs such as Mix.

The options are selected using a menu:

|  |
| --- |
| ``` Discrete character parsimony algorithm, version 3.6  Setting for this run:   U                 Search for best tree?  Yes   S                        Search option?  More thorough search   V              Number of trees to save?  100   J     Randomize input order of species?  No. Use input order   O                        Outgroup root?  No, use as outgroup species 1   T              Use Threshold parsimony?  No, use ordinary parsimony   W                       Sites weighted?  No   M           Analyze multiple data sets?  No   I            Input species interleaved?  Yes   0   Terminal type (IBM PC, ANSI, none)?  ANSI   1    Print out the data at start of run  No   2  Print indications of progress of run  Yes   3                        Print out tree  Yes   4          Print out steps in each site  No   5  Print character at all nodes of tree  No   6       Write out trees onto tree file?  Yes    Y to accept these or type the letter for one to change ``` |

The Weights (W) option
takes the weights from a file whose default name is "weights". The weights
follow the format described in the main documentation file, with integer
weights from 0 to 35 allowed by using the characters 0, 1, 2, ..., 9 and
A, B, ... Z.

The User tree (option U) is read from a file whose default name is
intree.
The trees can be multifurcating. They must be preceded in the file by a
line giving the number of trees in the file.

The options J, O, T, and M are the usual Jumble, Outgroup,
Threshold parsimony, and Multiple Data Sets options,
described either
in the main documentation file or in the Discrete Characters Programs
documentation file.

The S (search) option controls how, and how much, rearrangement is done on the
tied trees that are saved by the program. If the "More thorough search"
option (the default) is chosen, the program will save multiple tied trees,
without collapsing internal branches that have no evidence of change on them.
It will subsequently rearrange on all parts of each of those trees.
If the "Less thorough search" option is chosen, before saving,
the program will collapse
all branches that have no evidence that there is any change on that branch.
This leads to less attempted rearrangement. If the "Rearrange on one
best tree" option is chosen, only the first of the tied trees is used for
rearrangement. This is faster but less thorough. If your trees are likely
to have large multifurcations, do not use the default "More thorough search"
option as it could result in too large a number of trees being saved.

The M (multiple data sets option) will ask you whether you want to
use multiple sets of weights (from the weights file) or multiple data sets.
The ability to use a single data set with multiple weights means that
much less disk space will be used for this input data. The bootstrapping
and jackknifing tool Seqboot has the ability to create a weights file with
multiple weights.

The O (outgroup) option will have no effect if the U (user-defined tree)
option is in effect.
The T (threshold) option allows a continuum of methods
between parsimony and compatibility. Thresholds less than or equal to 1.0 do
not have any meaning and should
not be used: they will result in a tree dependent only on the input
order of species and not at all on the data!

## OUTPUT FORMAT

Output is standard: if option 1 is toggled on, the data is printed out,
with the convention that "." means "the same as in the first species".
Then comes a list of equally parsimonious trees.
Each tree has branch lengths. These are computed using an algorithm
published by Hochbaum and Pathria (1997) which I first heard of from
Wayne Maddison who invented it independently of them. This algorithm
averages the number of reconstructed changes of state over all sites a
over all possible most parsimonious placements of the changes of state
among branches. Note that it does not correct in any way for multiple
changes that overlay each other.

If option 2 is
toggled on a table of the
number of changes of state required in each character is also
printed. If option 5 is toggled
on, a table is printed
out after each tree, showing for each branch whether there are known to be
changes in the branch, and what the states are inferred to have been at the
top end of the branch. This is a reconstruction of the ancestral sequences
in the tree. If you choose option 5, a menu item D appears which gives you
the opportunity to turn off dot-differencing so that complete ancestral
sequences are shown. If the inferred state is a "?",
there will be multiple
equally-parsimonious assignments of states; the user must work these out for
themselves by hand.
If option 6 is left in its default state the trees
found will be written to a tree file, so that they are available to be used
in other programs. If the program finds multiple
trees tied for best, all of these are written out onto the output tree
file. Each is followed by a numerical weight in square brackets (such as
[0.25000]). This is needed when we use the trees to make a consensus
tree of the results of bootstrapping or jackknifing, to avoid overrepresenting
replicates that find many tied trees.

If the U (User Tree) option is used and more than one tree is supplied, the
program also performs a statistical test of each of these trees against the
best tree. This test, which is a version of the test proposed by
Alan Templeton (1983) and evaluated in a test case by me (1985a). It is
closely parallel to a test using log likelihood differences
due to Kishino and Hasegawa (1989), and
uses the mean and variance of
step differences between trees, taken across sites. If the mean
is more than 1.96 standard deviations different then the trees are declared
significantly different. The program
prints out a table of the steps for each tree, the differences of
each from the best one, the variance of that quantity as determined by
the step differences at individual sites, and a conclusion as to
whether that tree is or is not significantly worse than the best one.
It is important to understand that the test assumes that all the discrete
characters are evolving independently, which is unlikely to be true for

If there are more than two trees, the test done is an extension of
the KHT test, due to Shimodaira and Hasegawa (1999). They pointed out
that a correction for the number of trees was necessary, and they
introduced a resampling method to make this correction. In the version
used here the variances and covariances of the sums of steps across
characters are computed for all pairs of trees. To test whether the
difference between each tree and the best one is larger than could have
been expected if they all had the same expected number of steps,
numbers of steps for all trees are sampled with these covariances and equal
means (Shimodaira and Hasegawa's "least favorable hypothesis"),
and a P value is computed from the fraction of times the difference between
the tree's value and the lowest number of steps exceeds that actually
observed. Note that this sampling needs random numbers, and so the
program will prompt the user for a random number seed if one has not
already been supplied. With the two-tree KHT test no random numbers
are used.

In either the KHT or the SH test the program
prints out a table of the number of steps for each tree, the differences of
each from the lowest one, the variance of that quantity as determined by
the differences of the numbers of steps at individual characters,
and a conclusion as to
whether that tree is or is not significantly worse than the best one.

Option 6 in the menu controls whether the tree estimated by the program
is written onto a tree file. The default name of this output tree file
is "outtree". If the U option is in effect, all the user-defined
trees are written to the output tree file.

---

### TEST DATA SET

|  |
| --- |
| ```      5    6 Alpha     110110 Beta      110000 Gamma     100110 Delta     001001 Epsilon   001110 ``` |

---

### TEST SET OUTPUT (with all numerical options on)

|  |
| --- |
| ``` Discrete character parsimony algorithm, version 3.66   5 species,   6  sites   Name         Sequences ----         ---------  Alpha        110110 Beta         ...00. Gamma        .0.... Delta        001001 Epsilon      001...   One most parsimonious tree found:                               +Epsilon               +----------------3     +--------2                +-------------------------Delta        |        |     |        +Gamma        |     1----------------Beta         |     +Alpha        requires a total of      8.000    between      and       length   -------      ---       ------      1           2         1.00      2           3         2.00      3      Epsilon        0.00      3      Delta          3.00      2      Gamma          0.00      1      Beta           2.00      1      Alpha          0.00  steps in each site:          0   1   2   3   4   5   6   7   8   9      *-----------------------------------------     0|       1   1   1   2   2   1              From    To     Any Steps?    State at upper node                              ( . means same as in the node below it on tree)            1                110110    1      2         yes    .0....    2      3         yes    0.1...    3   Epsilon      no     ......    3   Delta        yes    ...001    2   Gamma        no     ......    1   Beta         yes    ...00.    1   Alpha        no     ...... ``` |
