# Supplementary material for: Probabilistic Phylogenetic Inference with Insertions and Deletions
Source: PLoS Comput Biol. 2008 Sep 19;4(9):e1000172. doi: 10.1371/journal.pcbi.1000172 (PMC2527138; doi:10.1371/journal.pcbi.1000172)
Supplement: Dataset S1 — Supplemental Material (24.89 MB GZ) [file pcbi.1000172.s001.gz › erate-supplement-R2/src/phylip3.66-erate/doc/move.html]

move


version 3.6

# Move - Interactive mixed method parsimony

© Copyright 1986-2004 by the University of
Washington. Written by Joseph Felsenstein. Permission is granted to copy
this document provided that no fee is charged for it and that this copyright
notice is not removed.

Move is an interactive parsimony program, inspired by Wayne Maddison and
David Maddison's marvellous program MacClade, which is written for Apple
Macintosh computers. Move reads in a data set which is prepared in almost the
same format as one for the mixed method parsimony program Mix. It allows
the user to choose an initial tree, and displays this tree on the screen. The
user can look at different characters and the way their states are
distributed on that tree, given the most parsimonious reconstruction of state
changes for that particular tree. The user then can specify how the tree is to
be rearraranged, rerooted or written out to a file. By looking at different
rearrangements of the tree the user can manually search for the most
parsimonious tree, and can get a feel for how different characters are affected by changes in the tree topology.

This program is compatible with fewer computer systems than the other
programs in PHYLIP. It can be adapted to MSDOS systems or to
any system whose screen or terminals emulate DEC VT100
terminals (such as Telnet programs for logging in to remote computers over a
TCP/IP network,
VT100-compatible windows in the X windowing system, and any
terminal compatible with ANSI standard terminals).
For any other screen types, there is a generic option which does
not make use of screen graphics characters to display the character
states. This will be less effective, as the states will be less
easy to see when displayed.

The input data file is set up almost identically to the data files for
Mix.

The user interaction starts with the program presenting a menu. The
menu looks like this:

|  |
| --- |
| ``` Interactive mixed parsimony algorithm, version 3.6  Settings for this run:   X                         Use Mixed method?  No   P                         Parsimony method?  Wagner   A                     Use ancestral states?  No   F                  Use factors information?  No   O                            Outgroup root?  No, use as outgroup species   1   W                           Sites weighted?  No   T                  Use Threshold parsimony?  No, use ordinary parsimony   U  Initial tree (arbitrary, user, specify)?  Arbitrary   0       Graphics type (IBM PC, ANSI, none)?  ANSI   S                 Width of terminal screen?  80   L                Number of lines on screen?  24  Are these settings correct? (type Y or the letter for one to change) ``` |

The P (Parsimony method) option selects among Wagner parsimony
and Camin-Sokal parsimony. If X (miXed methods) is selected the
P menu item disappears, as it is then irrelevant.

The X (miXed methods), A (Ancestors), F (Factors),
O (Outgroup), T (Threshold), and 0 (Graphics type) options are the usual
ones and are described in the main documentation page and in the
discrete characters program documentation page.

The U (initial tree) option allows the user to choose whether
the initial tree is to be arbitrary, interactively specified by the user, or
read from a tree file. Typing U causes the program to change among the
three possibilities in turn. I
would recommend that for a first run, you allow the tree to be set up
arbitrarily (the default), as the "specify" choice is difficult
to use and the "user tree" choice requires that you have available a tree file
with the tree topology of the initial tree.
Its default name is intree. The program will ask you for its name if
it looks for the input tree file and does not find one of this name.
If you wish to set up some
particular tree you can also do that by the rearrangement commands specified
below.

The T (threshold) option allows a continuum of methods between
parsimony and compatibility. Thresholds less than or equal to 1.0 do not
have any
meaning and should not be used: they will result in a tree dependent only on
the input order of species and not at all on the data!

The usual W (Weights) option is available in Move. It allows integer
weights up to 36, using the symbols 0-9 and A-Z. Increased weight on a step
increases both the number of parsimony steps on the character and the
contribution it makes to the number of compatibilities.

The F (Factors)
option is available in this program. It is only used to inform the program
which
groups of characters are to be counted together in computing the number of
characters compatible with the tree. Thus if three binary characters are all
factors of the same multistate character, the multistate character will
be counted as compatible with the tree only if all three factors are compatible
with it.

The S (Screen width) option allows the width in characters of the
display to be adjusted when more then 80 characters can be displayed on
the user's screen.

The L (screen Lines) option allows the user to change the height of the
screen (in lines of characters) that is assumed to be available on the
display. This may be particularly helpful when displaying large trees
on terminals that have more than 24 lines per screen, or on workstation
or X-terminal screens that can emulate the ANSI terminals with more than
24 lines.

After the initial menu is displayed and the choices are made,
the program then sets up an initial tree and displays it. Below it will be a
one-line menu of possible commands, which looks like this:

```
NEXT? (Options: R # + - S . T U W O F C H ? X Q) (H or ? for Help)
```

If you type H or ? you will get a single screen showing a description of each
of these commands in a few words. Here are slightly more detailed
descriptions:

R: ("Rearrange"). This command asks for the number of a node which is to be removed from the tree. It and everything to the right of it on the tree is to be removed (by breaking the branch immediately below it). The command also asks for the number of a node below which that group is to be inserted. If an impossible number is given, the program refuses to carry out the rearrangement and asks for a new command. The rearranged tree is displayed: it will often have a different number of steps than the original. If you wish to undo a rearrangement, use the Undo command, for which see below. #: This command, and the +, - and S commands described below, determine which character has its states displayed on the branches of the trees. The initial tree displayed by the program does not show states of sites. When # is typed, the program does not ask the user which character is to be shown but automatically shows the states of the next binary character that is not compatible with the tree (the next character that does not perfectly fit the current tree). The search for this character "wraps around" so that if it reaches the last character without finding one that is not compatible with the tree, the search continues at the first character; if no incompatible character is found the current character is shown, and if no current character is shown then the first character is shown. The display takes the form of different symbols or textures on the branches of the tree. The state of each branch is actually the state of the node above it. A key of the symbols or shadings used for states 0, 1 and ? are shown next to the tree. State ? means that either state 0 or state 1 could exist at that point on the tree, and that the user may want to consider the different possibilities, which are usually apparent by inspection. +: This command is the same as # except that it goes forward one character, showing the states of the next character. If no character has been shown, using + will cause the first character to be shown. Once the last character has been reached, using + again will show the first character. -: This command is the same as + except that it goes backwards, showing the states of the previous character. If no character has been shown, using - will cause the last character to be shown. Once character number 1 has been reached, using - again will show the last character. S: ("Show"). This command is the same as + and - except that it causes the program to ask you for the number of a character. That character is the one whose states will be displayed. If you give the character number as 0, the program will go back to not showing the states of the characters. . (dot): This command simply causes the current tree to be redisplayed. It is of use when the tree has partly disappeared off of the top of the screen owing to too many responses to commands being printed out at the bottom of the screen. T: ("Try rearrangements"). This command asks for the name of a node. The part of the tree at and above that node is removed from the tree. The program tries to re-insert it in each possible location on the tree (this may take some time, and the program reminds you to wait). Then it prints out a summary. For each possible location the program prints out the number of the node to the right of the place of insertion and the number of steps required in each case. These are divided into those that are better, tied, or worse than the current tree. Once this summary is printed out, the group that was removed is inserted into its original position. It is up to you to use the R command to actually carry out any the arrangements that have been tried. U: ("Undo"). This command reverses the effect of the most recent rearrangement, outgroup re-rooting, or flipping of branches. It returns to the previous tree topology. It will be of great use when rearranging the tree and when a rearrangement proves worse than the preceding one -- it permits you to abandon the new one and return to the previous one without remembering its topology in detail. W: ("Write"). This command writes out the current tree onto a tree output file. If the file already has been written to by this run of Move, it will ask you whether you want to replace the contents of the file, add the tree to the end of the file, or not write out the tree to the file. The tree is written in the standard format used by PHYLIP (a subset of the Newick standard). It is in the proper format to serve as the User-Defined Tree for setting up the initial tree in a subsequent run of the program. Note that if you provided the initial tree topology in a tree file and replace its contents, that initial tree will be lost. O: ("Outgroup"). This asks for the number of a node which is to be the outgroup. The tree will be redisplayed with that node as the left descendant of the bottom fork. Under some options (for example the Camin-Sokal parsimony method or the Ancestor state options), the number of steps required on the tree may change on re-rooting. Note that it is possible to use this to make a multi-species group the outgroup (i.e., you can give the number of an interior node of the tree as the outgroup, and the program will re-root the tree properly with that on the left of the bottom fork). F: ("Flip"). This asks for a node number and then flips the two branches at that node, so that the left-right order of branches at that node is changed. This does not actually change the tree topology (or the number of steps on that tree) but it does change the appearance of the tree. .br C: ("Clade"). When the data consist of more than 12 species (or more than half the number of lines on the screen if this is not 24), it may be difficult to display the tree on one screen. In that case the tree will be squeezed down to one line per species. This is too small to see all the interior states of the tree. The C command instructs the program to print out only that part of the tree (the "clade") from a certain node on up. The program will prompt you for the number of this node. Remember that thereafter you are not looking at the whole tree. To go back to looking at the whole tree give the C command again and enter "0" for the node number when asked. Most users will not want to use this option unless forced to. H: ("Help"). Prints a one-screen summary of what the commands do, a few words for each command. ?: ("huh?"). A synonym for H. Same as Help command. X: ("Exit"). Exit from program. If the current tree has not yet been saved into a file, the program will ask you whether it should be saved. Q: ("Quit"). A synonym for X. Same as the eXit command.

## ADAPTING THE PROGRAM TO YOUR COMPUTER AND TO YOUR TERMINAL

As we have seen, the initial menu of the program allows you to choose
among three screen types (PCDOS, Ansi, and none).
If you want to avoid having to make this choice every time, you can change
some of the constants in the file phylip.h to have the terminal type
initialize itself in the proper way, and recompile. We have tried to
have the default values be correct for PC, Macintosh, and Unix
screens. If the setting is "none" (which is necessary on
Macintosh MacOS 9 screens), the special graphics
characters will not be used to indicate nucleotide states, but only letters
will be used for the four nucleotides. This is less easy to look at.

The constants that need attention are ANSICRT and IBMCRT.
Currently these are both set to "false" on Macintosh MacOS 9 systems,
to "true" on MacOS X and on Unix/Linux
systems, and IBMCRT is set to "true" on Windows systems. If your system
has an ANSI compatible terminal, you might want to find the
definition of ANSICRT in phylip.h and set it to "true", and
IBMCRT to "false".

## MORE ABOUT THE PARSIMONY CRITERION

Move uses as its numerical criterion the Wagner and
Camin-Sokal parsimony methods in mixture, where each character can have
its method specified separately. The program defaults to carrying out Wagner
parsimony.

The Camin-Sokal parsimony method explains the data by assuming that changes 0
--> 1 are allowed but not changes 1 --> 0. Wagner parsimony allows both kinds
of changes. (This under the assumption that 0 is the ancestral state, though
the program allows reassignment of the ancestral state, in which case we must
reverse the state numbers 0 and 1 throughout this discussion). The criterion
is to find the tree which requires the minimum number of changes. The Camin-
Sokal method is due to Camin and Sokal (1965) and the Wagner method to Eck and
Dayhoff (1966) and to Kluge and Farris (1969).

Here are the assumptions of these two methods:

1. Ancestral states are known (Camin-Sokal) or unknown (Wagner).- Different characters evolve independently.- Different lineages evolve independently.- Changes 0 --> 1 are much more probable than changes 1 --> 0 (Camin-Sokal)
         or equally probable (Wagner).- Both of these kinds of changes are a priori improbable over the
           evolutionary time spans involved in the differentiation of the group in
           question.- Other kinds of evolutionary event such as retention of
             polymorphism are far less probable than 0 --> 1 changes.- Rates of
               evolution in different lineages are sufficiently low that two changes in a long
               segment of the tree are far less probable than one change in a short segment.

That these are the assumptions of parsimony methods has been documented
in a series of papers of mine: (1973a, 1978b, 1979, 1981b,
1983b, 1988b). For an opposing view arguing that the parsimony methods
make no substantive
assumptions such as these, see the papers by Farris (1983) and Sober (1983a,
1983b), but also read the exchange between Felsenstein and Sober (1986).

Below is a test data set, but we cannot show the
output it generates because of the interactive nature of the program.

---

### TEST DATA SET

|  |
| --- |
| ```      5    6 Alpha     110110 Beta      110000 Gamma     100110 Delta     001001 Epsilon   001110 ``` |
