# Supplementary material for: Probabilistic Phylogenetic Inference with Insertions and Deletions
Source: PLoS Comput Biol. 2008 Sep 19;4(9):e1000172. doi: 10.1371/journal.pcbi.1000172 (PMC2527138; doi:10.1371/journal.pcbi.1000172)
Supplement: Dataset S1 — Supplemental Material (24.89 MB GZ) [file pcbi.1000172.s001.gz › erate-supplement-R2/src/phylip3.66-erate/doc/dnamlk.html]

dnamlk


version 3.66

# Dnamlk -- DNA Maximum Likelihood program with molecular clock

© Copyright 1986-2006 by the University of
Washington. Written by Joseph Felsenstein. Permission is granted to copy
this document provided that no fee is charged for it and that this copyright
notice is not removed.

This program implements the maximum likelihood method for DNA
sequences under the constraint that the trees estimated must be
consistent with a molecular clock. The molecular clock is the
assumption that the tips of the tree are all equidistant, in branch
length, from its root. This program is indirectly related to Dnaml.
Details of the algorithm are not yet published, but many aspects
of it are similar to Dnaml, and these are published in
the paper by Felsenstein and Churchill (1996).
The model of base substitution allows the expected frequencies
of the four bases to be unequal, allows the expected frequencies of
transitions and transversions to be unequal, and has several
ways of allowing different rates of evolution at
different sites.

The assumptions of the model are:

1. Each site in the sequence evolves independently.- Different lineages evolve independently.- There is a molecular clock.- Each site undergoes substitution at an expected rate which is
         chosen from a series of rates (each with a probability of occurrence)
         which we specify.- All relevant sites are included in the sequence, not just those that
           have changed or those that are "phylogenetically informative".- A substitution consists of one of two sorts of events:

             (a): The first kind of event consists of the replacement of the existing base by a base drawn from a pool of purines or a pool of pyrimidines (depending on whether the base being replaced was a purine or a pyrimidine). It can lead either to no change or to a transition. (b): The second kind of event consists of the replacement of the existing base by a base drawn at random from a pool of bases at known frequencies, independently of the identity of the base which is being replaced. This could lead either to a no change, to a transition or to a transversion.

             The ratio of the two
             purines in the purine replacement pool is the same as their ratio in the
             overall pool, and similarly for the pyrimidines.

             The ratios of transitions to transversions can be set by the
             user. The substitution process can be diagrammed as follows:
             Suppose that you specified A, C, G, and T base frequencies of
             0.24, 0.28, 0.27, and 0.21.

             - First kind of event:

               1. Determine whether the existing base is a purine or a pyrimidine.- Draw from the proper pool:

                    ```
                          Purine pool:                Pyrimidine pool:

                         |               |            |               |
                         |   0.4706 A    |            |   0.5714 C    |
                         |   0.5294 G    |            |   0.4286 T    |
                         | (ratio is     |            | (ratio is     |
                         |  0.24 : 0.27) |            |  0.28 : 0.21) |
                         |_______________|            |_______________|
                    ```

               - Second kind of event:

                 Draw from the overall pool:

                 ```
                               |                  |
                               |      0.24 A      |
                               |      0.28 C      |
                               |      0.27 G      |
                               |      0.21 T      |
                               |__________________|
                 ```

             Note that if the existing base is, say, an A, the first kind of event has
             a 0.4706 probability of "replacing" it by another A. The second kind of
             event has a 0.24 chance of replacing it by another A. This rather
             disconcerting model is used because it has nice mathematical properties that
             make likelihood calculations far easier. A closely similar, but not
             precisely identical model having different rates of transitions and
             transversions has been used by Hasegawa et. al. (1985b). The transition
             probability formulas for the current model were given (with my
             permission) by Kishino and Hasegawa (1989). Another explanation is
             available in the paper by Felsenstein and Churchill (1996).

Note the assumption that we are looking at all sites, including those
that have not changed at all. It is important not to restrict attention
to some sites based on whether or not they have changed; doing that
would bias branch lengths by making them too long, and that in turn
would cause the method to misinterpret the meaning of those sites that
had changed.

This program uses a Hidden Markov Model (HMM)
method of inferring different rates of evolution at different sites. This
was described in a paper by me and Gary Churchill (1996). It allows us to
specify to the program that there will be
a number of different possible evolutionary rates, what the prior
probabilities of occurrence of each is, and what the average length of a
patch of sites all having the same rate. The rates can also be chosen
by the program to approximate a Gamma distribution of rates, or a
Gamma distribution plus a class of invariant sites. The program computes the
the likelihood by summing it over all possible assignments of rates to sites,
weighting each by its prior probability of occurrence.

For example, if we have used the C and A options (described below) to specify
that there are three possible rates of evolution, 1.0, 2.4, and 0.0,
that the prior probabilities of a site having these rates are 0.4, 0.3, and
0.3, and that the average patch length (number of consecutive sites
with the same rate) is 2.0, the program will sum the likelihood over
all possibilities, but giving less weight to those that (say) assign all
sites to rate 2.4, or that fail to have consecutive sites that have the
same rate.

The Hidden Markov Model framework for rate variation among sites
was independently developed by Yang (1993, 1994, 1995). We have
implemented a general scheme for a Hidden Markov Model of
rates; we allow the rates and their prior probabilities to be specified
arbitrarily by the user, or by a discrete approximation to a Gamma
distribution of rates (Yang, 1995), or by a mixture of a Gamma
distribution and a class of invariant sites.

This feature effectively removes the artificial assumption that all sites
have the same rate, and also means that we need not know in advance the
identities of the sites that have a particular rate of evolution.

Another layer of rate variation also is available. The user can assign
categories of rates to each site (for example, we might want first, second,
and third codon positions in a protein coding sequence to be three different
categories. This is done with the categories input file and the C option.
We then specify (using the menu) the relative rates of evolution of sites
in the different categories. For example, we might specify that first,
second, and third positions evolve at relative rates of 1.0, 0.8, and 2.7.

If both user-assigned rate categories and Hidden Markov Model rates
are allowed, the program assumes that the
actual rate at a site is the product of the user-assigned category rate
and the Hidden Markov Model regional rate. (This may not always make
perfect biological sense: it would be more natural to assume some upper
bound to the rate, as we have discussed in the Felsenstein and Churchill
paper). Nevertheless you may want to use both types of rate variation.

## INPUT FORMAT AND OPTIONS

Subject to these assumptions, the program is a
correct maximum likelihood method. The
input is fairly standard, with one addition. As usual the first line of the
file gives the number of species and the number of sites.

Next come the species data. Each
sequence starts on a new line, has a ten-character species name
that must be blank-filled to be of that length, followed immediately
by the species data in the one-letter code. The sequences must either
be in the "interleaved" or "sequential" formats
described in the Molecular Sequence Programs document. The I option
selects between them. The sequences can have internal
blanks in the sequence but there must be no extra blanks at the end of the
terminated line. Note that a blank is not a valid symbol for a deletion.

The options are selected using an interactive menu. The menu looks like this:

|  |
| --- |
| ``` Nucleic acid sequence    Maximum Likelihood method with molecular clock, version 3.6  Settings for this run:   U                 Search for best tree?  Yes   T        Transition/transversion ratio:  2.0   F       Use empirical base frequencies?  Yes   C   One category of substitution rates?  Yes   R           Rate variation among sites?  constant rate   G                Global rearrangements?  No   W                       Sites weighted?  No   J   Randomize input order of sequences?  No. Use input order   M           Analyze multiple data sets?  No   I          Input sequences interleaved?  Yes   0   Terminal type (IBM PC, ANSI, none)?  ANSI   1    Print out the data at start of run  No   2  Print indications of progress of run  Yes   3                        Print out tree  Yes   4       Write out trees onto tree file?  Yes   5   Reconstruct hypothetical sequences?  No  Are these settings correct? (type Y or the letter for one to change) ``` |

The user either types "Y" (followed, of course, by a carriage-return)
if the settings shown are to be accepted, or the letter or digit corresponding
to an option that is to be changed.

The options U, W, J, O, M, and 0 are the usual ones. They are described in the
main documentation file of this package. Option I is the same as in
other molecular sequence programs and is described in the documentation file
for the sequence programs.

The T option in this program does not stand for Threshold,
but instead is the Transition/transversion option. The user is prompted for
a real number greater than 0.0, as the expected ratio of transitions to
transversions. Note
that this is not the ratio of the first to the second kinds of events,
but the resulting expected ratio of transitions to transversions. The exact
relationship between these two quantities depends on the frequencies in the
base pools. The default value of the T parameter if you do not use the T
option is 2.0.

The F (Frequencies) option is one which may save users much time. If you
want to use the empirical frequencies of the bases, observed in the input
sequences, as the base frequencies, you simply use the default setting of
the F option. These empirical
frequencies are not really the maximum likelihood estimates of the base
frequencies, but they will often be close to those values (what they are is
maximum likelihood estimates under a "star" or "explosion" phylogeny).
If you change the setting of the F option you will be prompted for the
frequencies of the four bases. These must add to 1 and are to be typed on
one line separated by blanks, not commas.

The R (Hidden Markov Model rates) option allows the user to
approximate a Gamma distribution of rates among sites, or a
Gamma distribution plus a class of invariant sites, or to specify how
many categories of
substitution rates there will be in a Hidden Markov Model of rate
variation, and what are the rates and probabilities
for each. By repeatedly selecting the R option one toggles among
no rate variation, the Gamma, Gamma+I, and general HMM possibilities.

If you choose Gamma or Gamma+I the program will ask how many rate
categories you want. If you have chosen Gamma+I, keep in mind that
one rate category will be set aside for the invariant class and only
the remaining ones used to approximate the Gamma distribution.
For the approximation we do not use the quantile method of Yang (1995)
but instead use a quadrature method using generalized Laguerre
polynomials. This should give a good approximation to the Gamma
distribution with as few as 5 or 6 categories.

In the Gamma and Gamma+I cases, the user will be
asked to supply the coefficient of variation of the rate of substitution
among sites. This is different from the parameters used by Nei and Jin
(1990) but
related to them: their parameter *a* is also known as "alpha",
the shape parameter of the Gamma distribution. It is
related to the coefficient of variation by

     CV = 1 / a1/2

or

     a = 1 / (CV)2

(their parameter *b* is absorbed here by the requirement that time is scaled so
that the mean rate of evolution is 1 per unit time, which means that *a = b*).
As we consider cases in which the rates are less variable we should set *a*
larger and larger, as *CV* gets smaller and smaller.

If the user instead chooses the general Hidden Markov Model option,
they are first asked how many HMM rate categories there
will be (for the moment there is an upper limit of 9,
which should not be restrictive). Then
the program asks for the rates for each category. These rates are
only meaningful relative to each other, so that rates 1.0, 2.0, and 2.4
have the exact same effect as rates 2.0, 4.0, and 4.8. Note that an
HMM rate category
can have rate of change 0, so that this allows us to take into account that
there may be a category of sites that are invariant. Note that the run time
of the program will be proportional to the number of HMM rate categories:
twice as
many categories means twice as long a run. Finally the program will ask for
the probabilities of a random site falling into each of these
regional rate categories. These probabilities must be nonnegative and sum to
1. Default
for the program is one category, with rate 1.0 and probability 1.0 (actually
the rate does not matter in that case).

If more than one HMM rate category is specified, then another
option, A, becomes
If more than one category is specified, then another option, A, becomes
visible in the menu. This allows us to specify that we want to assume that
sites that have the same HMM rate category are expected to be clustered
so that there is autocorrelation of rates. The
program asks for the value of the average patch length. This is an expected
length of patches that have the same rate. If it is 1, the rates of
successive sites will be independent. If it is, say, 10.25, then the
chance of change to a new rate will be 1/10.25 after every site. However
the "new rate" is randomly drawn from the mix of rates, and hence could
even be the same. So the actual observed length of patches with the same
rate will be a bit larger than 10.25. Note below that if you choose
multiple patches, there will be an estimate in the output file as to
which combination of rate categories contributed most to the likelihood.

Note that the autocorrelation scheme we use is somewhat different
from Yang's (1995) autocorrelated Gamma distribution. I am unsure
whether this difference is of any importance -- our scheme is chosen
for the ease with which it can be implemented.

The C option allows user-defined rate categories. The user is prompted
for the number of user-defined rates, and for the rates themselves,
which cannot be negative but can be zero. These numbers, which must be
nonnegative (some could be 0),
are defined relative to each other, so that if rates for three categories
are set to 1 : 3 : 2.5 this would have the same meaning as setting them
to 2 : 6 : 5.
The assignment of rates to
sites is then made by reading a file whose default name is "categories".
It should contain a string of digits 1 through 9. A new line or a blank
can occur after any character in this string. Thus the categories file
might look like this:

```
122231111122411155
1155333333444
```

With the current options R, A, and C the program has gained greatly in its
ability to infer different rates at different sites and estimate
phylogenies under a more realistic model. Note that Likelihood Ratio
Tests can be used to test whether one combination of rates is
significantly better than another, provided one rate scheme represents
a restriction of another with fewer parameters. The number of parameters
needed for rate variation is the number of regional rate categories, plus
the number of user-defined rate categories less 2, plus one if the
regional rate categories have a nonzero autocorrelation.

The G (global search) option causes, after the last species is added to
the tree, each possible group to be removed and re-added. This improves the
result, since the position of every species is reconsidered. It
approximately triples the run-time of the program.

The User tree (option U) is read from a file whose default name is
intree.
The trees can be multifurcating. This allows us to test the
hypothesis that a given branch has zero length.

If the U (user tree) option is chosen another option appears in
the menu, the L option. If it is selected,
it signals the program that it
should take any branch lengths that are in the user tree and
simply evaluate the likelihood of that tree, without further altering
those branch lengths. In the case of a clock, if some branches have lengths
and others do not, the program does not estimate the lengths of those that
do not have lengths given in the user tree. If any of the branches
do not have lengths, the program re-estimates the lengths of all of them.
This is done because estimating some and not others is hard in the
case of a clock.

The W (Weights) option is invoked in the usual way, with only weights 0
and 1 allowed. It selects a set of sites to be analyzed, ignoring the
others. The sites selected are those with weight 1. If the W option is
not invoked, all sites are analyzed.
The Weights (W) option
takes the weights from a file whose default name is "weights". The weights
follow the format described in the main documentation file.

The M (multiple data sets) option will ask you whether you want to
use multiple sets of weights (from the weights file) or multiple data sets
from the input file.
The ability to use a single data set with multiple weights means that
much less disk space will be used for this input data. The bootstrapping
and jackknifing tool Seqboot has the ability to create a weights file with
multiple weights. Note also that when we use multiple weights for
bootstrapping we can also then maintain different rate categories for
different sites in a meaningful way. You should not use the multiple
data sets option without using multiple weights, you should not at the
same time use the user-defined rate categories option (option C).

The algorithm used for searching among trees is faster than it was in
version 3.5, thanks to using a technique invented by David Swofford
and J. S. Rogers. This involves not iterating most branch lengths on most
trees when searching among tree topologies, This is of necessity a
"quick-and-dirty" search but it saves much time.

## OUTPUT FORMAT

The output starts by giving the number of species, the number of sites,
and the base frequencies for A, C, G, and T that have been specified. It
then prints out the transition/transversion ratio that was specified or
used by default. It also uses the base frequencies to compute the actual
transition/transversion ratio implied by the parameter.

If the R (HMM rates) option is used a table of the relative rates of
expected substitution at each category of sites is printed, as well
as the probabilities of each of those rates.

There then follow the data sequences, if the user has selected the menu
option to print them out, with the base sequences printed in
groups of ten bases along the lines of the Genbank and EMBL formats. The
trees found are printed as a rooted
tree topology. The
internal nodes are numbered arbitrarily for the sake of
identification. The number of trees evaluated so far and the log
likelihood of the tree are also given. The branch lengths in the diagram are
roughly proportional to the estimated branch lengths, except that very short
branches are printed out at least three characters in length so that the
connections can be seen.

A table is printed
showing the length of each tree segment, and the time (in units of
expected nucleotide substitutions per site) of each fork in the tree,
measured from the root of the tree. I have not attempted in include
code for approximate confidence limits on branch points, as I have done
for branch lengths in Dnaml, both because of the extreme crudeness of
that test, and because the variation of times for different forks would be
highly correlated.

The log likelihood printed out with the final tree can be used to perform
various likelihood ratio tests. One can, for example, compare runs with
different values of the expected transition/transversion ratio to determine
which value is the maximum likelihood estimate, and what is the allowable range
of values (using a likelihood ratio test, which you will find described in
mathematical statistics books). One could also estimate the base frequencies
in the same way. Both of these, particularly the latter, require multiple runs
of the program to evaluate different possible values, and this might get
expensive.

This program makes possible a (reasonably) legitimate
statistical test of the molecular clock. To do such a test, run Dnaml
and Dnamlk on the same data. If the trees obtained are of the same
topology (when considered as unrooted), it is legitimate to compare
their likelihoods by the likelihood ratio test. In Dnaml the likelihood
has been computed by estimating 2n-3 branch lengths, if their are n tips
on the tree. In Dnamlk it has been computed by estimating n-1 branching
times (in effect, n-1 branch lengths). The difference in the number of
parameters is (2n-3)-(n-1) = n-2. To perform the test take the
difference in log likelihoods between the two runs (Dnaml should be the
higher of the two, barring numerical iteration difficulties) and double
it. Look this up on a chi-square distribution with n-2 degrees of
freedom. If the result is significant, the log likelihood has been
significantly increased by allowing all 2n-3 branch lengths to be
estimated instead of just n-1, and molecular clock may be rejected.

If the U (User Tree) option is used and more than one tree is supplied,
and the program is not told to assume autocorrelation between the
rates at different sites, the
program also performs a statistical test of each of these trees against the
one with highest likelihood. If there are two user trees, the test
done is one which is due to Kishino and Hasegawa (1989), a version
of a test originally introduced by Templeton (1983). In this
implementation it uses the mean and variance of
log-likelihood differences between trees, taken across sites. If the two
trees' means are more than 1.96 standard deviations different
then the trees are
declared significantly different. This use of the empirical variance of
log-likelihood differences is more robust and nonparametric than the
classical likelihood ratio test, and may to some extent compensate for the
any lack of realism in the model underlying this program.

If there are more than two trees, the test done is an extension of
the KHT test, due to Shimodaira and Hasegawa (1999). They pointed out
that a correction for the number of trees was necessary, and they
introduced a resampling method to make this correction. In the version
used here the variances and covariances of the sum of log likelihoods across
sites are computed for all pairs of trees. To test whether the
difference between each tree and the best one is larger than could have
been expected if they all had the same expected log-likelihood,
log-likelihoods for all trees are sampled with these covariances and equal
means (Shimodaira and Hasegawa's "least favorable hypothesis"),
and a P value is computed from the fraction of times the difference between
the tree's value and the highest log-likelihood exceeds that actually
observed. Note that this sampling needs random numbers, and so the
program will prompt the user for a random number seed if one has not
already been supplied. With the two-tree KHT test no random numbers
are used.

In either the KHT or the SH test the program
prints out a table of the log-likelihoods of each tree, the differences of
each from the highest one, the variance of that quantity as determined by
the log-likelihood differences at individual sites, and a conclusion as to
whether that tree is or is not significantly worse than the best one. However
the test is not available if we assume that there
is autocorrelation of rates at neighboring sites (option A) and is not
done in those cases.

The branch lengths printed out are scaled in terms of expected numbers of
substitutions, counting both transitions and transversions but not
replacements of a base by itself, and scaled so that the average rate of
change, averaged over all sites analyzed, is set to 1.0
if there are multiple categories of sites. This means that whether or not
there are multiple categories of sites, the expected fraction of change
for very small branches is equal to the branch length. Of course,
when a branch is twice as
long this does not mean that there will be twice as much net change expected
along it, since some of the changes occur in the same site and overlie or
even reverse each
other. The branch length estimates here are in terms of the expected
underlying numbers of changes. That means that a branch of length 0.26
is 26 times as long as one which would show a 1% difference between
the nucleotide sequences at the beginning and end of the branch. But we
would not expect the sequences at the beginning and end of the branch to be
26% different, as there would be some overlaying of changes.

Because of limitations of the numerical
algorithm, branch length estimates of zero will often print out as small
numbers such as 0.00001. If you see a branch length that small, it is really
estimated to be of zero length.

Another possible source of confusion is the existence of negative values for
the log likelihood. This is not really a problem; the log likelihood is not a
probability but the logarithm of a probability. When it is
negative it simply means that the corresponding probability is less
than one (since we are seeing its logarithm). The log likelihood is
maximized by being made more positive: -30.23 is worse than -29.14.

At the end of the output, if the R option is in effect with multiple
HMM rates, the program will print a list of what site categories
contributed the most to the final likelihood. This combination of
HMM rate categories need not have contributed a majority of the likelihood,
just a plurality. Still, it will be helpful as a view of where the
program infers that the higher and lower rates are. Note that the
use in this calculations of the prior probabilities of different rates,
and the average patch length, gives this inference a "smoothed"
appearance: some other combination of rates might make a greater
contribution to the likelihood, but be discounted because it conflicts
with this prior information. See the example output below to see
what this printout of rate categories looks like.

A second list will also be printed out, showing for each site which
rate accounted for the highest fraction of the likelihood. If the fraction
of the likelihood accounted for is less than 95%, a dot is printed instead.

Option 3 in the menu controls whether the tree is printed out into
the output file. This is on by default, and usually you will want to
leave it this way. However for runs with multiple data sets such as
bootstrapping runs, you will primarily be interested in the trees
which are written onto the output tree file, rather than the trees
printed on the output file. To keep the output file from becoming too
large, it may be wisest to use option 3 to prevent trees being
printed onto the output file.

Option 4 in the menu controls whether the tree estimated by the program
is written onto a tree file. The default name of this output tree file
is "outtree". If the U option is in effect, all the user-defined
trees are written to the output tree file.

Option 5 in the menu controls whether ancestral states are estimated
at each node in the tree. If it is in effect, a table of ancestral
sequences is printed out (including the sequences in the tip species which
are the input sequences). In that table, if a site has a base which
accounts for more than 95% of the likelihood, it is printed in capital
letters (A rather than a). If the best nucleotide accounts for less
than 50% of the likelihood, the program prints out an ambiguity code
(such as M for "A or C") for the set of nucleotides which, taken together,
account for more half of the likelihood. The ambiguity codes are listed
in the sequence programs documentation file. One limitation of the current
version of the program is that when there are multiple HMM rates
(option R) the reconstructed nucleotides are based on only the single
assignment of rates to sites which accounts for the largest amount of the
likelihood. Thus the assessment of 95% of the likelihood, in tabulating
the ancestral states, refers to 95% of the likelihood that is accounted
for by that particular combination of rates.

## PROGRAM CONSTANTS

The constants defined at the beginning of the program include "maxtrees",
the maximum number of user trees that can be processed. It is small (100)
at present to save some further memory but the cost of increasing it
is not very great. Other constants
include "maxcategories", the maximum number of site
categories, "namelength", the length of species names in
characters, and three others, "smoothings", "iterations", and "epsilon", that
help "tune" the algorithm and define the compromise between execution speed and
the quality of the branch lengths found by iteratively maximizing the
likelihood. Reducing iterations and smoothings, and increasing epsilon, will
result in faster execution but a worse result. These values
will not usually have to be changed.

The program spends most of its time doing real arithmetic.
The algorithm, with separate and independent computations
occurring for each pattern, lends itself readily to parallel processing.

## PAST AND FUTURE OF THE PROGRAM

This program was developed in 1989 by combining code from Dnapars and from
Dnaml. It was speeded up
by two major developments, the use of aliasing of nucleotide sites (version
3.1) and pretabulation of some exponentials (added by Akiko Fuseki in version
3.4). In version 3.5 the Hidden Markov Model code was added and the method
of iterating branch lengths was changed from an EM algorithm to direct
search. The Hidden Markov Model code slows things down, especially if
there is autocorrelation between sites, so this version is slower than
version 3.4. Nevertheless we hope that the sacrifice is worth it.

One change that is needed in the future is to put in some way of
allowing for base composition of nucleotide sequences in different parts
of the phylogeny.

---

### TEST DATA SET

|  |
| --- |
| ```    5   13 Alpha     AACGTGGCCAAAT Beta      AAGGTCGCCAAAC Gamma     CATTTCGTCACAA Delta     GGTATTTCGGCCT Epsilon   GGGATCTCGGCCC ``` |

---

### CONTENTS OF OUTPUT FILE (with all numerical options on)

(It was run with HMM rates having gamma-distributed rates
approximated by 5 rate categories,
with coefficient of variation of rates 1.0, and with patch length
parameter = 1.5. Two user-defined rate categories were used, one for
the first 6 sites, the other for the last 7, with rates 1.0 : 2.0.
Weights were used, with sites 1 and 13 given weight 0, and all others
weight 1.)

|  |
| --- |
| ``` Nucleic acid sequence    Maximum Likelihood method with molecular clock, version 3.66   5 species,  13  sites      Site categories are:               1111112222 222       Sites are weighted as follows:               01111 11111 110   Name            Sequences ----            ---------  Alpha        AACGTGGCCA AAT Beta         ..G..C.... ..C Gamma        C.TT.C.T.. C.A Delta        GGTA.TT.GG CC. Epsilon      GGGA.CT.GG CCC   Empirical Base Frequencies:     A       0.23636    C       0.29091    G       0.25455   T(U)     0.21818  Transition/transversion ratio =   2.000000   Discrete approximation to gamma distributed rates  Coefficient of variation of rates = 1.000000  (alpha = 1.000000)  State in HMM    Rate of change    Probability          1           0.264            0.522         2           1.413            0.399         3           3.596            0.076         4           7.086            0.0036         5          12.641            0.000023  Expected length of a patch of sites having the same rate =    1.500   Site category   Rate of change          1           1.000         2           2.000                                                         +-Epsilon      +---------------------------------------------------4     !                                                   +-Delta      --3     !                                             +-------Gamma        +---------------------------------------------2                                                   !     +-Beta                                                       +-----1                                                         +-Alpha        Ln Likelihood =   -58.51728   Ancestor      Node      Node Height     Length  --------      ----      ---- ------     ------  root            3          3             4          4.14816      4.14816    4          Epsilon       4.29765      0.14949    4          Delta         4.29765      0.14949    3             2          3.67519      3.67519    2          Gamma         4.29765      0.62246    2             1          4.12426      0.44907    1          Beta          4.29765      0.17339    1          Alpha         4.29765      0.17339  Combination of categories that contributes the most to the likelihood:               1122121111 111  Most probable category at each site if > 0.95 probability ("." otherwise)               .......... ...   Probable sequences at interior nodes:    node       Reconstructed sequence (caps if > 0.95)      3        .rsstysysr ym.     4        .GgaTctCgg Cc.  Epsilon     GGGATCTCGG CCC  Delta       GGTATTTCGG CCT     2        .AykTcGcCA mA.  Gamma       CATTTCGTCA CAA     1        .AcgTcGCCA AA.  Beta        AAGGTCGCCA AAC  Alpha       AACGTGGCCA AAT ``` |
