# Supplementary material for: Probabilistic Phylogenetic Inference with Insertions and Deletions
Source: PLoS Comput Biol. 2008 Sep 19;4(9):e1000172. doi: 10.1371/journal.pcbi.1000172 (PMC2527138; doi:10.1371/journal.pcbi.1000172)
Supplement: Dataset S1 — Supplemental Material (24.89 MB GZ) [file pcbi.1000172.s001.gz › erate-supplement-R2/src/phylip3.66-erate/doc/restml.html]

restml


version 3.66

# Restml -- Restriction sites Maximum Likelihood program

© Copyright 1986-2006 by The University of
Washington. Written by Joseph Felsenstein. Permission is granted to copy
this document provided that no fee is charged for it and that this copyright
notice is not removed.

This program implements a maximum likelihood method for restriction sites
data (not restriction fragment data). This program is one of the slowest
programs in this package, and can be very tedious to run. It is
possible to have the program search for the maximum likelihood tree.
It will be more practical for some users (those that do not have
fast machines) to use the U (User Tree)
option, which takes less run time, optimizing branch lengths and computing
likelihoods for particular tree topologies suggested by the user. The
model used here is essentially identical to that used by Smouse and Li (1987)
who give explicit expressions for computing the likelihood for three-species
trees. It does not place prior probabilities on trees as they do. The present
program extends their approach to multiple species by
a technique which, while it does not give explicit expressions for likelihoods,
does enable their computation and the iterative improvement of branch
lengths. It also allows for multiple restriction enzymes. The algorithm
has been described in a paper (Felsenstein, 1992). Another relevant
paper is that of DeBry and Slade (1985).

The assumptions of the present model are:

1. Each restriction site evolves independently.- Different lineages evolve independently.- Each site undergoes substitution at an expected rate which we specify.- Substitutions consist of replacement of a nucleotide by one of the
         other three nucleotides, chosen at random.

Note that if the existing base is, say, an A, the chance of it being
replaced by a G is 1/3, and so is the chance that it is replaced by
a T. This means that there can be no difference in the (expected)
rate of transitions and transversions. Users who are upset at this
might ponder the fact that a version allowing different rates of
transitions and transversions would run an estimated 16 times
slower. If it also allowed for unequal frequencies of the four bases,
it would run about 300,000 times slower! For the moment, until a better
method is available, I guess I'll stick with this one!

## INPUT FORMAT AND OPTIONS

Subject to these assumptions, the program is an approximately
correct maximum likelihood method. The
input is fairly standard, with one addition. As usual the first line of the
file gives the number of species and the number of sites, but there is also
a third number, which is the number of different restriction enzymes that were
used to detect the restriction sites. Thus a data set with 10 species and
35 different sites, representing digestion with 4 different enzymes, would
have the first line of the data file look like this:

```
   10   35    4
```

The site data are in standard form. Each species starts with a species name
whose maximum length is given by the constant "nmlngth"
(whose value in the
program as distributed is 10 characters). The name should, as usual, be padded
out to that length with blanks if necessary. The sites data then follows, one
character per site (any blanks will
be skipped and ignored). Like the DNA and protein sequence data, the
restriction sites data may be either in the "interleaved" form or the
"sequential" form. Note that if you are analyzing restriction sites
data with the programs Dollop or Mix or other discrete character
programs, at the moment those programs do not use the "aligned" or
"interleaved" data format. Therefore you may want to avoid that format
when you have restriction sites data that you will want to feed into
those programs.

The presence of a site is indicated by a "+" and the absence by a "-". I have
also allowed the use of "1" and "0" as synonyms for "+" and "-", for
compatibility with Mix and Dollop which do not allow "+" and "-". If the
presence of
the site is unknown (for example, if the DNA containing it has been deleted so
that one
does not know whether it would have contained the site) then the state "?" can
be used to indicate that the state of this site is unknown.

User-defined trees may follow the
data in the usual way. The trees must be unrooted, which means that at their
base they must have a trifurcation.

The options are selected by a menu, which looks like this:

|  |
| --- |
| ``` Restriction site Maximum Likelihood method, version 3.6  Settings for this run:   U                 Search for best tree?  Yes   A               Are all sites detected?  No   S        Speedier but rougher analysis?  Yes   G                Global rearrangements?  No   J   Randomize input order of sequences?  No. Use input order   L                          Site length?  6   O                        Outgroup root?  No, use as outgroup species  1   M           Analyze multiple data sets?  No   I          Input sequences interleaved?  Yes   0   Terminal type (IBM PC, ANSI, none)?  ANSI   1    Print out the data at start of run  No   2  Print indications of progress of run  Yes   3                        Print out tree  Yes   4       Write out trees onto tree file?  Yes    Y to accept these or type the letter for one to change ``` |

The U, J, O, M, and 0 options are the usual ones, described in the main
documentation file. The user trees for option U are read from a file whose
default name is intree. The I option selects between Interleaved and
Sequential input data formats, and is described in the documentation file for
the molecular sequences programs.

The G
(global search) option causes, after the last species is added to the tree,
each possible group to be removed and re-added. This improves the result,
since the position of every species is reconsidered. It approximately triples
the run-time of the program.

The two options specific to this program are the A, and L options. The L
(Length) option allows the user to specify the length in bases of the
restriction sites. At the
moment the program assumes that all sites have the same length (for example,
that all enzymes are 6-base-cutters). The default value for this parameter is
6, which will be used if the L option is not invoked. A desirable future
development for the package would be allowing the L parameter to be different
for every site. It would also be desirable to allow for ambiguities in the
recognition site, since some enzymes recognize 2 or 4 sequences. Both of these
would require fairly complicated programming or else slower execution times.

The A (All) option specifies that all sites are detected, even those for which
all of the species have the recognition sequence absent (character state
"-"). The default condition is that it is assumed that such sites will not
occur in the data. The likelihood computed when the A option is not used is
the probability of the pattern of sites given that tree and conditional on the
pattern not being all absences. This will be realistic for most data, except
for cases in which the data are extracted from sites data for a larger number
of species, in which case some of the site positions could have all absences in
the subset of species. In such cases an effective way of analyzing the data
would be to omit those sites and not use the A option, as such positions, even
if not absolutely excluded, are nevertheless less likely than random to have
been incorporated in the data set.

The W (Weights) option, which is invoked in the input file rather than in
the menu, allows the user to select a subset of sites to
be analyzed. It is invoked in the usual way, except that only weights
0 and 1 are allowed. If the W option is not used, all sites will be
analyzed. If the Weights option is used, there must be a W in the first
line of the input file.

## OUTPUT FORMAT

The output starts by giving the number of species, and the number of sites. If
the default condition is used instead of the A option the program states that
it is assuming that sites absent in all species have been omitted. The value
of the site length (6 bases, for example) is also given.

If option 2 (print out data) has been selected,
there then follow the restriction site sequences, printed in
groups of ten sites. The trees found are printed as an unrooted
tree topology (possibly rooted by outgroup if so requested). The
internal nodes are numbered arbitrarily for the sake of
identification. The number of trees evaluated so far and the log
likelihood of the tree are also given.

A table is printed
showing the length of each tree segment, as well as (very) rough confidence
limits on the length. As with Dnaml, if a confidence limit is
negative, this indicates that rearrangement of the tree in that region
is not excluded, while if both limits are positive, rearrangement is
still not necessarily excluded because the variance calculation on which
the confidence limits are based results in an underestimate, which makes
the confidence limits too narrow.

In addition to the confidence limits,
the program performs a crude Likelihood Ratio Test (LRT) for each
branch of the tree. The program computes the ratio of likelihoods with and
without this branch length forced to zero length. This done by comparing the
likelihoods changing only that branch length. A truly correct LRT would
force that branch length to zero and also allow the other branch lengths to
adjust to that. The result would be a likelihood ratio closer to 1. Therefore
the present LRT will err on the side of being too significant.

One should also
realize that if you are looking not at a previously-chosen branch but at all
branches, that you are seeing the results of multiple tests. With 20 tests,
one is expected to reach significance at the P = .05 level purely by
chance. You should therefore use a much more conservative significance level,
such as .05 divided by the number of tests. The significance of these tests
is shown by printing asterisks next to
the confidence interval on each branch length. It is important to keep
in mind that both the confidence limits and the tests
are very rough and approximate, and probably indicate more significance than
they should. Nevertheless, maximum likelihood is one of the few methods that
can give you any indication of its own error; most other methods simply fail to
warn the user that there is any error! (In fact, whole philosophical schools
of taxonomists exist whose main point seems to be that there isn't any
error, that the "most parsimonious" tree is the best tree by definition and
that's that).

The log likelihood printed out with the final tree can be used to perform
various likelihood ratio tests. Remember that testing one tree topology
against another is not a simple matter, because two different tree topologies
are not hypotheses that are
nested one within the other. If the trees differ by only one branch
swap, it seems to be conservative to test the difference between their
likelihoods with one degree of freedom, but other than that little is known and
more work on this is needed.

If the U (User Tree) option is used and more than one tree is supplied,
and the program is not told to assume autocorrelation between the
rates at different sites, the
program also performs a statistical test of each of these trees against the
one with highest likelihood. If there are two user trees, the test
done is one which is due to Kishino and Hasegawa (1989), a version
of a test originally introduced by Templeton (1983). In this
implementation it uses the mean and variance of
log-likelihood differences between trees, taken across sites. If the two
trees' means are more than 1.96 standard deviations different
then the trees are
declared significantly different. This use of the empirical variance of
log-likelihood differences is more robust and nonparametric than the
classical likelihood ratio test, and may to some extent compensate for the
any lack of realism in the model underlying this program.

If there are more than two trees, the test done is an extension of
the KHT test, due to Shimodaira and Hasegawa (1999). They pointed out
that a correction for the number of trees was necessary, and they
introduced a resampling method to make this correction. In the version
used here the variances and covariances of the sum of log likelihoods across
sites are computed for all pairs of trees. To test whether the
difference between each tree and the best one is larger than could have
been expected if they all had the same expected log-likelihood,
log-likelihoods for all trees are sampled with these covariances and equal
means (Shimodaira and Hasegawa's "least favorable hypothesis"),
and a P value is computed from the fraction of times the difference between
the tree's value and the highest log-likelihood exceeds that actually
observed. Note that this sampling needs random numbers, and so the
program will prompt the user for a random number seed if one has not
already been supplied. With the two-tree KHT test no random numbers
are used.

In either the KHT or the SH test the program
prints out a table of the log-likelihoods of each tree, the differences of
each from the highest one, the variance of that quantity as determined by
the log-likelihood differences at individual sites, and a conclusion as to
whether that tree is or is not significantly worse than the best one.

The branch lengths printed out are scaled in terms of expected numbers of
base substitutions, not counting replacements of a base by itself. Of course,
when a branch is twice as long this does not mean that there will be twice as
much net change expected along it, since some of the changes occur in the same
site and overlie or even reverse each other. Confidence limits on the branch
lengths are also given. Of course a negative value of the branch length is
meaningless, and a confidence limit overlapping zero simply means that the
branch length is not necessarily significantly different from zero. Because of
limitations of the numerical algorithm, branch length estimates of zero will
often print out as small numbers such as 0.00001. If you see a branch length
that small, it is really estimated to be of zero length.

Another possible source of confusion is the existence of negative values for
the log likelihood. This is not really a problem; the log likelihood is not a
probability but the logarithm of a probability, and since probabilities never
exceed 1.0 this logarithm will typically be negative. The log likelihood is
maximized by being made more positive: -30.23 is worse than -29.14. The log
likelihood will not always be negative since a combinatorial constant has been
left out of the expression for the likelihood. This does not affect the tree
found or the likelihood ratios (or log likelihood differences) between trees.

## THE ALGORITHM

The program uses a Newton-Raphson algorithm to update one branch length at a
time. This is faster than the EM algorithm which was described in my
paper on restriction sites maximum likelihood (Felsenstein, 1992). The
likelihood that is being
maximized is the same one used by Smouse and Li (1987) extended for multiple
species.
moving down on the likelihood surface. You may have to "tune" the value of
extrapol to suit your data.

## PROGRAM CONSTANTS

The constants include "maxcutter" (set in phylip.h),
the maximum length of an enzyme
recognition site. The memory used by the program will be approximately
proportional to this value, which is 8 in the distribution copy.
The program also uses constants
"iterations" and "smoothings", and decreasing "epsilon". Reducing
"iterations" and "smoothings" or increasing "epsilon"
will result in faster execution but a worse result. These values will
not usually have to be changed.

The program spends most of its time doing real arithmetic. The algorithm, with
separate and independent computations
occurring at each site, lends itself readily to parallel processing.

A feature of the algorithm is that it saves time by recognizing sites at which
the pattern of presence/absence is the same, and does that computation only
once. Thus if we have only four species but a large number of sites, there are
only about (ignoring ambiguous bases) 16 different patterns of presence/absence
(2 x 2 x 2 x 2) that can occur. The program automatically counts
occurrences of each and does the computation for each pattern only once, so
that it only needs to do as much computation as would be needed with at most
16 sites, even though the number of sites is actually much larger. Thus
the program will run very effectively with few species and many sites.

## PAST AND FUTURE OF THE PROGRAM

This program was developed by modifying Dnaml version 3.1 and also adding
some of the modifications that were added to Dnaml version 3.2, with which
it shares many of its data structures and much of its strategy. Version
3.6 changed from EM iterations of branch lengths, which involved arbitrary
extrapolation factors, to the Newton-Raphson algorithm, which improved the
speed of the program (though only from "very slow" to "slow").

There are a number of obvious directions in which the program needs to be
modified in the future. Extension to allow for different rates of transition
and transversion is straightforward, but would slow down the program
considerably, as I have mentioned above. I have not included in the program
any provision for saving and printing out
multiple trees tied for highest likelihood, in part because an exact tie is
unlikely.

---

### TEST DATA SET

|  |
| --- |
| ```    5   13   2 Alpha     ++-+-++--+++- Beta      ++++--+--+++- Gamma     -+--+-++-+-++ Delta     ++-+----++--- Epsilon   ++++----++--- ``` |

---

### CONTENTS OF OUTPUT FILE (if all numerical options are on)

|  |
| --- |
| ``` Restriction site Maximum Likelihood method, version 3.66     5 Species,   13 Sites,   2 Enzymes    Recognition sequences all 6 bases long  Sites absent from all species are assumed to have been omitted   Name            Sites ----            -----  Alpha        ++-+-++--+ ++- Beta         ++++--+--+ ++- Gamma        -+--+-++-+ -++ Delta        ++-+----++ --- Epsilon      ++++----++ ---     +Beta         |     |      +Epsilon      |  +---3     2--1   +Delta        |  |     |  +-----Gamma        |     +Alpha        remember: this is an unrooted tree!  Ln Likelihood =   -40.48038     Between        And            Length      Approx. Confidence Limits  -------        ---            ------      ------- ---------- ------    2          Beta            0.00050     (     zero,    infinity)    2             1            0.00050     (     zero,     0.04040)    1             3            0.05860     (     zero,     0.12697) **    3          Epsilon         0.00100     (     zero,    infinity)    3          Delta           0.01463     (     zero,     0.04498) **    1          Gamma           0.11421     (  0.01684,     0.22614) **    2          Alpha           0.02471     (     zero,     0.06173) **       *  = significantly positive, P < 0.05      ** = significantly positive, P < 0.01 ``` |
