# Supplementary material for: Probabilistic Phylogenetic Inference with Insertions and Deletions
Source: PLoS Comput Biol. 2008 Sep 19;4(9):e1000172. doi: 10.1371/journal.pcbi.1000172 (PMC2527138; doi:10.1371/journal.pcbi.1000172)
Supplement: Dataset S1 — Supplemental Material (24.89 MB GZ) [file pcbi.1000172.s001.gz › erate-supplement-R2/src/phylip3.66-erate/doc/sequence.html]

sequence


version 3.66

# Molecular Sequence Programs

(c) Copyright 1986-2004 by The University of
Washington. Written by Joseph Felsenstein. Permission is granted to copy
this document provided that no fee is charged for it and that this copyright
notice is not removed.

These programs estimate phylogenies from protein
sequence or nucleic acid sequence data. Protpars uses a parsimony method
intermediate between Eck and Dayhoff's
method (1966) of allowing transitions between all amino acids and counting
those, and Fitch's (1971) method of counting the number of nucleotide changes
that would be needed to evolve the protein sequence. Dnapars uses the
parsimony method allowing changes between all bases
and counting the number of those. Dnamove is an interactive parsimony
program allowing the user to rearrange trees by hand and see where
characters states change. Dnapenny
uses the branch-and-bound method to search for all most
parsimonious trees in the nucleic acid sequence case. Dnacomp
adapts to nucleotide sequences the compatibility (largest clique)
approach. Dnainvar does not directly estimate a phylogeny, but computes Lake's
(1987) and Cavender's (Cavender and Felsenstein, 1987) phylogenetic invariants,
which are quantities whose values depend on the phylogeny. Dnaml does a
maximum likelihood estimate of the phylogeny (Felsenstein, 1981a). Dnamlk
is similar to Dnaml but assumes a molecular clock. Dnadist
computes distance measures between pairs of species from nucleotide sequences,
distances that can then be used by the distance matrix programs Fitch and
Kitsch. Restml does a maximum likelihood estimate from restriction
sites data. Seqboot allows you to read in a data set and then produce
multiple data sets from it by bootstrapping, delete-half jackknifing, or
by permuting within sites. This
then allows most of these methods to be bootstrapped or jackknifed, and
for the Permutation Tail Probability Test of Archie (1989) and Faith and
Cranston (1991) to be carried out.

The input and output format for Restml is described in
its document files. In general its input format is similar to
those described here, except that the one-letter codes for restriction sites
is specific to that program and is described in that document file. Since
the input formats for the eight DNA sequence and two protein sequence
programs apply to more than one program, they are described here. Their
input formats are standard, making use of the IUPAC standards.
.sp 2
.ce
INTERLEAVED AND SEQUENTIAL FORMATS

The sequences can continue over multiple lines; when this is done the
sequences must be either in "interleaved" format, similar to the
output of alignment programs, or "sequential" format. These are
described in the main document file. In sequential format all
of one sequence is given, possibly on multiple lines, before the next starts.
In interleaved format the first part of the file should contain the first
part of each of the sequences, then possibly a line containing nothing
but a carriage-return character, then the second part of each sequence,
and so on. Only the first parts of the sequences should be preceded by
names. Here is a hypothetical example of interleaved format:

|  |
| --- |
| ```   5    42 Turkey    AAGCTNGGGC ATTTCAGGGT Salmo gairAAGCCTTGGC AGTGCAGGGT H. SapiensACCGGTTGGC CGTTCAGGGT Chimp     AAACCCTTGC CGTTACGCTT Gorilla   AAACCCTTGC CGGTACGCTT  GAGCCCGGGC AATACAGGGT AT GAGCCGTGGC CGGGCACGGT AT ACAGGTTGGC CGTTCAGGGT AA AAACCGAGGC CGGGACACTC AT AAACCATTGC CGGTACGCTT AA ``` |

while in sequential format the same sequences would be:

|  |
| --- |
| ```   5    42 Turkey    AAGCTNGGGC ATTTCAGGGT GAGCCCGGGC AATACAGGGT AT Salmo gairAAGCCTTGGC AGTGCAGGGT GAGCCGTGGC CGGGCACGGT AT H. SapiensACCGGTTGGC CGTTCAGGGT ACAGGTTGGC CGTTCAGGGT AA Chimp     AAACCCTTGC CGTTACGCTT AAACCGAGGC CGGGACACTC AT Gorilla   AAACCCTTGC CGGTACGCTT AAACCATTGC CGGTACGCTT AA ``` |

Note, of course, that a portion of a sequence like this:

300 AAGCGTGAAC GTTGTACTAA TRCAG

is perfectly legal, assuming that the species name has gone before, and is
filled out to full length by blanks. The above
digits and blanks will be ignored, the sequence being taken as starting
at the first base symbol (in this case an A). This should enable you to
use output from many multiple-sequence alignment programs with only
minimal editing.

In interleaved format
the present versions of the programs may sometimes have difficulties with the
blank lines between groups of lines, and if so you might want to retype
those lines, making sure that they have only a carriage-return and no blank
characters on them, or you may perhaps have to eliminate them. The symptoms
of this problem are that the programs complain that the sequences are not
properly aligned, and you can find no other cause for this complaint.

## INPUT FOR THE DNA SEQUENCE PROGRAMS

The input format for the DNA sequence programs is
standard: the data have A's, G's, C's and T's (or U's). The first line of the
input file contains the number of species and the number of sites. As
with the other programs, options information may follow this. Following this,
each species starts on a new line. The first 10
characters of that line are the species name. There then follows
the base sequence of that species, each character
being one of the letters A, B, C, D, G, H, K, M, N, O, R, S, T, U, V,
W, X, Y, ?, or - (a period was also previously allowed but it is no longer
allowed, because it sometimes is used in different senses in other
programs). Blanks will be ignored, and so will numerical
digits. This allows GENBANK and EMBL sequence entries to be read with
minimum editing.

These characters can be either upper or lower case. The algorithms
convert all input characters to upper case (which is how they
are treated). The characters constitute the IUPAC (IUB) nucleic acid code
plus some slight
extensions. They enable input of nucleic acid sequences taking full account
of any ambiguities in the sequence.

|  |  |  |  |
| --- | --- | --- | --- |
| **Symbol** **Meaning** |  | | |
|  |  |
| A Adenine |  | | |
| G Guanine |  | | |
| C Cytosine |  | | |
| T Thymine |  | | |
| U Uracil |  | | |
| Y pYrimidine (C or T) | | | | |
| R puRine (A or G) | | | | |
| W "Weak" (A or T) | | | | |
| S "Strong" (C or G) | | | | |
| K "Keto" (T or G) | | | | |
| M "aMino" (C or A) | | | | |
| B not A (C or G or T) | | | | |
| D not C (A or G or T) | | | | |
| H not G (A or C or T) | | | | |
| V not T (A or C or G) | | | | |
| X,N,? unknown (A or C or G or T) | | | | |
| O deletion |  | | |
| - deletion |  | | |

## INPUT FOR THE PROTEIN SEQUENCE PROGRAMS

The input for the protein sequence programs is fairly standard. The first
line contains the
number of species and the number of amino acid positions (counting any
stop codons that you want to include). These are followed on the same line
by the options. The only options which
need information in the input file are U (User Tree) and W (Weights). They are
as described in the main documentation file. If the W (Weights) option is
used there must be a W in the first line of the input file.

Next come the species data. Each
sequence starts on a new line, has a ten-character species name
that must be blank-filled to be of that length, followed immediately
by the species data in the one-letter code. The sequences must either
be in the "interleaved" or "sequential" formats. The I option
selects between them. The sequences can have internal
blanks in the sequence but there must be no extra blanks at the end of the
terminated line. Note that a blank is not a valid symbol for a deletion.

The protein sequences are given by the one-letter code used by
the late Margaret Dayhoff's group in the Atlas of Protein Sequences,
and consistent with the IUB standard abbreviations.
In the present version it is:

|  |  |
| --- | --- |
| **Symbol** | **Stands for** |
|  |  |
| A | ala |
| B | asx |
| C | cys |
| D | asp |
| E | glu |
| F | phe |
| G | gly |
| H | his |
| I | ileu |
| J | (not used) |
| K | lys |
| L | leu |
| M | met |
| N | asn |
| O | (not used) |
| P | pro |
| Q | gln |
| R | arg |
| S | ser |
| T | thr |
| U | (not used) |
| V | val |
| W | trp |
| X | unknown amino acid |
| Y | tyr |
| Z | glx |
| \* | nonsense (stop) |
| ? | unknown amino acid or deletion |
| - | deletion |

where "nonsense", and "unknown" mean respectively a nonsense (chain
termination) codon and an amino acid whose identity has not been
determined. The state "asx" means "either asn or asp",
and the state "glx" means "either gln or glu" and the state "deletion"
means that alignment studies indicate a deletion has happened in the
ancestry of this position, so that it is no longer present. Note that
if two polypeptide chains are being used that are of different length
owing to one terminating before the other, they can be coded as (say)

```
             HIINMA*????
             HIPNMGVWABT
```

since after the stop codon we do not definitely know that
there has been a deletion, and do not know what amino acid would
have been there. If DNA studies tell us that there is
DNA sequence in that region, then we could use "X" rather than "?". Note
that "X" means an unknown amino acid, but definitely an amino acid,
while "?" could mean either that or a deletion. Otherwise one will usually
want to use "?" after a stop codon, if one does not know what amino acid is
there. If the DNA sequence has been observed there, one probably ought to
resist putting in the amino acids that this DNA would code for, and one should
use "X" instead, because under the assumptions implicit in this either the
parsimony or the distance
methods, changes to any noncoding sequence are much easier than
changes in a coding region that change the amino acid

Here are the same one-letter codes tabulated the other way 'round:

|  |  |
| --- | --- |
| **Amino acid** | **One-letter code** |
|  |  |
| ala | A |
| arg | R |
| asn | N |
| asp | D |
| asx | B |
| cys | C |
| gln | Q |
| glu | E |
| gly | G |
| glx | Z |
| his | H |
| ileu | I |
| leu | L |
| lys | K |
| met | M |
| phe | F |
| pro | P |
| ser | S |
| thr | T |
| trp | W |
| tyr | Y |
| val | V |
| deletion | - |
| nonsense (stop) | \* |
| unknown amino acid | X |
| unknown (incl. deletion) | ? |

## THE OPTIONS

The programs allow options chosen from their menus. Many of these are as described in the
main documentation file, particularly the options J, O, U, T, W,
and Y. (Although T has a different meaning in the programs Dnaml and
Dnadist than in the others).

The U option indicates that
user-defined trees are provided at the end of the input file. This
happens in the usual way, except that for Protpars, Dnapars, Dnacomp, and
Dnamlk, the trees must be strictly
bifurcating, containing only two-way splits, e. g.: ((A,B),(C,(D,E)));. For
Dnaml and Restml it must have a trifurcation at its base,
e. g.: ((A,B),C,(D,E));. The
root of the tree may in those cases be placed arbitrarily, since the trees
needed are actually unrooted, though they look different when printed out. The
program Retree should enable you to reroot the trees without having to
hand-edit or retype them. For
Dnamove the U option is not available (although
there is an equivalent feature which uses rooted user trees).

A feature of the nucleotide sequence programs other than Dnamove
is that they save time and computer memory space by recognizing sites
at which the pattern of bases is the same, and doing their computation only
once. Thus if we have only four species but a large number of sites, there
are (ignoring ambiguous bases) only about 256 different patterns of
nucleotides (4 x 4 x 4 x 4) that can occur. The programs automatically
count how many occurrences there are of each and then only needs to do as much
computation as would be
needed with 256 sites, even though the number of sites is actually much
larger. If there are ambiguities (such as Y or R nucleotides), these are also
handled correctly, and do not cause trouble. The programs store the full
sequences but reserve other space for bookkeeping only for the distinct
patterns. This saves space. Thus the programs will run very effectively
with few species and many sites. On larger numbers of species,
if rates of evolution are small, many of the sites will be invariant
(such as having all A's) and thus will mostly have one of four patterns. The
programs will in this way automatically avoid doing duplicate
computations for such sites.
