# Supplementary material for: Probabilistic Phylogenetic Inference with Insertions and Deletions
Source: PLoS Comput Biol. 2008 Sep 19;4(9):e1000172. doi: 10.1371/journal.pcbi.1000172 (PMC2527138; doi:10.1371/journal.pcbi.1000172)
Supplement: Dataset S1 — Supplemental Material (24.89 MB GZ) [file pcbi.1000172.s001.gz › erate-supplement-R2/src/phylip3.66-erate/doc/protpars.html]

protpars


version 3.66

# Protpars -- Protein Sequence Parsimony Method

© Copyright 1986-2006 by the University of
Washington. Written by Joseph Felsenstein. Permission is granted to copy
this document provided that no fee is charged for it and that this copyright
notice is not removed.

This program infers an unrooted phylogeny from protein sequences, using a
new method intermediate between the approaches of Eck and Dayhoff (1966) and
Fitch (1971). Eck and Dayhoff (1966) allowed any amino acid to change to
any other, and counted the number of such changes needed to evolve the
protein sequences on each given phylogeny. This has the problem that it
allows replacements which are not consistent with the genetic code, counting
them equally with replacements that are consistent. Fitch, on the other hand,
counted the minimum number of nucleotide substitutions that would be
needed to achieve the given protein sequences. This counts silent
changes equally with those that change the amino acid.

The present method insists that any changes of amino acid be consistent
with the genetic code so that, for example, lysine is allowed to change
to methionine but not to proline. However, changes between two amino acids
via a third are allowed and counted as two changes if each of the two
replacements is individually allowed. This sometimes allows changes that
at first sight you would think should be outlawed. Thus we can change from
phenylalanine to glutamine via leucine in two steps
total. Consulting the genetic code, you will find that there is a leucine
codon one step away from a phenylalanine codon, and a leucine codon one
step away from glutamine. But they are not the same leucine codon. It
actually takes three base substitutions to get from either of the
phenylalanine codons TTT and TTC to either of the glutamine codons
CAA or CAG. Why then does this program count only two? The answer
is that recent DNA sequence comparisons seem to show that synonymous
changes are considerably faster and easier than ones that change the
amino acid. We are assuming that, in effect, synonymous changes occur
so much more readily that they need not be counted. Thus, in the chain
of changes TTT (Phe) --> CTT (Leu) --> CTA (Leu) --> CAA (Glu), the middle
one is not counted because it does not change the amino acid (leucine).

To maintain consistency with the genetic code, it is necessary for the
program internally to treat serine as two separate states (ser1 and ser2)
since the two groups of serine codons are not adjacent in the
code. Changes to the state "deletion" are counted as three steps to prevent the
algorithm from assuming unnecessary deletions. The state "unknown" is
simply taken to mean that the amino acid, which has not been determined,
will in each part of a tree that is evaluated be assumed be whichever one
causes the fewest steps.

The assumptions of this method (which has not been described in the
literature), are thus something like this:

1. Change in different sites is independent.- Change in different lineages is independent.- The probability of a base substitution that changes the amino
       acid sequence is small over the lengths of time involved in
       a branch of the phylogeny.- The expected amounts of change in different branches of the phylogeny
         do not vary by so much that two changes in a high-rate branch
         are more probable than one change in a low-rate branch.- The expected amounts of change do not vary enough among sites that two
           changes in one site are more probable than one change in another.- The probability of a base change that is synonymous is much higher
             than the probability of a change that is not synonymous.

That these are the assumptions of parsimony methods has been documented
in a series of papers of mine: (1973a, 1978b, 1979, 1981b, 1983b, 1988b). For
an opposing view arguing that the parsimony methods make no substantive
assumptions such as these, see the works by Farris (1983) and Sober (1983a,
1983b, 1988), but also read the exchange between Felsenstein and Sober (1986).

The input for the program is fairly standard. The first line contains the
number of species and the number of amino acid positions (counting any
stop codons that you want to include).

Next come the species data. Each
sequence starts on a new line, has a ten-character species name
that must be blank-filled to be of that length, followed immediately
by the species data in the one-letter code. The sequences must either
be in the "interleaved" or "sequential" formats
described in the Molecular Sequence Programs document. The I option
selects between them. The sequences can have internal
blanks in the sequence but there must be no extra blanks at the end of the
terminated line. Note that a blank is not a valid symbol for a deletion.

The protein sequences are given by the one-letter code used by
described in the Molecular Sequence Programs documentation file. Note that
if two polypeptide chains are being used that are of different length
owing to one terminating before the other, they should be coded as (say)

```
             HIINMA*????
             HIPNMGVWABT
```

since after the stop codon we do not definitely know that
there has been a deletion, and do not know what amino acid would
have been there. If DNA studies tell us that there is
DNA sequence in that region, then we could use "X" rather than "?". Note
that "X" means an unknown amino acid, but definitely an amino acid,
while "?" could mean either that or a deletion. The distinction is often
significant in regions where there are deletions: one may want to encode
a six-base deletion as "-?????" since that way the program will only count
one deletion, not six deletion events, when the deletion arises. However,
if there are overlapping deletions it may not be so easy to know what
coding is correct.

One will usually want to
use "?" after a stop codon, if one does not know what amino acid is there. If
the DNA sequence has been observed there, one probably ought to resist
putting in the amino acids that this DNA would code for, and one should use
"X" instead, because under the assumptions implicit in this parsimony
method, changes to any noncoding sequence are much easier than
changes in a coding region that change the amino acid, so that they
shouldn't be counted anyway!

The form of this information
is the standard one described in the main documentation file. For the U option
the tree
provided must be a rooted bifurcating tree, with the root placed anywhere
you want, since that root placement does not affect anything.

The options are selected using an interactive menu. The menu looks like this:

|  |
| --- |
| ``` Protein parsimony algorithm, version 3.6  Setting for this run:   U                 Search for best tree?  Yes   J   Randomize input order of sequences?  No. Use input order   O                        Outgroup root?  No, use as outgroup species  1   T              Use Threshold parsimony?  No, use ordinary parsimony   C               Use which genetic code?  Universal   W                       Sites weighted?  No   M           Analyze multiple data sets?  No   I          Input sequences interleaved?  Yes   0   Terminal type (IBM PC, ANSI, none)?  ANSI   1    Print out the data at start of run  No   2  Print indications of progress of run  Yes   3                        Print out tree  Yes   4          Print out steps in each site  No   5  Print sequences at all nodes of tree  No   6       Write out trees onto tree file?  Yes  Are these settings correct? (type Y or the letter for one to change) ``` |

The user either types "Y" (followed, of course, by a carriage-return)
if the settings shown are to be accepted, or the letter or digit corresponding
to an option that is to be changed.

The options U, J, O, T, W, M, and 0 are the usual ones. They are described in
the main documentation file of this package. Option I is the same as in
other molecular sequence programs and is described in the documentation file
for the sequence programs. Option C allows the user to select among various
nuclear and mitochondrial genetic codes. There is no provision for coping
with data where different genetic codes have been used in different
organisms.

In the U (User tree) option, the trees should
not be preceded by a line with the number of trees on it.

Output is standard: if option 1 is toggled on, the data is printed out,
with the convention that "." means "the same as in the first species".
Then comes a list of equally parsimonious trees, and (if option 2 is
toggled on) a table of the
number of changes of state required in each position. If option 5 is toggled
on, a table is printed
out after each tree, showing for each branch whether there are known to be
changes in the branch, and what the states are inferred to have been at the
top end of the branch. This is a reconstruction of the ancestral sequences
in the tree. If you choose option 5, a menu item "." appears which gives you
the opportunity to turn off dot-differencing so that complete ancestral
sequences are shown. If the inferred state is a "?" there will be multiple
equally-parsimonious assignments of states; the user must work these out for
themselves by hand. If option 6 is left in its default state the trees
found will be written to a tree file, so that they are available to be used
in other programs. If the program finds multiple
trees tied for best, all of these are written out onto the output tree
file. Each is followed by a numerical weight in square brackets (such as
[0.25000]). This is needed when we use the trees to make a consensus
tree of the results of bootstrapping or jackknifing, to avoid overrepresenting
replicates that find many tied trees.

If the U (User Tree) option is used and more than one tree is supplied, the
program also performs a statistical test of each of these trees against the
best tree. This test, which is a version of the test proposed by
Alan Templeton (1983) and evaluated in a test case by me (1985a). It is
closely parallel to a test using log likelihood differences
due to Kishino and Hasegawa (1989), and uses the mean
and variance of
step differences between trees, taken across positions. If the mean
is more than 1.96 standard deviations different then the trees are declared
significantly different. The program
prints out a table of the steps for each tree, the differences of
each from the best one, the variance of that quantity as determined by
the step differences at individual positions, and a conclusion as to
whether that tree is or is not significantly worse than the best one.

The program is derived from Mix but has had some rather elaborate
bookkeeping using sets of bits installed. It is not a very fast
program but is speeded up substantially over version 3.2.

---

### TEST DATA SET

|  |
| --- |
| ```      5    10 Alpha     ABCDEFGHIK Beta      AB--EFGHIK Gamma     ?BCDSFG*?? Delta     CIKDEFGHIK Epsilon   DIKDEFGHIK ``` |

---

### CONTENTS OF OUTPUT FILE (with all numerical options on)

|  |
| --- |
| ``` Protein parsimony algorithm, version 3.66   5 species,  10  sites   Name          Sequences ----          ---------  Alpha        ABCDEFGHIK  Beta         ..--......  Gamma        ?...S..*??  Delta        CIK.......  Epsilon      DIK.......         3 trees in all found        +--------Gamma           !     +--2     +--Epsilon      !  !  +--4     !  +--3  +--Delta        1     !     !     +-----Beta         !     +-----------Alpha         remember: this is an unrooted tree!   requires a total of     16.000  steps in each position:          0   1   2   3   4   5   6   7   8   9      *-----------------------------------------     0!       3   1   5   3   2   0   0   2   0    10!   0                                      From    To     Any Steps?    State at upper node                              ( . means same as in the node below it on tree)            1                ANCDEFGHIK    1      2         no     ..........    2   Gamma        yes    ?B..S..*??    2      3         yes    ..?.......    3      4         yes    ?IK.......    4   Epsilon     maybe   D.........    4   Delta        yes    C.........    3   Beta         yes    .B--......    1   Alpha       maybe   .B........               +--Epsilon            +--4        +--3  +--Delta           !  !     +--2  +-----Gamma        !  !     1  +--------Beta         !     +-----------Alpha         remember: this is an unrooted tree!   requires a total of     16.000  steps in each position:          0   1   2   3   4   5   6   7   8   9      *-----------------------------------------     0!       3   1   5   3   2   0   0   2   0    10!   0                                      From    To     Any Steps?    State at upper node                              ( . means same as in the node below it on tree)            1                ANCDEFGHIK    1      2         no     ..........    2      3        maybe   ?.........    3      4         yes    .IK.......    4   Epsilon     maybe   D.........    4   Delta        yes    C.........    3   Gamma        yes    ?B..S..*??    2   Beta         yes    .B--......    1   Alpha       maybe   .B........               +--Epsilon         +-----4        !     +--Delta        +--3     !  !     +--Gamma        1  +-----2     !        +--Beta         !     +-----------Alpha         remember: this is an unrooted tree!   requires a total of     16.000  steps in each position:          0   1   2   3   4   5   6   7   8   9      *-----------------------------------------     0!       3   1   5   3   2   0   0   2   0    10!   0                                      From    To     Any Steps?    State at upper node                              ( . means same as in the node below it on tree)            1                ANCDEFGHIK    1      3         no     ..........    3      4         yes    ?IK.......    4   Epsilon     maybe   D.........    4   Delta        yes    C.........    3      2         no     ..........    2   Gamma        yes    ?B..S..*??    2   Beta         yes    .B--......    1   Alpha       maybe   .B........ ``` |
