# Supplementary material for: Probabilistic Phylogenetic Inference with Insertions and Deletions
Source: PLoS Comput Biol. 2008 Sep 19;4(9):e1000172. doi: 10.1371/journal.pcbi.1000172 (PMC2527138; doi:10.1371/journal.pcbi.1000172)
Supplement: Dataset S1 — Supplemental Material (24.89 MB GZ) [file pcbi.1000172.s001.gz › erate-supplement-R2/src/phylip3.66-erate/phylip.html]

phylip


v3.6

# PHYLIP programs and documentation

PHYLIP, the PHYLogeny Inference Package, consists of 35 programs. There are
documentation files for each program, in the form of web pages in HTML 3.2.
There are also documentation web pages for each group of programs, and a
main documentation file that is the basic introduction to the package.
Before running any of the programs you should read it.

Below you will find a list of the programs and the documentation files.
The names of the documentation files are highlighted as links that will
take you to those documentation files.

## Introduction to PHYLIP

|  |
| --- |
| main documentation file |

## Molecular sequence methods

|  |  |
| --- | --- |
|  | molecular sequence programs documentation file |
| **protpars** | protein parsimony documentation file |
| **dnapars** | DNA sequence parsimony documentation file |
| **dnapenny** | DNA parsimony branch and bound documentation file |
| **dnamove** | interactive DNA parsimony documentation file |
| **dnacomp** | DNA compatibility documentation file |
| **dnaml** | DNA maximum likelihood documentation file |
| **dnamlk** | DNA maximum likelihood with clock documentation file |
| **proml** | Protein sequence maximum likelihood documentation file |
| **promlk** | Protein sequence maximum likelihood with clock documentation file |
| **dnainvar** | DNA invariants documentation file |
| **dnadist** | DNA distance documentation file |
| **protdist** | Protein sequence distance documentation file |
| **restdist** | Restriction sites and fragments distances documentation file |
| **restml** | Restriction sites maximum likelihood documentation file |
| **seqboot** | Bootstrapping/Jackknifing documentation file |

## Distance matrix methods

|  |  |
| --- | --- |
|  | Distance matrix programs documentation file |
| **fitch** | Fitch-Margoliash distance matrix method documentation file |
| **kitsch** | Fitch-Margoliash distance matrix with clock documentation file |
| **neighbor** | Neighbor-Joining and UPGMA method documentation file |

## Gene frequencies and continuous characters

|  |  |
| --- | --- |
|  | Continuous characters and gene frequencies documentation file |
| **contml** | Maximum likelihood continuous characters and gene frequencies documentation file |
| **contrast** | Contrast method documentation file |
| **gendist** | Genetic distance documentation file |

## Discrete characters methods

|  |  |
| --- | --- |
|  | Discrete characters methods documentation file |
| **pars** | Unordered multistate parsimony documentation file |
| **mix** | Mixed method parsimony documentation file |
| **penny** | Branch and bound mixed method parsimony documentation file |
| **move** | Interactive mixed method parsimony documentation file |
| **dollop** | Dollo and polymorphism parsimony documentation file |
| **dolpenny** | Dollo and polymorphism branch and bound parsimony documentation file |
| **dolmove** | Dollo and polymorphism interactive parsimony documentation file |
| **clique** | 0/1 characters compatibility method documentation file |
| **factor** | Character recoding program documentation file |

## Tree drawing, consensus, tree editing, tree distances

|  |  |
| --- | --- |
|  | Tree drawing programs documentation file |
| **drawgram** | Rooted tree drawing program documentation file |
| **drawtree** | Unrooted tree drawing program documentation file |
|  |  |
| **consense** | Consensus tree program documentation file |
| **treedist** | Tree distance program documentation file |
| **retree** | interactive tree rearrangement program documentation file |
