# Supplementary material for: Probabilistic Phylogenetic Inference with Insertions and Deletions
Source: PLoS Comput Biol. 2008 Sep 19;4(9):e1000172. doi: 10.1371/journal.pcbi.1000172 (PMC2527138; doi:10.1371/journal.pcbi.1000172)
Supplement: Text S1 — Appendix 1 (0.16 MB PDF) [file pcbi.1000172.s002.pdf]

Appendix 1: Derivation of the Markov process for a gap-augmented rate matrix

Here we present the differential equations that define a conditional matrix for an gap-augmented rate matrix R^{ε}. We also describe a particular quasi-reversible analytical solution for the extended conditional probabilities which applies for any arbitrary underlying reversible substitutions-only rate matrix R.

For an alphabet of K residues, those probabilistic models are defined by a $K \times K$ rate matrix R such that the matrix of conditional probabilities $Q_t(i,j) \equiv P(j|i,t)$ is given by

$$Q_t \ = \ e^{t\,R} \ = \sum_{l=0}^{\infty} \frac{(tR)^l}{l!}. \tag{32}$$

A well behaved rate matrix R has eigenvalues of the form $e_0 = 0$ and $-e_a < 0$, for $1 \leq a \leq A$ (where A is the number of non-degenerate eigenvalues other than zero), which is at most $K-1$. Assuming that the zero eigenvalue is not degenerate, the model has a saturation behavior given by

$$\lim_{t\to\infty} Q_t = \begin{pmatrix} \pi_1 & \dots & \pi_K \\ \vdots & \vdots & \vdots \\ \pi_1 & \dots & \pi_K \end{pmatrix} = \begin{pmatrix} 1 \\ \vdots \\ 1 \end{pmatrix} \left(\pi^1, \dots, \pi^K\right) = u\,\pi^T, \tag{33}$$

where the distribution π are called the equilibrium (or saturation) probabilities, and $u^T = (1, \dots, 1)$ is the unitary vector. In addition, if the rate R is reversible $[\pi_i R(i,j) = \pi_j R(j,i)]$, the conditional probabilities also satisfy the equivalent requirement of time reversibility,

$$\pi_i Q_t(i,j) = \pi_j Q_t(j,i), \quad \forall t. \tag{34}$$

For every diagonalizable rate matrix R, there is a similarity transformation such that for an invertible matrix U [86]

$$R \ = \ U\,R_{\text{diag}}\,U^{-1}. \tag{35}$$

Using the similarity transformation, one can cast Q_t into the form,

$$Q_t \ = \ U\,e^{t\,R_{\text{diag}}}\,U^{-1}. \tag{36}$$

For every eigenvalue of R, we can construct a $K \times K$ matrix O_a (for $0 \leq a \leq A$), such that if the eigenvalue is at position (k_a, k_a) of R_{diag}, then

$$O_a(i,j) = U(i,k_a)\,U^{-1}(k_a,j). \tag{37}$$

Using the O_a matrices, one can rewrite,

$$R \quad = \quad -\sum_{a=1}^{A} e_a\,O_a, \tag{38}$$

$$Q_t \;=\; O_0 + \sum_{a=1}^{A} O_a\, e^{-e_a t}. \tag{39}$$

Since O_0 controls the behavior at time infinity given by Eq. (33), then one has $O_0(i,j) = \pi_j$. Thus, Q_t can be cast into the form,

$$Q_t \;=\; u\pi^T + \sum_{a=1}^{A} O_a\, e^{-e_a t}. \tag{40}$$

The advantage of this representation of Q_t over the one given in terms of the matrix U in Eq. (36) is that the O_a matrices, unlike the similarity matrix U, are independent of the particular coordinate system used for $R_{\rm diag}$.

The O_a matrices consist of $K \times K$ real numbers that satisfy the conditions

$$\sum_{a=1}^{A} O_a + u\pi^T \;=\; U\,U^{-1} = I, \tag{41}$$

$$RO_a \;=\; -e_a O_a. \tag{42}$$

In addition, if R is reversible they also satisfy the conditions

$$\pi_i O_a(i,j) = \pi_j O_a(j,i). \tag{43}$$

For a general rate matrix R, the eigenvalues $\{-e_a\}$ have to be calculated numerically, as well as the $\{O_a\}$ matrices using Eqs. (41,42). In some simple cases, both the eigenvalues and the matrices $\{O_a\}$ can be calculated analytically.

The extended $(K+1) \times (K+1)$ rate matrix R^ε considered in this work depends on a rate of deletion $\mu \geq 0$ and a rate of insertion $\lambda \geq 0$ and is given by

$$R^\varepsilon = \left(\begin{array}{c|c} R - \mu\, I & \begin{array}{c} \mu \\ \vdots \\ \mu \end{array} \\ \hline \lambda p_1 \;\ldots\; \lambda p_K & -\lambda \end{array} \right), \tag{44}$$

where I is the $K \times K$ identity matrix, and p is the distribution of inserted residues ($\sum_{i=1}^{K} p_i = 1$). This extended rate matrix R^ε has the property that each row adds up to zero. The extended conditional probabilities are defined by

$$Q_t^\varepsilon \;\equiv\; e^{t\,R^\varepsilon} \;=\sum_{l=0}^{\infty} \frac{(tR^\varepsilon)^l}{l!}. \tag{45}$$

Generally, the extended conditionals Q_t^ε can be cast to the form,

$$Q_t^\varepsilon = \left(\begin{array}{c|c} & \gamma_t \\ M_t & \vdots \\ & \gamma_t \\ \hline \xi_t^1 \;\ldots\; \xi_t^K & \sigma_t \end{array} \right), \tag{46}$$

with the conditions $\sigma_t + \sum_{j=1}^K \xi_t^j = 1$ and $\gamma_t + \sum_{j=1}^K M_t(i,j) = 1$, for each row i. The analytic expression for Q_t^ε can be obtained by solving the linear differential equation $d\left(e^{tR^\varepsilon}\right)/dt = R^\varepsilon e^{tR^\varepsilon}$, which corresponds to

$$\begin{aligned} \dot{M}_t &= (R - \mu I) M_t + \mu u\, \xi_t^T, & (47)\\ \dot{\gamma}_t &= \mu\left(\sigma_t - \gamma_t\right), & (48)\\ \dot{\sigma}_t &= \lambda\left(\gamma_t - \sigma_t\right), & (49)\\ \dot{\xi}_t^i &= \lambda\,(p^T M_t)^i - \lambda \xi_t^i\,, & (50) \end{aligned}$$

where $\xi_t^T = (\xi_t^1, \ldots, \xi_t^K)$, and $(p^T M_t)^i = \sum_{j=1}^K p_j\, M_t(j,i)$. With the initialization conditions at $t = 0$

$$M_0 = I, \quad \gamma_0 = 0, \quad \sigma_0 = 1, \quad \xi_0^i = 0. \tag{51}$$

The linear differential equations for γ_t and σ_t are independent of the underlying substitution process and given by,

$$\begin{aligned} \gamma_t &= \frac{\mu}{\lambda + \mu}\left(1 - e^{-(\lambda+\mu)t}\right), & (52)\\ \sigma_t &= \frac{1}{\lambda + \mu}\left(\mu + \lambda e^{-(\lambda+\mu)t}\right). & (53) \end{aligned}$$

We will not attempt to solve the general case analytically, but rather the restricted case in which the underlying substitution process is reversible, and where the distribution of insertions introduced in Eq. (44) is the same as the equilibrium distribution of the substitution rate matrix (*i.e.* $p = \pi$). In that case, the reversibility condition for R together with the property $\pi_i R^\varepsilon(i,-) R^\varepsilon(-,j) = \pi_j R^\varepsilon(j,-) R^\varepsilon(-,i) = \pi_i \pi_j \lambda \mu$ guarantee that reversibility is maintained in the substitution subspace and therefore $(\pi^T M_t)^i = \pi_i (1 - \gamma_t)$. In this particular case, the equation for ξ_t^i becomes

$$\dot{\xi}_t^i + \lambda\, \xi_t^i = \lambda \pi_i (1 - \gamma_t), \tag{54}$$

which has the solution $\xi_t^i = \pi_i\, \xi_t$ for

$$\xi_t = 1 - \sigma_t = \frac{\lambda}{\lambda + \mu}\left(1 - e^{-(\lambda+\mu)t}\right). \tag{55}$$

We call this model quasi-reversible since for a reversible rate matrix R, the extended model maintains

reversibility in the substitution subspace, that is, $\pi_i M_t(i,j) = \pi_j M_t(j,i)$.

A key observation regarding obtaining the conditional probabilities associated to substitutions in the presence of insertions and substitutions (M_t) is to notice that if the eigenvalues of R are $\{0, -e_a\}$, then the eigenvalues of R^ε are given by $\{0, -(e_a+\mu), -(\lambda+\mu)\}$. One can see that the conditional substitutions that satisfy the linear non-homogeneous equation (47) are given by

$$M_t = u\pi^T \frac{\lambda}{\lambda+\mu} + \sum_a O_a\, e^{-(e_a+\mu)t} + u\pi^T \frac{\mu}{\lambda+\mu}\, e^{-(\lambda+\mu)t}, \tag{56}$$

provided that either the rate of insertions or the rate of deletion is positive. In the particular case $\lambda = \mu = 0$, then

$$M_t = u\pi^T + \sum_a O_a\, e^{-e_a t}. \tag{57}$$

In summary, for any reversible $K \times K$ substitution rate matrix R and using the stationary distribution of residues for R as the distribution of inserted residues, the solution for the extended conditional probabilities is quasi-reversible and given by

$$Q_t^\varepsilon = \left(\begin{array}{ccc|c} & & & \gamma_t \\ & M_t & & \vdots \\ & & & \gamma_t \\ \hline \xi_t \pi_1 & \ldots & \xi_t \pi_K & 1-\xi_t \end{array} \right), \tag{58}$$

where the functions γ_t, ξ_t, and the $K \times K$ matrix M_t are given by Eqs. (52), (55), and (56) respectively.

For instance, for the Jukes-Cantor model [7] with substitution rate $R(i \neq j) = \alpha$, there is only one non-zero eigenvalue, $e_1 = 4\alpha$, and the stationary probabilities are $\pi_i = \frac{1}{4}$. The matrix O_1 is given by $O_1(i,j) = \delta_{ij} - \frac{1}{4}$, where δ_{ij} is one if i and j are equal or zero otherwise. Introducing those values in Eq. (56), one obtains for the extended Jukes-Cantor model

$$M_t^{JC}(i,j) = \frac{1}{4}\frac{\lambda}{\lambda+\mu} + \left(\delta_{ij} - \frac{1}{4}\right) e^{-(4\alpha+\mu)\,t} + \frac{1}{4}\frac{\mu}{\lambda+\mu}\, e^{-(\lambda+\mu)\,t}, \tag{59}$$

such that for $\lambda = \mu = 0$ is given by the original Jukes-Cantor model

$$Q_t^{JC}(i,j) = \frac{1}{4} + \left(\delta_{ij} - \frac{1}{4}\right) e^{-4\alpha t}. \tag{60}$$

The asymptotic behavior for the quasi-reversible solution is as follows: for infinitesimally small times τ we have,

$$M_\tau \simeq I + \tau\,(R - \mu I), \tag{61}$$

$$\gamma_\tau \simeq \mu\,\tau, \tag{62}$$

$$\xi_\tau \simeq \lambda\,\tau, \tag{63}$$

$$\sigma_\tau \simeq 1 - \lambda\,\tau, \tag{64}$$

which is the expected behavior since λ and μ have been introduced as the rate of insertions and deletions respectively. The asymptotic behavior at time infinity is given by,

$$Q^{\varepsilon}_{\infty} = \left(\begin{array}{ccc|c} \frac{\lambda}{\lambda+\mu}\pi_1 & \cdots & \frac{\lambda}{\lambda+\mu}\pi_K & \frac{\mu}{\lambda+\mu} \\ \vdots & \vdots & \vdots & \vdots \\ \frac{\lambda}{\lambda+\mu}\pi_1 & \cdots & \frac{\lambda}{\lambda+\mu}\pi_K & \frac{\mu}{\lambda+\mu} \\ \hline \frac{\lambda}{\lambda+\mu}\pi_1 & \cdots & \frac{\lambda}{\lambda+\mu}\pi_K & \frac{\mu}{\lambda+\mu} \end{array} \right). \tag{65}$$

Thus, the saturation (or equilibrium) distribution for the gap-augmented rate matrix R^{ε} is given by

$$\pi^{\varepsilon}_i = \begin{cases} \frac{\lambda}{\lambda+\mu}\pi_i & \text{if} \quad \mathrm{i} \leq \mathrm{K}, \\ \frac{\mu}{\lambda+\mu} & \text{if} \quad \mathrm{i} = -. \end{cases} \tag{66}$$

For more general nonreversible substitution rate matrices, the extended conditional probabilities can always be calculated numerically using the Taylor expansion in (45).
