# Supplementary material for: Probabilistic Phylogenetic Inference with Insertions and Deletions
Source: PLoS Comput Biol. 2008 Sep 19;4(9):e1000172. doi: 10.1371/journal.pcbi.1000172 (PMC2527138; doi:10.1371/journal.pcbi.1000172)
Supplement: Text S2 — Appendix 2 (0.11 MB PDF) [file pcbi.1000172.s003.pdf]

Appendix 2: Properties of the generative model

The generative model allows us to calculate the probability that any sequence of length l generates any sequence of length $l' = s + i$ for $s \leq l$ substitutions and i insertions as

$$P_t\left(l' = s + i \mid l\right) = \frac{(l+i)!}{s!\,(l-s)!\,i!}\,(1-\xi_t)^{l+1}\,(1-\gamma_t)^s\,\gamma_t^{l-s}\,\xi_t^i, \tag{67}$$

where the conditional probability of a deletion γ_t and the conditional probability of an insertion ξ_t are given by Eqs. (6), (7) respectively. The global factor $(1-\xi_t)^{l+1}$ is required to have the correct normalization $\sum_{s=0}^{l}\sum_{i=0}^{\infty} P_t\left(s+i\,|\,l\right) = 1$.

The expected length of sequences generated starting from a sequence of length l is given by

$$\langle l'|l\rangle_t \equiv \sum_{i=0}^{\infty}\sum_{s=0}^{l}(s+i)\,P_t\left(s+i \mid l\right) \tag{68}$$

$$= l\,(1-\gamma_t) + (l+1)\,\frac{\xi_t}{1-\xi_t}. \tag{69}$$

Notice that for $\lambda = \mu = 0$, one has $\langle l'|l\rangle_t = l$, as expected.

In the limit $t = 0$, the expected length of sequence generated is l, and for small divergence times τ,

$$\langle l'|l\rangle_\tau = l\,(1-\,\mu\,\tau) + (l+1)\,\lambda\,\tau. \tag{70}$$

In the limit $t \to \infty$, then

$$\langle l'|l\rangle_\infty = l\,\frac{\lambda}{\lambda+\mu} + (l+1)\,\frac{\lambda}{\mu}. \tag{71}$$

which as one would expect is zero for $\lambda = 0$, and it becomes infinity for $\mu = 0$.

Using notation originally introduced for the TKF91 model [50], for a given evolving sequence, one can abstract the concept of a residue and replace it by a "link" representing the presence of a residue (any residue). Introducing also the concept of a "immortal link" placed at the beginning of the evolving sequence representing the possibility of generating links not associated to any previously existing link, then, one can introduce the following quantities: $p_n(t)$ ($n \geq 1$) as the conditional probability that after time t n links are descendant from a normal link, and one of them is the original; ${p'}_n(t)$ ($n \geq 0$) as the conditional probability that after time t the original link disappears and n other links have been created; and ${p''}_n(t)$ ($n \geq 1$) as the conditional probability that the immortally link has n descendants including itself. For the gap-augmented model, these conditional probabilities are given by

$$p_n^{\varepsilon}(t) = p_1^{\varepsilon}(t)\,\xi_t^{n-1}, \quad n \geq 1, \tag{72}$$

$${p'}_n^{\varepsilon}(t) = {p'}_0^{\varepsilon}(t)\,\xi_t^{n}, \quad n \geq 0, \tag{73}$$

$${p''}_n^{\varepsilon}(t) = {p''}_0^{\varepsilon}(t)\,\xi_t^{n-1}, \quad n \geq 1, \tag{74}$$

where

$$
\begin{aligned}
p_1^{\varepsilon}(t) &= (1-\xi_t)\,(1-\gamma_t), && (75)\\
{p'}_0^{\varepsilon}(t) &= (1-\xi_t)\,\gamma_t, && (76)\\
{p''}_0^{\varepsilon}(t) &= (1-\xi_t), && (77)
\end{aligned}
$$

where the functions γ_t and ξ_t are given by Eqs. (52) and (55) respectively.

Although the actual time parameterization of the different functions is different, these conditional probabilities have many resemblances with those obtained for the TKF91 model using a birth-death process [50]. One important difference being that while for this model the probability of insertions is always linear (in log space), for the TKF91 model it is more complex than that since ${p'}_1^{\text{TKF91}}(t)$ is not proportional to ${p'}_0^{\text{TKF91}}(t)$.
