# Supplementary material for: Probabilistic Phylogenetic Inference with Insertions and Deletions
Source: PLoS Comput Biol. 2008 Sep 19;4(9):e1000172. doi: 10.1371/journal.pcbi.1000172 (PMC2527138; doi:10.1371/journal.pcbi.1000172)
Supplement: Text S3 — Appendix 3 (0.14 MB PDF) [file pcbi.1000172.s004.pdf]

Appendix 3: Length distributions and expected frequencies of insertions and deletions

The probability distribution of pairwise alignments of arbitrary length between an ancestral and a descendant sequence that include s substitutions, d deletions and i insertions after summing to all possible residues observed both in the ancestral and descendant sequence is given by:

$$P^{\varepsilon}_t(s,d,i) = (1-p)\,(1-\xi_t)\,\frac{(s+d+i)!}{s!\,d!\,i!}\,p^{s+d}\,(1-\xi_t)^{s+d}\,(1-\gamma_t)^s\,\gamma_t^d\,\xi_t^i, \tag{78}$$

where the functions γ_t, ξ_t are given by the conditional model (Eqs. (52) and (55)). Note that in order to have the proper normalization when summing to alignments of all possible lengths ($L = s + d + i$) with all possible number of substitutions (s), insertions (i) and deletions (d), $\sum_{s,d,i=0}^{\infty} P^{\varepsilon}_t(s,d,i) = 1$, we need to add the extra factor $(1-p)\,(1-\xi_t)$.

Other related probability densities can be obtained from $P^{\varepsilon}_t(s,d,i)$. The probability that sequences of $l = s + d$ residues generate alignments of length $\mathrm{L} \geq l$ after time t follows the distribution

$$P^{\varepsilon}_t(\mathrm{L},l) = (1-p)\,(1-\xi_t)\binom{\mathrm{L}}{l}\,[p\,(1-\xi_t)]^l\;\xi_t^{\mathrm{L}-l}. \tag{79}$$

The probability of generating sequences of $l = s + i$ residues for alignments of length $\mathrm{L} \geq l$ after time t follows the distribution

$$P^{\varepsilon\prime}_t(\mathrm{L},l) = (1-p)\,(1-\xi_t)\binom{\mathrm{L}}{l}\,[p\,(1-\xi_t)\,(1-\gamma_t)+\xi_t]^l\;[p\,\gamma_t\,(1-\xi_t)]^{\mathrm{L}-l}\,. \tag{80}$$

The distribution of ancestral sequences $P^{\varepsilon}_t(l) \equiv \sum_{\mathrm{L}=l}^{\infty} P^{\varepsilon}_t(\mathrm{L},l)$, and the distribution of descendant sequences $P^{\varepsilon\prime}_t(l) \equiv \sum_{\mathrm{L}=l}^{\infty} P^{\varepsilon\prime}_t(\mathrm{L},l)$, and the distribution of alignments $P^{\varepsilon}_t(\mathrm{L}) \equiv \sum_{l=0}^{\mathrm{L}} P^{\varepsilon}_t(\mathrm{L},l)$ are geometric and given by

$$P^{\varepsilon}(l) = (1-p)\,p^l, \tag{81}$$
$$P^{\varepsilon\prime}_t(l) = (1-q_t)\;q_t^l, \tag{82}$$
$$P^{\varepsilon}_t(\mathrm{L}) = (1-r_t)\;r_t^{\mathrm{L}}. \tag{83}$$

where the time-dependent Bernoulli frequency parameters for the distribution of descendant sequences and alignments are given by

$$q_t = \frac{\xi_t + p\,(1-\xi_t)\,(1-\gamma_t)}{1-p\,\gamma_t\,(1-\xi_t)} = \begin{cases} \xrightarrow{t\sim 0} p + (1-p)\,(\lambda - p\,\mu)\,t, \\ \xrightarrow{t\to\infty} \lambda\,\frac{\lambda+\mu\,(p+1)}{(\lambda+\mu)^2-\mu^2\,p}, \end{cases} \tag{84}$$

$$r_t \;=\; \xi_t + p\,(1-\xi_t) = \begin{cases} \xrightarrow{t\sim 0} p + (1-p)\,\lambda\, t, \\ \xrightarrow{t\to\infty} \frac{\lambda + p\,\mu}{\lambda+\mu}, \end{cases} \tag{85}$$

The distribution of descendant sequences is in general different from that of ascendant sequences (the model is non-reversible), and time dependent (then model is non-stationary). Reversibility would require to satisfy the condition $q_t = p$. One can see that there is not time-independent solution for $0 < p < 1$ that would make the model reversible and stationary unless one imposes the condition of no gaps (*i.e.* $\lambda = \mu = 0$).

The expected length of ancestral sequences $\langle l \rangle$, the expected length of descendant sequences $\langle l \rangle'_t$, and the expected length of an alignment $\langle \mathrm{L} \rangle_t$ are given by,

$$\langle l \rangle \;=\; \frac{p}{1-p}, \tag{86}$$

$$\langle l \rangle'_t \;=\; \frac{q_t}{1-q_t} = \frac{\xi_t + p\,(1-\xi_t)\,(1-\gamma_t)}{(1-p)\,(1-\xi_t)}, \tag{87}$$

$$\langle \mathrm{L} \rangle_t \;=\; \frac{r_t}{1-r_t} = \frac{\xi_t + p\,(1-\xi_t)}{(1-p)\,(1-\xi_t)}. \tag{88}$$

For small times τ, we obtain,

$$\langle l \rangle'_\tau \;=\; \frac{p}{(1-p)} + \frac{\lambda - p\,\mu}{1-p}\,\tau, \tag{89}$$

$$\langle \mathrm{L} \rangle_\tau \;=\; \frac{p}{(1-p)} + \frac{\lambda}{1-p}\,\tau. \tag{90}$$

At equilibrium, we obtain,

$$\langle l \rangle'_\infty \;=\; \frac{\lambda}{\mu}\frac{\lambda + \mu + p\,\mu}{(\lambda+\mu)\,(1-p)} = \begin{cases} 0 & : \quad \lambda = 0 \\ \infty & : \quad \mu = 0 \end{cases}, \tag{91}$$

$$\langle \mathrm{L} \rangle_\infty \;=\; \frac{\lambda + p\,\mu}{\mu\,(1-p)} = \begin{cases} \frac{p}{1-p} & : \quad \lambda = 0 \\ \infty & : \quad \mu = 0 \end{cases}. \tag{92}$$

That is, at equilibrium both expected lengths go to infinity if the deletion rate is zero, while the insertion rate is positive. On the other hand, if insertion rate is zero while the deletion rate is positive, the expected length of descendant sequences goes to zero, while the expected length of alignments is equal to the expected length of ancestral sequences. This behavior is only possible due to the non-reversibility of the model. For a reversible model, both ancestral and descendant sequences should have the same time-independent probability distribution.

Finally, the expected frequencies of insertions $f_i^\varepsilon(t)$, deletions $f_d^\varepsilon(t)$, and substitutions $f_s^\varepsilon(t)$ are given

by

$$f_i^{\varepsilon}(t) \equiv \frac{\langle \mathrm{L}\rangle_t - \langle l \rangle}{\langle \mathrm{L}\rangle_t} = \frac{\xi_t}{\xi_t + p\,(1-\xi_t)}, \tag{93}$$

$$f_d^{\varepsilon}(t) \equiv \frac{\langle \mathrm{L}\rangle_t - \langle l \rangle_t'}{\langle \mathrm{L}\rangle_t} = \frac{p\,\gamma_t\,(1-\xi_t)}{\xi_t + p\,(1-\xi_t)}, \tag{94}$$

$$f_s^{\varepsilon}(t) \equiv \frac{\langle l \rangle + \langle l \rangle_t' - \langle \mathrm{L}\rangle_t}{\langle \mathrm{L}\rangle_t} = \frac{p\,(1-\gamma_t)\,(1-\xi_t)}{\xi_t + p\,(1-\xi_t)}. \tag{95}$$

For very small times τ we have

$$f_i^{\varepsilon}(\tau) \sim \frac{\lambda}{p}\,\tau, \tag{96}$$

$$f_d^{\varepsilon}(\tau) \sim \mu\,\tau, \tag{97}$$

$$f_s^{\varepsilon}(\tau) \sim 1 - \left(\mu + \frac{\lambda}{p}\right)\tau, \tag{98}$$

which implies that at time strictly zero, both frequencies of insertions and deletions are zero.

At equilibrium we have

$$f_i^{\varepsilon}(\infty) = \frac{\lambda}{\lambda + p\,\mu} = \begin{cases} 0 & : \lambda = 0 \\ 1 & : \mu = 0 \end{cases}, \tag{99}$$

$$f_d^{\varepsilon}(\infty) = \frac{\mu}{\lambda + \mu}\,\frac{p\,\mu}{\lambda + p\,\mu} = \begin{cases} 1 & : \lambda = 0 \\ 0 & : \mu = 0 \end{cases}, \tag{100}$$

$$f_s^{\varepsilon}(\infty) = \frac{\lambda}{\lambda + \mu}\,\frac{p\,\mu}{\lambda + p\,\mu} = \begin{cases} 0 & : \lambda = 0 \\ 0 & : \mu = 0 \end{cases}. \tag{101}$$

which indicates that in the absence of insertions, any sequence of finite length will evolve to having all residues deleted. In the absence of deletions, for any sequence of finite length there will be an infinite number of insertions.

One technical point on how to obtain the previous results. All the previous calculations presented in these appendices make use of the sums for the following series, for $0 < p,\, q < 1$ and $l \geq 0$,

Binomial series ([87] p. 4):

$$\sum_{k=0}^{l} \binom{l}{k} p^k\, q^{l-k} = (p+q)^l, \tag{102}$$

$$\sum_{k=1}^{l} \binom{l}{k} k\, p^k\, q^{l-k} = p\,l\,(p+q)^{l-1}. \tag{103}$$

Geometric series ([87] p. 107):

$$\sum_{k=0}^{\infty} p^k = (1-p)^{-1}, \tag{104}$$

$$\sum_{k=1}^{\infty} k\, p^k = p\,(1-p)^{-2}. \tag{105}$$

Hyper-geometric series ([87] p.160):

$$\sum_{k=0}^{\infty} \frac{(l+k)!}{l!\,k!}\, p^k = (1-p)^{-(l+1)}, \tag{106}$$

$$\sum_{k=1}^{\infty} \frac{(l+k)!}{l!\,k!}\, k\, p^k = p\,(l+1)\,(1-p)^{-(l+2)}. \tag{107}$$
