# Supplementary material for: Probabilistic Phylogenetic Inference with Insertions and Deletions
Source: PLoS Comput Biol. 2008 Sep 19;4(9):e1000172. doi: 10.1371/journal.pcbi.1000172 (PMC2527138; doi:10.1371/journal.pcbi.1000172)
Supplement: Text S4 — Appendix 4 (0.13 MB PDF) [file pcbi.1000172.s005.pdf]

Appendix 4: Expected frequency of insertions and deletions for the TKF91 model

Here we calculate the density distribution of pairwise alignments generated by the TKF91 model, as well as the expected frequencies of insertions deletions and substitutions.

For the TKF91 model and for the regime $0 \leq \lambda \leq \mu$, the joint probability of a substitution is given by $\frac{\lambda}{\mu}\, p_1^t$; the joint probability of a lone deletion with no insertion associated to it is given by $\frac{\lambda}{\mu}\, q_0^t$; the joint probability of a deletion accompanied by one insertion is given by $\frac{\lambda}{\mu}\, q_1^t$; and the joint probability of any additional insertion either associated to a deletion or a substitution is given by β_t. The values of those functions depend on the rates of insertion (λ) and deletion (μ) and divergence time as

$$
\begin{aligned}
p_1^t &= (1-\beta_t)\, e^{-\mu\, t}, && (108)\\
q_1^t &= (1-\beta_t)\left(1 - e^{-\mu\, t} - \frac{\mu}{\lambda}\,\beta_t\right), && (109)\\
q_0^t &= \frac{\mu}{\lambda}\,\beta_t, && (110)\\
\beta_t &= \frac{\lambda\left(1 - e^{(\lambda-\mu)\, t}\right)}{\mu - \lambda\, e^{(\lambda-\mu)\, t}}. && (111)
\end{aligned}
$$

For an ancestral and a descendant sequence after time t, the density distribution of all pairwise alignments that contain s substitutions, d_0 lone deletions, d_1 deletions followed by at least one insertion, and i additional insertions (be those associated to an s or a d_1 event) is given by,

$$
P_t^{\text{TKF91}}(s, d_1, d_0, i) = \left(1 - \frac{\lambda}{\mu}\right)(1-\beta_t)\,\frac{(s+d_1+d_0)!}{(s+d_1)!\, d_0!}\,\frac{(s+d_1+i)!}{s!\, d_1!\, i!}\left(\frac{\lambda}{\mu}\, p_1^t\right)^{s}\left(\frac{\lambda}{\mu}\, q_1^t\right)^{d_1}\left(\frac{\lambda}{\mu}\, q_0^t\right)^{d_0}(\beta_t)^{i}, \tag{112}
$$

where the extra factor $\left(1 - \frac{\lambda}{\mu}\right)(1 - \beta_t)$ is required in order to have the proper normalization $\sum_{s,d_1,d_0,i=0}^{\infty} P_t^{\text{TKF91}}(s, d_1, d_0, i) = 1$. The length of the ancestral sequence is given by $s + d_1 + d_0$, the length of the descendant sequence is given by $s + d_1 + i$, and the length of the alignment of the two sequences is given by $L = s + 2\, d_1 + d_0 + i$.

Using this probability distribution, one can calculate other related distributions. For instance, the length distribution for ancestral sequences $P_t^{\text{TKF91}}(l)$ and the length distribution for descendant sequences $P_t'^{\text{TKF91}}(l)$ are given by

$$
\begin{aligned}
P_t^{\text{TKF91}}(l) &\equiv \sum_{s+d_1+d_0=l}\sum_{i=0}^{\infty} P_t^{\text{TKF91}}(s, d_1, d_0, i) = \left(1 - \frac{\lambda}{\mu}\right)\left(\frac{\lambda}{\mu}\right)^{l}, && (113)\\
P_t'^{\text{TKF91}}(l) &\equiv \sum_{s+d_1+i=l}\sum_{d_0=0}^{\infty} P_t^{\text{TKF91}}(s, d_1, d_0, i) = \left(1 - \frac{\lambda}{\mu}\right)\left(\frac{\lambda}{\mu}\right)^{l}. && (114)
\end{aligned}
$$

This shows that the TKF91 model is both stationary (a time-independent length distribution for evolved sequences) and reversible $[P'^{\text{TKF91}}(l) = P^{\text{TKF91}}(l)]$. The choice of the geometric probability parameter $\frac{\lambda}{\mu}$, together with the condition $\frac{\lambda}{\mu}\, q_0^t = \beta_t$ are responsible for the above result.

One can also calculate the expected number of substitutions, insertions and deletions, which are given by

$$\langle s\rangle_t = \frac{\lambda/\mu}{\left(1-\frac{\lambda}{\mu}\right)}\, e^{-\mu\, t}, \tag{115}$$

$$\langle d_1\rangle_t = \frac{\lambda/\mu}{\left(1-\frac{\lambda}{\mu}\right)} \left(1 - e^{-\mu\, t} - \frac{\mu}{\lambda}\,\beta_t\right), \tag{116}$$

$$\langle d_0\rangle_t = \frac{\beta_t}{\left(1-\frac{\lambda}{\mu}\right)}, \tag{117}$$

$$\langle i\rangle_t = \frac{\beta_t}{\left(1-\frac{\lambda}{\mu}\right)}. \tag{118}$$

Then, the expected length of an alignment $\langle \mathrm{L}\rangle_t$ is given by,

$$\langle \mathrm{L}\rangle_t = \langle (s + 2\, d_1 + d_0 + i)\rangle_t = \frac{\lambda/\mu}{\left(1-\frac{\lambda}{\mu}\right)}\,(2 - e^{-\mu\, t}). \tag{119}$$

The expected length of descendant sequences $\langle l\rangle'$ is time independent and identical to that of ancestral sequences $\langle l\rangle$,

$$\langle l\rangle' = \langle l\rangle = \frac{\lambda/\mu}{\left(1-\frac{\lambda}{\mu}\right)}. \tag{120}$$

For any pairwise alignment, the expected frequencies of insertions $f_i^{\mathrm{TKF91}}(t)$, deletions $f_d^{\mathrm{TKF91}}(t)$, and substitutions $f_s^{\mathrm{TKF91}}(t)$ are given by

$$f_i^{\mathrm{TKF91}}(t) \equiv \frac{\langle d_1\rangle_t + \langle i\rangle_t}{\langle \mathrm{L}\rangle_t} = \frac{1-e^{-\mu\, t}}{2-e^{-\mu\, t}}, \tag{121}$$

$$f_d^{\mathrm{TKF91}}(t) \equiv \frac{\langle d_1\rangle_t + \langle d_0\rangle_t}{\langle \mathrm{L}\rangle_t} = \frac{1-e^{-\mu\, t}}{2-e^{-\mu\, t}}, \tag{122}$$

$$f_s^{\mathrm{TKF91}}(t) \equiv \frac{\langle s\rangle_t}{\langle \mathrm{L}\rangle_t} = \frac{e^{-\mu\, t}}{2-e^{-\mu\, t}}. \tag{123}$$

As a consequence of the reversibility of the model the expected frequencies of insertions and deletions are identical, and they asymptote to $1/2$ at equilibrium. In addition, these probabilities are independent of the rate of insertions λ for any divergence time.
