# Supplementary material for: Probabilistic Phylogenetic Inference with Insertions and Deletions
Source: PLoS Comput Biol. 2008 Sep 19;4(9):e1000172. doi: 10.1371/journal.pcbi.1000172 (PMC2527138; doi:10.1371/journal.pcbi.1000172)
Supplement: Dataset S1 — Supplemental Material (24.89 MB GZ) [file pcbi.1000172.s001.gz › erate-supplement-R2/src/easel/documentation/figures/rnaseP-bsu-alignment.pdf]

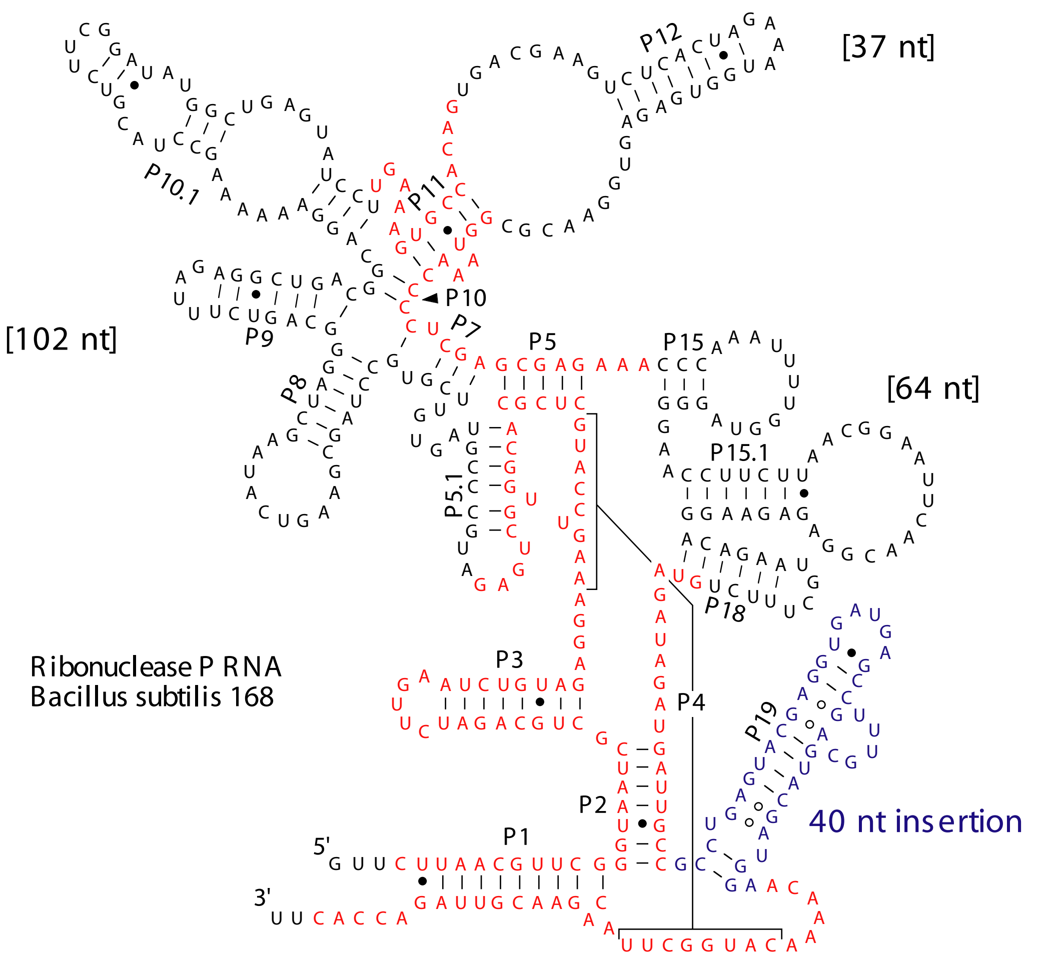
Ribonuclease P RNA
Bacillus subtilis 168
[37 nt]
[102 nt]
[64 nt]
40 nt insertion
P1
P2
P3
P4
P5
P5.1
P7
P8
P9
P10
P10.1
P11
P12
P15
P15.1
P18
P19
5'
3'
