# Supplementary material for: Probabilistic Phylogenetic Inference with Insertions and Deletions
Source: PLoS Comput Biol. 2008 Sep 19;4(9):e1000172. doi: 10.1371/journal.pcbi.1000172 (PMC2527138; doi:10.1371/journal.pcbi.1000172)
Supplement: Dataset S1 — Supplemental Material (24.89 MB GZ) [file pcbi.1000172.s001.gz › erate-supplement-R2/src/easel/documentation/figures/evd_basic.pdf]

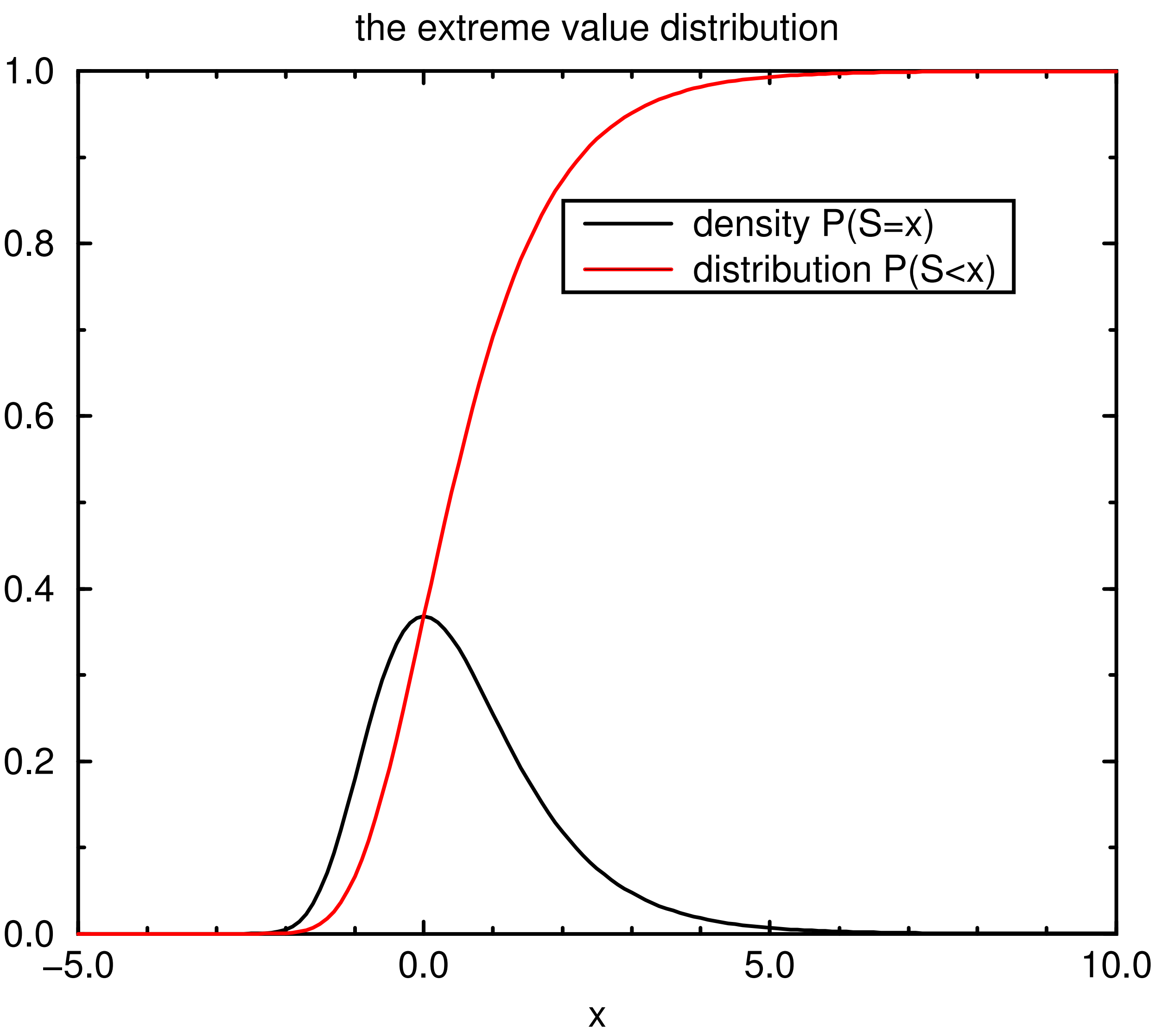

the extreme value distribution
density P(S=x)
distribution P(S<x)
1.0
0.8
0.6
0.4
0.2
0.0
–5.0
0.0
5.0
10.0
x
