# Supplementary material for: Probabilistic Phylogenetic Inference with Insertions and Deletions
Source: PLoS Comput Biol. 2008 Sep 19;4(9):e1000172. doi: 10.1371/journal.pcbi.1000172 (PMC2527138; doi:10.1371/journal.pcbi.1000172)

Ribonuclease P RNA
Escherichia coli K-12 W3110

Sequence : V00338, Reed, et al., 1982 Cell 30:627
Structure : Harris, et al., RNA (in press)

Image created 10/3/00 by JWBrown

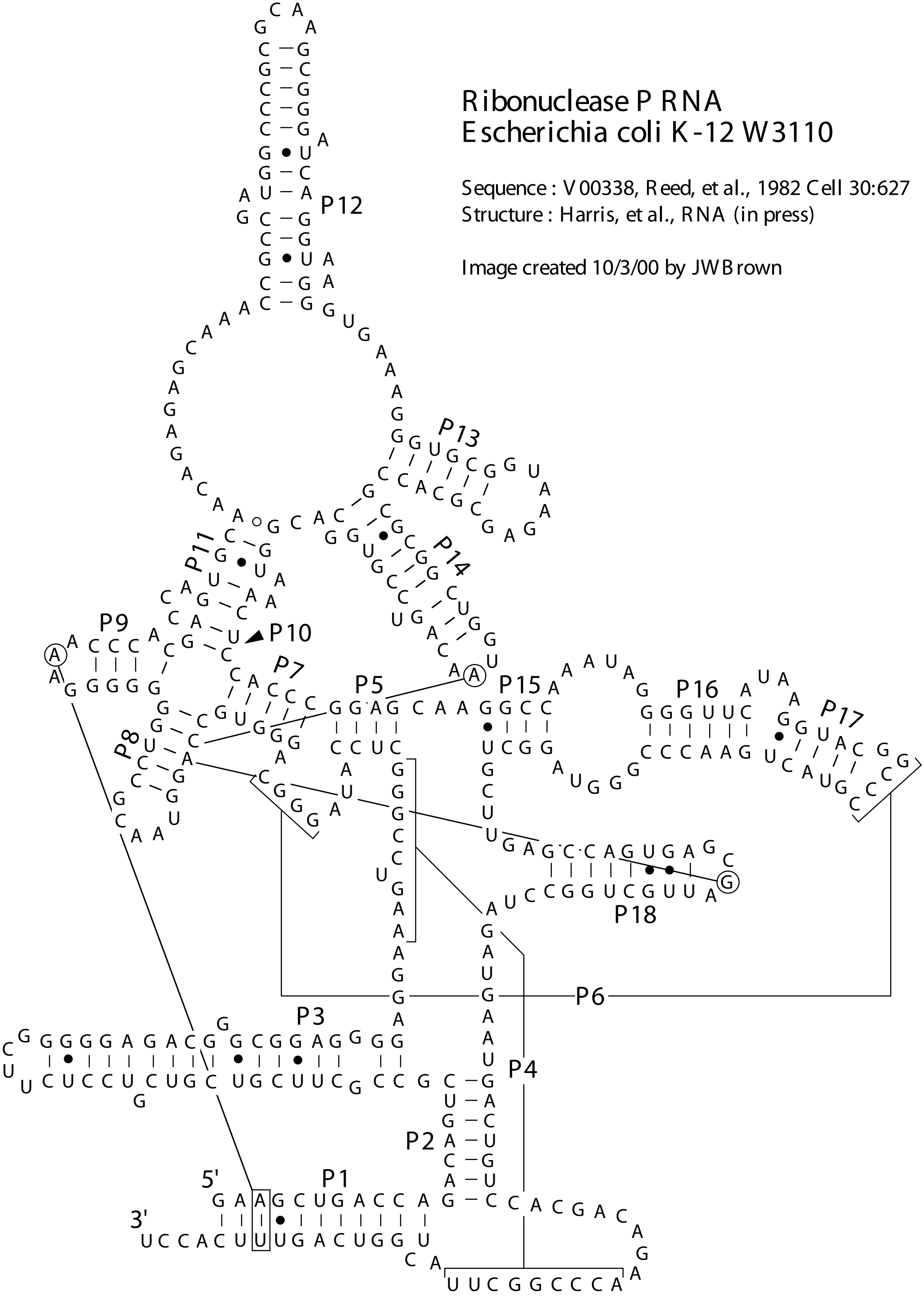

Supplement: Dataset S1 — Supplemental Material (24.89 MB GZ) [file pcbi.1000172.s001.gz › erate-supplement-R2/src/easel/documentation/figures/rnaseP-ecoli.pdf]
