# Supplementary material for: Probabilistic Phylogenetic Inference with Insertions and Deletions
Source: PLoS Comput Biol. 2008 Sep 19;4(9):e1000172. doi: 10.1371/journal.pcbi.1000172 (PMC2527138; doi:10.1371/journal.pcbi.1000172)
Supplement: Dataset S1 — Supplemental Material (24.89 MB GZ) [file pcbi.1000172.s001.gz › erate-supplement-R2/src/easel/documentation/figures/gev_logdensity.pdf]

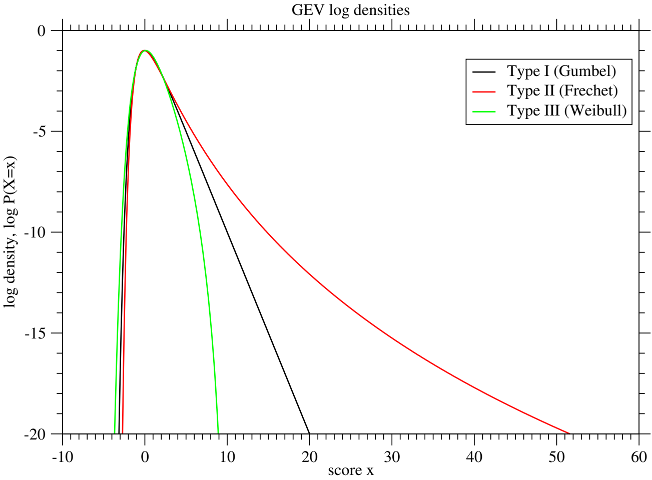

GEV log densities
0
-5
-10
-15
-20
log density, log P(X=x)
-10
0
10
20
30
40
50
60
score x
Type I (Gumbel)
Type II (Frechet)
Type III (Weibull)
