# Supplementary material for: Probabilistic Phylogenetic Inference with Insertions and Deletions
Source: PLoS Comput Biol. 2008 Sep 19;4(9):e1000172. doi: 10.1371/journal.pcbi.1000172 (PMC2527138; doi:10.1371/journal.pcbi.1000172)
Supplement: Dataset S1 — Supplemental Material (24.89 MB GZ) [file pcbi.1000172.s001.gz › erate-supplement-R2/src/easel/documentation/figures/evd_scale.pdf]

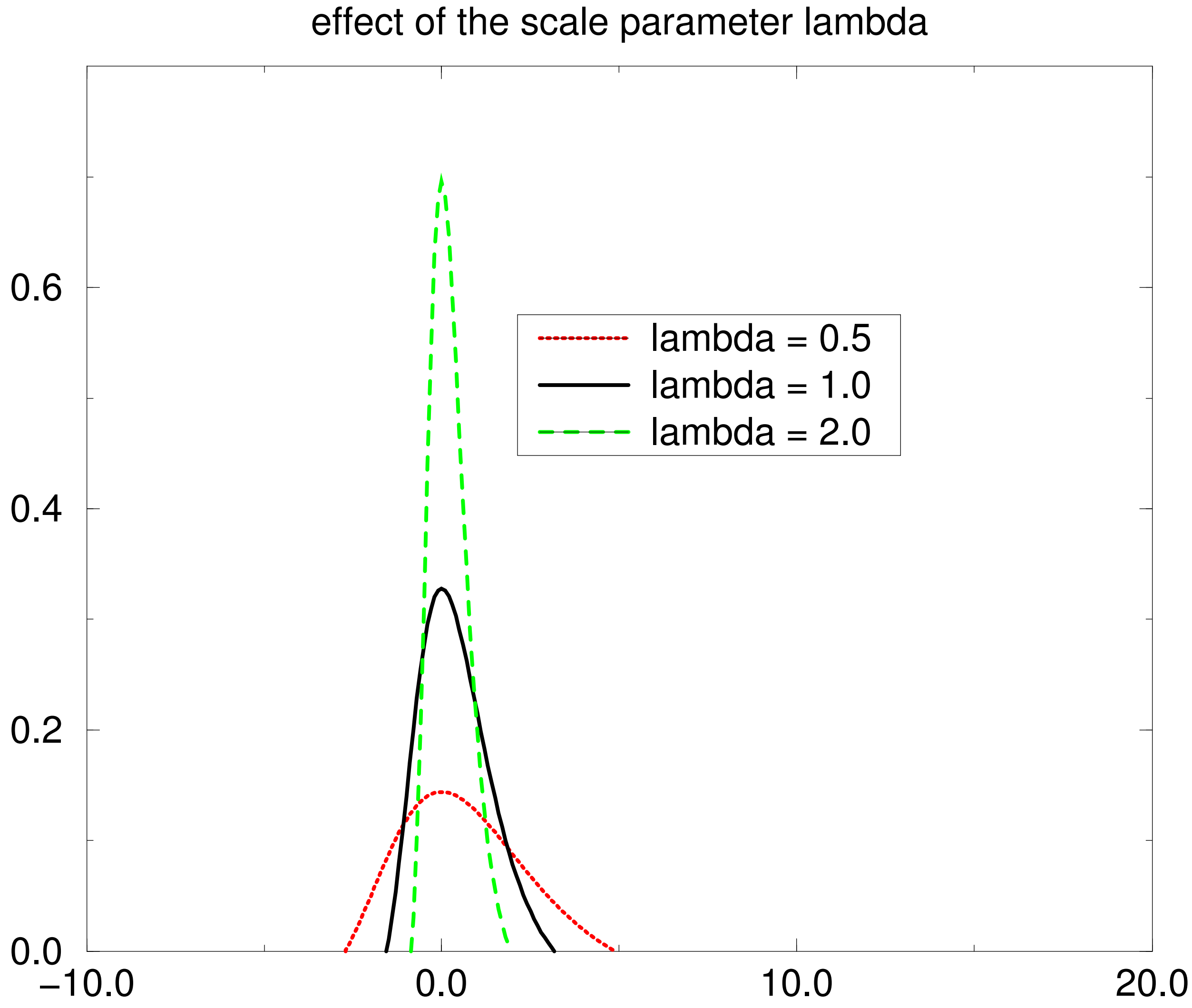
effect of the scale parameter lambda
lambda = 0.5
lambda = 1.0
lambda = 2.0
0.6
0.4
0.2
0.0
–10.0
0.0
10.0
20.0
