# Supplementary material for: Probabilistic Phylogenetic Inference with Insertions and Deletions
Source: PLoS Comput Biol. 2008 Sep 19;4(9):e1000172. doi: 10.1371/journal.pcbi.1000172 (PMC2527138; doi:10.1371/journal.pcbi.1000172)
Supplement: Dataset S1 — Supplemental Material (24.89 MB GZ) [file pcbi.1000172.s001.gz › erate-supplement-R2/src/easel/documentation/figures/evd_location.pdf]

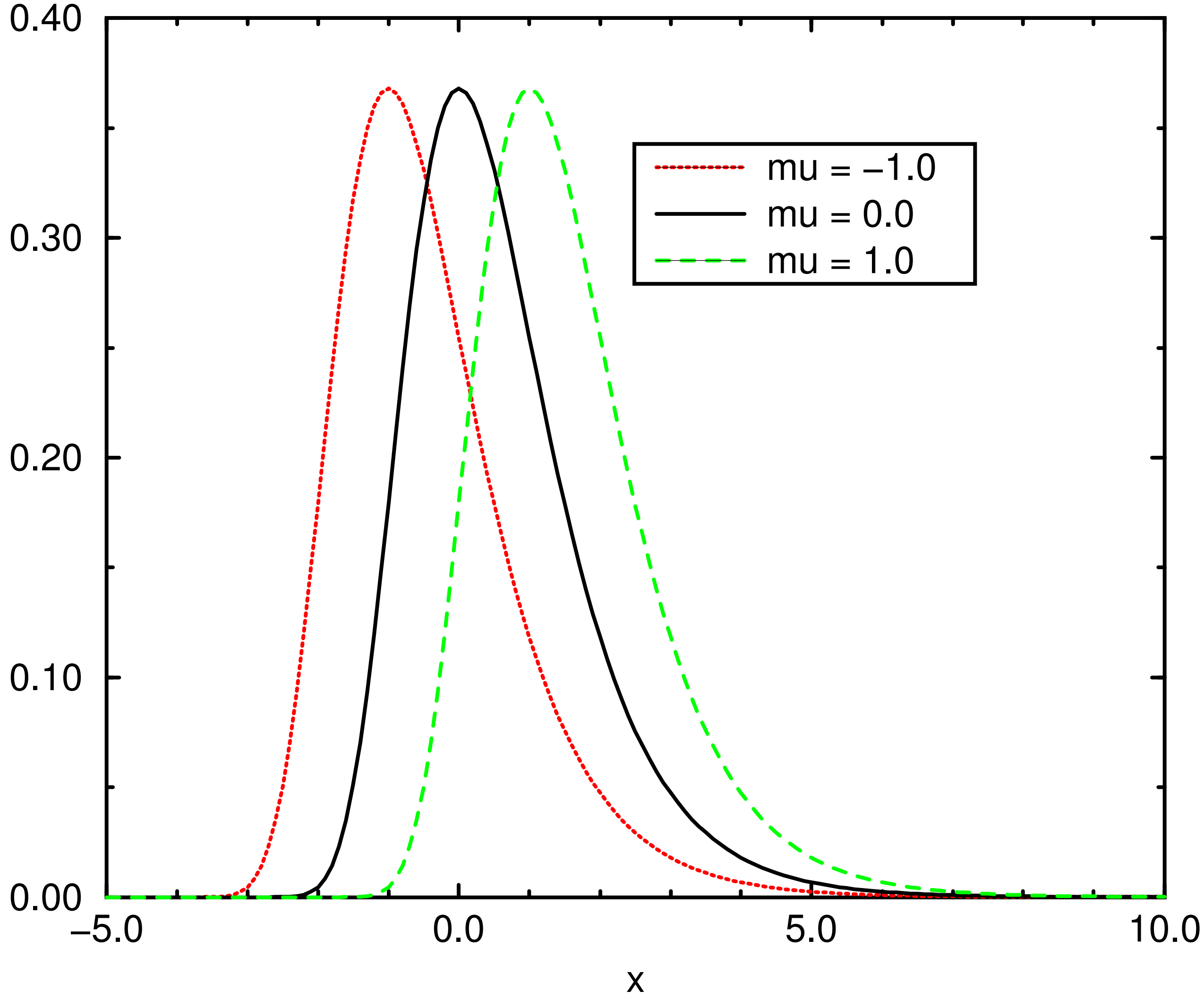

0.40
0.30
0.20
0.10
0.00
–5.0
0.0
5.0
10.0
x
mu = –1.0
mu = 0.0
mu = 1.0
